# A timed tally counter for microscopic examination of thick blood smears in malaria studies

**DOI:** 10.1186/s12936-020-03530-z

**Published:** 2021-01-05

**Authors:** Grégory Nuel, André Garcia

**Affiliations:** 1 Stochastics and Biology Group, Probability and Statistics (LPSM, CNRS 8001), Sorbonne University, Campus Pierre et Marie Curie, 4 Place Jussieu, 75005 Paris, France; 2 MERIT (IRD 261), University of Paris, Faculté de Pharmacie, 4 Avenue de l’Observatoire, 75006 Paris, France

**Keywords:** Malaria, Parasite density, Microscope, Quality control, Timed tally counter

## Abstract

**Background:**

Despite many technological advances for malaria parasite detection (e.g. high resolution image acquisition), microscopic reading of thick blood smear (TBS) remains the gold standard. Even though available in low technology environment, the microscopy of TBS is slow and time consuming. Moreover microscopy may induce errors at many levels and has no quality control.

**Methods:**

A electronic extension of the mechanical tally counter is proposed. In addition to the counting process it includes the process of counting itself that relies on the time elapsed between two successive pressures of the counting button leading to a timed tally counter (TTC). The microscopist performs the reading with the specific instruction starting by counting, in each high power fields, leucocytes first and then parasites. The time-stamp of all pressures of counting buttons are recorded along with the nature of the count. The data are recorded internally in CSV format and are exportable. The detection of HPFs locations and leukocyte/parasite counts per HPFs is performed through a hidden semi-Markov model (with outliers) allowing both to take into account the known distribution of leukocyte per HPFs (using a negative binomial distribution) and the pauses and hesitation of the microscopist during the reading. Parameters are estimated via the expectation-maximization algorithm. Hyper-parameters are calibrated using expert annotations. Forward/backward recursions are used to obtain the HPFs locations.

**Results:**

This approach provides richer data at no extra cost. It has been demonstrated that the method can derive parasites per HPF, leukocytes per HPF, and parasite/leukocyte ratio with robust non-parametric confidence intervals. Moreover a direct digital data entry leads to a less expensive process and decreased time-consuming and error-prone manual data entry. Lastly the TTC allows detecting possible protocol break during reading and prevents the risk of fraud.

**Discussion and conclusion:**

Introducing a programmed digital device in the data acquisition of TBS reading gives the opportunity to develop easily new (possible adaptive) reading protocols that will be easily followed by the reader since they will be embedded directly in the device. With the TTC the reader only has to read HPFs, counting leukocytes first and parasites second, and the counter will beep when the protocol is completed.

## Background

Malaria parasites can be identified by examining under the microscope a drop of the patient’s blood, spread out on a microscope slide [1]. Prior to examination, the specimen is stained by Giemsa. Microscopy of thick blood smears (TBSs) is the usual and most reliable diagnostic test for *Plasmodium falciparum* malaria and remains the gold standard for laboratory confirmation of malaria. Parasite density (PD), defined as the number of asexual parasites relative to a microlitre of blood, is classically assessed by counting parasites in a predetermined number of high power fields (HPFs) or by counting parasites according to a fixed number of leucocytes [2, 3]. The number of HPFs or of leucocytes and the protocols used during the counting process vary and depend on the study protocol [3]. The PD estimation and therefore the PD data-based inferences rely on the strong assumptions that the distribution of the thickness of the TBS, and hence the distribution of leucocytes and parasites within the TBS, is homogeneous. Under these assumptions parasites and leucocytes are evenly distributed and can be modeled by a Poisson distribution.

A great deal of literature exists concerning the validity of such hypotheses [4, 5] and we brought several arguments consistent with the real complexity of this question [3]. This last study showed that among simple parametric models the most appropriate is the negative binomial (NB) model which suggests that parasites and leucocytes tend to aggregate together. However, both the Poisson and the NB distributions impose some assumptions that need to be seriously assessed when statistical models for count data are constructed. Interestingly Dowling and Shute [4] had already noted in 1966 a phenomenon of “grouping”, in which parasites tend to aggregate together in a specific area of the smear. This spatial dependence between data has been explored through autocorrelation phenomenon by Hammami et al [3] by means of a simulation study. Under this hypothesis, on the basis of model selection criteria, hidden Markov models (HMMs) provided the better fit, including against mixtures models.

However, despite these results, most existing PD estimations methods assume homogeneity in the distribution of parasites and leucocytes in TBSs although this assumption clearly does not hold. Furthermore, despite the apparent importance of having data HPF by HPF and in an important number of HPFs, to the best of the autors’ knowledge, there is no simple and operational way to have such data in the context of epidemiological cohort follow-up with thousands of TBSs.

To take into account these constraints, the paper propose an adaptation of the classical tally counters used by microscopists to collect the counting of both leucocytes and parasites HPF by HPF. Furthermore the device is completed with the hability to measure the time elapsed between two successive counts of parasites or leucocytes.

The aim was to present the device, and the testing procedures together with the hypotheses we used. The paper will also demonstrate the enriched information that can be obtained with this timed tally counter as well as the control quality that can be associated with its use.

## Methods

### The timed tally counter

**Fig. 1:**
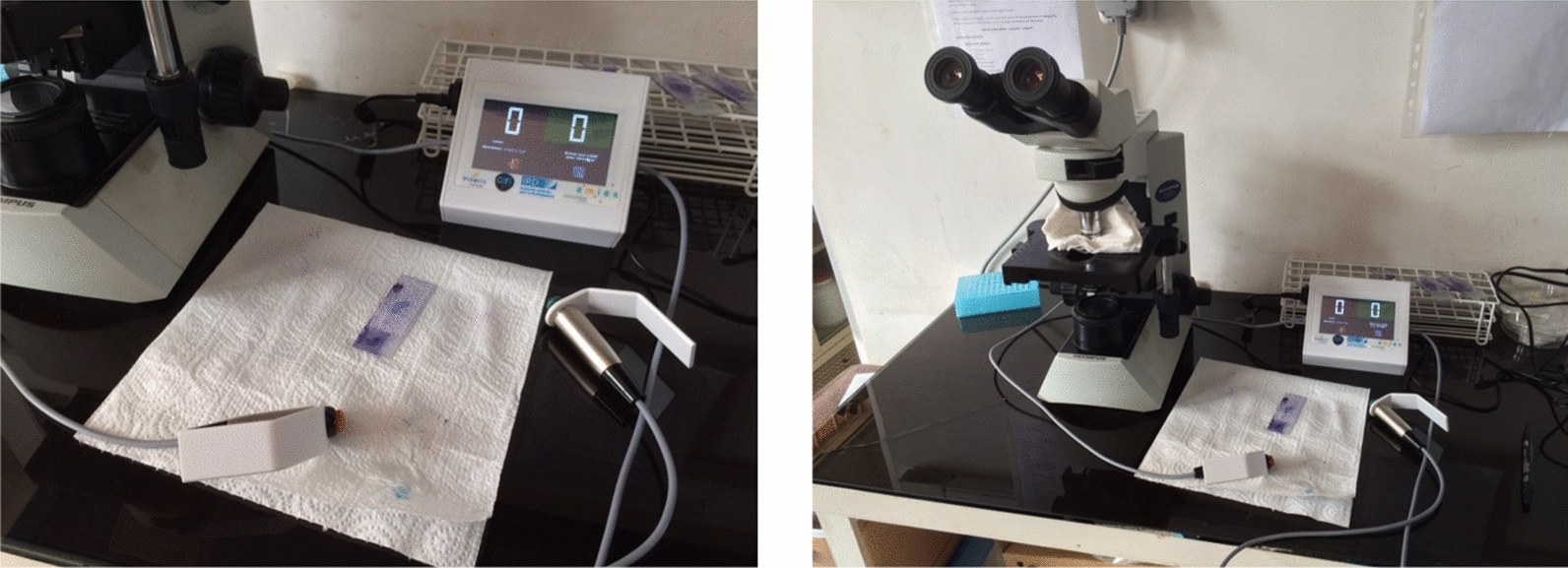
The timed tally counter. The left counter (Red button) counts the parasites and the right counter (Green button) counts the leukocytes. The current number of the two counts is displayed with the corresponding color on the LCD screen (here: 0 and 0 for the two buttons)

The device, jointly developed by the authors and Vivéris Technologies [6], is an electronic extension of the mechanical tally counter classically used for the counting of parasites in a TBS. This extension allows recording in addition to the counting, the process of counting itself. This process relies on the time elapsed between two successive pressures of the counting button leading to a timed tally counter (TTC). See Fig. 1 for a picture of the device.

The TTC is typically used by the microscopist as a replacement of the hand held pair of mechanical tally counter. The microscopist starts by entering the TBS identification number on the device, then performs the reading with the specific instruction to start by counting leucocytes and then parasites in each HPF, and finishes by ending the recording. During the reading, the time-stamp of all pressures of counting buttons are recorded along with the nature of the count (typically: leucocyte count or parasite count). The data are recorded internally in CSV format, and exported through a USB port when needed.

The tally counter in each hand was designed such as it was still possible for the microscopist to hold them while using the thumb and index to manipulate the microscope (typically moving the plate or adjusting the focus). This design was tested and validated by several trained microscopists.

This technical dispositive is protected by several patents under the following denomination and number respectively: “*Counting device for counting elements comprising a means for measuring time, and method for implementing same*” WO2015/052451.

### Experimental design

To test the characteristics of the TTC, TBS from a research programme conducted in southern Benin were used. A total of 9 high quality TBS were chosen by expert to cover the range of TBS processed. Three low parasitaemia TBS: A094, A100, OPT257; three medium parasitaemia: A098, OPT271, SL007; and three high parasitaemia: OPT211, SL/13, SL057. These TBS were read by a total of five different mircroscopists: A, B, C, D, and E. They were asked to read all the TBS once (B, C, D, E) and twice (A).

Leukocytes and parasites were counted simultaneously up to 200 leukocytes or 200 parasites, whichever came first. If no parasites were detected when 200 leukocytes had been counted the TBS was read up to 500 leukocytes (WHO, 2010).

On the total of $9 \times 6=54$ readings, 3 were lost during the experimental process (e.g. data corruption, wrong file identification), and 2 were excluded on quality control issues. This process ends up with a total 49 TBS readings (see Table 1).

**Table 1:** Experimental design

TBS	A	B	C	D	E
A094	0	1	0	1	0
A098	2	1	1	1	1
A100	2	1	1	1	1
OPT211	2	1	1	1	1
OPT257	2	1	1	1	1
OPT271	2	1	1	1	1
SL/13	2	1	1	1	1
SL007	2	1	0	1	1
SL057	2	1	1	1	1

Nine thick blood smears readings repartition among the five microscopists (denoted A, B, C, D, and E) after quality control

### The collected data

**Fig. 2:**
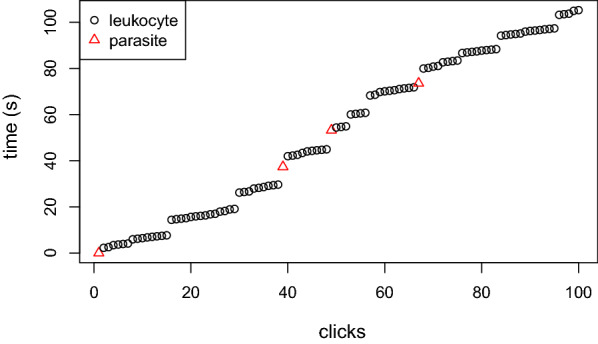
Raw data from A098 Microscopist B. 100 first clicks only

For each TBS and each reading, the collected data are: the identifier of the TBS, the identity of the reader, the reading date, a list of time stamps in second (s) with two digits and the type of button (G/R). e.g. 0.00s, G; 2.27s,R; 2.57s,R; 3.41s,R; etc. The red button (R) counts the parasites and the green (G) counts the leukocytes. One can see on Fig. 2 a graphical representation of these data. On the x-axis, the cumulative number of clicks, on the y-axis the cumulative time. Leukocyte counts are represented by black circles and parasite counts by red triangles. The typical “step-like” shape of the data corresponds to the different changes of HPFs and/or to unexpected pauses during the reading.

In principle, the elapsed time between two clicks is supposed to be short when the clicks correspond to items in the same HPF, and larger when the reader moved to a new HPF between two clicks. Unfortunately, there is many other reasons than a change of HPF for the reader to spend more time between to clicks: some distraction in the lab, a short break, or any visual identification issue (e.g.: TBS quality, form of a parasite, etc.). It is, therefore, necessary to build a model allowing such common behavior without erroneously taking them for HPF changes. To deal with this problem, one can take advantage of two additional facts: (1) if the TBS reading protocol ensures that leukocytes must be counted first in each TBS and then, and only after, parasites (if any), one knows for certain that a new TBS starts each time a leukocyte is counted after a parasite (step between the fourth and fifth series of clicks in Fig. 2); (2) the number of leukocytes in each TBS is supposed to follow a certain distribution (typically a negative binomial distribution with known parameters). If Information 1 is easy to take into account (under the optimistic, assumption that the protocol is strictly followed by the reader), Information 2 is much more difficult to incorporate into the model, since it requires to keep track of the number of leukocytes remaining in the HPF. This leads to a hidden semi-Markov model (HSMM) where the number of leukocytes in each HPF will follow an arbitrary distribution (e.g. a negative binomial with known parameters).

### The hidden Semi–Markov model (HSMM)

HSMM [7] are a generalization of the well known HMMs [8, 9] where the underlying unobserved Markov sequence is replaced by a semi-Markov process. The main advantage of HSMMs over HMMs is that the semi-Markovian component allows to specify prior number of leukocytes in a given HPF of arbitrary distribution (*e.g.* negative binomial) instead of the implicit geometric distribution of classical HMMs. In the case of our tally counter problem, this functionality is essential to take into account the known distribution of the number of leukocytes in a HPF.

**Fig. 3:**
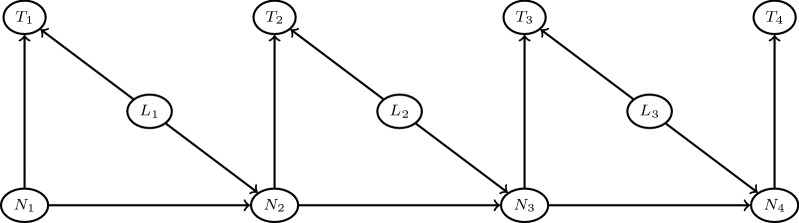
Dependence structure of the HSMM. $L_i\in \{0,1\}$ indicates if the click is a leukocyte count ($L_i=1$) or not ($L_i=0$). $T_i$ is the elapsed time (in seconds) since the last click. $N_i$ is the number of remaining leukocytes in the current HPF

Formally, one assumes a total of $i=1,\ldots ,n$ recorded clicks in the reading of the TBS. $L_i \in \{0,1\}$ indicates the nature of each click: $L_i=1$ if a leukocyte is counted, $L_i=0$ if it is a parasite. Let $T_i>0$ denotes the elapsed time between the previous click, with the convention that $T_1=0$, and let $N_i \in \mathbb {N}$ be the number of remaining leukocytes in the current HPF. When $N_i=0$, one knows that the next leukocyte count will correspond to a new HPF. The dependence relationships between these random variables are depicted in Fig. 3.

For the elapsed time between two leukocyte counts within the same HPF, one uses a mixture model with a (truncated) normal composant for standard elapsed time (between two leukocyte counts in the same HPF) and allowing for a prior proportion of outliers (for longer elapsed time due to other causes than a change of HPF – e.g. short pause, distraction, etc.) with a simple uniform distribution:

1 $$ {\mathbb{P}}(T_{i}  = t|N_{{i - 1}}  > 0,L_{i}  = 1) = f_{0} (t) = (1 - p) \texttt{dtnorm}(t,\tau ,\sigma ,0,\infty ) + \frac{p}{{T_{{{\text{max}}}} }} $$

where $p \in [0,1]$ is the proportion of outliers, $\texttt {dtnorm}$ denotes the truncated Gaussian density, $\tau$ is the mean, $\sigma$ the standard deviation, and $[0,\infty [$ is the truncation interval. For the elapsed time between the last count and the first count of a new HPF, the only constraint is that this elapsed time is not too short (e.g. greater than a $T_\text {min}$):

2 $$ {\mathbb{P}}(T_{i}  = t|N_{{i - 1}}  = 0,L_{i}  = 1) = f_{1} (t) = \texttt {dunif}(t,T_{{{\text{min}}}} ,T_{{{\text{max}}}} ) $$

where $\texttt {dunif}(\cdot ,a,b)$ is the density of the uniform distribution on the interval ]*a*, *b*[.

Finally, the number of leukocyte in a HPF (minus one) is supposed to follow a negative binomial distribution over the support $\{0,\ldots ,K-1\}$:

3 $$\begin{aligned} \mathbb {P}(N_i=j | N_{i-1}=0,L_i=1) = q_j \propto \texttt {dnbinom}(j,\mu ,r) \end{aligned}$$

where $\texttt {dnbinom}$ denotes the negative binomial density, $\mu$ is the size of the negative binomial distribution, and *r* the probability. The remaining conditional distributions only implement the protocol constraint that parasites are always counted after the leukocytes in a HPF gives: $\mathbb {P}(T_i=t | L_i=0)=\mathbb {P}(T_1=t)=1$, $\mathbb {P}(N_i=k | N_{i-1}=j, L_i=0)= \mathbb {1}_{k=j=0}$ (1 if $k=j=0$ and 0 else), and $N_n=0$. Finally, the fact that each leukocyte count within a HPF gives the following deterministic distribution: $\mathbb {P}(N_i=k | N_{i-1}=j, L_i=1)= \mathbb {1}_{(j-k)=1}$ (1 if $(j-k)=1$ and 0 else).

This model, therefore, has a total of five hyper-parameters: $p,\mu ,r,T_\text {min},K$ ($T_\text {max}$ is not really a parameters since it is taken directly from the data as the maximum elapsed time between to clicks); and a total of two parameters: $\tau , \sigma$ which are directly related to the microscopist speed of leukocyte counting on the current TBS (might depends on the TBS quality for example).

Note that the choice of a negative binomial prior distribution for the leukocyte counts per HPFs is both supported by the data collected and by the literature [10].

### HPFs Calling

**Fig. 4:**
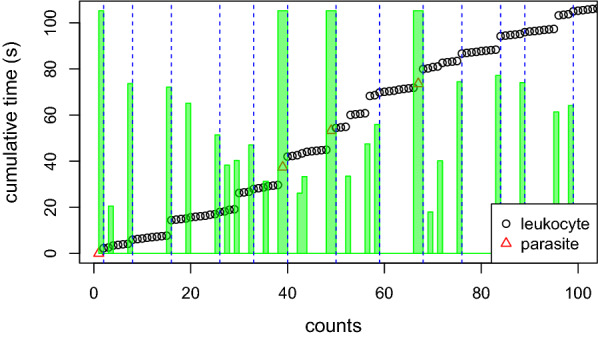
HPFs calling. Data from A098 Reader B. 100 first clicks only. HPFs calling using the HSMM. The green bars corresponds to the marginal posterior probability to reach $N_i=0$ (meaning that the next leukocyte count is in a new HPF). Vertical dashed blue lines correspond to HPFs calls (maximum a posteriori)

Since one knows that the last leukocyte count in a HPFs should correspond to $N_i=0$ in our model, one can compute the marginal posterior probability $\mathbb {P}(N_i=0 | \text {data})$ in each position. For calling the HPFs it is preferable to use maximum a posteriori (MAP) computation, in order to find the global configuration of HPFs starting/ending position which maximize the posterior distribution. On Fig. 4, the green line corresponds to the marginal posterior probability of ending a HPF, and the dashed blue lines correspond to the MAP HPF starting positions.

### Model Parameters

The parameters $\tau ,\sigma$ which correspond to the behavior/performance of the reader on a particular reading are locally estimated by maximum likelihood on each reading [*e.g.* using the well known EM algorithm [11] or by direct numeric optimization]. For calibrating our hyper- parameters $p,\mu ,r,T_\text {min},K$ one cannot rely on maximum likelihood estimation due to identification issues. A calibration dataset is hence designed with 5 samples of 100 consecutive counts each taken from 4 different TBS (low: A100, OPT257; medium: A098; high SL/13) and 4 sets of hyper-parameters. The two low-sensibility parameters are common to all sets ($T_\text {min} = 0.5$ and $K = 50$), two are chosen among two possible values ($p = 0.01$ or $p = 0.05$; $\mu = 10$ or $\mu = 15$ ), and the remaining one *r* is chosen such as 99% of the leukocyte count distribution is on the support [0, 30].

A total of $(5\times 4)\times 4=20\times 4=80$ graphical with HPFs callings (through the MAP approach described above) were submitted to a senior and experienced expert whose task was to confirm or correct the HPF callings by pointing out false positives and false negatives. Table 2 summarizes the results of this calibration experiment which led us to choose the hyper-parameter set $p = 0.05$ and $\mu = 10$. Even with this *optimal* parameter, the perfect agreement was only reached 11 times out to 20, but overall, with a false positives rate of $7.39\%$ and a false negative rate of $1.70\%$, the algorithm’s performances are certainly far from perfect but acceptable especially considering that the algorithm allows to obtain from the data a dramatically richer signal (*i.e.* leukocyte and parasite counts per HPF).

**Table 2:** Calibration. Comparisons between the HPF calling performed by the model and the expert decision. $$^{*}$$ HPF was detected only by the model but not confirm by the expert. $$^{**}$$ HPF was not detected by the model but detected by the expert only. $$^{***}$$ no FP nor FN on the whole sample

Calibration	False positive rate$$^{*}$$	False negative rate$$^{**}$$	Full agreement$$^{***}$$
$p=0.01$ $\mu =10$	20.18% (45/223)	1.35% (3/223)	9/20
$p=0.05$ $\mu =10$	7.39% (13/176)	1.70% (3/176)	11/20
$p=0.01$ $\mu =15$	11.67% (21/180)	2.78% (5/180)	9/20
$p=0.05$ $\mu =15$	9.21 % (14/152)	6.58 % (10/152)	11/20

## Results and discussion

**Fig. 5:**
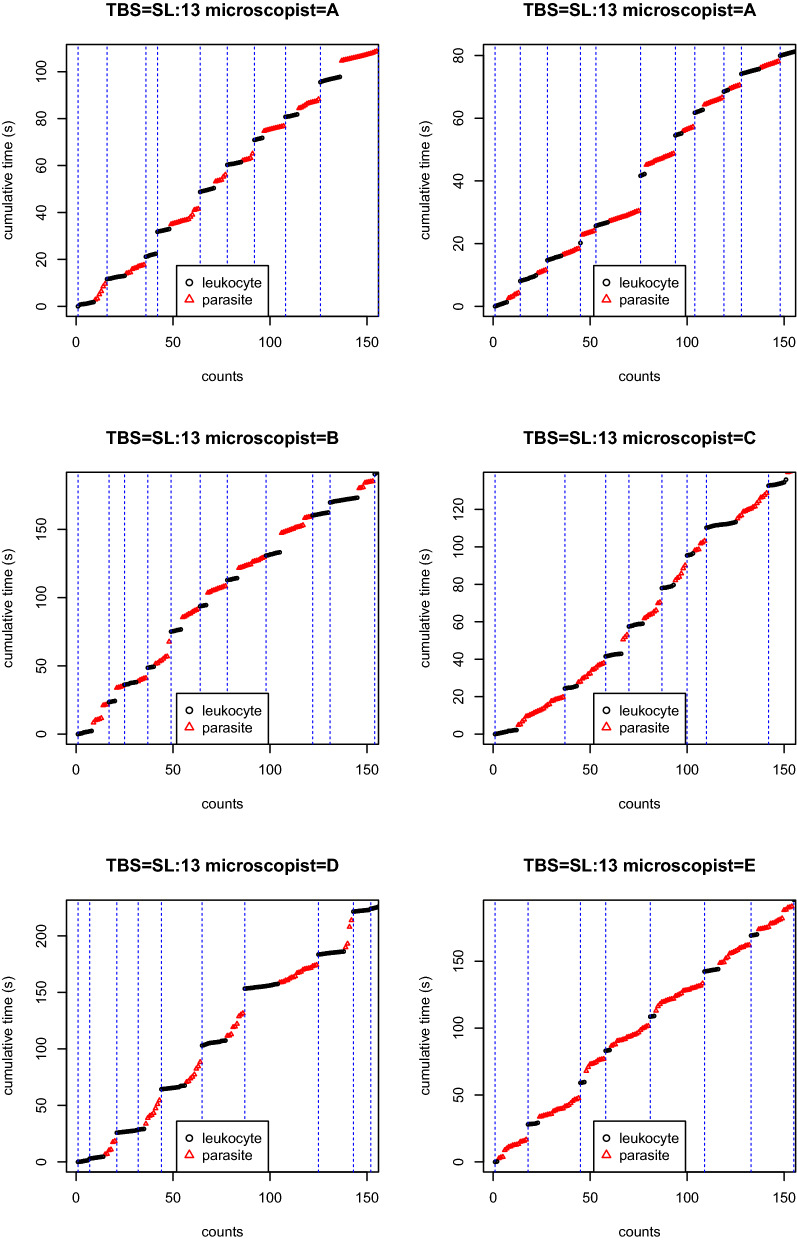
HPFs calling. First 150 readings of TBS SL/13. Vertical dashed blue lines correspond to HPFs calls (maximum a posteriori). The reading protocol was to read the TBS until reaching 200 leukocytes or 200 parasites, whichever comes the first

All TBS readings were processed through the model with calibrated hyper-parameter ($p = 0.05$ and $\mu = 10$) and got for each reading the count of leukocytes and parasites per HPF. See Additional file 1 and 2 for the whole dataset. See Table 3 for this data for the six readings of TBS SL:13 (medium parasitaemia). On Fig. 5 one can see that the number of read HPFs can differ between readings (including from the same reader—here A). In classical TBS reading, only the total number of leukocytes and parasites are reported, see the last row of Table 3. For the same work burden as classical readings, one gets much richer information with the per-HPF counts rather than global counts. For example, the first reading of reader A clearly ends with very few leukocytes per HPF which might be due to some heterogeneity of the local TBS leukocyte density and/or to a potential reader’s inattention.

**Table 3:** Count of parasite (Para) and leukocytes (Leuko) per HPF for each reader (A to E) for the TBS SL/13 (medium parasitaemia)

	Leuko A	Para A	Leuko A	Para A	Leuko B	Para B	Leuko C	Para C	Leuko D	Para D	Leuko E	Para E
HPF1	9	6	7	6	8	8	12	24	6	0	2	15
HPF2	10	10	9	5	4	4	7	14	8	6	6	21
HPF3	6	0	8	9	7	5	9	3	11	0	3	10
HPF4	7	15	1	7	4	8	8	9	4	8	3	20
HPF5	8	6	7	16	6	9	7	6	13	8	3	25
HPF6	8	6	3	15	4	10	4	6	13	9	8	16
HPF7	5	11	4	6	6	14	16	16	18	20	4	18
HPF8	7	11	5	10	8	16	10	9	14	4	3	17
HPF9	11	19	3	6	9	0	8	14	9	0	3	22
HPF10	3	7	10	10	15	8	8	0	9	8	6	15
HPF11	11	7	12	15	3	12	4	19	8	0	8	17
HPF12	12	0	7	12	6	0	8	19	4	19	8	18
HPF13	8	16	5	7	6	25	7	15	6	12	NA	NA
HPF14	6	5	10	14	2	3	5	12	13	11	NA	NA
HPF15	6	18	6	3	4	12	7	6	11	13	NA	NA
HPF16	9	11	11	7	9	7	12	3	15	15	NA	NA
HPF17	7	3	6	8	12	13	11	2	11	14	NA	NA
HPF18	5	0	11	9	8	7	10	4	11	19	NA	NA
HPF19	7	6	10	15	11	7	3	7	9	12	NA	NA
HPF20	7	0	8	15	13	11	3	9	9	20	NA	NA
HPF21	7	1	7	8	7	11	10	6	NA	NA	NA	NA
HPF22	6	4	NA	NA	10	3	12	2	NA	NA	NA	NA
HPF23	1	8	NA	NA	8	17	NA	NA	NA	NA	NA	NA
HPF24	12	5	NA	NA	12	0	NA	NA	NA	NA	NA	NA
HPF25	7	1	NA	NA	NA	NA	NA	NA	NA	NA	NA	NA
HPF26	4	1	NA	NA	NA	NA	NA	NA	NA	NA	NA	NA
HPF27	2	7	NA	NA	NA	NA	NA	NA	NA	NA	NA	NA
HPF28	4	5	NA	NA	NA	NA	NA	NA	NA	NA	NA	NA
HPF29	4	4	NA	NA	NA	NA	NA	NA	NA	NA	NA	NA
HPF30	6	6	NA	NA	NA	NA	NA	NA	NA	NA	NA	NA
total	205	199	150	203	182	210	181	205	202	198	57	214

The reading protocol was to read the TBS until reaching 200 leukocytes or 200 parasites, whichever comes the first. The “NA” entries are necessary to account for the different numbers of HPFs in the different readings

One can exploit the per-HPFs data to easily derive the mean number of leukocytes (respectively parasites) per HPF with empirical Confidence Interval (CI) by using a simple bootstrapping procedure using either one reading or combining several readings. The procedure is the following one: (1) pool together all pairs of (leukocyte, parasite) counts; (2) sample with replacement the same number of pairs from the pooled dataset; (3) repeat Step-2 a large number of times (e.g. 1000 times) and derive empirical quantiles or the mean (using median and 0.05/0.95 quantiles for 90% CI). For parasite/leukocyte ratio, we do the same procedure for the ratio of counts.

Usually, parasite density estimate are provided as simple numbers or ratio without any quantification of the estimation uncertainty. Even in the case where CIs are provided, they are always based on some questionable parametric assumption (e.g. Poisson or negative binomial distribution). With the new approach we suggest here one uses instead an empirical non-parametric estimate based on bootstrapping (resampling without replacement) which only assumption is that the observed counts (pairs of leukocyte and parasite counts per HPFs) are representative of the whole TBS.

In Table 4 one observes that the level of estimated parasitaemia are consistent with the selected TBS (low for the first three, medium for the following three, and high for the last four ones — see testing procedure section). In addition, the ratio estimated are given with 90% intervals providing some insight on the estimation uncertainty which is an interesting feature. Despite the fact that leukocytes and parasites should be distinguishable even in a case of high parasitaemia, one observes for OPT211 (with 40 parasites per leukocyte) that all the readers failed to count leukocytes in HPFs and report the low number of HPF reads (which hence results in a high estimate variability).

**Table 4:** Number of parasites per HPF and of Leukocytes per HPF with 90% empirical confidence Intervals for the nine TBS

TBS	Parasite median	90% CI	Leuko median	90% CI
A094	0.000	[0.000–0.000]	8.242	[7.762–8.812]
A100	0.000	[0.000–0.000]	12.792	[12.184–13.388]
OPT257	0.012	[0.004–0.023]	12.021	[11.445–12.617]
A098	0.298	[0.234–0.371]	7.587	[7.298–7.895]
OPT271	1.824	[1.406–2.315]	8.042	[7.382–8.764]
SL007	0.966	[0.771–1.203]	11.856	[11.076–12.755]
OPT211	105.667	[58.067–153.135]	2.625	[1.581–3.710]
SL:13	9.059	[8.214–10.022]	7.237	[6.733–7.778]
SL057	63.462	[37.023–94.890]	9.808	[7.308–12.538]

Table 5 contains the leukocytes per HPF and the parasite/leukocyte estimates of TBS SL/13 either for each reading or by pooling them (first line). The estimates are consistent between reading except for the reading of Microscopist E which might result from an atypical TBS region with low density of leukocyte and/or from a potential faulty reading. Unsurprisingly, CI range is smaller when pooling all the readings together.

**Table 5:** Leukocytes par HPF and Parasite/Leukocytes ratio estimates for the TBS SL/13

	Leuko median	90% CI	Parasite per leuko	90% CI
A	6.645	[5.742–7.484]	0.963	[0.740–1.213]
A	6.818	[5.727–7.955]	1.346	[1.140–1.602]
B	7.240	[6.120–8.480]	1.148	[0.847–1.509]
C	7.826	[6.609–9.087]	1.121	[0.838–1.444]
D	9.714	[8.143–11.095]	0.975	[0.739–1.258]
E	4.385	[3.308–5.462]	3.783	[3.034–4.870]
all	7.233	[6.719–7.749]	1.260	[1.114–1.414]

Fig. 5 contains the 150 first clicks of each of the six readings of TBS SL/13. Qualitatively, the four first readings are consistent which is also the case in Table 5. But the last two readings differ: with a slightly high leukocyte density in the reading Microscopist D (9.64 leukocytes per HPF while the range is $6.6-7.8$ for the four first ones), and a very low leukocyte density for the last reading from Microscopist E (4.31 leukocytes per HPF). This demonstrates how the data generated by the timed tally counter device might be useful both qualitatively and quantitatively for detecting discrepancies between readings.

**Fig. 6:**
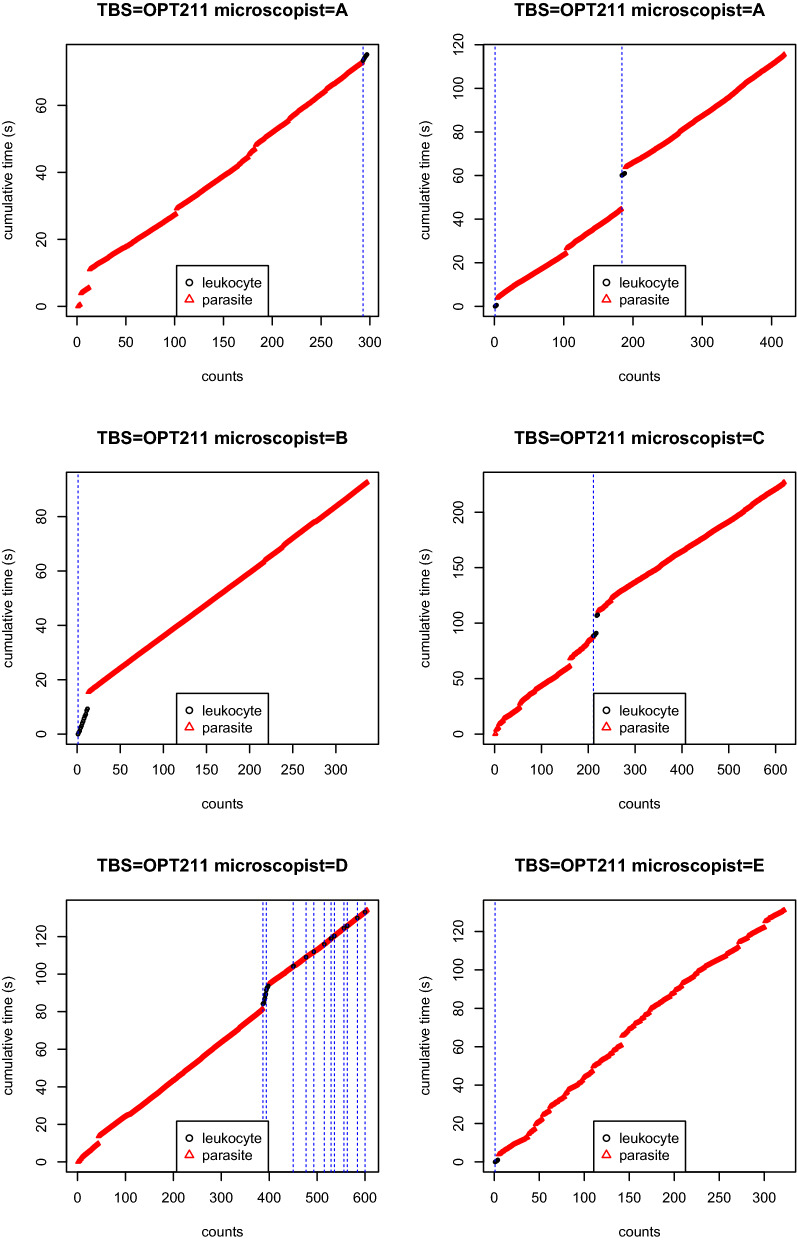
HPFs calling. First 150 readings of TBS OPT211. Vertical dashed blue lines correspond to HPFs calls (maximum a posteriori). The reading protocol was to read the TBS until reaching 200 leukocytes or 200 parasites, whichever comes the first

However, in most research project, systematic TBS reading replication are scarce (because time consuming and expansive). Quality control of isolated TBS reading is therefore a desirable feature which can be provided by the new device. Indeed, the rough data combined with HPF callings provide highly detailed information about how the reading protocol is applied by the microscopist. For exemple, in Fig. 6 one can see that all readings of TBS OPT211 somehow break the protocol: in the first reading from A, and the readings from C and D, the microscopist erroneously starts by counting the (very abundant) parasite instead of leukocytes; all readings but the one from D are done on only one or two HPFs. The multiple HPFs detected in the reading from D directly result from the alternance of sequences of leucocytes and parasites clicks. According to the protocol, such alternance must occur only once per HPF, but in the present case, it is likely that the microscopist did it multiple times in the same HPF, thus breaking the protocol and leading to inaccurate HPF detection. Since the protocol appears to have been followed correctly in most readings, the deviance to protocol observed in TBS OPT211 probably results from the extreme parasitaemia of the TBS and, therefore, highlight the possible inadequacy of the protocol for such extreme parasitaemia.

## Conclusions

This paper presented a new technical device called timed tally counter which is used as a replacement of mechanical tally counter used in microscopy’s lab for TBS reading. The device records not only the count of leukocytes and parasites during the reading but also the whole counting process. A semi-Markov Hidden Model was also introduced in order to process the raw data and identify HPFs, therefore providing the detailed number of leukocytes and parasites per HPFs without any additional burden for the reader.

This new approach has many advantages over the existing protocol: (1) it provides richer data at no extra cost; (2) one can easily derive density estimate (parasite per HPF, leukocyte per HPF, and parasite/leukocyte ratio) with robust non-parametric CI; (3) it allows a direct digital data entry by design and, therefore, avoid expensive, time consuming and error prone manual data entry; (4) it allows to detect possible protocol break during reading; (5) it prevents the risk of fraud.

Moreover, introducing a programmed digital device in the data acquisition of TBS reading offers the opportunity to develop easily new (possible adaptive) reading protocol that will be easily followed by the reader since they will be embedded directly in the device. For example, protocol where leukocytes, parasites and HPFs must be tracked separately with tree-shaped stopping criteria (e.g. if number of parasite is smaller than ...after reading ...HPFs then do ..., else read up to ...leukocytes) are difficult to follow with a classical tally counter. With the timed tally counter the reader only has to read HPFs counting leukocytes first and parasites second, and the counter will simply beep when the protocol is completed.

However, as pointed out during the calibration of the model for HPF detection, the current algorithm is able to process quickly the TBS data but at the cost both of false positives and false negatives. In order to avoid this source of error, it would be very useful to ask microscopists to count HPFs during the reading process. But since both microscopists hands are already used by tally counters, the adequate design remains to be found and the possible added work burden to be evaluated.

Despite many technological advances in the field of parasite detection in malaria (e.g. high resolution image acquisition and automatic treatment, molecular biology), microscopic reading of TBS remains the gold standard of parasite density estimation for the WHO. This might be motivated by the need to have a protocol available in low technology environment such as the ones where malaria epidemiology studies are typically conducted. However, the microscopy of TBS suffers from many shortcomings: 1) prone to error at many levels; 2) with little or no QC; 3) far outside the digital world. With the timed tally counter device, we provide a way to improve dramatically the microscopy of TBS without changing work protocols and habits, and opening a way to drive the microscopy-based parasite density estimation into the digital era.

## Supplementary Information

**Additional file 1.**

**Additional file 2.**

## Data Availability

The dataset supporting the conclusions of this article is available in the following data warehouse: https://dataverse.ird.fr/dataverse/umr_merit (IDxxxxx) .
