# Supplementary material for: A timed tally counter for microscopic examination of thick blood smears in malaria studies
Source: Malar J. 2021 Jan 5;20:6. doi: 10.1186/s12936-020-03530-z (PMC7786463; doi:10.1186/s12936-020-03530-z)
Supplement: Supplementary file 1 — Additional file 1. [file 12936_2020_3530_MOESM1_ESM.pdf]

Supplementary Material: Complete Raw and per HPFs Data

G. Nuel and A. Garcia

Contents

1 Raw data **2**
1.1 A094 . . . 3
1.2 A098 . . . 13
1.3 A100 . . . 24
1.4 OPT211 . . . 37
1.5 OPT257 . . . 50
1.6 OPT271 . . . 61
1.7 SL:13 . . . 72
1.8 SL007 . . . 80
1.9 SL057 . . . 91

2 Leukocyte and parasite counts per HPFs **100**
2.1 A094 . . . 101
2.2 A098 . . . 103
2.3 A100 . . . 105
2.4 OPT211 . . . 107
2.5 OPT257 . . . 108
2.6 OPT271 . . . 110
2.7 SL:13 . . . 112
2.8 SL007 . . . 113
2.9 SL057 . . . 114

1 Raw data

We here present the original data collected using the Timed Tally Counter. Elapsed time are expressed in seconds and the “leuko” columns indicates if the counting button pressed was or not the leukocyte button.

1.1 A094

Table 1: Raw data of TBS A094.

time B	leuko B	time D	leuko D
0.00	1	0.00	1
0.28	1	0.20	1
0.25	1	0.18	1
0.35	1	0.25	1
0.25	1	0.22	1
0.21	1	0.21	1
0.28	1	0.28	1
0.25	1	0.22	1
0.21	1	0.25	1
0.24	1	4.60	1
7.66	1	0.20	1
0.26	1	0.68	1
0.24	1	0.19	1
0.38	1	2.14	1
0.27	1	0.19	1
0.07	1	0.66	1
0.15	1	0.20	1
0.21	1	0.19	1
5.08	1	0.24	1
0.26	1	4.16	1
3.94	1	0.21	1
0.03	1	0.18	1
0.23	1	0.19	1
0.23	1	0.64	1
0.10	1	0.20	1
5.77	1	0.20	1
0.34	1	0.18	1
0.27	1	0.19	1
0.21	1	0.21	1
6.78	1	0.20	1
0.26	1	0.73	1
0.23	1	2.08	1
6.31	1	0.25	1
0.28	1	0.91	1
0.24	1	3.61	1
0.24	1	0.33	1
0.22	1	0.31	1
10.71	1	0.30	1
0.27	1	0.26	1
0.24	1	0.29	1
0.23	1	0.29	1
3.48	1	0.32	1
0.28	1	17.85	1
0.23	1	0.25	1
4.26	1	0.21	1
0.03	1	0.19	1
0.29	1	0.16	1
0.28	1	0.86	1
0.23	1	0.21	1

time B	leuko B	time D	leuko D
0.34	1	0.20	1
0.23	1	2.59	1
0.22	1	0.22	1
4.45	1	0.20	1
0.30	1	0.20	1
0.25	1	0.18	1
6.75	1	0.16	1
0.28	1	15.82	1
0.24	1	0.34	1
0.22	1	0.27	1
5.30	1	5.35	1
0.01	1	4.37	1
0.23	1	0.27	1
0.23	1	0.23	1
0.21	1	0.18	1
5.82	1	0.19	1
0.25	1	0.18	1
0.24	1	0.27	1
0.21	1	0.51	1
0.23	1	0.27	1
4.91	1	12.44	1
0.29	1	0.25	1
7.30	1	0.24	1
0.25	1	0.20	1
1.15	1	8.61	1
0.26	1	0.20	1
4.11	1	0.17	1
7.58	1	0.16	1
0.03	1	2.03	1
0.21	1	0.20	1
4.19	1	0.20	1
0.26	1	0.18	1
2.50	1	0.04	1
0.25	1	0.11	1
5.69	1	0.18	1
4.50	1	0.21	1
0.27	1	0.20	1
3.65	1	0.19	1
0.28	1	0.18	1
3.37	1	0.45	1
0.31	1	0.38	1
0.51	1	0.21	1
0.30	1	0.19	1
0.64	1	0.21	1
5.47	1	0.19	1
0.28	1	0.20	1
0.26	1	0.19	1
0.23	1	0.23	1
5.16	1	0.83	1
5.39	1	0.25	1
3.49	1	0.39	1
4.95	1	0.24	1

time B	leuko B	time D	leuko D
0.28	1	0.24	1
0.24	1	0.19	1
0.19	1	0.20	1
3.88	1	0.23	1
0.26	1	0.76	1
0.27	1	0.25	1
0.21	1	0.21	1
0.21	1	0.25	1
0.23	1	0.22	1
3.44	1	0.23	1
0.29	1	0.25	1
0.22	1	2.07	1
0.25	1	0.24	1
2.98	1	0.19	1
0.34	1	4.69	1
3.43	1	0.19	1
0.29	1	0.17	1
0.23	1	0.35	1
0.22	1	0.17	1
4.30	1	4.25	1
0.28	1	0.22	1
0.24	1	0.20	1
0.22	1	0.20	1
3.20	1	0.18	1
3.98	1	4.72	1
0.28	1	0.21	1
0.72	1	0.21	1
0.26	1	0.19	1
0.22	1	0.98	1
8.87	1	0.21	1
0.29	1	0.20	1
0.25	1	0.17	1
0.22	1	0.19	1
5.29	1	0.30	1
0.26	1	0.19	1
5.39	1	0.16	1
0.25	1	0.19	1
0.22	1	0.21	1
0.22	1	0.92	1
0.24	1	0.33	1
0.30	1	0.27	1
6.82	1	0.26	1
0.23	1	0.51	1
0.05	1	0.32	1
0.13	1	0.32	1
10.29	1	1.06	1
0.29	1	0.67	1
0.26	1	0.34	1
0.23	1	0.61	1
6.26	1	4.36	1
0.27	1	0.21	1
0.25	1	0.20	1

time B	leuko B	time D	leuko D
0.24	1	0.18	1
0.85	1	0.19	1
0.32	1	0.21	1
0.24	1	0.21	1
0.18	1	0.22	1
4.21	1	0.22	1
5.25	1	0.22	1
0.31	1	0.24	1
5.81	1	0.37	1
0.28	1	0.21	1
0.24	1	8.71	1
7.46	1	0.24	1
0.29	1	0.18	1
0.23	1	1.05	1
0.24	1	0.21	1
0.32	1	0.18	1
0.23	1	7.32	1
4.71	1	0.21	1
0.29	1	0.16	1
0.05	1	0.17	1
0.20	1	0.88	1
0.25	1	0.21	1
0.35	1	0.18	1
0.23	1	0.17	1
3.96	1	0.20	1
0.27	1	0.22	1
0.04	1	0.22	1
0.19	1	0.20	1
0.22	1	0.86	1
0.04	1	0.24	1
3.32	1	0.19	1
0.30	1	0.18	1
0.91	1	0.16	1
0.27	1	0.18	1
2.59	1	0.17	1
2.31	1	0.19	1
0.27	1	0.18	1
0.24	1	0.14	1
0.24	1	0.18	1
0.22	1	0.18	1
2.57	1	0.19	1
0.29	1	0.19	1
0.71	1	0.15	1
0.27	1	0.38	1
2.28	1	0.67	1
0.28	1	0.23	1
0.26	1	0.22	1
0.22	1	0.19	1
0.23	1	0.22	1
3.88	1	0.22	1
0.29	1	0.22	1
0.65	1	0.20	1

time B	leuko B	time D	leuko D
0.27	1	0.21	1
0.26	1	1.60	1
0.17	1	0.20	1
2.44	1	0.18	1
0.25	1	0.16	1
0.23	1	0.76	1
0.23	1	0.17	1
0.22	1	0.20	1
0.22	1	0.18	1
4.69	1	2.51	1
0.30	1	0.21	1
0.26	1	0.18	1
3.46	1	0.22	1
0.27	1	0.21	1
0.24	1	0.52	1
0.23	1	0.21	1
3.82	1	0.22	1
0.29	1	0.08	1
0.25	1	0.22	1
0.25	1	0.12	1
4.50	1	0.15	1
0.28	1	0.27	1
0.23	1	0.75	1
0.37	1	0.23	1
0.27	1	0.15	1
0.23	1	0.17	1
0.22	1	0.18	1
6.54	1	11.78	1
0.27	1	0.18	1
0.62	1	0.17	1
0.26	1	0.20	1
0.24	1	0.30	1
0.23	1	0.24	1
0.28	1	0.29	1
0.23	1	0.30	1
0.21	1	0.24	1
4.16	1	0.58	1
0.27	1	4.44	1
0.24	1	0.24	1
0.21	1	0.17	1
0.20	1	0.12	1
0.42	1	0.17	1
0.22	1	0.17	1
0.34	1	0.19	1
0.24	1	0.14	1
6.01	1	0.24	1
0.31	1	1.26	1
0.24	1	0.20	1
0.24	1	0.19	1
0.30	1	0.16	1
0.24	1	0.19	1
0.23	1	0.30	1

time B	leuko B	time D	leuko D
0.24	1	0.20	1
0.31	1	0.16	1
0.25	1	0.56	1
0.23	1	1.05	1
0.21	1	0.28	1
0.27	1	0.20	1
0.25	1	0.18	1
0.22	1	0.19	1
6.99	1	0.19	1
0.25	1	0.21	1
0.23	1	0.17	1
0.23	1	0.15	1
0.36	1	0.18	1
0.24	1	0.60	1
0.23	1	0.21	1
0.23	1	0.20	1
0.27	1	0.19	1
0.24	1	0.19	1
0.21	1	0.32	1
5.51	1	0.19	1
0.26	1	0.21	1
0.26	1	0.36	1
0.21	1	0.19	1
0.67	1	0.93	1
0.26	1	0.19	1
4.83	1	0.18	1
0.28	1	0.19	1
0.26	1	0.18	1
0.26	1	0.19	1
0.25	1	0.20	1
0.21	1	0.41	1
0.24	1	0.21	1
0.35	1	0.37	1
0.25	1	0.81	1
0.24	1	2.05	1
0.22	1	0.19	1
0.33	1	0.24	1
0.25	1	0.19	1
0.22	1	0.19	1
0.23	1	0.19	1
5.65	1	0.19	1
0.28	1	0.20	1
0.26	1	0.18	1
0.89	1	1.59	1
0.27	1	0.15	1
0.23	1	0.18	1
0.23	1	0.19	1
0.22	1	0.22	1
0.27	1	0.20	1
0.25	1	0.22	1
0.23	1	0.21	1
0.23	1	0.27	1

time B	leuko B	time D	leuko D
5.15	1	0.21	1
0.25	1	0.21	1
0.39	1	0.19	1
0.25	1	1.00	1
0.22	1	1.23	1
3.85	1	0.17	1
0.27	1	1.55	1
0.23	1	0.25	1
0.24	1	0.17	1
0.21	1	0.16	1
3.64	1	0.17	1
0.26	1	0.22	1
0.21	1	0.24	1
0.24	1	0.32	1
0.42	1	0.35	1
0.26	1	10.18	1
0.26	1	0.32	1
0.22	1	0.24	1
0.38	1	0.21	1
0.27	1	0.18	1
0.25	1	0.19	1
0.23	1	0.18	1
0.31	1	0.99	1
0.23	1	0.20	1
0.23	1	0.20	1
0.22	1	0.22	1
7.34	1	0.20	1
0.28	1	0.20	1
0.25	1	0.21	1
0.24	1	1.45	1
0.29	1	0.25	1
0.23	1	0.30	1
0.22	1	3.41	1
0.24	1	1.02	1
0.35	1	0.48	1
0.24	1	4.35	1
0.23	1	0.25	1
0.28	1	0.21	1
0.23	1	0.19	1
0.22	1	0.18	1
0.20	1	0.21	1
0.22	1	0.15	1
0.21	1	0.18	1
5.09	1	0.17	1
0.26	1	0.74	1
0.25	1	0.20	1
0.25	1	0.18	1
0.34	1	0.20	1
0.27	1	0.32	1
0.23	1	0.19	1
0.36	1	0.17	1
0.24	1	0.18	1

time B	leuko B	time D	leuko D
0.30	1	0.41	1
0.23	1	0.18	1
6.23	1	0.19	1
0.23	1	0.16	1
0.47	1	0.20	1
0.38	1	1.26	1
0.24	1	0.18	1
0.22	1	0.18	1
0.27	1	0.18	1
0.22	1	0.18	1
0.21	1	0.20	1
5.92	1	0.20	1
0.26	1	0.19	1
0.22	1	0.17	1
0.32	1	0.18	1
0.24	1	0.20	1
0.20	1	0.26	1
4.57	1	0.21	1
0.25	1	0.23	1
0.23	1	0.18	1
0.19	1	0.20	1
2.18	1	1.28	1
0.25	1	0.26	1
0.21	1	0.20	1
6.48	1	0.17	1
0.24	1	0.19	1
0.23	1	0.18	1
0.20	1	0.19	1
0.24	1	0.75	1
0.22	1	0.19	1
0.22	1	0.21	1
4.22	1	0.21	1
0.20	1	0.62	1
0.30	1	0.21	1
0.22	1	0.22	1
0.19	1	0.20	1
4.36	1	0.19	1
0.30	1	1.04	1
5.25	1	0.19	1
0.25	1	0.20	1
0.22	1	0.20	1
0.33	1	0.18	1
0.19	1	0.21	1
3.91	1	0.91	1
0.23	1	0.23	1
0.34	1	0.20	1
4.10	1	0.19	1
0.25	1	0.38	1
0.27	1	0.22	1
0.71	1	0.23	1
0.24	1	0.59	1
0.22	1	0.19	1

time B	leuko B	time D	leuko D
3.89	1	0.23	1
0.29	1	0.19	1
0.25	1	0.21	1
0.21	1	0.19	1
6.73	1	0.20	1
0.27	1	0.19	1
0.23	1	0.21	1
0.23	1	0.22	1
0.22	1	0.22	1
0.20	1	0.21	1
0.24	1	0.23	1
4.17	1	0.25	1
3.07	1	0.21	1
0.26	1	0.53	1
2.82	1	0.37	1
8.44	1	0.25	1
0.24	1	0.36	1
0.24	1	0.25	1
1.62	1	0.26	1
0.27	1	0.25	1
0.23	1	0.24	1
2.01	1	0.23	1
0.25	1	0.22	1
0.25	1	0.31	1
0.20	1	0.22	1
5.39	1	0.22	1
0.25	1	0.20	1
0.26	1	0.48	1
10.57	1	0.23	1
0.28	1	0.45	1
0.23	1	0.31	1
0.22	1	0.32	1
4.38	1	0.31	1
0.24	1	0.47	1
0.25	1	6.76	1
0.23	1	0.28	1
0.74	1	0.22	1
5.22	1	0.23	1
0.26	1	4.45	1
2.73	1	0.21	1
0.27	1	0.23	1
2.35	1	0.21	1
0.31	1	0.29	1
0.23	1	0.29	1
0.23	1	0.24	1
0.23	1	0.22	1
0.21	1	3.58	1
5.08	1	0.29	1
0.27	1	0.25	1
0.24	1	0.19	1
0.26	1	0.95	1
0.58	1	0.37	1

time B	leuko B	time D	leuko D
3.51	1	0.24	1
0.25	1	0.13	1
0.25	1	0.20	1
1.28	1	0.22	1
2.76	1	0.18	1
0.23	1	0.18	1
0.25	1	0.21	1
0.23	1	1.92	1
0.23	1	0.40	1
0.24	1	0.22	1
0.22	1	0.46	1
3.15	1	0.38	1
0.29	1	0.39	1
4.17	1	0.19	1
2.79	1	0.20	1
0.26	1	0.16	1
3.47	1	0.20	1
0.26	1	0.19	1
3.24	1	0.19	1
4.24	1	0.19	1
0.25	1	2.02	1
0.22	1	0.39	1
3.97	1	0.22	1
0.27	1	0.20	1
0.24	1	0.22	1
0.22	1	0.16	1
1.21	1	0.14	1
0.25	1	0.23	1
0.23	1	0.20	1
0.20	1	0.14	1
4.25	1	0.20	1
0.25	1	0.18	1
0.23	1	0.93	1
7.02	1	0.35	1
5.48	1	0.18	1
0.26	1	0.18	1
0.26	1	0.17	1
0.24	1	NA	NA
0.56	1	NA	NA
0.26	1	NA	NA
0.18	1	NA	NA

1.2 A098

Table 2: Raw data of TBS A098.

time A	leuko A	time A	leuko A	time B	leuko B	time C	leuko C	time D	leuko D	time E	leuko E
0.00	1	0.00	0	0.00	0	0.00	1	0.00	1	0.00	1
0.29	1	0.22	0	2.27	1	0.21	1	0.21	1	0.26	1
0.22	1	17.10	1	0.30	1	0.29	1	0.22	1	0.38	1
0.20	1	0.25	1	0.84	1	0.22	1	1.67	1	0.27	1
0.21	1	3.53	1	0.28	1	0.48	1	0.21	1	0.62	1
0.19	1	0.21	1	0.24	1	0.26	1	0.55	1	18.21	1
0.20	1	2.66	1	0.20	1	0.42	1	0.19	1	0.56	1
5.96	1	0.25	1	1.82	1	0.62	1	0.47	1	0.27	1
6.03	1	7.51	1	0.27	1	16.46	1	0.18	1	0.27	1
0.22	1	0.25	1	0.23	1	0.18	1	0.18	1	0.31	1
0.17	1	0.23	1	0.33	1	0.22	1	0.40	1	14.07	1
0.16	1	0.25	1	0.25	1	0.26	1	0.23	1	0.28	1
0.17	1	15.23	1	0.22	1	0.22	1	0.33	1	22.03	1
0.17	1	0.21	1	0.21	1	3.19	1	0.61	1	0.30	1
1.22	1	0.19	1	0.22	1	0.18	1	1.28	1	11.09	1
0.21	1	0.18	1	6.72	1	0.21	1	0.24	1	0.24	1
0.32	1	0.23	1	0.27	1	0.27	1	0.22	1	0.40	1
1.63	1	2.83	1	0.24	1	3.27	1	0.25	1	0.25	1
0.22	1	0.21	1	0.20	1	0.24	1	0.30	1	0.31	1
0.84	1	0.19	1	0.55	1	0.22	1	1.48	0	0.32	1
1.44	1	0.18	1	0.24	1	0.21	1	1.78	0	14.18	1
0.20	1	0.19	1	0.21	1	0.20	1	0.77	0	0.27	1
0.21	1	0.19	1	0.21	1	0.23	1	1.91	0	0.25	1
0.18	1	0.18	1	0.47	1	0.21	1	5.38	1	0.39	1
0.21	1	0.21	1	0.22	1	0.27	1	0.20	1	0.28	1
0.23	1	0.14	1	0.95	1	2.39	1	0.22	1	0.26	1
1.56	1	0.27	1	0.26	1	0.22	1	0.17	1	3.35	0
0.23	1	4.09	1	0.69	1	0.23	1	0.65	1	7.08	1
0.30	1	0.26	1	0.22	1	0.21	1	0.21	1	0.32	1
0.18	1	0.18	1	7.08	1	0.21	1	0.22	1	2.61	0
0.18	1	0.11	1	0.25	1	0.20	1	0.64	1	8.94	1
0.13	1	0.22	1	0.25	1	0.32	1	0.41	1	0.28	1
0.26	1	0.18	1	1.25	1	0.21	1	0.24	1	0.38	1
1.59	1	0.17	1	0.30	1	0.20	1	0.26	1	0.30	1
0.22	1	0.32	1	0.33	1	0.48	1	0.39	1	15.35	1
0.17	1	0.19	1	0.55	1	2.42	1	0.27	1	0.30	1
0.16	1	0.13	1	0.26	1	0.18	1	0.30	1	21.64	1
0.21	1	0.18	1	0.26	1	0.20	1	0.61	1	0.35	1
1.72	1	2.02	1	7.72	0	0.53	1	0.87	1	0.32	1
2.16	1	0.22	1	4.61	1	0.20	1	11.21	1	0.28	1
0.20	1	0.31	1	0.26	1	0.23	1	0.22	1	12.42	1
0.17	1	0.17	1	0.34	1	0.32	1	0.21	1	0.26	1
0.19	1	0.20	1	0.79	1	0.59	1	0.19	1	0.26	1
0.15	1	0.18	1	0.67	1	1.98	1	0.22	1	0.28	1
2.75	1	0.23	1	0.25	1	0.20	1	0.20	1	0.37	1
5.99	1	0.12	1	0.23	1	0.05	1	0.21	1	0.25	1
1.83	1	0.29	1	0.22	1	0.21	1	0.18	1	0.25	1
0.21	1	1.88	1	0.20	1	0.22	1	0.23	1	12.93	1

time A	leuko A	time A	leuko A	time B	leuko B	time C	leuko C	time D	leuko D	time E	leuko E
0.44	1	3.62	1	8.30	0	0.19	1	0.17	1	0.24	1
6.81	1	0.26	1	1.15	1	0.22	1	0.22	1	0.29	1
0.27	1	0.18	1	0.26	1	3.54	1	0.20	1	0.25	1
1.04	1	0.21	1	0.25	1	0.20	1	0.47	1	0.28	1
2.93	1	0.20	1	5.15	1	0.20	1	8.75	0	0.26	1
0.23	1	0.18	1	0.26	1	0.23	1	0.51	1	0.24	1
0.20	1	0.19	1	0.23	1	0.32	1	0.21	1	10.31	0
0.30	1	1.82	1	0.23	1	0.41	1	0.22	1	0.45	0
0.18	1	3.57	1	7.51	1	2.21	1	0.23	1	16.99	1
2.68	1	6.65	1	0.32	1	0.26	1	0.21	1	0.29	1
0.23	1	8.11	1	1.18	1	0.24	1	0.19	1	0.27	1
0.18	1	2.87	1	0.30	1	0.21	1	2.24	1	0.32	1
0.23	1	0.26	1	0.25	1	0.24	1	0.21	1	0.01	1
0.21	1	0.88	1	0.24	1	0.21	1	0.20	1	0.29	1
0.20	1	2.43	1	0.48	1	0.21	1	0.21	1	0.29	1
2.87	1	0.27	1	0.29	1	0.21	1	0.20	1	0.36	1
0.25	1	0.21	1	0.23	1	0.20	1	0.26	1	0.37	1
0.25	1	0.21	1	0.24	1	2.81	1	0.21	1	12.10	1
1.70	1	2.22	1	1.76	0	0.22	1	0.39	1	0.25	1
0.22	1	0.24	1	6.39	1	0.21	1	0.29	1	0.28	1
0.22	1	0.20	1	0.27	1	0.22	1	0.35	1	0.28	1
0.22	1	0.18	1	0.57	1	0.24	1	0.31	1	0.57	1
0.21	1	0.12	1	0.26	1	0.24	1	0.39	1	0.32	1
0.21	1	0.25	1	1.59	1	0.20	1	8.28	1	0.31	1
0.20	1	0.29	1	0.27	1	2.13	1	0.18	1	0.34	1
1.70	1	5.21	1	0.25	1	0.23	1	0.21	1	5.40	0
0.26	1	0.24	1	0.25	1	0.21	1	0.55	1	4.71	1
0.20	1	1.11	1	3.26	1	0.32	1	0.30	1	0.28	1
0.17	1	2.70	1	0.26	1	0.26	1	0.20	1	0.30	1
0.22	1	0.24	1	0.24	1	0.23	1	0.23	1	0.25	1
1.74	1	3.40	1	0.22	1	0.21	1	0.56	1	9.64	0
0.20	1	0.24	1	0.33	1	0.21	1	0.27	1	13.23	1
2.77	1	0.18	1	0.21	1	0.29	1	0.31	1	0.31	1
0.21	1	2.04	1	0.21	1	0.21	1	0.26	1	4.02	0
0.18	1	0.24	1	0.21	1	2.69	1	0.26	1	5.76	1
0.19	1	0.18	1	5.85	1	0.20	1	0.27	1	3.20	1
0.17	1	0.19	1	0.25	1	0.23	1	0.25	1	0.28	1
0.17	1	0.12	1	0.25	1	0.19	1	0.29	1	0.27	1
0.18	1	0.22	1	0.21	1	0.19	1	12.42	1	10.92	1
2.15	1	0.23	1	0.23	1	0.20	1	0.22	1	0.26	1
0.22	1	0.22	1	0.81	1	0.20	1	0.24	1	0.24	1
0.16	1	0.33	1	0.28	1	0.20	1	0.19	1	0.24	1
0.19	1	2.37	1	0.18	1	0.30	1	0.23	1	0.34	1
2.60	1	0.21	1	0.23	1	0.23	1	0.23	1	0.27	1
0.24	1	0.19	1	0.29	1	0.21	1	0.21	1	0.25	1
0.19	1	0.19	1	0.21	1	0.29	1	0.22	1	21.90	1
0.28	1	0.24	1	0.20	1	2.41	1	0.21	1	0.26	1
2.58	1	4.76	1	5.88	1	0.18	1	0.19	1	0.25	1
0.23	1	0.31	1	0.27	1	0.07	1	0.22	1	0.29	1
0.17	1	0.21	1	0.23	1	0.15	1	1.93	0	0.25	1
0.27	1	0.17	1	1.24	1	0.22	1	3.60	0	0.31	1

time A	leuko A	time A	leuko A	time B	leuko B	time C	leuko C	time D	leuko D	time E	leuko E
18.43	1	0.22	1	0.28	1	0.20	1	0.49	1	0.29	1
0.19	1	5.01	1	0.22	1	0.26	1	0.25	1	3.46	0
0.21	1	0.22	1	0.30	1	0.24	1	0.22	1	8.49	1
0.21	1	0.20	1	0.24	1	0.20	1	0.23	1	0.28	1
7.07	1	0.20	1	0.19	1	0.22	1	0.22	1	0.29	1
5.95	1	1.92	1	0.21	1	5.55	1	0.20	1	13.72	1
0.88	1	0.24	1	0.36	1	0.23	1	0.20	1	0.24	1
1.74	1	0.21	1	7.90	1	0.86	1	11.72	1	0.25	1
0.22	1	0.18	1	0.27	1	0.31	1	0.20	1	0.29	1
0.20	1	0.19	1	0.24	1	0.23	1	0.48	1	0.27	1
0.21	1	0.18	1	0.23	1	2.60	1	0.22	1	9.65	1
0.16	1	0.18	1	0.23	1	0.20	1	0.40	1	0.24	1
0.18	1	0.19	1	0.43	1	0.23	1	0.22	1	0.25	1
4.28	0	2.54	1	0.96	1	0.42	1	0.56	0	0.24	1
7.03	1	0.25	1	0.25	1	0.22	1	0.71	1	0.19	1
0.26	1	0.19	1	0.22	1	0.27	1	0.42	1	0.50	0
0.19	1	0.24	1	0.32	1	0.23	1	0.08	0	4.45	1
0.20	1	0.30	1	0.22	1	0.25	1	0.27	1	0.27	1
0.17	1	0.20	1	0.88	1	1.53	1	0.25	1	0.24	1
0.20	1	0.17	1	0.25	1	5.30	1	0.25	1	0.26	1
0.16	1	0.22	1	15.84	1	0.19	1	1.17	1	0.25	1
5.03	1	0.17	1	0.26	1	0.01	1	0.26	1	0.24	1
0.24	1	0.20	1	0.24	1	0.03	1	0.30	1	0.25	1
0.22	1	0.20	1	0.21	1	0.14	1	8.13	1	0.25	1
0.20	1	0.20	1	0.51	1	0.18	1	0.21	1	0.27	1
0.22	1	0.19	1	0.24	1	0.18	1	0.91	1	0.25	1
0.90	0	1.77	1	0.83	1	0.29	1	0.24	1	0.30	1
0.29	0	0.24	1	0.24	1	0.21	1	0.13	1	5.24	0
1.76	0	0.32	1	0.50	1	3.83	1	0.18	1	13.11	1
2.83	1	0.18	1	0.24	1	0.24	1	11.08	1	0.27	1
0.24	1	0.20	1	0.34	1	0.07	1	0.19	1	0.27	1
0.21	1	0.16	1	0.22	1	0.12	1	0.35	1	7.82	0
0.17	1	0.16	1	0.21	1	0.07	1	0.55	1	4.48	1
0.24	1	4.41	0	0.22	1	1.23	1	0.21	1	0.26	1
0.24	1	16.61	1	0.31	1	0.19	1	0.26	1	0.25	1
5.13	1	0.21	1	0.21	1	0.07	1	0.41	1	0.26	1
3.56	1	0.19	1	0.22	1	0.12	1	0.21	1	0.27	1
0.96	1	0.16	1	0.20	1	0.06	1	0.34	1	0.26	1
2.51	1	0.17	1	0.27	1	0.13	1	0.26	1	0.24	1
0.25	1	0.17	1	0.21	1	0.93	1	0.30	1	19.38	1
0.19	1	0.23	1	0.29	1	0.21	1	14.81	0	0.32	1
0.17	1	2.39	1	0.20	1	0.20	1	3.58	1	0.32	1
0.19	1	0.24	1	0.22	1	0.22	1	3.70	1	35.28	1
0.19	1	0.18	1	0.20	1	0.07	1	0.18	1	0.98	1
0.17	1	0.17	1	9.45	0	0.12	1	0.17	1	4.42	1
2.55	1	0.21	1	6.51	1	0.20	1	0.25	1	0.36	1
0.32	1	0.22	1	0.24	1	3.22	1	0.26	1	7.17	1
0.27	1	0.18	1	0.23	1	0.23	1	0.25	1	0.62	1
3.67	1	0.16	1	0.22	1	0.21	1	0.23	1	3.93	1
0.34	1	2.74	1	0.22	1	0.20	1	8.45	1	0.25	1
0.22	1	0.25	1	0.39	1	0.24	1	0.21	1	10.63	1

time A	leuko A	time A	leuko A	time B	leuko B	time C	leuko C	time D	leuko D	time E	leuko E
0.20	1	0.22	1	0.22	1	0.19	1	0.57	1	0.26	1
0.18	1	0.13	1	0.95	1	0.21	1	0.21	1	0.29	1
1.94	0	0.27	1	0.22	1	2.26	1	0.65	1	0.41	1
6.69	1	0.20	1	0.22	1	0.19	1	0.19	1	0.35	1
0.32	1	0.17	1	0.20	1	0.21	1	0.29	1	0.25	1
4.72	1	0.18	1	0.27	1	0.19	1	0.29	1	0.24	1
0.42	1	0.30	0	0.24	1	0.22	1	0.17	1	4.93	1
0.23	1	3.30	1	6.02	0	0.22	1	0.20	1	0.29	1
0.22	1	0.24	1	0.63	0	0.21	1	0.27	1	0.28	1
0.21	1	0.18	1	8.16	1	0.23	1	0.22	1	10.71	1
2.68	1	0.22	1	0.28	1	0.25	1	0.17	1	0.27	1
0.23	1	0.32	1	0.25	1	1.39	1	1.80	1	0.27	1
0.22	1	1.02	0	0.22	1	0.20	1	17.27	1	0.24	1
0.19	1	3.23	1	0.44	1	0.24	1	0.19	1	6.90	1
0.19	1	0.24	1	0.25	1	0.22	1	0.17	1	0.22	1
0.20	1	0.28	1	0.22	1	1.54	1	0.20	1	0.59	1
3.88	1	0.33	1	0.20	1	0.22	1	0.26	1	0.23	1
0.22	1	0.66	1	0.33	1	0.22	1	0.16	1	0.70	1
0.21	1	0.22	1	0.20	1	0.19	1	0.21	1	0.57	0
0.18	1	0.19	1	3.31	1	0.21	1	0.19	1	5.43	1
0.20	1	0.18	1	0.27	1	0.20	1	0.25	1	0.27	1
0.17	1	0.19	1	0.23	1	0.26	1	0.38	1	0.23	1
0.21	1	0.18	1	0.52	1	0.17	1	0.20	1	0.26	1
3.35	1	4.94	1	0.27	1	0.20	1	0.24	1	3.68	0
0.30	1	0.27	1	0.23	1	1.62	1	0.19	1	6.66	1
0.80	1	0.21	1	0.52	1	0.21	1	0.23	1	0.28	1
0.49	0	0.17	1	0.24	1	0.18	1	0.24	1	0.27	1
0.32	0	0.19	1	0.39	1	0.20	1	0.19	1	0.86	0
0.40	0	0.18	1	0.25	1	2.71	1	15.33	0	5.16	1
4.54	1	0.21	1	0.24	1	0.23	1	0.76	0	0.30	1
0.75	1	0.19	1	0.24	1	0.21	1	0.80	1	0.22	1
0.41	1	3.12	1	0.23	1	0.42	1	0.21	1	0.26	1
0.32	1	0.28	1	9.67	0	1.44	1	0.49	1	0.44	1
4.58	1	0.19	1	2.56	1	2.44	1	0.21	1	0.31	1
0.22	1	0.22	1	0.28	1	0.21	1	0.22	1	0.25	1
0.22	1	4.78	1	0.25	1	0.38	1	0.27	1	7.52	1
0.19	1	1.97	1	0.12	1	2.36	1	1.24	1	0.27	1
0.17	1	0.22	1	2.25	1	0.21	1	0.87	1	0.88	1
0.19	1	0.20	1	0.23	1	0.18	1	2.94	1	0.31	1
2.22	0	0.17	1	0.13	1	0.22	1	0.22	1	0.25	1
2.76	1	0.19	1	10.06	1	1.70	1	0.22	1	11.57	1
0.69	1	2.71	1	0.23	1	0.21	1	0.22	1	0.25	1
3.47	1	0.20	1	0.83	1	0.20	1	0.20	1	0.28	1
0.25	1	5.43	1	0.24	1	1.14	1	0.21	1	0.52	1
0.28	1	0.25	1	0.21	1	0.21	1	0.30	1	0.25	1
0.57	1	0.23	1	0.22	1	0.21	1	0.21	1	0.24	1
0.25	1	3.54	0	0.34	1	0.19	1	0.17	1	0.24	1
0.22	1	7.62	1	0.22	1	0.20	1	0.20	1	6.18	0
1.94	1	0.21	1	0.22	1	0.87	1	0.19	1	3.79	1
11.30	1	0.39	1	0.22	1	0.22	1	4.01	0	0.27	1
0.25	1	1.81	0	0.20	1	0.24	1	0.70	1	0.43	1

time A	leuko A	time A	leuko A	time B	leuko B	time C	leuko C	time D	leuko D	time E	leuko E
0.20	1	5.27	1	0.26	1	0.26	1	0.20	1	0.26	1
0.21	1	0.23	1	0.19	1	0.22	1	0.18	1	0.27	1
5.13	0	0.38	0	0.19	1	2.82	1	0.24	1	0.26	1
0.37	1	0.19	0	0.19	1	0.21	1	0.64	1	0.27	1
0.27	1	6.09	1	6.76	1	0.25	1	0.20	1	16.09	1
0.22	1	0.22	1	0.24	1	1.77	1	0.19	1	0.24	1
0.20	1	0.22	1	0.23	1	0.20	1	0.25	1	0.24	1
0.22	1	0.20	1	0.20	1	0.18	1	0.19	1	0.23	1
3.24	1	0.18	1	0.75	1	0.25	1	0.82	1	0.38	1
0.27	1	0.31	0	0.23	1	0.19	1	0.24	1	0.24	1
0.19	1	1.12	0	0.37	1	0.21	1	0.25	1	11.13	1
0.47	0	3.22	1	0.19	1	0.17	1	13.92	1	0.26	1
NA	NA	0.20	1	19.38	1	1.07	1	0.21	1	0.26	1
NA	NA	0.19	1	0.25	1	1.27	1	0.21	1	0.24	1
NA	NA	0.17	1	0.21	1	0.22	1	0.21	1	0.22	1
NA	NA	0.17	1	0.20	1	0.22	1	1.04	0	0.23	1
NA	NA	0.18	1	0.19	1	0.23	1	0.85	1	0.25	1
NA	NA	NA	NA	0.16	1	0.21	1	0.19	1	0.67	0
NA	NA	NA	NA	4.84	1	0.57	1	2.47	1	NA	NA
NA	NA	NA	NA	0.25	1	0.21	1	0.21	1	NA	NA
NA	NA	NA	NA	0.23	1	0.08	1	0.48	0	NA	NA
NA	NA	NA	NA	0.20	1	0.12	1	0.32	1	NA	NA
NA	NA	NA	NA	0.24	1	0.20	1	0.44	1	NA	NA
NA	NA	NA	NA	0.19	1	0.19	1	0.36	1	NA	NA
NA	NA	NA	NA	1.66	0	2.03	1	0.21	1	NA	NA
NA	NA	NA	NA	4.38	1	0.20	1	0.22	1	NA	NA
NA	NA	NA	NA	0.27	1	1.94	1	0.19	1	NA	NA
NA	NA	NA	NA	0.24	1	0.20	1	0.21	1	NA	NA
NA	NA	NA	NA	0.23	1	0.18	1	0.19	1	NA	NA
NA	NA	NA	NA	5.70	1	0.19	1	0.18	1	NA	NA
NA	NA	NA	NA	0.24	1	2.04	1	0.20	1	NA	NA
NA	NA	NA	NA	0.22	1	0.19	1	NA	NA	NA	NA
NA	NA	NA	NA	0.21	1	0.20	1	NA	NA	NA	NA
NA	NA	NA	NA	4.76	1	0.21	1	NA	NA	NA	NA
NA	NA	NA	NA	0.25	1	1.29	1	NA	NA	NA	NA
NA	NA	NA	NA	0.23	1	0.21	1	NA	NA	NA	NA
NA	NA	NA	NA	0.19	1	0.21	1	NA	NA	NA	NA
NA	NA	NA	NA	0.21	1	0.22	1	NA	NA	NA	NA
NA	NA	NA	NA	0.20	1	0.20	1	NA	NA	NA	NA
NA	NA	NA	NA	3.50	1	0.18	1	NA	NA	NA	NA
NA	NA	NA	NA	0.22	1	0.22	1	NA	NA	NA	NA
NA	NA	NA	NA	0.57	1	0.23	1	NA	NA	NA	NA
NA	NA	NA	NA	0.19	1	0.39	1	NA	NA	NA	NA
NA	NA	NA	NA	0.29	1	0.61	1	NA	NA	NA	NA
NA	NA	NA	NA	0.22	1	6.04	1	NA	NA	NA	NA
NA	NA	NA	NA	0.19	1	0.25	1	NA	NA	NA	NA
NA	NA	NA	NA	0.20	1	0.23	1	NA	NA	NA	NA
NA	NA	NA	NA	5.61	1	0.09	1	NA	NA	NA	NA
NA	NA	NA	NA	0.24	1	0.19	1	NA	NA	NA	NA
NA	NA	NA	NA	0.23	1	0.56	1	NA	NA	NA	NA
NA	NA	NA	NA	0.21	1	0.21	1	NA	NA	NA	NA

time A	leuko A	time A	leuko A	time B	leuko B	time C	leuko C	time D	leuko D	time E	leuko E
NA	NA	NA	NA	0.57	1	0.95	1	NA	NA	NA	NA
NA	NA	NA	NA	0.25	1	0.22	1	NA	NA	NA	NA
NA	NA	NA	NA	0.32	1	0.24	1	NA	NA	NA	NA
NA	NA	NA	NA	0.22	1	0.24	1	NA	NA	NA	NA
NA	NA	NA	NA	6.49	1	0.23	1	NA	NA	NA	NA
NA	NA	NA	NA	0.26	1	0.26	1	NA	NA	NA	NA
NA	NA	NA	NA	0.23	1	0.47	1	NA	NA	NA	NA
NA	NA	NA	NA	0.21	1	1.61	1	NA	NA	NA	NA
NA	NA	NA	NA	0.42	1	0.22	1	NA	NA	NA	NA
NA	NA	NA	NA	0.22	1	0.23	1	NA	NA	NA	NA
NA	NA	NA	NA	3.39	1	0.32	1	NA	NA	NA	NA
NA	NA	NA	NA	0.24	1	0.37	1	NA	NA	NA	NA
NA	NA	NA	NA	0.55	1	0.19	1	NA	NA	NA	NA
NA	NA	NA	NA	0.22	1	0.21	1	NA	NA	NA	NA
NA	NA	NA	NA	0.55	1	0.29	1	NA	NA	NA	NA
NA	NA	NA	NA	0.19	1	0.32	1	NA	NA	NA	NA
NA	NA	NA	NA	3.20	0	2.71	1	NA	NA	NA	NA
NA	NA	NA	NA	3.01	1	0.19	1	NA	NA	NA	NA
NA	NA	NA	NA	3.58	0	0.22	1	NA	NA	NA	NA
NA	NA	NA	NA	0.92	1	0.20	1	NA	NA	NA	NA
NA	NA	NA	NA	0.28	1	0.21	1	NA	NA	NA	NA
NA	NA	NA	NA	0.22	1	0.22	1	NA	NA	NA	NA
NA	NA	NA	NA	0.62	1	0.31	1	NA	NA	NA	NA
NA	NA	NA	NA	0.25	1	0.23	1	NA	NA	NA	NA
NA	NA	NA	NA	0.22	1	0.22	1	NA	NA	NA	NA
NA	NA	NA	NA	0.49	1	0.08	1	NA	NA	NA	NA
NA	NA	NA	NA	0.25	1	0.20	1	NA	NA	NA	NA
NA	NA	NA	NA	3.74	1	0.29	1	NA	NA	NA	NA
NA	NA	NA	NA	0.24	1	0.11	1	NA	NA	NA	NA
NA	NA	NA	NA	0.25	1	0.15	1	NA	NA	NA	NA
NA	NA	NA	NA	0.22	1	0.21	1	NA	NA	NA	NA
NA	NA	NA	NA	6.48	1	0.19	1	NA	NA	NA	NA
NA	NA	NA	NA	0.27	1	1.70	1	NA	NA	NA	NA
NA	NA	NA	NA	0.23	1	0.07	1	NA	NA	NA	NA
NA	NA	NA	NA	0.22	1	0.10	1	NA	NA	NA	NA
NA	NA	NA	NA	0.27	1	0.22	1	NA	NA	NA	NA
NA	NA	NA	NA	0.22	1	0.26	1	NA	NA	NA	NA
NA	NA	NA	NA	0.21	1	0.24	1	NA	NA	NA	NA
NA	NA	NA	NA	0.20	1	0.21	1	NA	NA	NA	NA
NA	NA	NA	NA	0.26	1	0.22	1	NA	NA	NA	NA
NA	NA	NA	NA	5.00	1	0.42	1	NA	NA	NA	NA
NA	NA	NA	NA	0.25	1	0.20	1	NA	NA	NA	NA
NA	NA	NA	NA	0.73	1	0.98	1	NA	NA	NA	NA
NA	NA	NA	NA	0.22	1	0.23	1	NA	NA	NA	NA
NA	NA	NA	NA	3.27	1	0.21	1	NA	NA	NA	NA
NA	NA	NA	NA	0.25	1	0.21	1	NA	NA	NA	NA
NA	NA	NA	NA	0.24	1	0.20	1	NA	NA	NA	NA
NA	NA	NA	NA	0.20	1	0.22	1	NA	NA	NA	NA
NA	NA	NA	NA	0.20	1	0.36	1	NA	NA	NA	NA
NA	NA	NA	NA	10.02	1	0.07	1	NA	NA	NA	NA
NA	NA	NA	NA	0.27	1	0.14	1	NA	NA	NA	NA

time A	leuko A	time A	leuko A	time B	leuko B	time C	leuko C	time D	leuko D	time E	leuko E
NA	NA	NA	NA	0.23	1	0.17	1	NA	NA	NA	NA
NA	NA	NA	NA	0.28	1	1.87	1	NA	NA	NA	NA
NA	NA	NA	NA	0.19	1	0.23	1	NA	NA	NA	NA
NA	NA	NA	NA	0.20	1	0.22	1	NA	NA	NA	NA
NA	NA	NA	NA	0.20	1	0.23	1	NA	NA	NA	NA
NA	NA	NA	NA	5.65	1	0.29	1	NA	NA	NA	NA
NA	NA	NA	NA	0.25	1	0.18	1	NA	NA	NA	NA
NA	NA	NA	NA	0.25	1	0.21	1	NA	NA	NA	NA
NA	NA	NA	NA	0.22	1	0.23	1	NA	NA	NA	NA
NA	NA	NA	NA	5.56	1	0.25	1	NA	NA	NA	NA
NA	NA	NA	NA	0.29	1	0.21	1	NA	NA	NA	NA
NA	NA	NA	NA	0.24	1	0.35	1	NA	NA	NA	NA
NA	NA	NA	NA	0.23	1	0.23	1	NA	NA	NA	NA
NA	NA	NA	NA	0.22	1	0.06	1	NA	NA	NA	NA
NA	NA	NA	NA	3.92	1	0.12	1	NA	NA	NA	NA
NA	NA	NA	NA	0.24	1	0.09	1	NA	NA	NA	NA
NA	NA	NA	NA	0.24	1	0.10	1	NA	NA	NA	NA
NA	NA	NA	NA	0.22	1	0.08	1	NA	NA	NA	NA
NA	NA	NA	NA	0.22	1	1.34	1	NA	NA	NA	NA
NA	NA	NA	NA	0.18	1	0.21	1	NA	NA	NA	NA
NA	NA	NA	NA	3.43	0	0.08	1	NA	NA	NA	NA
NA	NA	NA	NA	2.37	1	0.10	1	NA	NA	NA	NA
NA	NA	NA	NA	0.31	1	0.25	1	NA	NA	NA	NA
NA	NA	NA	NA	0.22	1	0.08	1	NA	NA	NA	NA
NA	NA	NA	NA	0.20	1	0.25	1	NA	NA	NA	NA
NA	NA	NA	NA	4.47	1	0.09	1	NA	NA	NA	NA
NA	NA	NA	NA	0.25	1	0.12	1	NA	NA	NA	NA
NA	NA	NA	NA	0.22	1	0.09	1	NA	NA	NA	NA
NA	NA	NA	NA	0.21	1	0.97	1	NA	NA	NA	NA
NA	NA	NA	NA	3.23	1	0.09	1	NA	NA	NA	NA
NA	NA	NA	NA	0.26	1	0.12	1	NA	NA	NA	NA
NA	NA	NA	NA	0.23	1	0.22	1	NA	NA	NA	NA
NA	NA	NA	NA	0.22	1	0.30	1	NA	NA	NA	NA
NA	NA	NA	NA	0.20	1	0.23	1	NA	NA	NA	NA
NA	NA	NA	NA	0.19	1	0.09	1	NA	NA	NA	NA
NA	NA	NA	NA	0.22	1	0.27	1	NA	NA	NA	NA
NA	NA	NA	NA	0.20	1	0.21	1	NA	NA	NA	NA
NA	NA	NA	NA	4.41	1	1.18	1	NA	NA	NA	NA
NA	NA	NA	NA	0.24	1	0.22	1	NA	NA	NA	NA
NA	NA	NA	NA	0.21	1	0.08	1	NA	NA	NA	NA
NA	NA	NA	NA	0.22	1	0.10	1	NA	NA	NA	NA
NA	NA	NA	NA	0.20	1	0.23	1	NA	NA	NA	NA
NA	NA	NA	NA	0.19	1	0.21	1	NA	NA	NA	NA
NA	NA	NA	NA	5.74	1	0.21	1	NA	NA	NA	NA
NA	NA	NA	NA	0.28	1	0.21	1	NA	NA	NA	NA
NA	NA	NA	NA	0.23	1	0.30	1	NA	NA	NA	NA
NA	NA	NA	NA	0.21	1	0.22	1	NA	NA	NA	NA
NA	NA	NA	NA	8.07	1	0.95	1	NA	NA	NA	NA
NA	NA	NA	NA	0.22	1	0.23	1	NA	NA	NA	NA
NA	NA	NA	NA	0.19	1	0.23	1	NA	NA	NA	NA
NA	NA	NA	NA	0.23	1	0.37	1	NA	NA	NA	NA

time A	leuko A	time A	leuko A	time B	leuko B	time C	leuko C	time D	leuko D	time E	leuko E
NA	NA	NA	NA	0.18	1	0.45	1	NA	NA	NA	NA
NA	NA	NA	NA	0.20	1	0.43	1	NA	NA	NA	NA
NA	NA	NA	NA	4.10	1	0.22	1	NA	NA	NA	NA
NA	NA	NA	NA	0.25	1	2.04	1	NA	NA	NA	NA
NA	NA	NA	NA	0.21	1	0.20	1	NA	NA	NA	NA
NA	NA	NA	NA	0.31	1	0.29	1	NA	NA	NA	NA
NA	NA	NA	NA	0.20	1	0.31	1	NA	NA	NA	NA
NA	NA	NA	NA	4.71	1	0.25	1	NA	NA	NA	NA
NA	NA	NA	NA	0.24	1	0.33	1	NA	NA	NA	NA
NA	NA	NA	NA	0.19	1	0.62	1	NA	NA	NA	NA
NA	NA	NA	NA	0.18	1	0.26	1	NA	NA	NA	NA
NA	NA	NA	NA	8.80	1	0.22	1	NA	NA	NA	NA
NA	NA	NA	NA	0.24	1	0.24	1	NA	NA	NA	NA
NA	NA	NA	NA	0.20	1	3.74	1	NA	NA	NA	NA
NA	NA	NA	NA	0.20	1	0.20	1	NA	NA	NA	NA
NA	NA	NA	NA	0.24	1	0.07	1	NA	NA	NA	NA
NA	NA	NA	NA	0.19	1	0.08	1	NA	NA	NA	NA
NA	NA	NA	NA	4.36	1	0.06	1	NA	NA	NA	NA
NA	NA	NA	NA	0.25	1	0.14	1	NA	NA	NA	NA
NA	NA	NA	NA	0.22	1	0.22	1	NA	NA	NA	NA
NA	NA	NA	NA	0.32	1	0.07	1	NA	NA	NA	NA
NA	NA	NA	NA	0.21	1	0.11	1	NA	NA	NA	NA
NA	NA	NA	NA	0.47	1	0.08	1	NA	NA	NA	NA
NA	NA	NA	NA	0.24	1	0.11	1	NA	NA	NA	NA
NA	NA	NA	NA	0.20	1	0.21	1	NA	NA	NA	NA
NA	NA	NA	NA	0.22	1	0.43	1	NA	NA	NA	NA
NA	NA	NA	NA	3.82	1	0.22	1	NA	NA	NA	NA
NA	NA	NA	NA	0.24	1	0.20	1	NA	NA	NA	NA
NA	NA	NA	NA	0.22	1	0.25	1	NA	NA	NA	NA
NA	NA	NA	NA	0.28	1	0.21	1	NA	NA	NA	NA
NA	NA	NA	NA	0.20	1	1.12	1	NA	NA	NA	NA
NA	NA	NA	NA	6.36	0	0.06	1	NA	NA	NA	NA
NA	NA	NA	NA	1.27	1	0.10	1	NA	NA	NA	NA
NA	NA	NA	NA	0.25	1	0.21	1	NA	NA	NA	NA
NA	NA	NA	NA	0.23	1	0.42	1	NA	NA	NA	NA
NA	NA	NA	NA	3.70	0	0.08	1	NA	NA	NA	NA
NA	NA	NA	NA	0.69	1	0.11	1	NA	NA	NA	NA
NA	NA	NA	NA	5.31	1	0.07	1	NA	NA	NA	NA
NA	NA	NA	NA	0.25	1	0.07	1	NA	NA	NA	NA
NA	NA	NA	NA	0.21	1	0.07	1	NA	NA	NA	NA
NA	NA	NA	NA	5.45	1	0.15	1	NA	NA	NA	NA
NA	NA	NA	NA	0.22	1	0.08	1	NA	NA	NA	NA
NA	NA	NA	NA	3.55	1	0.10	1	NA	NA	NA	NA
NA	NA	NA	NA	4.90	1	0.09	1	NA	NA	NA	NA
NA	NA	NA	NA	0.23	1	2.59	1	NA	NA	NA	NA
NA	NA	NA	NA	0.23	1	0.20	1	NA	NA	NA	NA
NA	NA	NA	NA	0.18	1	0.24	1	NA	NA	NA	NA
NA	NA	NA	NA	3.53	1	0.06	1	NA	NA	NA	NA
NA	NA	NA	NA	0.22	1	0.11	1	NA	NA	NA	NA
NA	NA	NA	NA	2.06	1	0.23	1	NA	NA	NA	NA
NA	NA	NA	NA	0.24	1	0.08	1	NA	NA	NA	NA

time A	leuko A	time A	leuko A	time B	leuko B	time C	leuko C	time D	leuko D	time E	leuko E
NA	NA	NA	NA	3.49	1	0.26	1	NA	NA	NA	NA
NA	NA	NA	NA	0.23	1	0.09	1	NA	NA	NA	NA
NA	NA	NA	NA	0.23	1	0.20	1	NA	NA	NA	NA
NA	NA	NA	NA	0.33	1	1.19	1	NA	NA	NA	NA
NA	NA	NA	NA	0.22	1	0.24	1	NA	NA	NA	NA
NA	NA	NA	NA	0.19	1	0.44	1	NA	NA	NA	NA
NA	NA	NA	NA	0.19	1	0.94	1	NA	NA	NA	NA
NA	NA	NA	NA	0.24	1	0.02	1	NA	NA	NA	NA
NA	NA	NA	NA	0.19	1	0.01	1	NA	NA	NA	NA
NA	NA	NA	NA	3.11	1	0.16	1	NA	NA	NA	NA
NA	NA	NA	NA	0.25	1	0.07	1	NA	NA	NA	NA
NA	NA	NA	NA	0.22	1	0.13	1	NA	NA	NA	NA
NA	NA	NA	NA	0.19	1	0.09	1	NA	NA	NA	NA
NA	NA	NA	NA	0.18	1	0.12	1	NA	NA	NA	NA
NA	NA	NA	NA	3.74	0	0.07	1	NA	NA	NA	NA
NA	NA	NA	NA	0.57	0	0.11	1	NA	NA	NA	NA
NA	NA	NA	NA	2.59	0	0.25	1	NA	NA	NA	NA
NA	NA	NA	NA	0.93	1	0.11	1	NA	NA	NA	NA
NA	NA	NA	NA	0.29	1	1.02	1	NA	NA	NA	NA
NA	NA	NA	NA	0.34	1	0.22	1	NA	NA	NA	NA
NA	NA	NA	NA	7.06	1	0.05	1	NA	NA	NA	NA
NA	NA	NA	NA	0.88	1	0.16	1	NA	NA	NA	NA
NA	NA	NA	NA	0.28	1	0.44	1	NA	NA	NA	NA
NA	NA	NA	NA	0.21	1	0.18	1	NA	NA	NA	NA
NA	NA	NA	NA	0.22	1	1.45	1	NA	NA	NA	NA
NA	NA	NA	NA	0.25	1	0.26	1	NA	NA	NA	NA
NA	NA	NA	NA	4.52	1	0.22	1	NA	NA	NA	NA
NA	NA	NA	NA	1.23	1	0.20	1	NA	NA	NA	NA
NA	NA	NA	NA	0.26	1	0.20	1	NA	NA	NA	NA
NA	NA	NA	NA	0.22	1	0.30	1	NA	NA	NA	NA
NA	NA	NA	NA	0.20	1	2.77	1	NA	NA	NA	NA
NA	NA	NA	NA	9.69	1	0.22	1	NA	NA	NA	NA
NA	NA	NA	NA	0.24	1	0.27	1	NA	NA	NA	NA
NA	NA	NA	NA	0.22	1	0.20	1	NA	NA	NA	NA
NA	NA	NA	NA	0.20	1	0.60	1	NA	NA	NA	NA
NA	NA	NA	NA	0.40	1	0.22	1	NA	NA	NA	NA
NA	NA	NA	NA	0.24	1	0.23	1	NA	NA	NA	NA
NA	NA	NA	NA	0.21	1	0.05	1	NA	NA	NA	NA
NA	NA	NA	NA	0.19	1	0.10	1	NA	NA	NA	NA
NA	NA	NA	NA	0.23	1	1.05	1	NA	NA	NA	NA
NA	NA	NA	NA	0.19	1	0.26	1	NA	NA	NA	NA
NA	NA	NA	NA	0.20	1	0.30	1	NA	NA	NA	NA
NA	NA	NA	NA	5.58	1	0.26	1	NA	NA	NA	NA
NA	NA	NA	NA	0.25	1	0.07	1	NA	NA	NA	NA
NA	NA	NA	NA	0.21	1	0.13	1	NA	NA	NA	NA
NA	NA	NA	NA	0.19	1	0.22	1	NA	NA	NA	NA
NA	NA	NA	NA	0.18	1	0.28	1	NA	NA	NA	NA
NA	NA	NA	NA	8.37	1	0.26	1	NA	NA	NA	NA
NA	NA	NA	NA	0.45	1	0.02	1	NA	NA	NA	NA
NA	NA	NA	NA	0.24	1	0.01	1	NA	NA	NA	NA
NA	NA	NA	NA	0.22	1	0.15	1	NA	NA	NA	NA

time A	leuko A	time A	leuko A	time B	leuko B	time C	leuko C	time D	leuko D	time E	leuko E
NA	NA	NA	NA	0.22	1	0.08	1	NA	NA	NA	NA
NA	NA	NA	NA	0.20	1	0.11	1	NA	NA	NA	NA
NA	NA	NA	NA	0.19	1	0.09	1	NA	NA	NA	NA
NA	NA	NA	NA	0.28	1	0.12	1	NA	NA	NA	NA
NA	NA	NA	NA	0.21	1	0.07	1	NA	NA	NA	NA
NA	NA	NA	NA	0.20	1	0.28	1	NA	NA	NA	NA
NA	NA	NA	NA	0.19	1	0.23	1	NA	NA	NA	NA
NA	NA	NA	NA	5.99	0	0.17	1	NA	NA	NA	NA
NA	NA	NA	NA	1.53	0	0.28	1	NA	NA	NA	NA
NA	NA	NA	NA	1.00	1	0.11	1	NA	NA	NA	NA
NA	NA	NA	NA	0.26	1	1.15	1	NA	NA	NA	NA
NA	NA	NA	NA	0.23	1	0.21	1	NA	NA	NA	NA
NA	NA	NA	NA	0.35	1	0.22	1	NA	NA	NA	NA
NA	NA	NA	NA	0.20	1	0.25	1	NA	NA	NA	NA
NA	NA	NA	NA	0.21	1	0.09	1	NA	NA	NA	NA
NA	NA	NA	NA	0.20	1	0.30	1	NA	NA	NA	NA
NA	NA	NA	NA	0.26	1	0.03	1	NA	NA	NA	NA
NA	NA	NA	NA	0.18	1	0.14	1	NA	NA	NA	NA
NA	NA	NA	NA	0.32	1	0.08	1	NA	NA	NA	NA
NA	NA	NA	NA	0.19	1	0.12	1	NA	NA	NA	NA
NA	NA	NA	NA	3.73	1	0.25	1	NA	NA	NA	NA
NA	NA	NA	NA	0.26	1	0.01	1	NA	NA	NA	NA
NA	NA	NA	NA	0.23	1	0.02	1	NA	NA	NA	NA
NA	NA	NA	NA	0.21	1	0.18	1	NA	NA	NA	NA
NA	NA	NA	NA	0.23	1	0.31	1	NA	NA	NA	NA
NA	NA	NA	NA	0.29	1	0.35	1	NA	NA	NA	NA
NA	NA	NA	NA	0.22	1	0.25	1	NA	NA	NA	NA
NA	NA	NA	NA	0.21	1	3.17	1	NA	NA	NA	NA
NA	NA	NA	NA	1.10	1	0.21	1	NA	NA	NA	NA
NA	NA	NA	NA	4.67	1	0.21	1	NA	NA	NA	NA
NA	NA	NA	NA	0.25	1	0.27	1	NA	NA	NA	NA
NA	NA	NA	NA	0.20	1	0.06	1	NA	NA	NA	NA
NA	NA	NA	NA	0.27	1	0.12	1	NA	NA	NA	NA
NA	NA	NA	NA	0.21	1	0.24	1	NA	NA	NA	NA
NA	NA	NA	NA	0.21	1	0.07	1	NA	NA	NA	NA
NA	NA	NA	NA	0.20	1	0.12	1	NA	NA	NA	NA
NA	NA	NA	NA	0.26	1	0.07	1	NA	NA	NA	NA
NA	NA	NA	NA	0.20	1	0.13	1	NA	NA	NA	NA
NA	NA	NA	NA	0.18	1	0.23	1	NA	NA	NA	NA
NA	NA	NA	NA	0.20	1	0.39	1	NA	NA	NA	NA
NA	NA	NA	NA	0.27	1	0.24	1	NA	NA	NA	NA
NA	NA	NA	NA	0.19	1	0.69	1	NA	NA	NA	NA
NA	NA	NA	NA	0.26	1	0.07	1	NA	NA	NA	NA
NA	NA	NA	NA	0.18	1	1.30	1	NA	NA	NA	NA
NA	NA	NA	NA	13.27	1	0.21	1	NA	NA	NA	NA
NA	NA	NA	NA	0.28	1	0.11	1	NA	NA	NA	NA
NA	NA	NA	NA	0.23	1	0.10	1	NA	NA	NA	NA
NA	NA	NA	NA	0.31	1	0.21	1	NA	NA	NA	NA
NA	NA	NA	NA	0.21	1	0.21	1	NA	NA	NA	NA
NA	NA	NA	NA	0.34	1	0.18	1	NA	NA	NA	NA
NA	NA	NA	NA	0.21	1	1.16	1	NA	NA	NA	NA

time A	leuko A	time A	leuko A	time B	leuko B	time C	leuko C	time D	leuko D	time E	leuko E
NA	NA	NA	NA	0.30	1	0.20	1	NA	NA	NA	NA
NA	NA	NA	NA	0.21	1	0.24	1	NA	NA	NA	NA
NA	NA	NA	NA	1.17	1	0.21	1	NA	NA	NA	NA
NA	NA	NA	NA	0.25	1	0.42	1	NA	NA	NA	NA
NA	NA	NA	NA	0.21	1	1.01	1	NA	NA	NA	NA
NA	NA	NA	NA	0.19	1	0.18	1	NA	NA	NA	NA
NA	NA	NA	NA	0.09	1	0.22	1	NA	NA	NA	NA
NA	NA	NA	NA	0.10	1	0.08	1	NA	NA	NA	NA
NA	NA	NA	NA	7.70	0	0.11	1	NA	NA	NA	NA
NA	NA	NA	NA	5.22	1	0.18	1	NA	NA	NA	NA
NA	NA	NA	NA	0.24	1	0.25	1	NA	NA	NA	NA
NA	NA	NA	NA	0.22	1	0.99	1	NA	NA	NA	NA
NA	NA	NA	NA	0.21	1	0.21	1	NA	NA	NA	NA
NA	NA	NA	NA	0.20	1	0.23	1	NA	NA	NA	NA
NA	NA	NA	NA	0.31	1	0.09	1	NA	NA	NA	NA
NA	NA	NA	NA	0.22	1	0.16	1	NA	NA	NA	NA
NA	NA	NA	NA	0.19	1	0.25	1	NA	NA	NA	NA
NA	NA	NA	NA	0.19	1	0.19	1	NA	NA	NA	NA
NA	NA	NA	NA	1.76	1	1.20	1	NA	NA	NA	NA
NA	NA	NA	NA	0.28	1	0.19	1	NA	NA	NA	NA
NA	NA	NA	NA	0.22	1	0.09	1	NA	NA	NA	NA
NA	NA	NA	NA	0.24	1	0.12	1	NA	NA	NA	NA
NA	NA	NA	NA	0.80	1	0.21	1	NA	NA	NA	NA
NA	NA	NA	NA	0.27	1	0.21	1	NA	NA	NA	NA
NA	NA	NA	NA	0.24	1	0.25	1	NA	NA	NA	NA
NA	NA	NA	NA	0.24	1	0.07	1	NA	NA	NA	NA
NA	NA	NA	NA	1.99	0	0.16	1	NA	NA	NA	NA
NA	NA	NA	NA	NA	NA	0.26	1	NA	NA	NA	NA
NA	NA	NA	NA	NA	NA	1.31	1	NA	NA	NA	NA
NA	NA	NA	NA	NA	NA	0.22	1	NA	NA	NA	NA
NA	NA	NA	NA	NA	NA	0.50	1	NA	NA	NA	NA
NA	NA	NA	NA	NA	NA	0.10	1	NA	NA	NA	NA
NA	NA	NA	NA	NA	NA	0.14	1	NA	NA	NA	NA
NA	NA	NA	NA	NA	NA	1.15	1	NA	NA	NA	NA

1.3 A100

Table 3: Raw data of TBS A100.

time A	leuko A	time A	leuko A	time B	leuko B	time C	leuko C	time D	leuko D	time E	leuko E
0.00	1	0.00	1	0.00	1	0.00	1	0.00	1	0.00	1
0.31	1	0.21	1	0.39	1	0.22	1	0.19	1	0.22	1
0.22	1	0.18	1	0.38	1	0.29	1	0.21	1	0.23	1
0.21	1	0.35	1	0.82	1	0.35	1	0.41	1	0.24	1
0.18	1	0.41	1	0.32	1	0.41	1	0.21	1	0.30	1
0.21	1	0.21	1	0.29	1	0.75	1	0.32	1	4.74	1
0.50	1	0.20	1	0.27	1	0.23	1	0.20	1	0.26	1
0.24	1	0.16	1	0.61	1	0.26	1	0.25	1	0.22	1
0.21	1	0.17	1	0.30	1	0.49	1	0.21	1	0.28	1
0.21	1	0.16	1	0.29	1	0.47	1	0.26	1	0.28	1
0.55	1	0.19	1	0.25	1	0.42	1	0.20	1	0.23	1
0.21	1	1.59	1	0.77	1	0.22	1	0.29	1	6.59	1
0.19	1	0.20	1	0.30	1	0.28	1	0.29	1	0.25	1
0.21	1	0.15	1	0.33	1	0.29	1	0.42	1	0.31	1
0.19	1	0.19	1	0.85	1	14.62	1	0.52	1	0.30	1
0.17	1	0.19	1	0.30	1	0.24	1	0.22	1	0.23	1
3.32	1	0.17	1	0.26	1	0.23	1	0.18	1	14.51	1
0.25	1	0.14	1	0.26	1	0.23	1	6.30	1	0.29	1
0.19	1	0.18	1	0.36	1	0.31	1	0.27	1	0.25	1
0.17	1	0.14	1	0.28	1	0.27	1	0.27	1	0.27	1
0.18	1	1.28	1	0.25	1	0.19	1	0.39	1	0.24	1
0.06	1	0.23	1	0.32	1	0.24	1	0.38	1	4.12	1
0.08	1	0.12	1	0.28	1	0.19	1	2.31	1	0.29	1
0.18	1	0.18	1	0.24	1	0.25	1	0.28	1	0.27	1
0.18	1	0.15	1	0.24	1	0.19	1	0.22	1	0.29	1
0.17	1	0.16	1	0.48	1	0.28	1	0.35	1	0.25	1
0.21	1	0.14	1	0.32	1	0.20	1	0.19	1	0.24	1
2.65	1	0.13	1	0.28	1	2.83	1	0.21	1	0.31	1
0.25	1	0.14	1	0.31	1	0.22	1	0.51	1	5.16	1
0.20	1	1.84	1	0.29	1	0.19	1	0.30	1	0.29	1
0.17	1	0.21	1	8.40	1	0.23	1	0.23	1	0.27	1
0.18	1	0.17	1	0.30	1	0.28	1	9.17	1	0.27	1
0.17	1	0.18	1	0.26	1	0.25	1	0.21	1	0.24	1
0.19	1	0.16	1	0.27	1	0.36	1	0.22	1	0.29	1
0.17	1	0.18	1	2.37	1	0.18	1	0.48	1	0.23	1
0.19	1	0.18	1	0.27	1	0.24	1	0.19	1	0.29	1
0.19	1	0.19	1	0.26	1	0.35	1	0.18	1	0.26	1
0.18	1	0.18	1	0.27	1	0.20	1	0.34	1	0.26	1
0.18	1	0.19	1	0.87	1	0.20	1	0.19	1	0.30	1
0.18	1	0.20	1	0.30	1	0.23	1	0.44	1	0.26	1
0.17	1	3.01	1	0.29	1	0.19	1	0.40	1	0.30	1
0.20	1	0.21	1	1.20	1	0.21	1	0.19	1	0.35	1
0.20	1	0.16	1	0.28	1	0.24	1	0.21	1	3.18	1
0.19	1	0.18	1	0.27	1	0.19	1	0.29	1	0.25	1
3.40	1	0.16	1	0.27	1	0.26	1	0.52	1	0.25	1
0.30	1	0.17	1	0.58	1	0.22	1	0.21	1	0.28	1
0.18	1	0.17	1	0.28	1	0.23	1	0.22	1	0.27	1
0.23	1	0.17	1	0.27	1	5.16	1	0.37	1	2.82	1

time A	leuko A	time A	leuko A	time B	leuko B	time C	leuko C	time D	leuko D	time E	leuko E
0.29	1	0.17	1	0.27	1	0.21	1	0.43	1	0.24	1
0.23	1	0.16	1	0.51	1	0.28	1	0.17	1	0.28	1
0.19	1	0.16	1	0.27	1	0.21	1	0.31	1	0.25	1
0.26	1	0.18	1	0.38	1	0.20	1	0.20	1	0.27	1
0.17	1	0.17	1	0.26	1	0.21	1	10.26	1	0.25	1
0.22	1	0.15	1	3.22	1	0.21	1	0.20	1	0.26	1
0.19	1	1.95	1	0.28	1	0.22	1	0.37	1	0.07	1
0.22	1	0.18	1	0.26	1	0.19	1	0.21	1	0.19	1
2.97	1	0.19	1	0.53	1	0.22	1	0.36	1	0.05	1
0.28	1	0.18	1	0.24	1	0.24	1	0.44	1	0.21	1
0.18	1	0.18	1	0.26	1	0.19	1	0.23	1	0.25	1
0.21	1	0.18	1	0.21	1	0.19	1	0.24	1	2.37	1
0.18	1	0.18	1	0.38	1	0.19	1	0.26	1	0.29	1
0.17	1	0.18	1	0.23	1	0.20	1	0.20	1	0.23	1
0.19	1	0.17	1	0.34	1	0.28	1	0.21	1	0.31	1
0.20	1	0.17	1	0.27	1	0.19	1	0.21	1	0.27	1
0.18	1	0.18	1	0.25	1	0.20	1	0.20	1	0.22	1
0.20	1	0.18	1	0.23	1	0.21	1	0.22	1	0.28	1
0.18	1	0.16	1	0.35	1	0.27	1	0.19	1	0.25	1
0.20	1	0.18	1	0.24	1	4.63	1	0.23	1	0.27	1
0.18	1	0.17	1	0.24	1	0.23	1	0.18	1	3.04	1
0.21	1	0.18	1	0.33	1	0.31	1	0.41	1	0.29	1
0.19	1	0.16	1	0.26	1	0.29	1	0.18	1	0.25	1
0.19	1	2.07	1	0.23	1	0.18	1	2.26	1	0.26	1
0.19	1	0.22	1	0.21	1	0.18	1	0.20	1	0.27	1
4.10	1	0.19	1	8.18	1	0.21	1	0.21	1	0.29	1
0.26	1	0.18	1	0.28	1	0.26	1	0.20	1	0.28	1
0.18	1	0.18	1	0.27	1	0.30	1	0.21	1	0.25	1
0.19	1	0.17	1	0.25	1	0.21	1	0.19	1	0.31	1
0.19	1	0.17	1	0.45	1	0.27	1	0.52	1	0.26	1
0.17	1	0.17	1	0.26	1	0.20	1	0.21	1	0.32	1
0.19	1	0.20	1	0.25	1	0.20	1	0.20	1	3.28	1
0.17	1	0.18	1	0.38	1	0.21	1	0.17	1	0.27	1
0.17	1	0.18	1	0.27	1	0.19	1	8.22	1	0.24	1
0.28	1	0.16	1	0.24	1	0.21	1	0.20	1	0.25	1
0.18	1	0.18	1	0.40	1	0.20	1	0.83	1	0.24	1
0.21	1	0.18	1	0.28	1	0.22	1	0.20	1	0.26	1
0.15	1	0.17	1	0.26	1	0.22	1	0.17	1	0.24	1
0.19	1	0.17	1	0.43	1	2.92	1	0.46	1	0.28	1
2.02	1	2.16	1	0.27	1	0.21	1	0.62	1	0.24	1
0.24	1	0.24	1	0.25	1	0.21	1	0.20	1	0.26	1
0.20	1	0.21	1	0.24	1	0.24	1	2.19	1	0.31	1
0.18	1	0.18	1	0.23	1	0.22	1	0.22	1	2.71	1
0.19	1	0.19	1	2.97	1	0.20	1	0.40	1	0.28	1
0.19	1	0.19	1	0.28	1	0.20	1	0.29	1	0.25	1
0.19	1	0.20	1	0.24	1	0.20	1	0.19	1	0.26	1
0.20	1	0.18	1	0.23	1	0.23	1	0.31	1	0.32	1
0.19	1	0.18	1	0.35	1	0.26	1	9.60	1	0.20	1
0.14	1	0.18	1	0.25	1	0.21	1	0.20	1	0.27	1
0.26	1	0.18	1	0.24	1	0.20	1	0.31	1	0.23	1
0.18	1	0.18	1	0.22	1	0.19	1	0.41	1	0.25	1

time A	leuko A	time A	leuko A	time B	leuko B	time C	leuko C	time D	leuko D	time E	leuko E
0.19	1	0.20	1	2.18	1	0.19	1	0.19	1	0.24	1
0.15	1	4.36	1	0.26	1	0.20	1	0.24	1	0.25	1
0.20	1	0.21	1	0.21	1	0.21	1	0.17	1	2.57	1
0.20	1	0.17	1	0.24	1	2.23	1	0.23	1	0.26	1
0.19	1	0.18	1	0.40	1	0.20	1	0.19	1	0.45	1
0.19	1	0.18	1	0.26	1	0.21	1	0.20	1	0.30	1
0.21	1	0.17	1	0.24	1	0.23	1	0.17	1	0.26	1
0.20	1	0.20	1	0.22	1	0.20	1	0.26	1	0.25	1
0.26	1	0.18	1	0.32	1	0.24	1	0.25	1	0.25	1
0.45	1	0.17	1	0.23	1	0.23	1	0.17	1	0.28	1
0.35	1	0.35	1	0.22	1	0.19	1	0.19	1	6.77	1
0.23	1	0.12	1	0.21	1	0.08	1	0.21	1	0.31	1
0.19	1	0.17	1	4.86	1	0.12	1	0.18	1	0.26	1
0.22	1	0.23	1	0.27	1	0.23	1	0.19	1	0.28	1
0.20	1	0.19	1	0.26	1	0.26	1	0.21	1	0.08	1
4.90	1	0.17	1	0.24	1	0.26	1	0.21	1	0.16	1
0.27	1	0.19	1	0.38	1	0.24	1	0.24	1	0.26	1
0.18	1	0.18	1	0.23	1	0.22	1	0.29	1	0.26	1
0.17	1	0.20	1	0.24	1	0.21	1	0.22	1	0.28	1
0.17	1	0.20	1	0.28	1	0.21	1	0.26	1	0.27	1
0.19	1	3.89	1	0.24	1	2.40	1	0.19	1	5.95	1
0.18	1	0.21	1	0.17	1	0.21	1	0.18	1	0.26	1
0.19	1	0.19	1	0.22	1	0.21	1	9.46	1	0.34	1
0.19	1	0.17	1	0.30	1	0.20	1	0.20	1	0.24	1
0.19	1	0.20	1	0.23	1	0.21	1	0.20	1	0.21	1
0.17	1	0.19	1	0.22	1	0.20	1	0.24	1	0.26	1
0.20	1	0.16	1	0.23	1	0.28	1	0.49	1	0.25	1
0.19	1	0.18	1	0.23	1	0.22	1	0.20	1	0.24	1
0.20	1	0.18	1	4.62	1	0.22	1	0.22	1	0.25	1
0.19	1	0.22	1	0.27	1	0.21	1	0.45	1	0.25	1
0.18	1	0.45	1	0.26	1	0.22	1	0.21	1	0.24	1
0.20	1	0.25	1	0.42	1	0.24	1	0.21	1	2.40	1
0.19	1	0.18	1	0.22	1	0.22	1	0.56	1	0.25	1
0.19	1	0.20	1	0.25	1	0.21	1	0.32	1	0.26	1
0.19	1	0.20	1	0.25	1	0.32	1	0.63	1	0.23	1
0.21	1	0.20	1	0.52	1	0.23	1	0.57	1	0.24	1
0.15	1	0.19	1	0.37	1	0.24	1	0.62	1	3.66	1
3.17	1	0.19	1	0.27	1	0.22	1	0.23	1	0.30	1
0.24	1	0.21	1	0.26	1	3.70	1	0.34	1	0.25	1
0.19	1	0.21	1	0.25	1	0.20	1	0.33	1	0.27	1
0.19	1	0.20	1	0.32	1	0.20	1	0.20	1	0.26	1
0.19	1	13.82	1	0.56	1	0.29	1	0.33	1	0.27	1
0.19	1	0.24	1	0.29	1	0.22	1	0.57	1	0.26	1
0.16	1	0.18	1	0.23	1	0.21	1	0.36	1	0.26	1
0.20	1	0.19	1	0.25	1	0.18	1	0.24	1	0.07	1
0.18	1	0.16	1	0.31	1	0.21	1	5.66	1	0.13	1
0.20	1	0.19	1	0.24	1	0.21	1	0.24	1	4.57	1
0.20	1	0.17	1	0.22	1	0.08	1	0.23	1	0.26	1
0.19	1	0.16	1	0.28	1	0.39	1	0.45	1	0.28	1
0.19	1	0.18	1	0.24	1	0.23	1	0.22	1	0.27	1
0.20	1	0.17	1	0.23	1	0.22	1	0.55	1	0.23	1

time A	leuko A	time A	leuko A	time B	leuko B	time C	leuko C	time D	leuko D	time E	leuko E
0.20	1	0.19	1	4.26	1	0.21	1	0.20	1	0.28	1
0.19	1	0.18	1	0.26	1	0.23	1	0.29	1	0.24	1
0.19	1	0.34	1	0.23	1	0.24	1	0.20	1	0.25	1
0.19	1	0.20	1	0.35	1	0.22	1	0.20	1	0.23	1
0.19	1	2.97	1	0.23	1	0.21	1	0.22	1	4.89	1
1.82	1	0.39	1	0.23	1	0.23	1	0.18	1	0.27	1
0.23	1	0.16	1	0.23	1	0.24	1	0.26	1	0.24	1
0.20	1	0.12	1	0.31	1	0.22	1	0.22	1	0.26	1
0.18	1	0.25	1	0.23	1	0.20	1	0.20	1	0.25	1
0.18	1	0.22	1	0.23	1	4.29	1	0.26	1	0.26	1
0.20	1	0.16	1	0.20	1	0.21	1	0.19	1	0.24	1
0.17	1	0.17	1	1.27	1	0.23	1	0.36	1	5.89	1
0.18	1	0.17	1	0.27	1	0.19	1	0.48	1	0.27	1
0.18	1	0.19	1	0.24	1	0.20	1	0.23	1	0.32	1
0.18	1	0.20	1	0.21	1	0.19	1	0.29	1	0.25	1
0.19	1	0.18	1	0.24	1	0.25	1	0.30	1	0.27	1
0.19	1	0.17	1	0.27	1	0.21	1	13.90	1	0.26	1
0.19	1	1.79	1	0.22	1	0.26	1	0.20	1	0.24	1
0.19	1	2.70	1	0.23	1	0.20	1	0.18	1	0.25	1
0.20	1	0.24	1	0.24	1	0.19	1	0.29	1	0.24	1
0.16	1	0.17	1	0.22	1	0.20	1	0.20	1	0.23	1
0.20	1	0.18	1	0.32	1	0.22	1	0.18	1	0.24	1
0.20	1	0.19	1	0.27	1	0.32	1	0.19	1	3.20	1
0.18	1	0.18	1	0.25	1	0.21	1	0.21	1	0.23	1
2.78	1	0.19	1	0.26	1	0.21	1	0.21	1	0.27	1
0.25	1	0.17	1	3.10	1	0.23	1	0.17	1	0.21	1
0.17	1	0.21	1	0.26	1	5.89	1	0.16	1	0.25	1
0.20	1	0.20	1	0.63	1	0.19	1	0.34	1	0.27	1
0.17	1	0.18	1	0.23	1	0.23	1	0.23	1	0.24	1
0.17	1	0.17	1	0.45	1	0.19	1	0.18	1	0.26	1
0.17	1	0.18	1	0.25	1	0.25	1	0.20	1	0.23	1
0.18	1	0.19	1	0.35	1	0.22	1	0.19	1	0.22	1
0.18	1	3.86	1	0.25	1	0.21	1	0.18	1	0.22	1
0.18	1	0.21	1	0.25	1	0.20	1	0.18	1	0.30	1
0.19	1	0.18	1	1.00	1	0.26	1	0.19	1	5.23	1
0.18	1	0.19	1	0.26	1	0.20	1	0.17	1	0.32	1
0.19	1	0.18	1	0.22	1	0.25	1	0.15	1	0.26	1
0.18	1	0.18	1	0.32	1	0.21	1	0.19	1	0.28	1
0.20	1	0.18	1	0.22	1	0.08	1	0.18	1	0.23	1
0.18	1	0.19	1	0.31	1	3.54	1	0.20	1	0.26	1
0.20	1	0.20	1	0.23	1	0.19	1	5.19	1	0.27	1
0.20	1	0.15	1	0.23	1	0.20	1	0.19	1	0.25	1
3.20	1	0.19	1	0.24	1	0.21	1	0.21	1	0.28	1
0.18	1	0.19	1	2.26	1	0.20	1	2.02	1	0.25	1
0.20	1	0.19	1	0.29	1	0.22	1	0.20	1	0.26	1
0.19	1	0.16	1	0.24	1	0.22	1	0.27	1	8.33	1
0.35	1	0.20	1	0.23	1	0.21	1	0.22	1	0.25	1
0.22	1	0.17	1	0.29	1	0.26	1	0.25	1	0.29	1
0.21	1	3.74	1	0.24	1	0.09	1	0.20	1	0.28	1
2.70	1	0.26	1	0.21	1	0.57	1	0.24	1	0.30	1
0.24	1	0.21	1	0.22	1	0.22	1	0.14	1	0.24	1

time A	leuko A	time A	leuko A	time B	leuko B	time C	leuko C	time D	leuko D	time E	leuko E
0.19	1	0.18	1	0.26	1	0.21	1	0.20	1	0.29	1
0.19	1	0.20	1	0.23	1	0.22	1	0.38	1	0.26	1
0.17	1	0.16	1	0.22	1	0.21	1	0.17	1	0.26	1
0.18	1	0.16	1	0.28	1	0.34	1	0.29	1	0.24	1
0.18	1	0.18	1	0.24	1	3.12	1	0.26	1	0.28	1
0.18	1	0.18	1	0.20	1	0.21	1	0.26	1	0.26	1
0.18	1	0.16	1	0.28	1	0.21	1	9.50	1	0.25	1
0.16	1	0.18	1	0.21	1	0.20	1	0.19	1	0.25	1
0.17	1	0.17	1	5.13	1	0.21	1	0.28	1	0.25	1
0.22	1	0.18	1	0.29	1	0.39	1	0.23	1	0.27	1
0.19	1	0.20	1	0.24	1	0.35	1	0.22	1	4.61	1
0.18	1	2.96	1	0.31	1	2.72	1	0.24	1	0.24	1
0.22	1	0.21	1	0.25	1	0.22	1	0.23	1	0.30	1
0.22	1	0.16	1	0.22	1	0.24	1	0.23	1	0.29	1
0.18	1	0.20	1	0.23	1	0.46	1	0.23	1	0.28	1
2.09	1	0.17	1	0.29	1	0.23	1	0.21	1	0.24	1
0.27	1	0.19	1	0.24	1	0.20	1	0.23	1	0.22	1
0.17	1	0.18	1	0.24	1	0.22	1	0.22	1	0.28	1
0.19	1	0.19	1	0.22	1	0.25	1	0.22	1	0.24	1
0.19	1	0.19	1	0.34	1	0.24	1	0.22	1	0.27	1
0.19	1	0.19	1	0.23	1	0.25	1	0.20	1	0.23	1
0.20	1	0.19	1	0.22	1	0.19	1	0.23	1	2.98	1
0.17	1	0.17	1	0.22	1	0.18	1	0.20	1	0.24	1
0.18	1	0.19	1	2.97	1	0.22	1	0.24	1	0.50	1
0.18	1	0.45	1	0.28	1	0.23	1	0.24	1	0.30	1
0.21	1	2.96	1	0.25	1	0.19	1	0.24	1	0.25	1
0.17	1	0.20	1	0.26	1	0.19	1	0.26	1	0.26	1
2.30	1	0.21	1	0.42	1	0.21	1	0.25	1	0.26	1
0.28	1	0.23	1	0.26	1	0.22	1	0.24	1	0.25	1
0.24	1	0.19	1	0.24	1	0.07	1	0.24	1	0.25	1
0.20	1	0.17	1	0.26	1	1.92	1	0.21	1	0.30	1
0.21	1	0.19	1	0.24	1	0.22	1	0.24	1	0.28	1
0.18	1	0.20	1	0.30	1	0.20	1	0.25	1	6.14	1
0.20	1	0.18	1	0.22	1	0.20	1	0.23	1	0.23	1
0.19	1	0.16	1	0.23	1	0.20	1	0.23	1	0.28	1
0.20	1	0.16	1	0.30	1	0.36	1	0.23	1	0.26	1
0.18	1	0.18	1	0.20	1	0.21	1	0.23	1	0.25	1
0.18	1	0.19	1	3.24	1	0.28	1	0.24	1	0.26	1
0.20	1	0.19	1	0.31	1	0.25	1	0.23	1	0.25	1
0.20	1	0.17	1	0.27	1	0.35	1	0.23	1	0.27	1
0.20	1	0.18	1	0.32	1	0.20	1	0.20	1	0.28	1
0.19	1	0.24	1	0.28	1	0.20	1	0.24	1	0.28	1
0.18	1	0.24	1	0.24	1	0.21	1	0.23	1	2.54	1
2.52	1	1.99	1	0.34	1	0.21	1	0.21	1	0.30	1
0.22	1	0.23	1	0.26	1	0.41	1	0.23	1	0.27	1
0.18	1	0.16	1	0.22	1	0.28	1	0.19	1	0.26	1
0.19	1	0.18	1	0.39	1	0.30	1	0.19	1	0.26	1
0.18	1	0.17	1	0.28	1	0.65	1	0.21	1	0.26	1
0.18	1	0.19	1	0.31	1	5.50	1	7.92	1	0.27	1
0.17	1	0.18	1	0.24	1	0.21	1	0.18	1	0.26	1
0.17	1	0.17	1	0.21	1	0.22	1	0.18	1	0.30	1

time A	leuko A	time A	leuko A	time B	leuko B	time C	leuko C	time D	leuko D	time E	leuko E
4.08	1	0.14	1	3.59	1	0.21	1	0.19	1	3.39	1
0.24	1	0.17	1	0.22	1	0.25	1	0.30	1	0.23	1
0.19	1	0.17	1	0.22	1	0.19	1	0.20	1	0.30	1
0.20	1	0.16	1	0.27	1	0.21	1	0.18	1	0.25	1
0.18	1	1.58	1	0.22	1	0.20	1	0.21	1	0.24	1
0.18	1	0.20	1	3.86	1	0.48	1	0.18	1	0.32	1
0.18	1	0.17	1	0.28	1	0.20	1	0.25	1	0.28	1
0.19	1	0.17	1	0.25	1	0.23	1	0.21	1	0.26	1
0.19	1	0.34	1	0.24	1	0.30	1	0.18	1	0.27	1
0.19	1	0.19	1	0.24	1	0.21	1	0.23	1	0.28	1
0.18	1	0.17	1	0.45	1	0.21	1	0.89	1	0.29	1
0.17	1	1.83	1	0.25	1	0.24	1	0.20	1	0.29	1
0.17	1	0.19	1	0.22	1	0.20	1	0.16	1	0.30	1
0.19	1	0.18	1	0.33	1	0.19	1	0.16	1	0.23	1
0.18	1	0.18	1	0.23	1	0.28	1	2.11	1	0.21	1
1.97	1	0.13	1	0.24	1	0.80	1	0.22	1	0.24	1
0.23	1	0.23	1	0.23	1	0.21	1	0.19	1	3.01	1
0.18	1	0.21	1	0.21	1	4.11	1	0.22	1	0.28	1
0.17	1	0.17	1	0.38	1	0.21	1	0.23	1	0.28	1
0.21	1	0.19	1	0.52	1	0.22	1	0.19	1	0.25	1
0.15	1	0.18	1	0.29	1	0.08	1	0.20	1	0.29	1
0.19	1	0.19	1	0.25	1	0.10	1	0.19	1	0.26	1
0.18	1	0.19	1	0.28	1	0.22	1	10.56	1	0.26	1
0.18	1	0.17	1	0.54	1	0.18	1	0.27	1	0.27	1
0.19	1	3.53	1	0.28	1	0.21	1	0.23	1	0.29	1
0.17	1	0.20	1	0.25	1	0.27	1	0.17	1	0.29	1
0.18	1	0.18	1	0.46	1	0.20	1	0.30	1	0.35	1
0.17	1	0.16	1	0.26	1	0.21	1	0.18	1	0.35	1
0.18	1	0.17	1	5.52	1	0.24	1	0.26	1	6.52	1
0.18	1	0.17	1	0.26	1	0.39	1	0.18	1	0.22	1
1.83	1	0.18	1	0.23	1	0.08	1	0.28	1	0.29	1
0.23	1	0.18	1	0.19	1	0.44	1	0.15	1	0.34	1
0.20	1	0.14	1	0.70	1	2.44	1	0.19	1	0.27	1
0.19	1	0.19	1	0.29	1	0.20	1	0.17	1	0.27	1
0.19	1	0.17	1	0.27	1	0.28	1	0.25	1	0.28	1
0.17	1	0.18	1	0.64	1	0.21	1	0.19	1	0.26	1
0.20	1	0.19	1	0.68	1	0.48	1	0.18	1	0.25	1
0.17	1	0.34	1	0.25	1	0.71	1	0.19	1	0.27	1
0.18	1	1.84	1	0.37	1	0.24	1	0.29	1	4.24	1
0.20	1	0.20	1	0.25	1	2.23	1	0.40	1	0.31	1
0.23	1	0.16	1	0.21	1	0.22	1	0.18	1	0.09	1
0.20	1	0.19	1	0.21	1	0.24	1	0.22	1	0.19	1
0.21	1	0.18	1	5.46	1	0.24	1	0.19	1	0.06	1
2.20	1	0.10	1	0.32	1	0.06	1	0.21	1	0.19	1
0.21	1	0.20	1	0.28	1	0.47	1	0.35	1	0.29	1
0.19	1	0.28	1	0.34	1	0.65	1	0.29	1	0.26	1
0.18	1	0.19	1	0.27	1	4.02	1	0.23	1	0.26	1
0.18	1	0.19	1	0.25	1	0.20	1	0.39	1	0.25	1
0.18	1	0.19	1	0.25	1	2.33	1	0.19	1	0.25	1
0.22	1	0.18	1	0.35	1	0.22	1	0.19	1	0.11	1
0.13	1	0.19	1	0.24	1	0.23	1	12.12	1	0.16	1

time A	leuko A	time A	leuko A	time B	leuko B	time C	leuko C	time D	leuko D	time E	leuko E
0.15	1	0.18	1	0.23	1	3.36	1	0.23	1	0.27	1
3.58	1	0.20	1	0.24	1	0.20	1	0.20	1	0.27	1
0.21	1	0.19	1	0.29	1	0.22	1	0.17	1	0.26	1
0.21	1	0.20	1	0.23	1	0.21	1	0.23	1	0.26	1
0.20	1	2.00	1	0.21	1	0.19	1	0.19	1	0.10	1
0.32	1	0.20	1	4.59	1	0.20	1	0.21	1	0.18	1
0.21	1	0.20	1	0.26	1	2.60	1	0.25	1	4.24	1
0.17	1	0.19	1	0.23	1	0.20	1	0.23	1	0.30	1
0.18	1	0.20	1	5.14	1	0.19	1	0.20	1	0.10	1
0.18	1	0.19	1	0.27	1	0.19	1	0.19	1	0.15	1
0.19	1	0.20	1	0.92	1	0.21	1	0.18	1	0.24	1
0.19	1	0.18	1	0.27	1	0.23	1	0.18	1	0.25	1
0.20	1	0.21	1	0.60	1	0.19	1	0.18	1	0.24	1
0.28	1	0.21	1	0.26	1	0.21	1	0.27	1	0.26	1
10.98	1	0.19	1	0.23	1	0.19	1	0.21	1	0.30	1
0.23	1	0.19	1	0.28	1	0.25	1	0.22	1	0.27	1
0.18	1	0.23	1	0.24	1	2.25	1	0.19	1	0.29	1
0.13	1	0.19	1	0.22	1	0.20	1	0.17	1	0.25	1
0.17	1	0.16	1	0.23	1	0.30	1	4.90	1	0.27	1
0.16	1	2.12	1	0.21	1	0.24	1	0.24	1	0.25	1
0.18	1	0.33	1	0.28	1	0.20	1	0.32	1	0.32	1
0.18	1	0.52	1	0.23	1	0.19	1	0.20	1	5.35	1
0.33	1	0.21	1	0.22	1	0.19	1	0.17	1	0.26	1
0.20	1	0.18	1	0.22	1	0.26	1	0.18	1	0.07	1
0.18	1	0.19	1	0.22	1	0.25	1	0.19	1	0.12	1
0.19	1	0.22	1	4.30	1	0.19	1	0.18	1	0.23	1
0.18	1	0.20	1	0.27	1	2.56	1	0.16	1	0.25	1
0.19	1	0.19	1	0.23	1	0.21	1	0.19	1	0.25	1
0.20	1	0.16	1	0.25	1	0.19	1	0.17	1	0.41	1
1.82	1	4.00	1	1.62	1	0.19	1	0.16	1	0.27	1
0.19	1	0.20	1	0.24	1	0.37	1	0.21	1	0.24	1
0.41	1	0.18	1	0.33	1	0.31	1	0.18	1	0.26	1
0.20	1	0.18	1	0.23	1	0.22	1	0.17	1	0.25	1
0.12	1	0.17	1	0.34	1	0.23	1	0.19	1	0.25	1
0.17	1	0.19	1	0.23	1	0.20	1	0.33	1	0.27	1
0.17	1	0.20	1	0.41	1	0.22	1	0.19	1	0.27	1
0.16	1	0.17	1	0.24	1	0.26	1	0.17	1	0.25	1
0.17	1	4.67	1	0.24	1	0.22	1	2.04	1	0.25	1
0.18	1	0.21	1	0.22	1	0.23	1	0.21	1	3.73	1
0.15	1	0.13	1	0.38	1	0.30	1	0.18	1	0.25	1
0.19	1	0.28	1	0.24	1	0.23	1	0.18	1	0.28	1
0.19	1	0.19	1	0.23	1	0.07	1	0.18	1	0.29	1
5.01	1	0.18	1	0.22	1	2.34	1	0.05	1	0.27	1
0.24	1	0.18	1	0.22	1	0.20	1	0.11	1	0.28	1
0.19	1	0.20	1	0.34	1	0.20	1	0.18	1	0.27	1
0.21	1	0.16	1	0.24	1	0.25	1	0.06	1	0.26	1
0.17	1	0.18	1	0.23	1	0.22	1	0.11	1	0.28	1
0.18	1	1.91	1	0.29	1	0.21	1	0.18	1	0.28	1
0.18	1	0.22	1	0.23	1	0.21	1	0.34	1	0.25	1
0.17	1	0.19	1	0.21	1	0.25	1	0.22	1	0.24	1
0.19	1	0.19	1	4.16	1	0.35	1	0.22	1	0.26	1

time A	leuko A	time A	leuko A	time B	leuko B	time C	leuko C	time D	leuko D	time E	leuko E
0.18	1	0.18	1	0.25	1	0.21	1	0.18	1	11.45	1
0.20	1	1.62	1	0.24	1	0.06	1	0.15	1	0.24	1
2.51	1	0.23	1	0.42	1	0.15	1	4.99	1	0.25	1
0.23	1	0.23	1	0.25	1	0.41	1	0.21	1	0.25	1
0.17	1	0.20	1	0.30	1	1.80	1	0.16	1	0.24	1
0.18	1	0.19	1	0.22	1	0.21	1	0.23	1	0.09	1
0.17	1	1.83	1	0.23	1	0.20	1	0.19	1	0.13	1
0.19	1	0.20	1	0.22	1	0.19	1	0.19	1	0.22	1
0.18	1	4.30	1	0.20	1	0.19	1	0.19	1	0.26	1
0.18	1	0.18	1	0.29	1	0.22	1	0.18	1	0.25	1
0.20	1	0.18	1	0.24	1	0.21	1	0.20	1	0.26	1
0.17	1	0.16	1	0.24	1	0.19	1	0.20	1	0.23	1
0.19	1	0.17	1	0.22	1	0.23	1	0.18	1	0.24	1
0.19	1	0.16	1	5.96	1	0.38	1	0.20	1	0.29	1
0.20	1	0.17	1	0.26	1	0.22	1	0.18	1	0.25	1
0.21	1	0.17	1	0.20	1	0.21	1	0.17	1	0.25	1
0.20	1	0.17	1	0.23	1	0.24	1	0.22	1	0.27	1
0.20	1	0.17	1	0.30	1	0.20	1	0.20	1	0.25	1
2.12	1	0.18	1	0.22	1	0.23	1	0.17	1	0.26	1
0.49	1	0.16	1	0.20	1	0.20	1	0.15	1	0.27	1
0.25	1	0.07	1	0.28	1	0.22	1	0.14	1	0.23	1
0.13	1	0.09	1	0.22	1	0.27	1	0.19	1	3.21	1
0.22	1	2.90	1	0.21	1	2.11	1	0.19	1	0.28	1
0.18	1	0.19	1	0.22	1	0.21	1	0.19	1	0.25	1
0.17	1	0.16	1	0.75	1	0.20	1	0.19	1	0.28	1
0.18	1	0.17	1	3.20	1	0.19	1	0.18	1	0.26	1
0.16	1	0.17	1	0.25	1	0.25	1	0.19	1	0.28	1
0.18	1	0.18	1	0.22	1	0.20	1	0.19	1	0.28	1
0.16	1	0.16	1	0.43	1	0.19	1	0.16	1	0.27	1
0.18	1	0.19	1	0.23	1	0.22	1	0.19	1	0.27	1
0.17	1	0.19	1	0.21	1	0.51	1	0.21	1	0.28	1
0.17	1	0.17	1	0.30	1	2.02	1	0.19	1	0.27	1
0.19	1	0.18	1	0.20	1	0.20	1	0.18	1	0.27	1
0.18	1	2.31	1	0.70	1	0.28	1	0.17	1	0.29	1
0.12	1	0.19	1	0.26	1	0.21	1	0.15	1	2.54	1
0.19	1	0.17	1	0.22	1	0.22	1	6.09	1	0.26	1
0.23	1	0.18	1	0.20	1	0.28	1	0.21	1	0.27	1
0.23	1	0.17	1	0.22	1	0.21	1	0.27	1	0.39	1
2.57	1	0.36	1	0.27	1	0.21	1	0.19	1	0.46	1
0.20	1	0.19	1	2.92	1	0.20	1	0.19	1	0.20	1
0.18	1	0.19	1	0.33	1	0.24	1	0.19	1	0.31	1
0.18	1	0.17	1	0.27	1	0.21	1	0.19	1	0.30	1
0.18	1	0.22	1	0.38	1	0.22	1	0.20	1	0.27	1
0.17	1	0.19	1	0.23	1	0.19	1	0.21	1	0.29	1
0.15	1	3.80	1	0.17	1	0.20	1	0.24	1	0.26	1
0.18	1	0.19	1	0.22	1	0.24	1	0.24	1	0.24	1
0.15	1	0.19	1	0.29	1	0.21	1	0.20	1	0.24	1
0.15	1	0.18	1	0.21	1	2.38	1	0.16	1	0.27	1
0.17	1	0.19	1	3.90	1	0.20	1	0.18	1	0.26	1
0.19	1	0.16	1	4.99	1	0.25	1	0.18	1	0.25	1
0.19	1	0.17	1	0.26	1	0.21	1	0.19	1	2.85	1

time A	leuko A	time A	leuko A	time B	leuko B	time C	leuko C	time D	leuko D	time E	leuko E
0.20	1	0.17	1	0.50	1	0.21	1	0.17	1	0.36	1
0.17	1	0.17	1	0.26	1	0.22	1	0.17	1	0.79	1
0.20	1	0.05	1	0.20	1	0.23	1	9.44	1	0.25	1
0.35	1	3.41	1	0.22	1	0.06	1	0.20	1	0.26	1
0.22	1	0.20	1	0.24	1	0.14	1	0.16	1	0.28	1
0.19	1	0.19	1	0.29	1	0.25	1	0.28	1	0.27	1
2.52	1	0.18	1	0.21	1	0.22	1	0.18	1	0.24	1
0.23	1	0.18	1	0.29	1	0.19	1	0.20	1	1.87	1
0.26	1	0.18	1	0.21	1	0.21	1	0.17	1	0.27	1
0.23	1	0.18	1	0.22	1	0.25	1	0.18	1	0.26	1
0.18	1	0.34	1	0.20	1	0.38	1	0.17	1	0.24	1
0.20	1	0.20	1	0.29	1	0.32	1	0.17	1	0.25	1
0.18	1	0.17	1	0.20	1	0.23	1	0.33	1	0.27	1
0.17	1	0.18	1	2.31	1	0.25	1	0.20	1	0.25	1
0.19	1	4.55	1	0.28	1	0.24	1	0.19	1	0.24	1
0.18	1	0.22	1	0.24	1	0.38	1	0.20	1	2.07	1
0.18	1	0.20	1	0.23	1	0.53	1	0.17	1	0.30	1
0.21	1	0.13	1	0.30	1	0.29	1	0.23	1	0.24	1
0.27	1	0.22	1	0.22	1	0.07	1	0.18	1	0.25	1
2.29	1	0.18	1	0.23	1	0.14	1	0.19	1	0.28	1
0.21	1	0.18	1	0.74	1	0.22	1	0.16	1	0.24	1
0.18	1	0.21	1	0.26	1	0.23	1	0.25	1	0.28	1
0.17	1	0.28	1	0.20	1	0.23	1	0.31	1	0.29	1
0.19	1	0.22	1	0.21	1	0.24	1	0.21	1	0.23	1
0.18	1	0.17	1	0.26	1	0.22	1	0.19	1	0.24	1
0.19	1	0.22	1	0.22	1	2.62	1	0.20	1	0.28	1
0.20	1	0.21	1	0.24	1	0.22	1	0.18	1	0.24	1
0.18	1	0.21	1	0.21	1	0.21	1	0.20	1	0.26	1
0.20	1	2.59	1	0.30	1	0.08	1	0.20	1	4.59	1
0.19	1	0.22	1	0.21	1	0.14	1	0.18	1	1.22	1
0.20	1	0.16	1	2.80	1	0.21	1	0.18	1	0.31	1
3.42	1	0.21	1	0.27	1	0.21	1	0.16	1	0.27	1
0.22	1	0.17	1	0.47	1	0.20	1	0.17	1	1.02	1
0.19	1	0.19	1	0.24	1	0.19	1	5.51	1	0.22	1
0.17	1	0.18	1	0.22	1	0.22	1	0.21	1	0.27	1
0.17	1	0.18	1	0.21	1	0.20	1	0.19	1	0.25	1
0.18	1	0.19	1	0.18	1	0.23	1	0.19	1	0.25	1
0.17	1	0.17	1	0.36	1	0.23	1	0.19	1	3.86	1
0.15	1	0.18	1	0.20	1	0.21	1	0.18	1	0.31	1
0.19	1	0.20	1	0.21	1	5.97	1	0.19	1	0.43	1
0.18	1	2.41	1	0.22	1	0.24	1	0.20	1	0.30	1
0.18	1	0.24	1	0.21	1	0.20	1	0.20	1	0.27	1
0.16	1	0.19	1	2.77	1	0.20	1	0.17	1	0.25	1
0.21	1	0.17	1	0.29	1	0.20	1	0.20	1	0.28	1
0.20	1	0.19	1	0.28	1	0.21	1	0.19	1	0.25	1
0.29	1	0.19	1	0.26	1	0.19	1	0.17	1	0.25	1
0.19	1	0.20	1	0.37	1	0.20	1	0.19	1	0.27	1
0.18	1	0.18	1	0.27	1	0.20	1	0.19	1	3.61	1
0.20	1	0.21	1	0.24	1	0.28	1	0.16	1	0.33	1
0.20	1	0.16	1	0.31	1	0.21	1	0.19	1	0.26	1
0.16	1	1.85	1	0.24	1	0.25	1	0.18	1	0.29	1

time A	leuko A	time A	leuko A	time B	leuko B	time C	leuko C	time D	leuko D	time E	leuko E
1.84	1	0.22	1	0.31	1	0.08	1	0.18	1	0.27	1
0.20	1	0.17	1	0.24	1	0.15	1	0.18	1	0.28	1
0.17	1	0.19	1	0.24	1	0.22	1	0.19	1	0.22	1
0.18	1	0.18	1	0.22	1	0.21	1	0.17	1	3.70	1
0.17	1	0.21	1	0.50	1	0.20	1	0.18	1	0.27	1
0.17	1	0.19	1	0.27	1	0.23	1	0.22	1	0.29	1
0.18	1	0.19	1	0.28	1	0.20	1	6.75	1	0.25	1
0.18	1	0.19	1	0.25	1	2.39	1	0.18	1	0.25	1
0.22	1	0.18	1	0.21	1	0.20	1	0.20	1	0.27	1
0.18	1	0.19	1	0.22	1	0.20	1	0.19	1	0.29	1
0.18	1	0.27	1	3.65	1	0.21	1	0.20	1	0.27	1
0.18	1	0.12	1	0.28	1	0.20	1	0.16	1	0.25	1
0.18	1	0.17	1	0.24	1	0.56	1	0.19	1	0.25	1
2.05	1	0.18	1	0.23	1	0.21	1	0.18	1	0.27	1
0.24	1	0.22	1	0.31	1	4.90	1	0.20	1	0.30	1
0.24	1	3.66	1	0.23	1	0.23	1	0.20	1	2.99	1
0.17	1	0.23	1	0.22	1	0.21	1	0.21	1	0.24	1
0.21	1	0.20	1	0.35	1	0.22	1	0.19	1	0.28	1
0.19	1	0.17	1	0.23	1	0.19	1	0.17	1	0.25	1
0.17	1	0.19	1	0.21	1	0.20	1	0.22	1	0.28	1
0.18	1	0.20	1	3.61	1	0.21	1	0.17	1	0.29	1
0.14	1	0.17	1	0.27	1	0.05	1	0.20	1	0.27	1
0.18	1	0.19	1	0.25	1	0.08	1	0.18	1	2.90	1
0.17	1	0.19	1	0.25	1	0.21	1	0.20	1	0.35	1
0.32	1	0.23	1	0.23	1	0.20	1	0.20	1	0.30	1
0.19	1	0.15	1	0.23	1	0.21	1	0.19	1	0.24	1
0.18	1	0.18	1	0.22	1	0.28	1	0.22	1	0.28	1
0.14	1	0.20	1	0.32	1	0.25	1	0.20	1	0.25	1
3.14	1	0.18	1	0.31	1	0.23	1	0.20	1	0.27	1
0.21	1	2.79	1	0.28	1	0.23	1	0.19	1	0.29	1
0.18	1	0.20	1	0.33	1	0.22	1	4.14	1	0.28	1
0.18	1	0.17	1	0.26	1	2.66	1	0.17	1	1.97	1
0.20	1	0.18	1	0.25	1	0.20	1	0.18	1	0.26	1
0.16	1	0.17	1	0.25	1	0.20	1	0.20	1	0.31	1
0.34	1	0.19	1	0.33	1	0.08	1	0.21	1	2.20	1
0.28	1	0.18	1	0.49	1	0.12	1	0.23	1	0.33	1
0.23	1	0.18	1	0.27	1	0.31	1	0.21	1	0.28	1
0.19	1	0.19	1	0.25	1	0.26	1	0.19	1	0.28	1
0.17	1	0.19	1	0.29	1	0.34	1	0.19	1	0.29	1
0.18	1	0.20	1	7.15	1	3.07	1	0.16	1	0.25	1
0.20	1	3.74	1	0.24	1	0.19	1	0.18	1	0.26	1
0.17	1	0.23	1	0.24	1	0.20	1	0.19	1	0.30	1
0.16	1	0.19	1	0.24	1	0.14	1	0.18	1	2.79	1
0.20	1	0.18	1	0.22	1	0.23	1	4.06	1	0.24	1
0.19	1	0.18	1	0.20	1	0.20	1	0.20	1	0.28	1
1.93	1	0.20	1	0.26	1	0.19	1	0.22	1	0.25	1
0.25	1	0.18	1	0.22	1	0.42	1	0.39	1	0.24	1
0.19	1	0.17	1	0.22	1	0.22	1	0.19	1	0.24	1
0.16	1	0.18	1	0.20	1	0.23	1	0.32	1	0.25	1
0.20	1	0.17	1	0.32	1	0.25	1	0.21	1	0.10	1
0.26	1	0.17	1	0.26	1	0.28	1	0.19	1	0.18	1

time A	leuko A	time A	leuko A	time B	leuko B	time C	leuko C	time D	leuko D	time E	leuko E
0.22	1	0.18	1	0.22	1	0.20	1	0.27	1	NA	NA
0.18	1	0.18	1	0.23	1	0.28	1	0.19	1	NA	NA
0.17	1	0.20	1	0.24	1	0.23	1	0.21	1	NA	NA
0.19	1	0.17	1	0.25	1	2.43	1	0.19	1	NA	NA
0.18	1	0.21	1	NA	NA	0.21	1	0.21	1	NA	NA
0.18	1	0.17	1	NA	NA	0.23	1	0.17	1	NA	NA
0.41	1	0.16	1	NA	NA	0.20	1	0.22	1	NA	NA
NA	NA	0.18	1	NA	NA	0.19	1	0.18	1	NA	NA
NA	NA	NA	NA	NA	NA	0.25	1	0.19	1	NA	NA
NA	NA	NA	NA	NA	NA	0.21	1	0.19	1	NA	NA
NA	NA	NA	NA	NA	NA	0.21	1	0.18	1	NA	NA
NA	NA	NA	NA	NA	NA	0.23	1	0.18	1	NA	NA
NA	NA	NA	NA	NA	NA	0.22	1	0.18	1	NA	NA
NA	NA	NA	NA	NA	NA	0.20	1	0.23	1	NA	NA
NA	NA	NA	NA	NA	NA	0.20	1	0.28	1	NA	NA
NA	NA	NA	NA	NA	NA	0.21	1	0.20	1	NA	NA
NA	NA	NA	NA	NA	NA	0.21	1	0.19	1	NA	NA
NA	NA	NA	NA	NA	NA	0.18	1	0.17	1	NA	NA
NA	NA	NA	NA	NA	NA	2.19	1	0.17	1	NA	NA
NA	NA	NA	NA	NA	NA	0.19	1	0.17	1	NA	NA
NA	NA	NA	NA	NA	NA	0.06	1	NA	NA	NA	NA
NA	NA	NA	NA	NA	NA	0.13	1	NA	NA	NA	NA
NA	NA	NA	NA	NA	NA	0.23	1	NA	NA	NA	NA
NA	NA	NA	NA	NA	NA	0.19	1	NA	NA	NA	NA
NA	NA	NA	NA	NA	NA	0.21	1	NA	NA	NA	NA
NA	NA	NA	NA	NA	NA	0.51	1	NA	NA	NA	NA
NA	NA	NA	NA	NA	NA	0.20	1	NA	NA	NA	NA
NA	NA	NA	NA	NA	NA	0.22	1	NA	NA	NA	NA
NA	NA	NA	NA	NA	NA	0.07	1	NA	NA	NA	NA
NA	NA	NA	NA	NA	NA	0.12	1	NA	NA	NA	NA
NA	NA	NA	NA	NA	NA	0.06	1	NA	NA	NA	NA
NA	NA	NA	NA	NA	NA	0.11	1	NA	NA	NA	NA
NA	NA	NA	NA	NA	NA	0.26	1	NA	NA	NA	NA
NA	NA	NA	NA	NA	NA	0.21	1	NA	NA	NA	NA
NA	NA	NA	NA	NA	NA	0.43	1	NA	NA	NA	NA
NA	NA	NA	NA	NA	NA	2.13	1	NA	NA	NA	NA
NA	NA	NA	NA	NA	NA	0.20	1	NA	NA	NA	NA
NA	NA	NA	NA	NA	NA	0.20	1	NA	NA	NA	NA
NA	NA	NA	NA	NA	NA	0.32	1	NA	NA	NA	NA
NA	NA	NA	NA	NA	NA	0.21	1	NA	NA	NA	NA
NA	NA	NA	NA	NA	NA	0.28	1	NA	NA	NA	NA
NA	NA	NA	NA	NA	NA	0.21	1	NA	NA	NA	NA
NA	NA	NA	NA	NA	NA	0.23	1	NA	NA	NA	NA
NA	NA	NA	NA	NA	NA	0.22	1	NA	NA	NA	NA
NA	NA	NA	NA	NA	NA	0.21	1	NA	NA	NA	NA
NA	NA	NA	NA	NA	NA	0.22	1	NA	NA	NA	NA
NA	NA	NA	NA	NA	NA	0.28	1	NA	NA	NA	NA
NA	NA	NA	NA	NA	NA	0.07	1	NA	NA	NA	NA
NA	NA	NA	NA	NA	NA	0.12	1	NA	NA	NA	NA
NA	NA	NA	NA	NA	NA	0.23	1	NA	NA	NA	NA
NA	NA	NA	NA	NA	NA	0.18	1	NA	NA	NA	NA

time A	leuko A	time A	leuko A	time B	leuko B	time C	leuko C	time D	leuko D	time E	leuko E
NA	NA	NA	NA	NA	NA	0.24	1	NA	NA	NA	NA
NA	NA	NA	NA	NA	NA	2.77	1	NA	NA	NA	NA
NA	NA	NA	NA	NA	NA	0.20	1	NA	NA	NA	NA
NA	NA	NA	NA	NA	NA	0.20	1	NA	NA	NA	NA
NA	NA	NA	NA	NA	NA	0.22	1	NA	NA	NA	NA
NA	NA	NA	NA	NA	NA	0.27	1	NA	NA	NA	NA
NA	NA	NA	NA	NA	NA	0.45	1	NA	NA	NA	NA
NA	NA	NA	NA	NA	NA	0.33	1	NA	NA	NA	NA
NA	NA	NA	NA	NA	NA	0.04	1	NA	NA	NA	NA
NA	NA	NA	NA	NA	NA	0.39	1	NA	NA	NA	NA
NA	NA	NA	NA	NA	NA	0.21	1	NA	NA	NA	NA
NA	NA	NA	NA	NA	NA	2.22	1	NA	NA	NA	NA
NA	NA	NA	NA	NA	NA	0.23	1	NA	NA	NA	NA
NA	NA	NA	NA	NA	NA	0.27	1	NA	NA	NA	NA
NA	NA	NA	NA	NA	NA	0.01	1	NA	NA	NA	NA
NA	NA	NA	NA	NA	NA	0.18	1	NA	NA	NA	NA
NA	NA	NA	NA	NA	NA	0.36	1	NA	NA	NA	NA
NA	NA	NA	NA	NA	NA	0.20	1	NA	NA	NA	NA
NA	NA	NA	NA	NA	NA	0.21	1	NA	NA	NA	NA
NA	NA	NA	NA	NA	NA	0.23	1	NA	NA	NA	NA
NA	NA	NA	NA	NA	NA	0.20	1	NA	NA	NA	NA
NA	NA	NA	NA	NA	NA	0.28	1	NA	NA	NA	NA
NA	NA	NA	NA	NA	NA	0.23	1	NA	NA	NA	NA
NA	NA	NA	NA	NA	NA	0.06	1	NA	NA	NA	NA
NA	NA	NA	NA	NA	NA	0.12	1	NA	NA	NA	NA
NA	NA	NA	NA	NA	NA	0.23	1	NA	NA	NA	NA
NA	NA	NA	NA	NA	NA	0.22	1	NA	NA	NA	NA
NA	NA	NA	NA	NA	NA	0.27	1	NA	NA	NA	NA
NA	NA	NA	NA	NA	NA	0.23	1	NA	NA	NA	NA
NA	NA	NA	NA	NA	NA	0.21	1	NA	NA	NA	NA
NA	NA	NA	NA	NA	NA	0.25	1	NA	NA	NA	NA
NA	NA	NA	NA	NA	NA	0.06	1	NA	NA	NA	NA
NA	NA	NA	NA	NA	NA	0.13	1	NA	NA	NA	NA
NA	NA	NA	NA	NA	NA	0.23	1	NA	NA	NA	NA
NA	NA	NA	NA	NA	NA	2.12	1	NA	NA	NA	NA
NA	NA	NA	NA	NA	NA	0.19	1	NA	NA	NA	NA
NA	NA	NA	NA	NA	NA	0.06	1	NA	NA	NA	NA
NA	NA	NA	NA	NA	NA	0.12	1	NA	NA	NA	NA
NA	NA	NA	NA	NA	NA	0.19	1	NA	NA	NA	NA
NA	NA	NA	NA	NA	NA	0.19	1	NA	NA	NA	NA
NA	NA	NA	NA	NA	NA	0.22	1	NA	NA	NA	NA
NA	NA	NA	NA	NA	NA	0.20	1	NA	NA	NA	NA
NA	NA	NA	NA	NA	NA	0.20	1	NA	NA	NA	NA
NA	NA	NA	NA	NA	NA	0.19	1	NA	NA	NA	NA
NA	NA	NA	NA	NA	NA	0.07	1	NA	NA	NA	NA
NA	NA	NA	NA	NA	NA	0.13	1	NA	NA	NA	NA
NA	NA	NA	NA	NA	NA	0.21	1	NA	NA	NA	NA
NA	NA	NA	NA	NA	NA	0.23	1	NA	NA	NA	NA
NA	NA	NA	NA	NA	NA	0.23	1	NA	NA	NA	NA
NA	NA	NA	NA	NA	NA	0.26	1	NA	NA	NA	NA
NA	NA	NA	NA	NA	NA	0.23	1	NA	NA	NA	NA

time A	leuko A	time A	leuko A	time B	leuko B	time C	leuko C	time D	leuko D	time E	leuko E
NA	NA	NA	NA	NA	NA	0.30	1	NA	NA	NA	NA
NA	NA	NA	NA	NA	NA	0.23	1	NA	NA	NA	NA
NA	NA	NA	NA	NA	NA	0.26	1	NA	NA	NA	NA
NA	NA	NA	NA	NA	NA	0.23	1	NA	NA	NA	NA
NA	NA	NA	NA	NA	NA	0.22	1	NA	NA	NA	NA
NA	NA	NA	NA	NA	NA	0.22	1	NA	NA	NA	NA
NA	NA	NA	NA	NA	NA	0.09	1	NA	NA	NA	NA
NA	NA	NA	NA	NA	NA	0.30	1	NA	NA	NA	NA

1.4 OPT211

Table 4: Raw data of TBS OPT211.

time A	leuko A	time A	leuko A	time B	leuko B	time C	leuko C	time D	leuko D	time E	leuko E
0.00	0	0.00	1	0.00	1	0.00	0	0.00	0	0.00	1
0.28	0	0.29	1	0.69	1	0.59	0	0.20	0	0.27	1
0.21	0	0.25	1	0.52	1	3.37	0	0.20	0	0.29	1
3.42	0	3.35	0	1.11	1	0.27	0	0.21	0	0.46	1
0.26	0	0.27	0	0.69	1	0.25	0	0.64	0	2.93	0
0.20	0	0.24	0	1.00	1	0.65	0	0.21	0	0.26	0
0.20	0	0.28	0	0.77	1	0.31	0	0.19	0	0.26	0
0.19	0	0.24	0	0.99	1	0.31	0	0.59	0	0.59	0
0.21	0	0.21	0	0.88	1	3.21	0	0.20	0	0.26	0
0.17	0	0.23	0	0.64	1	0.29	0	0.24	0	0.21	0
0.20	0	0.25	0	1.37	1	0.24	0	0.19	0	0.48	0
0.21	0	0.25	0	0.67	1	0.46	0	0.28	0	0.27	0
5.49	0	0.25	0	6.15	0	0.35	0	0.22	0	0.23	0
0.24	0	0.21	0	0.27	0	0.45	0	0.22	0	0.23	0
0.19	0	0.21	0	0.24	0	0.24	0	0.19	0	0.43	0
0.17	0	0.24	0	0.26	0	0.38	0	0.20	0	0.29	0
0.19	0	0.23	0	0.20	0	0.31	0	0.19	0	0.23	0
0.20	0	0.23	0	0.33	0	0.25	0	0.23	0	0.40	0
0.26	0	0.21	0	0.21	0	1.95	0	0.19	0	0.29	0
0.19	0	0.22	0	0.22	0	0.28	0	0.22	0	0.39	0
0.17	0	0.24	0	0.22	0	0.22	0	0.22	0	0.29	0
0.18	0	0.22	0	0.21	0	0.28	0	0.21	0	0.23	0
0.16	0	0.21	0	0.29	0	0.25	0	0.18	0	0.24	0
0.16	0	0.20	0	0.24	0	0.24	0	0.21	0	0.19	0
0.16	0	0.21	0	0.25	0	0.22	0	0.19	0	0.20	0
0.17	0	0.32	0	0.23	0	0.31	0	0.20	0	0.22	0
0.18	0	0.21	0	0.22	0	0.24	0	0.22	0	0.24	0
0.16	0	0.21	0	0.27	0	0.22	0	0.32	0	0.25	0
0.18	0	0.19	0	0.24	0	0.25	0	0.23	0	0.18	0
0.16	0	0.21	0	0.24	0	0.30	0	0.24	0	0.23	0
0.21	0	0.22	0	0.22	0	0.23	0	0.22	0	0.22	0
0.29	0	0.18	0	0.23	0	0.23	0	0.23	0	0.19	0
0.18	0	0.19	0	0.26	0	0.44	0	0.22	0	0.21	0
0.20	0	0.19	0	0.24	0	0.35	0	0.21	0	0.45	0
0.21	0	0.19	0	0.23	0	0.35	0	0.21	0	0.26	0
0.20	0	0.17	0	0.23	0	0.23	0	0.22	0	0.19	0
0.17	0	0.15	0	0.24	0	0.21	0	0.22	0	0.19	0
0.18	0	0.19	0	0.25	0	0.38	0	0.19	0	1.79	0
0.17	0	0.19	0	0.24	0	0.23	0	0.20	0	0.26	0
0.17	0	0.19	0	0.23	0	0.43	0	0.20	0	0.64	0
0.18	0	0.22	0	0.24	0	0.23	0	0.21	0	0.23	0
0.17	0	0.18	0	0.24	0	0.27	0	0.20	0	0.50	0
0.17	0	0.19	0	0.24	0	0.23	0	0.26	0	0.24	0
0.18	0	0.20	0	0.23	0	0.29	0	3.88	0	0.43	0
0.18	0	0.18	0	0.22	0	0.41	0	0.27	0	0.30	0
0.16	0	0.21	0	0.24	0	0.24	0	0.20	0	2.54	0
0.14	0	0.19	0	0.24	0	0.37	0	0.20	0	0.56	0
0.11	0	0.19	0	0.26	0	0.29	0	0.20	0	0.25	0

time A	leuko A	time A	leuko A	time B	leuko B	time C	leuko C	time D	leuko D	time E	leuko E
0.19	0	0.21	0	0.24	0	0.30	0	0.20	0	0.32	0
0.19	0	0.19	0	0.23	0	0.43	0	0.16	0	0.30	0
0.06	0	0.20	0	0.23	0	0.37	0	0.20	0	0.41	0
0.25	0	0.20	0	0.23	0	0.30	0	0.18	0	0.23	0
0.19	0	0.22	0	0.23	0	0.43	0	0.18	0	0.60	0
0.16	0	0.19	0	0.22	0	3.58	0	0.19	0	2.15	0
0.19	0	0.21	0	0.24	0	1.09	0	0.21	0	0.28	0
0.27	0	0.20	0	0.23	0	0.21	0	0.18	0	0.21	0
0.21	0	0.21	0	0.21	0	0.64	0	0.20	0	0.44	0
0.18	0	0.18	0	0.28	0	0.47	0	0.20	0	0.25	0
0.18	0	0.19	0	0.25	0	0.31	0	0.19	0	0.21	0
0.25	0	0.18	0	0.23	0	0.28	0	0.19	0	0.22	0
0.19	0	0.18	0	0.23	0	0.50	0	0.19	0	0.50	0
0.19	0	0.20	0	0.25	0	0.27	0	0.19	0	2.15	0
0.18	0	0.19	0	0.25	0	0.28	0	0.19	0	0.48	0
0.20	0	0.19	0	0.24	0	0.33	0	0.18	0	0.28	0
0.17	0	0.20	0	0.21	0	0.33	0	0.20	0	0.26	0
0.18	0	0.20	0	0.24	0	0.26	0	0.20	0	0.23	0
0.19	0	0.20	0	0.24	0	0.34	0	0.19	0	0.54	0
0.18	0	0.18	0	0.26	0	0.25	0	0.20	0	0.24	0
0.19	0	0.20	0	0.24	0	0.47	0	0.20	0	0.23	0
0.17	0	0.18	0	0.23	0	0.29	0	0.19	0	0.22	0
0.18	0	0.20	0	0.23	0	0.43	0	0.19	0	0.62	0
0.19	0	0.20	0	0.23	0	0.61	0	0.19	0	0.26	0
0.20	0	0.20	0	0.22	0	0.39	0	0.19	0	0.24	0
0.20	0	0.19	0	0.23	0	0.27	0	0.17	0	0.25	0
0.20	0	0.20	0	0.25	0	0.24	0	0.20	0	0.27	0
0.19	0	0.20	0	0.23	0	0.23	0	0.19	0	0.52	0
0.19	0	0.18	0	0.23	0	0.23	0	0.19	0	0.22	0
0.19	0	0.22	0	0.23	0	0.26	0	0.19	0	0.27	0
0.18	0	0.21	0	0.23	0	0.27	0	0.19	0	1.61	0
0.17	0	0.22	0	0.23	0	0.21	0	0.19	0	0.27	0
0.18	0	0.21	0	0.22	0	0.22	0	0.20	0	0.54	0
0.20	0	0.20	0	0.23	0	0.36	0	0.20	0	1.20	0
0.18	0	0.19	0	0.24	0	0.26	0	0.19	0	0.29	0
0.20	0	0.18	0	0.24	0	0.22	0	0.18	0	0.25	0
0.16	0	0.19	0	0.25	0	0.89	0	0.18	0	0.25	0
0.21	0	0.19	0	0.22	0	0.96	0	0.19	0	0.20	0
0.18	0	0.21	0	0.25	0	0.22	0	0.19	0	0.25	0
0.21	0	0.19	0	0.23	0	0.25	0	0.18	0	0.22	0
0.22	0	0.18	0	0.22	0	0.28	0	0.20	0	0.24	0
0.19	0	0.20	0	0.23	0	0.30	0	0.19	0	0.26	0
0.19	0	0.22	0	0.24	0	0.22	0	0.19	0	0.27	0
0.20	0	0.21	0	0.23	0	0.23	0	0.20	0	0.24	0
0.20	0	0.21	0	0.23	0	0.43	0	0.20	0	0.66	0
0.16	0	0.21	0	0.26	0	0.25	0	0.17	0	0.23	0
0.18	0	0.24	0	0.21	0	0.24	0	0.18	0	0.29	0
0.20	0	0.20	0	0.24	0	0.23	0	0.18	0	0.32	0
0.21	0	0.22	0	0.22	0	0.22	0	0.16	0	0.67	0
0.22	0	0.20	0	0.23	0	0.40	0	0.15	0	0.27	0
0.19	0	0.19	0	0.24	0	0.25	0	0.18	0	1.29	0

time A	leuko A	time A	leuko A	time B	leuko B	time C	leuko C	time D	leuko D	time E	leuko E
0.19	0	0.22	0	0.23	0	0.30	0	0.16	0	0.51	0
0.19	0	0.21	0	0.24	0	0.30	0	0.18	0	0.27	0
1.66	0	0.21	0	0.24	0	0.29	0	0.06	0	0.27	0
0.26	0	0.20	0	0.23	0	0.24	0	0.11	0	0.27	0
0.18	0	2.34	0	0.25	0	0.25	0	0.19	0	0.28	0
0.19	0	0.23	0	0.24	0	0.23	0	0.05	0	0.27	0
0.16	0	0.20	0	0.23	0	0.28	0	0.10	0	0.63	0
0.21	0	0.21	0	0.25	0	0.36	0	0.06	0	0.25	0
0.20	0	0.21	0	0.23	0	0.22	0	0.11	0	0.51	0
0.21	0	0.21	0	0.24	0	0.24	0	0.18	0	0.25	0
0.16	0	0.20	0	0.23	0	0.36	0	0.20	0	2.53	0
0.18	0	0.23	0	0.24	0	0.36	0	0.19	0	0.24	0
0.21	0	0.31	0	0.24	0	0.23	0	0.20	0	0.44	0
0.19	0	0.30	0	0.23	0	0.31	0	0.20	0	0.28	0
0.19	0	0.24	0	0.23	0	0.21	0	0.18	0	0.23	0
0.20	0	0.42	0	0.23	0	0.26	0	0.20	0	0.24	0
0.20	0	0.20	0	0.22	0	0.21	0	0.22	0	0.24	0
0.22	0	0.18	0	0.23	0	0.21	0	0.20	0	0.76	0
0.17	0	0.20	0	0.23	0	0.22	0	0.18	0	0.29	0
0.20	0	0.19	0	0.21	0	0.31	0	0.20	0	0.45	0
0.19	0	0.21	0	0.22	0	0.24	0	0.19	0	0.25	0
0.19	0	0.19	0	0.23	0	0.25	0	0.19	0	0.26	0
0.20	0	0.24	0	0.24	0	0.24	0	0.19	0	0.25	0
0.20	0	0.19	0	0.23	0	0.31	0	0.19	0	0.22	0
0.27	0	0.13	0	0.23	0	0.25	0	0.19	0	0.36	0
0.22	0	0.30	0	0.23	0	0.27	0	0.17	0	0.30	0
0.22	0	0.24	0	0.23	0	0.33	0	0.18	0	0.26	0
0.19	0	0.20	0	0.24	0	0.23	0	0.19	0	0.24	0
0.21	0	0.31	0	0.23	0	0.28	0	0.18	0	0.87	0
0.25	0	0.28	0	0.23	0	0.29	0	0.21	0	0.26	0
0.21	0	0.19	0	0.25	0	0.36	0	0.18	0	0.24	0
0.19	0	0.24	0	0.23	0	0.29	0	0.20	0	0.26	0
0.22	0	0.22	0	0.24	0	0.43	0	0.19	0	1.19	0
0.20	0	0.21	0	0.23	0	0.58	0	0.20	0	0.28	0
0.19	0	0.20	0	0.24	0	0.30	0	0.20	0	0.22	0
0.21	0	0.21	0	0.25	0	0.32	0	0.21	0	0.24	0
0.20	0	0.21	0	0.23	0	0.35	0	0.22	0	0.81	0
0.22	0	0.17	0	0.22	0	0.30	0	0.17	0	0.26	0
0.19	0	0.20	0	0.24	0	0.30	0	0.21	0	0.25	0
0.19	0	0.21	0	0.23	0	0.28	0	0.19	0	0.26	0
0.20	0	0.23	0	0.22	0	0.30	0	0.19	0	0.24	0
0.22	0	0.20	0	0.25	0	0.27	0	0.19	0	0.24	0
0.19	0	0.37	0	0.23	0	0.31	0	0.22	0	5.02	0
0.19	0	0.24	0	0.23	0	0.30	0	0.19	0	0.24	0
0.22	0	0.20	0	0.23	0	0.26	0	0.19	0	0.23	0
0.21	0	0.19	0	0.24	0	0.31	0	0.22	0	0.58	0
0.22	0	0.22	0	0.24	0	0.28	0	0.21	0	0.25	0
0.19	0	0.21	0	0.23	0	0.25	0	0.21	0	0.68	0
0.21	0	0.20	0	0.23	0	0.37	0	0.21	0	0.40	0
0.20	0	0.21	0	0.24	0	0.23	0	0.18	0	0.87	0
0.20	0	0.20	0	0.23	0	0.21	0	0.20	0	0.24	0

time A	leuko A	time A	leuko A	time B	leuko B	time C	leuko C	time D	leuko D	time E	leuko E
0.20	0	0.22	0	0.24	0	0.23	0	0.20	0	0.23	0
0.19	0	0.20	0	0.25	0	0.49	0	0.21	0	0.27	0
0.20	0	0.21	0	0.23	0	0.24	0	0.19	0	0.56	0
0.15	0	0.22	0	0.24	0	0.23	0	0.21	0	0.22	0
0.19	0	0.22	0	0.24	0	0.26	0	0.19	0	0.26	0
0.22	0	0.21	0	0.24	0	0.32	0	0.19	0	1.20	0
0.21	0	0.21	0	0.23	0	0.32	0	0.19	0	0.22	0
0.21	0	0.22	0	0.24	0	0.35	0	0.16	0	0.20	0
0.22	0	0.23	0	0.23	0	0.33	0	0.21	0	0.25	0
0.22	0	0.21	0	0.24	0	0.42	0	0.19	0	0.25	0
0.20	0	0.21	0	0.24	0	6.96	0	0.16	0	0.21	0
0.21	0	0.21	0	0.24	0	0.30	0	0.19	0	0.27	0
0.19	0	0.25	0	0.23	0	0.24	0	0.20	0	1.06	0
0.23	0	0.24	0	0.23	0	0.22	0	0.21	0	0.22	0
0.40	0	0.25	0	0.21	0	0.44	0	0.17	0	0.25	0
0.22	0	0.30	0	0.23	0	0.25	0	0.22	0	0.26	0
0.24	0	0.23	0	0.22	0	1.21	0	0.21	0	1.20	0
0.35	0	0.22	0	0.24	0	0.25	0	0.19	0	0.19	0
0.23	0	0.23	0	0.23	0	0.28	0	0.21	0	0.23	0
0.23	0	0.21	0	0.24	0	0.31	0	0.21	0	0.23	0
0.22	0	0.22	0	0.25	0	0.27	0	0.25	0	0.46	0
0.21	0	0.21	0	0.24	0	0.25	0	0.20	0	0.25	0
0.21	0	0.23	0	0.24	0	0.63	0	0.22	0	1.57	0
0.20	0	0.25	0	0.24	0	0.24	0	0.23	0	0.69	0
0.19	0	0.20	0	0.25	0	0.22	0	0.23	0	0.26	0
0.87	0	0.27	0	0.23	0	0.35	0	0.21	0	0.25	0
0.29	0	0.24	0	0.23	0	0.26	0	0.20	0	0.25	0
0.36	0	0.26	0	0.22	0	0.23	0	0.20	0	0.45	0
0.25	0	0.36	0	0.23	0	0.29	0	0.21	0	0.26	0
0.21	0	0.24	0	0.23	0	0.25	0	0.21	0	0.25	0
0.22	0	0.20	0	0.24	0	0.91	0	0.22	0	0.23	0
0.19	0	0.21	0	0.23	0	0.24	0	0.22	0	0.22	0
1.33	0	0.22	0	0.21	0	0.21	0	0.22	0	0.53	0
0.18	0	15.48	1	0.24	0	0.22	0	0.19	0	0.23	0
0.30	0	0.25	1	0.24	0	0.22	0	0.20	0	0.31	0
0.21	0	0.24	1	0.23	0	0.23	0	0.18	0	0.33	0
0.23	0	0.20	1	0.23	0	0.93	0	0.22	0	0.43	0
0.18	0	0.24	1	0.24	0	0.26	0	0.20	0	0.28	0
0.19	0	2.83	0	0.23	0	0.53	0	0.20	0	0.30	0
0.21	0	0.25	0	0.23	0	0.22	0	0.21	0	0.29	0
0.22	0	0.20	0	0.25	0	0.23	0	0.19	0	0.23	0
0.35	0	0.17	0	0.25	0	0.57	0	0.20	0	0.31	0
0.23	0	0.16	0	0.22	0	0.25	0	0.19	0	0.23	0
0.20	0	0.19	0	0.24	0	0.24	0	0.20	0	0.23	0
0.21	0	0.18	0	0.24	0	0.95	0	0.18	0	0.23	0
0.20	0	0.17	0	0.22	0	0.21	0	0.17	0	1.15	0
0.21	0	0.18	0	0.22	0	1.47	0	0.37	0	0.20	0
0.21	0	0.25	0	0.22	0	0.26	0	0.02	0	0.25	0
0.21	0	0.22	0	0.24	0	0.89	0	0.18	0	0.24	0
0.22	0	0.15	0	0.23	0	0.25	0	0.20	0	0.27	0
0.21	0	0.15	0	0.24	0	0.22	0	0.20	0	1.30	0

time A	leuko A	time A	leuko A	time B	leuko B	time C	leuko C	time D	leuko D	time E	leuko E
0.23	0	0.18	0	0.23	0	0.28	0	0.20	0	0.19	0
0.20	0	0.17	0	0.24	0	0.28	0	0.20	0	0.28	0
0.22	0	0.18	0	0.22	0	0.26	0	0.20	0	0.24	0
0.21	0	0.15	0	0.24	0	0.30	0	0.18	0	0.24	0
0.20	0	0.18	0	0.25	0	0.31	0	0.20	0	0.52	0
0.20	0	0.16	0	0.22	0	0.93	0	0.33	0	0.24	0
0.22	0	0.16	0	0.24	0	0.75	0	0.23	0	0.36	0
0.16	0	0.12	0	0.25	0	0.24	0	0.18	0	1.58	0
0.20	0	0.22	0	0.23	0	0.26	0	0.19	0	0.24	0
0.21	0	0.29	0	0.26	0	0.74	1	0.16	0	0.28	0
0.20	0	0.19	0	0.21	0	0.24	1	0.19	0	0.23	0
0.22	0	0.16	0	0.24	0	0.38	1	0.19	0	0.27	0
0.21	0	0.15	0	0.24	0	0.26	1	0.19	0	0.28	0
0.22	0	0.20	0	0.23	0	0.91	1	0.19	0	0.26	0
0.21	0	0.18	0	0.25	0	0.20	1	0.19	0	0.22	0
0.62	0	0.20	0	0.61	0	0.45	1	0.18	0	0.33	0
0.28	0	0.18	0	0.25	0	15.82	1	0.17	0	0.32	0
0.25	0	0.20	0	0.24	0	0.38	1	0.21	0	0.21	0
0.25	0	0.19	0	0.24	0	0.36	1	0.35	0	0.37	0
0.23	0	0.20	0	0.22	0	3.54	0	0.23	0	0.24	0
0.22	0	0.22	0	0.23	0	0.28	0	0.23	0	0.84	0
0.20	0	0.19	0	0.23	0	0.25	0	0.23	0	0.48	0
0.24	0	0.21	0	0.23	0	0.24	0	0.18	0	0.24	0
0.24	0	0.19	0	0.24	0	0.20	0	0.19	0	0.25	0
0.34	0	0.20	0	0.24	0	0.22	0	0.18	0	1.04	0
0.21	0	0.22	0	0.21	0	0.26	0	0.19	0	0.25	0
0.20	0	0.19	0	0.24	0	0.23	0	0.20	0	0.22	0
0.21	0	0.18	0	0.22	0	0.33	0	0.17	0	0.27	0
0.20	0	0.22	0	0.25	0	0.25	0	0.20	0	0.27	0
0.22	0	0.32	0	0.23	0	0.75	0	0.20	0	0.36	0
0.20	0	0.23	0	0.23	0	0.26	0	0.19	0	0.25	0
0.20	0	0.15	0	0.23	0	0.26	0	0.21	0	0.29	0
0.15	0	0.18	0	0.23	0	0.64	0	0.20	0	0.64	0
0.28	0	0.22	0	0.26	0	0.20	0	0.18	0	0.26	0
0.22	0	0.19	0	0.23	0	0.24	0	0.18	0	0.23	0
0.20	0	0.20	0	0.25	0	0.25	0	0.22	0	0.24	0
0.15	0	0.18	0	0.51	0	1.20	0	0.22	0	0.23	0
0.28	0	0.20	0	0.35	0	0.27	0	0.15	0	0.25	0
0.23	0	0.20	0	0.30	0	0.31	0	0.19	0	0.28	0
0.19	0	0.18	0	0.25	0	0.19	0	0.20	0	0.25	0
0.24	0	0.19	0	0.26	0	0.42	0	0.20	0	0.23	0
0.24	0	0.21	0	0.26	0	0.40	0	0.18	0	0.23	0
0.25	0	0.22	0	0.24	0	0.23	0	0.20	0	0.23	0
0.24	0	0.22	0	0.24	0	0.36	0	0.20	0	0.26	0
0.23	0	0.20	0	0.23	0	0.21	0	0.21	0	0.23	0
0.22	0	0.22	0	0.24	0	0.30	0	0.19	0	0.22	0
0.19	0	0.21	0	0.25	0	0.35	0	0.21	0	0.23	0
0.26	0	0.21	0	0.25	0	2.39	0	0.20	0	0.26	0
0.18	0	0.20	0	0.24	0	0.28	0	0.20	0	0.25	0
0.22	0	0.22	0	0.25	0	0.29	0	0.20	0	0.22	0
0.22	0	0.23	0	0.24	0	0.44	0	0.19	0	0.21	0

time A	leuko A	time A	leuko A	time B	leuko B	time C	leuko C	time D	leuko D	time E	leuko E
0.25	0	0.21	0	0.24	0	0.40	0	0.21	0	0.22	0
0.23	0	0.25	0	0.23	0	0.21	0	0.18	0	0.22	0
0.52	0	0.25	0	0.24	0	0.34	0	0.21	0	0.23	0
0.22	0	0.20	0	0.25	0	0.29	0	0.28	0	0.23	0
0.22	0	0.26	0	0.25	0	0.22	0	0.20	0	0.23	0
0.21	0	0.27	0	0.20	0	0.27	0	0.29	0	1.04	0
0.21	0	0.21	0	0.24	0	0.50	0	0.24	0	0.17	0
0.21	0	0.19	0	0.25	0	0.31	0	0.25	0	0.36	0
0.19	0	0.21	0	0.24	0	0.22	0	0.21	0	0.25	0
0.20	0	0.24	0	0.26	0	0.52	0	0.20	0	0.24	0
0.10	0	0.27	0	0.23	0	0.22	0	0.19	0	0.25	0
0.27	0	0.25	0	0.24	0	0.34	0	0.21	0	0.25	0
0.21	0	0.29	0	0.24	0	0.20	0	0.21	0	0.32	0
0.21	0	0.54	0	0.24	0	0.27	0	0.20	0	0.28	0
0.21	0	0.29	0	0.23	0	0.40	0	0.20	0	0.24	0
0.21	0	0.22	0	0.25	0	0.28	0	0.22	0	0.26	0
0.23	0	0.24	0	0.24	0	0.23	0	0.20	0	0.30	0
0.16	0	0.22	0	0.23	0	0.21	0	0.20	0	0.54	0
0.27	0	0.23	0	0.24	0	0.26	0	0.21	0	0.24	0
0.23	0	0.25	0	0.24	0	0.22	0	0.19	0	2.59	0
0.22	0	0.18	0	0.22	0	0.22	0	0.21	0	0.21	0
0.27	0	0.22	0	0.23	0	0.25	0	0.19	0	0.21	0
0.25	0	0.22	0	0.01	0	0.27	0	0.18	0	0.23	0
0.29	0	0.21	0	0.20	0	0.23	0	0.21	0	0.22	0
0.24	0	0.22	0	0.22	0	0.34	0	0.18	0	0.22	0
0.22	0	0.23	0	0.23	0	0.28	0	0.21	0	0.22	0
0.21	0	0.23	0	0.23	0	0.20	0	0.19	0	0.23	0
0.22	0	0.23	0	0.23	0	0.24	0	0.20	0	0.25	0
0.21	0	0.22	0	0.24	0	0.49	0	0.23	0	0.23	0
0.21	0	0.21	0	0.24	0	0.24	0	0.29	0	1.13	0
0.22	0	0.20	0	0.24	0	0.20	0	0.19	0	0.25	0
0.20	0	0.21	0	0.24	0	0.24	0	0.21	0	0.24	0
0.24	0	0.21	0	0.25	0	0.22	0	0.18	0	0.41	0
0.21	0	0.21	0	0.24	0	0.45	0	0.20	0	0.25	0
0.18	0	0.34	0	0.24	0	0.26	0	0.20	0	0.27	0
0.22	0	0.28	0	0.23	0	0.22	0	0.21	0	0.20	0
0.19	0	0.24	0	0.24	0	0.20	0	0.20	0	0.23	0
0.23	0	0.22	0	0.24	0	0.29	0	0.20	0	0.30	0
0.19	0	0.20	0	0.24	0	0.24	0	0.20	0	0.27	0
0.20	0	0.23	0	0.27	0	0.27	0	0.20	0	0.25	0
0.61	1	0.15	0	0.22	0	0.26	0	0.19	0	0.22	0
0.55	1	0.24	0	0.24	0	0.41	0	0.21	0	0.23	0
0.49	1	0.19	0	0.23	0	0.19	0	0.18	0	0.25	0
0.40	1	0.22	0	0.26	0	0.23	0	0.21	0	0.24	0
0.36	1	0.21	0	0.24	0	0.18	0	0.18	0	0.23	0
NA	NA	0.20	0	0.23	0	0.30	0	0.20	0	0.22	0
NA	NA	0.23	0	0.23	0	0.17	0	0.19	0	0.22	0
NA	NA	0.19	0	0.25	0	0.36	0	0.20	0	0.20	0
NA	NA	0.24	0	0.25	0	0.36	0	0.18	0	3.18	0
NA	NA	0.20	0	0.26	0	0.28	0	0.21	0	0.23	0
NA	NA	0.19	0	0.23	0	0.23	0	0.20	0	0.22	0

time A	leuko A	time A	leuko A	time B	leuko B	time C	leuko C	time D	leuko D	time E	leuko E
NA	NA	0.21	0	0.23	0	0.26	0	0.21	0	0.21	0
NA	NA	0.22	0	0.23	0	0.22	0	0.16	0	0.85	0
NA	NA	0.20	0	0.24	0	0.26	0	0.17	0	0.12	0
NA	NA	0.19	0	0.24	0	0.22	0	0.19	0	0.23	0
NA	NA	0.23	0	0.23	0	0.23	0	0.18	0	0.24	0
NA	NA	0.22	0	0.24	0	0.19	0	0.20	0	0.31	0
NA	NA	0.22	0	0.24	0	0.28	0	0.18	0	0.30	0
NA	NA	0.20	0	0.24	0	0.35	0	0.21	0	0.23	0
NA	NA	0.24	0	0.24	0	0.30	0	0.21	0	0.22	0
NA	NA	0.24	0	0.27	0	0.29	0	0.17	0	0.24	0
NA	NA	0.24	0	0.25	0	0.20	0	0.21	0	0.22	0
NA	NA	0.21	0	0.25	0	0.30	0	0.19	0	0.23	0
NA	NA	0.25	0	0.24	0	0.22	0	0.19	0	0.24	0
NA	NA	0.25	0	0.25	0	0.21	0	0.18	0	0.25	0
NA	NA	0.26	0	0.25	0	0.24	0	0.21	0	0.23	0
NA	NA	0.20	0	0.26	0	0.31	0	0.20	0	0.24	0
NA	NA	0.23	0	0.25	0	0.22	0	0.20	0	0.51	0
NA	NA	0.21	0	0.24	0	0.21	0	0.19	0	0.25	0
NA	NA	0.21	0	0.27	0	0.24	0	0.18	0	0.24	0
NA	NA	0.21	0	0.23	0	0.43	0	0.19	0	NA	NA
NA	NA	0.21	0	0.25	0	0.28	0	0.19	0	NA	NA
NA	NA	0.24	0	0.25	0	0.42	0	0.18	0	NA	NA
NA	NA	0.22	0	0.25	0	0.26	0	0.21	0	NA	NA
NA	NA	0.22	0	0.24	0	0.22	0	0.20	0	NA	NA
NA	NA	0.20	0	0.24	0	0.26	0	0.23	0	NA	NA
NA	NA	0.22	0	0.25	0	0.22	0	0.17	0	NA	NA
NA	NA	0.20	0	0.25	0	0.23	0	0.19	0	NA	NA
NA	NA	0.23	0	0.24	0	0.19	0	0.21	0	NA	NA
NA	NA	0.28	0	0.25	0	0.23	0	0.19	0	NA	NA
NA	NA	0.27	0	0.25	0	0.21	0	0.20	0	NA	NA
NA	NA	0.24	0	0.24	0	0.22	0	0.22	0	NA	NA
NA	NA	0.21	0	0.23	0	0.36	0	0.22	0	NA	NA
NA	NA	0.20	0	0.23	0	0.30	0	0.52	0	NA	NA
NA	NA	0.23	0	NA	NA	0.18	0	0.21	0	NA	NA
NA	NA	0.24	0	NA	NA	0.25	0	0.18	0	NA	NA
NA	NA	0.29	0	NA	NA	0.19	0	0.22	0	NA	NA
NA	NA	0.31	0	NA	NA	0.23	0	0.21	0	NA	NA
NA	NA	0.24	0	NA	NA	0.21	0	0.19	0	NA	NA
NA	NA	0.25	0	NA	NA	0.35	0	0.21	0	NA	NA
NA	NA	0.20	0	NA	NA	0.17	0	0.22	0	NA	NA
NA	NA	0.26	0	NA	NA	0.39	0	0.19	0	NA	NA
NA	NA	0.38	0	NA	NA	0.29	0	0.20	0	NA	NA
NA	NA	0.23	0	NA	NA	0.25	0	0.22	0	NA	NA
NA	NA	0.21	0	NA	NA	0.34	0	0.19	0	NA	NA
NA	NA	0.25	0	NA	NA	0.21	0	0.20	0	NA	NA
NA	NA	0.38	0	NA	NA	0.37	0	0.21	0	NA	NA
NA	NA	0.26	0	NA	NA	0.21	0	0.19	0	NA	NA
NA	NA	0.30	0	NA	NA	0.54	0	0.19	0	NA	NA
NA	NA	0.26	0	NA	NA	0.57	0	0.20	0	NA	NA
NA	NA	0.23	0	NA	NA	0.26	0	0.18	0	NA	NA
NA	NA	0.36	0	NA	NA	0.37	0	0.23	0	NA	NA

time A	leuko A	time A	leuko A	time B	leuko B	time C	leuko C	time D	leuko D	time E	leuko E
NA	NA	0.22	0	NA	NA	0.50	0	0.19	0	NA	NA
NA	NA	0.23	0	NA	NA	0.23	0	0.18	0	NA	NA
NA	NA	0.22	0	NA	NA	0.34	0	0.21	0	NA	NA
NA	NA	0.33	0	NA	NA	0.24	0	0.21	0	NA	NA
NA	NA	0.21	0	NA	NA	0.23	0	0.19	0	NA	NA
NA	NA	0.39	0	NA	NA	0.29	0	0.20	0	NA	NA
NA	NA	0.29	0	NA	NA	0.33	0	0.19	0	NA	NA
NA	NA	0.26	0	NA	NA	0.22	0	0.22	0	NA	NA
NA	NA	0.22	0	NA	NA	0.29	0	0.18	0	NA	NA
NA	NA	0.24	0	NA	NA	0.27	0	0.21	0	NA	NA
NA	NA	0.21	0	NA	NA	0.35	0	0.20	0	NA	NA
NA	NA	0.22	0	NA	NA	0.26	0	0.18	0	NA	NA
NA	NA	0.20	0	NA	NA	0.30	0	0.20	0	NA	NA
NA	NA	0.23	0	NA	NA	0.38	0	0.20	0	NA	NA
NA	NA	0.19	0	NA	NA	0.27	0	0.20	0	NA	NA
NA	NA	0.22	0	NA	NA	0.24	0	0.21	0	NA	NA
NA	NA	0.19	0	NA	NA	0.21	0	0.20	0	NA	NA
NA	NA	0.19	0	NA	NA	0.37	0	0.20	0	NA	NA
NA	NA	0.30	0	NA	NA	0.21	0	0.20	0	NA	NA
NA	NA	0.16	0	NA	NA	0.34	0	0.23	0	NA	NA
NA	NA	0.25	0	NA	NA	0.40	0	0.20	0	NA	NA
NA	NA	0.28	0	NA	NA	0.29	0	0.21	0	NA	NA
NA	NA	0.28	0	NA	NA	0.26	0	0.22	0	NA	NA
NA	NA	0.12	0	NA	NA	0.29	0	0.22	0	NA	NA
NA	NA	0.28	0	NA	NA	0.28	0	0.19	0	NA	NA
NA	NA	0.23	0	NA	NA	0.43	0	0.19	0	NA	NA
NA	NA	0.21	0	NA	NA	0.25	0	0.21	0	NA	NA
NA	NA	0.22	0	NA	NA	0.38	0	0.19	0	NA	NA
NA	NA	0.31	0	NA	NA	0.21	0	0.21	0	NA	NA
NA	NA	0.24	0	NA	NA	0.31	0	0.45	0	NA	NA
NA	NA	0.25	0	NA	NA	0.21	0	0.22	0	NA	NA
NA	NA	0.14	0	NA	NA	0.22	0	0.28	0	NA	NA
NA	NA	0.18	0	NA	NA	0.23	0	2.58	1	NA	NA
NA	NA	0.19	0	NA	NA	0.25	0	0.29	1	NA	NA
NA	NA	0.25	0	NA	NA	0.23	0	0.29	1	NA	NA
NA	NA	0.23	0	NA	NA	0.23	0	1.80	1	NA	NA
NA	NA	0.24	0	NA	NA	0.25	0	0.53	1	NA	NA
NA	NA	0.17	0	NA	NA	0.30	0	1.69	1	NA	NA
NA	NA	0.26	0	NA	NA	0.22	0	0.62	1	NA	NA
NA	NA	0.21	0	NA	NA	0.25	0	2.09	1	NA	NA
NA	NA	0.20	0	NA	NA	0.23	0	0.96	1	NA	NA
NA	NA	0.27	0	NA	NA	0.27	0	0.40	1	NA	NA
NA	NA	0.24	0	NA	NA	0.22	0	0.36	1	NA	NA
NA	NA	0.15	0	NA	NA	0.26	0	0.54	1	NA	NA
NA	NA	0.26	0	NA	NA	0.25	0	0.97	0	NA	NA
NA	NA	0.26	0	NA	NA	0.23	0	0.18	0	NA	NA
NA	NA	0.20	0	NA	NA	0.21	0	0.20	0	NA	NA
NA	NA	0.23	0	NA	NA	0.27	0	0.18	0	NA	NA
NA	NA	0.22	0	NA	NA	0.28	0	0.18	0	NA	NA
NA	NA	0.23	0	NA	NA	0.19	0	0.20	0	NA	NA
NA	NA	0.25	0	NA	NA	0.21	0	0.19	0	NA	NA

time A	leuko A	time A	leuko A	time B	leuko B	time C	leuko C	time D	leuko D	time E	leuko E
NA	NA	0.28	0	NA	NA	0.26	0	0.17	0	NA	NA
NA	NA	0.23	0	NA	NA	0.77	0	0.18	0	NA	NA
NA	NA	0.22	0	NA	NA	0.22	0	0.19	0	NA	NA
NA	NA	0.24	0	NA	NA	0.23	0	0.18	0	NA	NA
NA	NA	0.27	0	NA	NA	0.22	0	0.19	0	NA	NA
NA	NA	0.22	0	NA	NA	0.27	0	0.16	0	NA	NA
NA	NA	0.24	0	NA	NA	0.28	0	0.19	0	NA	NA
NA	NA	0.26	0	NA	NA	0.38	0	0.17	0	NA	NA
NA	NA	0.22	0	NA	NA	0.27	0	0.18	0	NA	NA
NA	NA	0.28	0	NA	NA	0.21	0	0.18	0	NA	NA
NA	NA	0.33	0	NA	NA	0.30	0	0.15	0	NA	NA
NA	NA	0.33	0	NA	NA	0.21	0	0.17	0	NA	NA
NA	NA	0.34	0	NA	NA	0.22	0	0.18	0	NA	NA
NA	NA	NA	NA	NA	NA	0.29	0	0.17	0	NA	NA
NA	NA	NA	NA	NA	NA	0.25	0	0.17	0	NA	NA
NA	NA	NA	NA	NA	NA	0.30	0	0.17	0	NA	NA
NA	NA	NA	NA	NA	NA	0.27	0	0.20	0	NA	NA
NA	NA	NA	NA	NA	NA	0.24	0	0.18	0	NA	NA
NA	NA	NA	NA	NA	NA	0.21	0	0.17	0	NA	NA
NA	NA	NA	NA	NA	NA	0.24	0	0.18	0	NA	NA
NA	NA	NA	NA	NA	NA	0.25	0	0.19	0	NA	NA
NA	NA	NA	NA	NA	NA	0.23	0	0.17	0	NA	NA
NA	NA	NA	NA	NA	NA	0.27	0	0.17	0	NA	NA
NA	NA	NA	NA	NA	NA	0.23	0	0.15	0	NA	NA
NA	NA	NA	NA	NA	NA	0.38	0	0.15	0	NA	NA
NA	NA	NA	NA	NA	NA	0.25	0	0.16	0	NA	NA
NA	NA	NA	NA	NA	NA	0.22	0	0.34	0	NA	NA
NA	NA	NA	NA	NA	NA	0.23	0	0.20	0	NA	NA
NA	NA	NA	NA	NA	NA	0.24	0	0.21	0	NA	NA
NA	NA	NA	NA	NA	NA	0.31	0	0.20	0	NA	NA
NA	NA	NA	NA	NA	NA	0.35	0	0.20	0	NA	NA
NA	NA	NA	NA	NA	NA	0.29	0	0.19	0	NA	NA
NA	NA	NA	NA	NA	NA	0.24	0	0.19	0	NA	NA
NA	NA	NA	NA	NA	NA	0.23	0	0.18	0	NA	NA
NA	NA	NA	NA	NA	NA	0.24	0	0.16	0	NA	NA
NA	NA	NA	NA	NA	NA	0.30	0	0.17	0	NA	NA
NA	NA	NA	NA	NA	NA	0.23	0	0.20	0	NA	NA
NA	NA	NA	NA	NA	NA	0.23	0	0.19	0	NA	NA
NA	NA	NA	NA	NA	NA	0.25	0	0.20	0	NA	NA
NA	NA	NA	NA	NA	NA	0.24	0	0.17	0	NA	NA
NA	NA	NA	NA	NA	NA	0.26	0	0.18	0	NA	NA
NA	NA	NA	NA	NA	NA	0.28	0	0.19	0	NA	NA
NA	NA	NA	NA	NA	NA	0.26	0	0.18	0	NA	NA
NA	NA	NA	NA	NA	NA	0.33	0	0.20	0	NA	NA
NA	NA	NA	NA	NA	NA	0.31	0	0.21	1	NA	NA
NA	NA	NA	NA	NA	NA	0.31	0	0.21	1	NA	NA
NA	NA	NA	NA	NA	NA	0.26	0	0.01	0	NA	NA
NA	NA	NA	NA	NA	NA	0.51	0	0.14	0	NA	NA
NA	NA	NA	NA	NA	NA	0.25	0	0.18	0	NA	NA
NA	NA	NA	NA	NA	NA	0.32	0	0.15	0	NA	NA
NA	NA	NA	NA	NA	NA	0.25	0	0.19	0	NA	NA

time A	leuko A	time A	leuko A	time B	leuko B	time C	leuko C	time D	leuko D	time E	leuko E
NA	NA	NA	NA	NA	NA	0.32	0	0.19	0	NA	NA
NA	NA	NA	NA	NA	NA	0.23	0	0.19	0	NA	NA
NA	NA	NA	NA	NA	NA	0.34	0	0.19	0	NA	NA
NA	NA	NA	NA	NA	NA	0.24	0	0.19	0	NA	NA
NA	NA	NA	NA	NA	NA	0.26	0	0.17	0	NA	NA
NA	NA	NA	NA	NA	NA	0.36	0	0.19	0	NA	NA
NA	NA	NA	NA	NA	NA	0.23	0	0.20	0	NA	NA
NA	NA	NA	NA	NA	NA	0.41	0	0.18	0	NA	NA
NA	NA	NA	NA	NA	NA	0.25	0	0.21	0	NA	NA
NA	NA	NA	NA	NA	NA	0.23	0	0.22	0	NA	NA
NA	NA	NA	NA	NA	NA	0.24	0	0.19	0	NA	NA
NA	NA	NA	NA	NA	NA	0.29	0	0.20	0	NA	NA
NA	NA	NA	NA	NA	NA	0.26	0	0.21	0	NA	NA
NA	NA	NA	NA	NA	NA	0.27	0	0.20	0	NA	NA
NA	NA	NA	NA	NA	NA	0.27	0	0.20	0	NA	NA
NA	NA	NA	NA	NA	NA	0.24	0	0.21	0	NA	NA
NA	NA	NA	NA	NA	NA	0.27	0	0.20	0	NA	NA
NA	NA	NA	NA	NA	NA	0.24	0	0.20	0	NA	NA
NA	NA	NA	NA	NA	NA	0.31	0	0.19	0	NA	NA
NA	NA	NA	NA	NA	NA	0.25	0	0.18	0	NA	NA
NA	NA	NA	NA	NA	NA	0.26	0	0.13	1	NA	NA
NA	NA	NA	NA	NA	NA	0.25	0	0.20	0	NA	NA
NA	NA	NA	NA	NA	NA	0.28	0	0.20	0	NA	NA
NA	NA	NA	NA	NA	NA	0.23	0	0.18	0	NA	NA
NA	NA	NA	NA	NA	NA	0.24	0	0.17	0	NA	NA
NA	NA	NA	NA	NA	NA	0.32	0	0.15	0	NA	NA
NA	NA	NA	NA	NA	NA	0.24	0	0.20	0	NA	NA
NA	NA	NA	NA	NA	NA	0.22	0	0.20	0	NA	NA
NA	NA	NA	NA	NA	NA	0.25	0	0.20	0	NA	NA
NA	NA	NA	NA	NA	NA	0.26	0	0.19	0	NA	NA
NA	NA	NA	NA	NA	NA	0.23	0	0.07	0	NA	NA
NA	NA	NA	NA	NA	NA	0.22	0	0.10	0	NA	NA
NA	NA	NA	NA	NA	NA	0.26	0	0.19	0	NA	NA
NA	NA	NA	NA	NA	NA	0.24	0	0.16	0	NA	NA
NA	NA	NA	NA	NA	NA	0.27	0	0.19	0	NA	NA
NA	NA	NA	NA	NA	NA	0.27	0	0.18	0	NA	NA
NA	NA	NA	NA	NA	NA	0.26	0	0.34	1	NA	NA
NA	NA	NA	NA	NA	NA	0.25	0	0.15	0	NA	NA
NA	NA	NA	NA	NA	NA	0.24	0	0.11	0	NA	NA
NA	NA	NA	NA	NA	NA	0.23	0	0.09	0	NA	NA
NA	NA	NA	NA	NA	NA	0.30	0	0.15	0	NA	NA
NA	NA	NA	NA	NA	NA	0.24	0	0.21	0	NA	NA
NA	NA	NA	NA	NA	NA	0.23	0	0.16	0	NA	NA
NA	NA	NA	NA	NA	NA	0.26	0	0.20	0	NA	NA
NA	NA	NA	NA	NA	NA	0.24	0	0.20	0	NA	NA
NA	NA	NA	NA	NA	NA	0.35	0	0.18	0	NA	NA
NA	NA	NA	NA	NA	NA	0.27	0	0.19	0	NA	NA
NA	NA	NA	NA	NA	NA	0.24	0	0.19	0	NA	NA
NA	NA	NA	NA	NA	NA	0.22	0	0.20	0	NA	NA
NA	NA	NA	NA	NA	NA	0.27	0	0.20	0	NA	NA
NA	NA	NA	NA	NA	NA	0.22	0	0.21	0	NA	NA

time A	leuko A	time A	leuko A	time B	leuko B	time C	leuko C	time D	leuko D	time E	leuko E
NA	NA	NA	NA	NA	NA	0.25	0	0.20	0	NA	NA
NA	NA	NA	NA	NA	NA	0.46	0	0.20	0	NA	NA
NA	NA	NA	NA	NA	NA	0.24	0	0.22	0	NA	NA
NA	NA	NA	NA	NA	NA	0.26	0	0.21	0	NA	NA
NA	NA	NA	NA	NA	NA	0.30	0	0.20	0	NA	NA
NA	NA	NA	NA	NA	NA	0.24	0	0.19	0	NA	NA
NA	NA	NA	NA	NA	NA	0.33	0	0.16	0	NA	NA
NA	NA	NA	NA	NA	NA	0.20	0	0.12	1	NA	NA
NA	NA	NA	NA	NA	NA	0.49	0	0.21	0	NA	NA
NA	NA	NA	NA	NA	NA	0.22	0	0.26	0	NA	NA
NA	NA	NA	NA	NA	NA	0.37	0	0.25	0	NA	NA
NA	NA	NA	NA	NA	NA	0.22	0	0.23	0	NA	NA
NA	NA	NA	NA	NA	NA	0.29	0	0.21	0	NA	NA
NA	NA	NA	NA	NA	NA	0.25	0	0.21	0	NA	NA
NA	NA	NA	NA	NA	NA	0.26	0	0.21	0	NA	NA
NA	NA	NA	NA	NA	NA	0.25	0	0.21	0	NA	NA
NA	NA	NA	NA	NA	NA	0.24	0	0.20	0	NA	NA
NA	NA	NA	NA	NA	NA	0.28	0	0.21	0	NA	NA
NA	NA	NA	NA	NA	NA	0.55	0	0.20	0	NA	NA
NA	NA	NA	NA	NA	NA	0.20	0	0.21	0	NA	NA
NA	NA	NA	NA	NA	NA	0.29	0	0.20	0	NA	NA
NA	NA	NA	NA	NA	NA	0.35	0	0.24	1	NA	NA
NA	NA	NA	NA	NA	NA	0.25	0	0.30	1	NA	NA
NA	NA	NA	NA	NA	NA	0.41	0	0.09	0	NA	NA
NA	NA	NA	NA	NA	NA	0.79	0	0.28	0	NA	NA
NA	NA	NA	NA	NA	NA	0.33	0	0.22	0	NA	NA
NA	NA	NA	NA	NA	NA	0.20	0	0.22	0	NA	NA
NA	NA	NA	NA	NA	NA	0.21	0	0.20	0	NA	NA
NA	NA	NA	NA	NA	NA	0.28	0	0.09	1	NA	NA
NA	NA	NA	NA	NA	NA	0.26	0	0.23	0	NA	NA
NA	NA	NA	NA	NA	NA	0.39	0	0.25	0	NA	NA
NA	NA	NA	NA	NA	NA	0.25	0	0.24	0	NA	NA
NA	NA	NA	NA	NA	NA	0.25	0	0.20	0	NA	NA
NA	NA	NA	NA	NA	NA	0.27	0	0.20	0	NA	NA
NA	NA	NA	NA	NA	NA	0.36	0	0.20	0	NA	NA
NA	NA	NA	NA	NA	NA	0.22	0	0.19	0	NA	NA
NA	NA	NA	NA	NA	NA	0.25	0	0.20	0	NA	NA
NA	NA	NA	NA	NA	NA	0.49	0	0.18	0	NA	NA
NA	NA	NA	NA	NA	NA	0.30	0	0.20	0	NA	NA
NA	NA	NA	NA	NA	NA	0.28	0	0.18	0	NA	NA
NA	NA	NA	NA	NA	NA	0.82	0	0.23	0	NA	NA
NA	NA	NA	NA	NA	NA	0.23	0	0.26	0	NA	NA
NA	NA	NA	NA	NA	NA	0.25	0	0.22	0	NA	NA
NA	NA	NA	NA	NA	NA	0.32	0	0.21	0	NA	NA
NA	NA	NA	NA	NA	NA	0.33	0	0.20	0	NA	NA
NA	NA	NA	NA	NA	NA	0.40	0	0.19	0	NA	NA
NA	NA	NA	NA	NA	NA	0.24	0	0.21	0	NA	NA
NA	NA	NA	NA	NA	NA	0.24	0	0.19	0	NA	NA
NA	NA	NA	NA	NA	NA	0.33	0	0.15	1	NA	NA
NA	NA	NA	NA	NA	NA	0.28	0	0.16	0	NA	NA
NA	NA	NA	NA	NA	NA	0.41	0	0.22	0	NA	NA

time A	leuko A	time A	leuko A	time B	leuko B	time C	leuko C	time D	leuko D	time E	leuko E
NA	NA	NA	NA	NA	NA	0.25	0	0.14	0	NA	NA
NA	NA	NA	NA	NA	NA	0.23	0	0.19	0	NA	NA
NA	NA	NA	NA	NA	NA	0.27	0	0.20	0	NA	NA
NA	NA	NA	NA	NA	NA	0.23	0	0.20	0	NA	NA
NA	NA	NA	NA	NA	NA	0.24	0	0.14	1	NA	NA
NA	NA	NA	NA	NA	NA	0.38	0	0.20	0	NA	NA
NA	NA	NA	NA	NA	NA	0.29	0	0.25	0	NA	NA
NA	NA	NA	NA	NA	NA	0.39	0	0.22	0	NA	NA
NA	NA	NA	NA	NA	NA	0.32	0	0.20	0	NA	NA
NA	NA	NA	NA	NA	NA	0.22	0	0.19	0	NA	NA
NA	NA	NA	NA	NA	NA	0.22	0	0.21	0	NA	NA
NA	NA	NA	NA	NA	NA	0.22	0	0.19	0	NA	NA
NA	NA	NA	NA	NA	NA	0.24	0	0.21	0	NA	NA
NA	NA	NA	NA	NA	NA	0.40	0	0.22	0	NA	NA
NA	NA	NA	NA	NA	NA	0.21	0	0.19	0	NA	NA
NA	NA	NA	NA	NA	NA	0.22	0	0.22	0	NA	NA
NA	NA	NA	NA	NA	NA	0.26	0	0.20	0	NA	NA
NA	NA	NA	NA	NA	NA	0.43	0	0.21	0	NA	NA
NA	NA	NA	NA	NA	NA	0.32	0	0.19	0	NA	NA
NA	NA	NA	NA	NA	NA	0.34	0	0.21	0	NA	NA
NA	NA	NA	NA	NA	NA	0.31	0	0.19	0	NA	NA
NA	NA	NA	NA	NA	NA	0.26	0	0.22	0	NA	NA
NA	NA	NA	NA	NA	NA	0.26	0	0.16	0	NA	NA
NA	NA	NA	NA	NA	NA	0.21	0	0.21	0	NA	NA
NA	NA	NA	NA	NA	NA	0.23	0	0.17	0	NA	NA
NA	NA	NA	NA	NA	NA	0.25	0	0.11	1	NA	NA
NA	NA	NA	NA	NA	NA	0.30	0	0.17	0	NA	NA
NA	NA	NA	NA	NA	NA	0.35	0	0.23	0	NA	NA
NA	NA	NA	NA	NA	NA	0.17	0	0.19	0	NA	NA
NA	NA	NA	NA	NA	NA	0.26	0	0.22	0	NA	NA
NA	NA	NA	NA	NA	NA	0.23	0	0.20	0	NA	NA
NA	NA	NA	NA	NA	NA	0.27	0	0.20	0	NA	NA
NA	NA	NA	NA	NA	NA	0.29	0	0.21	0	NA	NA
NA	NA	NA	NA	NA	NA	0.27	0	0.19	0	NA	NA
NA	NA	NA	NA	NA	NA	0.24	0	0.20	0	NA	NA
NA	NA	NA	NA	NA	NA	0.23	0	0.20	0	NA	NA
NA	NA	NA	NA	NA	NA	0.25	0	0.19	0	NA	NA
NA	NA	NA	NA	NA	NA	0.34	0	0.18	0	NA	NA
NA	NA	NA	NA	NA	NA	0.26	0	0.18	0	NA	NA
NA	NA	NA	NA	NA	NA	0.24	0	0.18	0	NA	NA
NA	NA	NA	NA	NA	NA	0.22	0	0.20	0	NA	NA
NA	NA	NA	NA	NA	NA	0.26	0	0.17	1	NA	NA
NA	NA	NA	NA	NA	NA	0.22	0	0.19	0	NA	NA
NA	NA	NA	NA	NA	NA	0.29	0	0.35	0	NA	NA
NA	NA	NA	NA	NA	NA	0.46	0	0.30	0	NA	NA
NA	NA	NA	NA	NA	NA	0.23	0	0.22	0	NA	NA
NA	NA	NA	NA	NA	NA	0.27	0	NA	NA	NA	NA
NA	NA	NA	NA	NA	NA	0.25	0	NA	NA	NA	NA
NA	NA	NA	NA	NA	NA	0.29	0	NA	NA	NA	NA
NA	NA	NA	NA	NA	NA	0.29	0	NA	NA	NA	NA
NA	NA	NA	NA	NA	NA	0.33	0	NA	NA	NA	NA

time A	leuko A	time A	leuko A	time B	leuko B	time C	leuko C	time D	leuko D	time E	leuko E
NA	NA	NA	NA	NA	NA	0.36	0	NA	NA	NA	NA
NA	NA	NA	NA	NA	NA	0.23	0	NA	NA	NA	NA
NA	NA	NA	NA	NA	NA	0.28	0	NA	NA	NA	NA
NA	NA	NA	NA	NA	NA	0.30	0	NA	NA	NA	NA
NA	NA	NA	NA	NA	NA	0.26	0	NA	NA	NA	NA
NA	NA	NA	NA	NA	NA	0.31	0	NA	NA	NA	NA
NA	NA	NA	NA	NA	NA	1.14	0	NA	NA	NA	NA
NA	NA	NA	NA	NA	NA	0.26	0	NA	NA	NA	NA
NA	NA	NA	NA	NA	NA	0.24	0	NA	NA	NA	NA

1.5 OPT257

Table 5: Raw data of TBS OPT257.

time A	leuko A	time A	leuko A	time B	leuko B	time C	leuko C	time D	leuko D	time E	leuko E
0.00	1	0.00	1	0.00	1	0.00	1	0.00	1	0.00	1
0.25	1	0.28	1	0.28	1	0.29	1	0.23	1	0.27	1
0.21	1	0.33	1	0.22	1	0.38	1	0.23	1	0.26	1
0.15	1	0.28	1	0.25	1	0.27	1	0.22	1	0.27	1
0.19	1	0.19	1	0.45	1	0.38	1	0.41	1	0.25	1
0.26	1	0.17	1	0.27	1	0.23	1	0.22	1	0.25	1
0.21	1	0.18	1	0.24	1	0.38	1	0.26	1	0.53	1
0.20	1	0.21	1	0.23	1	0.31	1	0.23	1	0.26	1
4.49	1	0.20	1	0.21	1	0.20	1	0.29	1	0.25	1
0.24	1	0.72	1	0.28	1	0.36	1	0.34	1	0.25	1
0.19	1	0.23	1	3.29	1	0.20	1	0.77	1	0.22	1
0.20	1	0.26	1	4.80	1	0.07	1	0.24	1	0.08	1
0.19	1	1.77	1	0.30	1	0.14	1	0.32	1	5.42	1
0.20	1	0.22	1	0.24	1	0.07	1	0.20	1	0.26	1
0.38	1	0.16	1	0.25	1	0.10	1	0.24	1	0.30	1
0.18	1	0.17	1	7.61	1	0.27	1	3.43	1	0.26	1
7.20	1	0.16	1	0.27	1	0.38	1	9.57	1	0.38	1
0.22	1	0.16	1	0.22	1	0.26	1	0.21	1	0.29	1
0.20	1	0.14	1	0.23	1	0.12	1	0.44	1	0.29	1
0.20	1	0.17	1	0.21	1	8.61	0	0.26	1	0.29	1
0.19	1	0.17	1	0.30	1	0.64	1	0.71	1	0.28	1
0.18	1	0.16	1	0.24	1	0.08	1	0.22	1	0.29	1
0.19	1	0.16	1	0.23	1	0.11	1	0.23	1	5.70	1
0.18	1	0.17	1	0.19	1	0.23	1	0.46	1	0.25	1
0.19	1	0.16	1	0.26	1	0.20	1	0.39	1	0.25	1
2.51	1	0.17	1	0.23	1	0.20	1	0.46	1	0.25	1
0.21	1	0.20	1	0.21	1	0.37	1	1.12	1	0.26	1
0.17	1	1.52	1	0.20	1	0.21	1	9.07	1	0.27	1
0.17	1	0.22	1	4.85	1	0.26	1	0.20	1	0.29	1
0.17	1	0.17	1	0.27	1	0.23	1	0.29	1	0.32	1
0.16	1	0.18	1	0.24	1	0.21	1	0.73	1	0.26	1
0.15	1	0.17	1	0.20	1	0.27	1	0.29	1	0.25	1
0.16	1	0.18	1	0.27	1	5.33	1	0.23	1	6.44	1
0.15	1	0.14	1	0.24	1	0.19	1	0.28	1	0.28	1
0.17	1	0.19	1	0.48	1	0.21	1	0.26	1	0.64	1
0.32	1	0.17	1	5.57	1	0.26	1	0.23	1	0.28	1
0.16	1	0.16	1	0.25	1	0.08	1	0.26	1	0.29	1
0.18	1	0.14	1	0.22	1	0.12	1	0.30	1	0.27	1
0.18	1	2.07	1	0.23	1	0.20	1	0.56	1	0.27	1
2.88	1	0.26	1	0.21	1	0.22	1	0.24	1	0.28	1
0.20	1	0.19	1	0.22	1	0.22	1	0.22	1	7.15	1
0.19	1	0.18	1	0.22	1	0.19	1	0.28	1	0.26	1
0.34	1	0.18	1	2.50	1	0.20	1	0.35	1	0.26	1
0.20	1	0.16	1	0.30	1	0.19	1	1.30	1	0.29	1
0.17	1	0.19	1	0.29	1	0.21	1	8.16	1	0.32	1
0.18	1	1.86	1	0.27	1	0.20	1	0.27	1	0.30	1
0.19	1	0.24	1	0.33	1	0.20	1	0.59	1	0.30	1
0.18	1	0.19	1	0.26	1	0.24	1	0.20	1	0.27	1

time A	leuko A	time A	leuko A	time B	leuko B	time C	leuko C	time D	leuko D	time E	leuko E
0.18	1	0.17	1	0.24	1	0.23	1	0.18	1	0.30	1
0.13	1	0.18	1	0.30	1	0.24	1	0.55	1	0.25	1
0.18	1	0.19	1	0.39	1	0.22	1	0.28	1	0.23	1
0.19	1	0.16	1	0.33	1	0.22	1	0.54	1	6.05	1
0.18	1	0.18	1	3.63	1	0.22	1	0.33	1	0.29	1
0.20	1	0.19	1	0.33	1	0.21	1	0.21	1	0.24	1
0.19	1	0.20	1	0.26	1	0.20	1	0.20	1	0.30	1
0.18	1	0.18	1	0.24	1	0.29	1	16.24	1	0.31	1
2.13	1	0.18	1	0.35	1	0.80	1	0.23	1	0.26	1
0.21	1	0.18	1	0.27	1	0.32	1	0.23	1	0.28	1
0.19	1	0.16	1	0.25	1	3.77	1	0.26	1	0.30	1
0.17	1	3.34	1	0.22	1	0.22	1	0.23	1	0.25	1
0.19	1	0.28	1	0.37	1	0.21	1	0.22	1	0.26	1
0.18	1	0.15	1	0.26	1	0.25	1	0.22	1	0.28	1
0.18	1	0.18	1	0.40	1	0.20	1	0.19	1	0.30	1
0.18	1	0.17	1	0.27	1	0.20	1	0.23	1	0.27	1
0.16	1	0.17	1	0.24	1	0.22	1	0.21	1	0.32	1
6.56	1	0.16	1	0.23	1	0.22	1	0.21	1	5.99	1
0.23	1	0.19	1	3.21	1	0.22	1	0.19	1	0.27	1
0.19	1	0.17	1	0.28	1	0.22	1	0.21	1	0.27	1
0.21	1	0.17	1	0.25	1	0.22	1	0.48	1	0.27	1
0.19	1	0.16	1	0.24	1	0.23	1	0.20	1	0.27	1
0.21	1	2.35	1	0.23	1	0.23	1	8.78	1	0.24	1
0.16	1	0.25	1	0.30	1	0.20	1	0.21	1	0.45	1
0.17	1	0.20	1	0.23	1	0.22	1	0.17	1	0.29	1
0.17	1	0.32	1	3.15	1	0.21	1	0.43	1	0.26	1
0.17	1	0.19	1	0.29	1	0.43	1	0.19	1	0.28	1
0.17	1	0.17	1	0.45	1	0.22	1	1.78	1	0.31	1
2.21	1	0.18	1	0.27	1	0.06	1	0.22	1	0.27	1
0.25	1	0.18	1	0.25	1	0.27	1	0.19	1	3.83	1
0.18	1	0.18	1	0.27	1	5.81	1	0.21	1	0.25	1
0.17	1	0.17	1	0.25	1	0.22	1	1.00	1	0.29	1
0.16	1	0.18	1	2.53	1	0.25	1	0.19	1	0.28	1
0.19	1	0.16	1	0.26	1	0.21	1	0.25	1	0.28	1
0.12	1	0.19	1	0.22	1	0.06	1	0.18	1	0.28	1
0.17	1	0.18	1	0.22	1	0.12	1	0.48	1	0.30	1
0.15	1	2.68	1	0.21	1	0.31	1	0.20	1	5.50	1
0.18	1	0.19	1	0.36	1	0.42	1	0.17	1	0.26	1
0.16	1	0.19	1	0.24	1	0.20	1	0.31	1	0.27	1
0.17	1	0.35	1	0.23	1	0.22	1	0.22	1	0.27	1
0.18	1	0.20	1	0.21	1	0.21	1	0.20	1	0.27	1
0.17	1	0.18	1	0.21	1	0.19	1	8.11	1	0.27	1
0.16	1	0.18	1	0.28	1	1.71	1	0.24	1	0.27	1
0.20	1	0.20	1	0.20	1	0.26	1	0.19	1	0.26	1
1.91	1	0.17	1	0.23	1	1.03	1	0.20	1	0.24	1
0.42	1	0.17	1	0.33	1	5.17	0	0.16	1	0.27	1
0.19	1	0.20	1	0.26	1	0.64	1	0.19	1	3.47	1
0.19	1	0.19	1	0.24	1	0.10	1	0.22	1	0.31	1
0.15	1	0.18	1	0.21	1	0.15	1	0.20	1	0.27	1
0.17	1	0.18	1	3.85	1	0.21	1	0.55	1	0.24	1
0.16	1	0.18	1	0.28	1	0.21	1	0.24	1	0.25	1

time A	leuko A	time A	leuko A	time B	leuko B	time C	leuko C	time D	leuko D	time E	leuko E
0.15	1	0.19	1	0.25	1	0.21	1	0.18	1	3.72	1
0.12	1	0.19	1	0.24	1	0.23	1	0.20	1	0.26	1
0.18	1	0.20	1	0.23	1	0.75	1	8.66	1	0.29	1
0.17	1	0.17	1	0.34	1	0.23	1	0.23	1	0.84	1
0.18	1	2.03	1	0.25	1	0.27	1	0.33	1	0.32	1
0.16	1	0.20	1	0.23	1	0.96	1	0.24	1	0.62	1
0.20	1	0.18	1	0.24	1	0.22	1	0.35	1	0.24	1
0.17	1	0.16	1	0.23	1	0.19	1	0.22	1	0.32	1
0.17	1	0.19	1	0.35	1	0.10	1	0.27	1	0.27	1
0.18	1	0.18	1	0.28	1	0.16	1	0.28	1	1.16	1
5.41	1	0.19	1	7.90	1	0.21	1	0.23	1	4.33	1
0.24	1	0.17	1	0.28	1	0.20	1	0.22	1	0.28	1
0.19	1	0.19	1	0.25	1	0.57	1	0.20	1	0.27	1
0.16	1	0.19	1	0.22	1	3.81	1	0.19	1	0.26	1
0.18	1	0.19	1	0.23	1	0.22	1	11.65	1	0.29	1
0.18	1	0.17	1	0.38	1	0.22	1	0.23	1	0.30	1
0.17	1	0.19	1	0.26	1	0.23	1	0.31	1	0.31	1
0.20	1	0.20	1	0.23	1	0.20	1	0.23	1	0.28	1
0.18	1	0.17	1	0.21	1	0.21	1	0.37	1	0.28	1
0.15	1	22.19	1	0.35	1	0.20	1	0.23	1	0.89	1
0.18	1	0.29	1	0.24	1	0.22	1	0.57	1	0.29	1
0.19	1	0.36	1	0.23	1	0.40	1	0.20	1	0.24	1
0.18	1	0.19	1	0.21	1	0.20	1	0.33	1	4.51	1
0.16	1	0.17	1	5.30	1	0.22	1	0.20	1	0.24	1
0.17	1	0.19	1	0.27	1	0.21	1	0.27	1	0.27	1
0.19	1	0.17	1	0.24	1	0.20	1	0.28	1	0.27	1
5.68	1	0.11	1	0.61	1	0.24	1	0.20	1	0.27	1
0.23	1	0.24	1	0.25	1	0.24	1	0.20	1	0.29	1
0.18	1	0.22	1	0.23	1	0.22	1	0.30	1	0.46	1
0.20	1	0.22	1	0.22	1	0.21	1	0.21	1	0.32	1
0.19	1	0.13	1	0.22	1	0.20	1	0.79	1	0.30	1
0.18	1	0.23	1	0.25	1	0.22	1	0.20	1	0.31	1
0.17	1	0.15	1	0.24	1	0.20	1	0.20	1	0.28	1
0.17	1	0.18	1	0.39	1	0.21	1	0.19	1	0.30	1
0.18	1	0.20	1	0.25	1	0.07	1	0.16	1	0.25	1
0.19	1	0.18	1	0.20	1	0.12	1	7.30	1	5.01	1
0.19	1	0.18	1	8.45	1	0.42	1	0.20	1	0.25	1
0.19	1	0.19	1	0.28	1	0.22	1	0.16	1	0.29	1
0.20	1	0.22	1	0.23	1	0.21	1	0.20	1	0.40	1
0.18	1	7.00	1	0.20	1	0.22	1	0.31	1	0.39	1
0.18	1	0.26	1	0.36	1	0.20	1	0.56	1	0.29	1
0.19	1	0.40	1	0.25	1	0.21	1	0.20	1	0.40	1
0.23	1	0.21	1	0.37	1	0.38	1	0.19	1	0.35	1
0.20	1	0.20	1	0.24	1	0.26	1	0.17	1	0.30	1
0.19	1	0.16	1	0.17	1	0.33	1	0.33	1	0.25	1
6.53	1	0.21	1	5.04	1	3.89	1	0.18	1	0.27	1
0.25	1	0.14	1	0.25	1	0.26	1	0.19	1	0.27	1
0.17	1	0.14	1	0.24	1	0.06	1	0.17	1	0.30	1
0.18	1	0.24	1	0.21	1	0.13	1	0.19	1	0.30	1
0.14	1	0.37	1	0.21	1	0.21	1	0.19	1	0.28	1
0.27	1	0.18	1	0.28	1	0.30	1	0.30	1	3.85	1

time A	leuko A	time A	leuko A	time B	leuko B	time C	leuko C	time D	leuko D	time E	leuko E
0.22	1	0.20	1	0.22	1	0.21	1	0.60	1	0.31	1
0.18	1	0.18	1	0.21	1	0.19	1	1.71	1	0.31	1
0.19	1	0.21	1	0.20	1	0.27	1	0.21	1	0.29	1
3.21	1	0.19	1	0.21	1	0.19	1	0.18	1	0.28	1
0.24	1	21.59	1	0.26	1	0.25	1	0.47	1	0.30	1
0.18	1	0.26	1	0.22	1	0.67	1	0.39	1	0.34	1
0.19	1	0.18	1	0.19	1	0.22	1	0.34	1	0.31	1
0.18	1	0.21	1	6.52	1	0.21	1	0.35	1	0.32	1
0.19	1	0.19	1	0.25	1	0.23	1	0.22	1	0.25	1
0.18	1	0.19	1	0.21	1	0.24	1	0.18	1	5.11	1
0.21	1	0.20	1	0.27	1	0.22	1	12.05	1	0.27	1
0.55	1	0.15	1	0.24	1	0.21	1	0.24	1	0.29	1
0.20	1	0.21	1	0.17	1	0.54	1	0.40	1	0.27	1
2.45	1	0.20	1	0.25	1	6.49	1	0.22	1	0.26	1
0.32	1	0.22	1	0.22	1	0.21	1	0.23	1	0.29	1
0.18	1	6.48	1	0.21	1	0.40	1	0.22	1	0.44	1
0.39	1	0.31	1	0.20	1	0.20	1	0.19	1	21.20	1
0.20	1	0.14	1	4.58	1	0.21	1	9.69	1	0.27	1
0.17	1	0.31	1	0.31	1	0.33	1	0.21	1	0.29	1
0.21	1	0.21	1	0.24	1	0.19	1	0.21	1	0.31	1
0.32	1	0.22	1	0.22	1	0.25	1	0.21	1	0.34	1
0.22	1	0.19	1	0.23	1	0.24	1	0.41	1	0.32	1
0.29	1	0.20	1	0.49	1	0.23	1	0.19	1	0.30	1
2.08	0	0.20	1	0.28	1	0.22	1	0.26	1	3.84	1
1.10	1	0.15	1	0.24	1	0.22	1	0.18	1	0.27	1
0.20	1	0.32	1	4.44	1	4.19	1	0.29	1	0.25	1
0.52	1	0.22	1	0.28	1	0.21	1	0.27	1	3.21	1
4.24	1	0.20	1	0.24	1	0.22	1	0.26	1	0.26	1
0.20	1	0.23	1	0.22	1	0.25	1	0.25	1	0.27	1
0.19	1	0.21	1	0.32	1	0.21	1	0.29	1	0.27	1
0.16	1	0.22	1	0.25	1	0.49	1	0.24	1	0.30	1
0.18	1	0.19	1	0.23	1	0.21	1	0.21	1	0.27	1
0.20	1	0.18	1	0.23	1	0.19	1	0.21	1	0.28	1
0.18	1	4.58	1	0.49	1	0.20	1	0.20	1	0.29	1
0.16	1	0.29	1	0.27	1	0.20	1	0.18	1	0.28	1
0.18	1	0.21	1	0.23	1	0.27	1	0.21	1	0.28	1
0.18	1	0.11	1	26.14	1	0.08	1	14.04	1	5.40	1
0.18	1	0.30	1	0.28	1	0.10	1	0.20	1	0.28	1
0.18	1	0.20	1	0.27	1	7.64	1	0.20	1	0.30	1
0.18	1	0.22	1	0.23	1	0.21	1	0.16	1	0.29	1
0.20	1	0.19	1	0.86	1	0.33	1	0.20	1	0.29	1
0.18	1	0.20	1	0.26	1	0.38	1	0.17	1	0.11	1
3.17	1	0.20	1	0.25	1	0.21	1	0.20	1	0.15	1
0.24	1	0.22	1	0.20	1	0.09	1	0.19	1	0.28	1
0.20	1	0.21	1	0.37	1	1.14	1	0.19	1	0.31	1
0.20	1	0.23	1	0.24	1	0.09	1	0.16	1	0.25	1
0.19	1	0.23	1	0.40	1	0.10	1	0.19	1	5.69	1
0.17	1	4.38	1	0.22	1	0.22	1	0.18	1	0.28	1
0.19	1	0.25	1	0.22	1	0.58	1	0.20	1	0.26	1
0.21	1	0.19	1	10.72	1	0.22	1	0.19	1	0.27	1
0.17	1	0.19	1	0.30	1	0.20	1	0.16	1	0.33	1

time A	leuko A	time A	leuko A	time B	leuko B	time C	leuko C	time D	leuko D	time E	leuko E
0.17	1	0.18	1	0.24	1	0.19	1	0.19	1	0.35	1
0.17	1	0.18	1	0.22	1	0.20	1	0.19	1	0.29	1
0.18	1	0.19	1	0.20	1	0.31	1	0.19	1	8.22	1
0.18	1	0.17	1	0.36	1	0.10	1	0.18	1	0.30	1
0.19	1	0.16	1	0.23	1	0.15	1	0.16	1	0.15	1
0.19	1	0.18	1	0.23	1	0.10	1	0.18	1	0.16	1
0.18	1	0.20	1	1.17	1	1.74	1	0.14	1	0.31	1
0.19	1	0.18	1	0.28	1	2.62	1	6.02	1	0.32	1
0.19	1	0.18	1	0.30	1	0.23	1	0.19	1	0.29	1
0.18	1	0.17	1	0.21	1	0.20	1	0.15	1	0.29	1
3.34	1	0.17	1	0.21	1	0.20	1	0.19	1	0.13	1
0.45	1	0.28	1	6.63	1	0.23	1	0.18	1	0.16	1
0.23	1	8.18	1	0.26	1	0.21	1	0.20	1	0.33	1
0.17	1	0.26	1	0.24	1	0.22	1	0.21	1	0.28	1
0.19	1	0.20	1	0.22	1	0.20	1	0.19	1	0.11	1
0.19	1	0.13	1	0.20	1	0.22	1	0.20	1	0.15	1
0.17	1	0.32	1	0.27	1	0.46	1	0.29	1	0.27	1
0.67	1	0.22	1	0.23	1	0.09	1	0.21	1	0.09	1
1.24	1	0.21	1	0.22	1	0.11	1	0.19	1	0.24	1
0.24	1	0.21	1	0.20	1	0.11	1	0.16	1	0.31	1
0.19	1	0.20	1	0.20	1	0.15	1	0.19	1	0.29	1
5.59	1	0.20	1	0.22	1	0.24	1	0.22	1	3.88	1
0.23	1	0.19	1	0.20	1	0.04	1	1.98	1	0.27	1
0.14	1	0.19	1	0.22	1	0.14	1	0.18	1	0.37	1
0.16	1	0.21	1	0.25	1	0.27	1	0.16	1	0.31	1
0.17	1	0.36	1	0.22	1	0.23	1	0.28	1	0.32	1
0.17	1	0.34	1	0.19	1	0.09	1	0.20	1	0.32	1
0.19	1	0.20	1	0.21	1	0.11	1	0.16	1	0.30	1
0.17	1	0.20	1	3.83	1	0.29	1	0.28	1	0.30	1
0.17	1	0.22	1	0.27	1	0.09	1	0.19	1	0.31	1
0.21	1	6.69	1	0.24	1	10.39	1	0.17	1	0.29	1
0.21	1	0.28	1	0.52	1	0.20	1	0.25	1	3.09	1
0.21	1	0.19	1	0.26	1	0.20	1	0.22	1	0.27	1
0.22	1	0.22	1	0.23	1	0.21	1	0.18	1	0.27	1
0.35	1	0.21	1	0.38	1	0.30	1	0.20	1	0.10	1
0.23	1	0.18	1	0.26	1	0.15	1	0.17	1	0.16	1
2.12	1	0.27	1	0.25	1	0.09	1	0.16	1	0.26	1
0.21	1	0.22	1	0.24	1	0.10	1	0.17	1	0.28	1
0.20	1	0.21	1	0.22	1	0.19	1	0.16	1	0.26	1
0.20	1	0.20	1	0.22	1	0.19	1	2.30	1	0.27	1
0.20	1	0.21	1	0.31	1	0.20	1	0.18	1	0.26	1
0.19	1	0.22	1	0.24	1	0.20	1	0.19	1	0.36	1
0.19	1	0.19	1	0.22	1	0.22	1	0.18	1	7.94	1
0.18	1	0.23	1	0.23	1	0.25	1	0.17	1	0.30	1
0.20	1	0.23	1	0.27	1	0.10	1	0.19	1	0.46	1
0.18	1	0.21	1	0.21	1	0.01	1	0.20	1	6.15	1
0.17	1	0.20	1	4.21	1	0.09	1	0.21	1	0.28	1
0.18	1	0.23	1	0.27	1	0.21	1	0.18	1	0.26	1
0.20	1	0.26	1	0.19	1	0.22	1	0.19	1	0.26	1
2.80	1	0.31	1	0.74	1	0.24	1	0.17	1	0.25	1
0.20	1	3.22	1	0.25	1	0.21	1	8.30	1	0.24	1

time A	leuko A	time A	leuko A	time B	leuko B	time C	leuko C	time D	leuko D	time E	leuko E
0.20	1	0.21	1	0.23	1	0.11	1	0.22	1	5.63	1
0.17	1	0.18	1	0.31	1	0.19	1	0.21	1	0.26	1
0.18	1	0.18	1	0.23	1	0.10	1	1.20	1	0.32	1
0.18	1	0.20	1	0.20	1	4.25	1	0.21	1	0.28	1
0.18	1	0.20	1	8.73	1	0.06	1	0.23	1	0.27	1
0.18	1	0.17	1	0.25	1	0.13	1	0.20	1	0.29	1
0.20	1	0.17	1	0.22	1	0.21	1	0.31	1	0.25	1
0.19	1	0.20	1	0.22	1	0.07	1	0.18	1	0.09	1
0.19	1	0.18	1	0.21	1	0.11	1	0.23	1	0.14	1
0.19	1	0.22	1	0.33	1	0.05	1	0.32	1	0.26	1
0.19	1	0.10	1	0.22	1	0.13	1	0.99	1	3.32	1
0.23	1	0.25	1	0.47	1	0.22	1	0.20	1	0.32	1
0.19	1	4.83	1	0.36	1	0.18	1	0.36	1	0.27	1
0.20	1	0.25	1	0.22	1	0.09	1	0.21	1	0.09	1
0.20	1	0.40	1	0.21	1	0.13	1	4.33	1	0.18	1
3.92	1	0.20	1	0.21	1	0.20	1	0.25	1	0.01	1
0.23	1	0.18	1	11.27	1	0.09	1	0.30	1	0.24	1
0.18	1	0.21	1	0.24	1	0.12	1	0.53	1	0.26	1
0.20	1	0.20	1	0.20	1	0.08	1	0.65	1	0.25	1
0.19	1	0.21	1	0.26	1	0.12	1	0.27	1	0.29	1
0.19	1	0.19	1	0.22	1	0.09	1	0.20	1	0.29	1
0.18	1	0.20	1	0.19	1	0.11	1	0.24	1	0.28	1
0.19	1	0.20	1	0.19	1	0.09	1	0.34	1	0.01	1
0.18	1	0.19	1	0.25	1	0.11	1	0.25	1	0.22	1
0.20	1	0.20	1	0.20	1	0.31	1	0.21	1	4.01	1
0.20	1	0.20	1	0.28	1	0.24	1	2.86	1	0.26	1
0.20	1	0.20	1	0.22	1	0.22	1	0.26	1	0.26	1
0.31	1	0.20	1	0.19	1	0.25	1	0.32	1	0.24	1
0.22	1	0.18	1	0.57	1	0.09	1	9.20	1	0.10	1
0.18	1	0.22	1	0.26	1	0.11	1	0.22	1	2.77	1
0.19	1	2.85	1	0.23	1	0.26	1	0.18	1	0.33	1
0.18	1	0.28	1	0.21	1	0.27	1	0.18	1	0.30	1
0.19	1	0.24	1	0.26	1	0.27	1	0.25	1	0.28	1
0.20	1	0.22	1	0.20	1	0.25	1	0.37	1	0.30	1
0.19	1	0.21	1	0.20	1	0.32	1	0.59	1	0.27	1
0.17	1	0.19	1	0.22	1	4.57	1	0.18	1	0.27	1
0.22	1	0.23	1	0.19	1	0.09	1	0.18	1	0.27	1
2.95	1	0.21	1	8.20	1	0.11	1	0.25	1	2.71	1
0.24	1	6.56	1	0.27	1	0.06	1	0.20	1	0.25	1
0.19	1	0.24	1	0.24	1	0.11	1	0.18	1	0.25	1
0.18	1	0.23	1	0.21	1	0.21	1	0.24	1	0.27	1
0.17	1	0.19	1	0.28	1	0.20	1	0.21	1	0.25	1
0.17	1	0.19	1	0.21	1	0.48	1	0.23	1	0.25	1
0.17	1	0.18	1	0.22	1	0.23	1	0.29	1	0.23	1
0.19	1	0.20	1	0.21	1	0.31	1	0.24	1	0.25	1
0.16	1	0.20	1	0.18	1	3.28	1	0.25	1	0.27	1
0.20	1	0.18	1	0.29	1	0.20	1	0.22	1	0.23	1
0.18	1	0.16	1	0.19	1	0.24	1	0.20	1	0.25	1
0.19	1	0.30	1	0.24	1	0.19	1	0.21	1	0.23	1
0.22	1	0.33	1	0.23	1	0.06	1	0.22	1	0.22	1
0.31	1	0.24	1	0.20	1	0.12	1	0.22	1	0.07	1

time A	leuko A	time A	leuko A	time B	leuko B	time C	leuko C	time D	leuko D	time E	leuko E
0.23	1	0.20	1	0.21	1	0.20	1	0.26	1	2.77	1
2.41	1	0.21	1	0.20	1	0.06	1	0.33	1	0.26	1
0.25	1	7.56	1	0.25	1	0.13	1	0.22	1	0.28	1
0.19	1	0.22	1	0.23	1	2.44	1	0.23	1	0.27	1
0.21	1	0.22	1	0.20	1	0.10	1	0.35	1	0.27	1
0.16	1	0.20	1	0.21	1	0.10	1	0.36	1	0.11	1
0.20	1	0.19	1	0.19	1	0.08	1	0.23	1	0.15	1
0.20	1	0.20	1	0.20	1	0.11	1	0.49	1	0.26	1
0.19	1	0.21	1	4.47	1	0.29	1	0.23	1	0.01	1
0.19	1	0.22	1	0.27	1	0.39	1	0.25	1	0.26	1
0.18	1	0.17	1	0.24	1	0.21	1	0.25	1	0.55	1
0.22	1	0.20	1	0.22	1	0.27	1	0.35	1	3.22	1
0.18	1	0.19	1	0.28	1	4.92	1	0.54	1	0.21	1
0.19	1	0.20	1	0.22	1	0.29	1	0.24	1	0.32	1
0.22	1	0.21	1	0.23	1	0.24	1	0.25	1	0.71	1
0.19	1	0.18	1	0.22	1	0.20	1	0.25	1	0.38	1
0.19	1	0.20	1	0.74	1	1.65	1	0.25	1	0.29	1
0.19	1	0.22	1	0.28	1	0.27	1	0.19	1	0.27	1
0.20	1	2.06	1	0.23	1	0.23	1	0.30	1	0.26	1
0.23	1	0.22	1	0.24	1	0.21	1	12.42	1	0.28	1
3.98	1	0.17	1	0.20	1	0.21	1	0.24	1	0.25	1
0.25	1	0.18	1	0.26	1	0.25	1	0.28	1	0.10	1
0.21	1	0.20	1	0.22	1	0.17	1	0.40	1	3.23	1
0.19	1	0.20	1	0.21	1	0.17	1	0.20	1	0.23	1
0.17	1	0.20	1	0.20	1	0.33	1	0.83	1	0.25	1
0.17	1	0.20	1	0.19	1	0.25	1	0.18	1	0.27	1
0.19	1	0.22	1	0.26	1	0.07	1	0.30	1	0.27	1
0.19	1	0.19	1	0.18	1	0.10	1	0.46	1	0.10	1
0.19	1	0.20	1	0.29	1	0.23	1	0.26	1	0.14	1
0.24	1	0.20	1	4.90	1	0.19	1	0.42	1	0.25	1
0.19	1	0.19	1	0.27	1	0.06	1	0.29	1	0.10	1
0.18	1	0.20	1	0.22	1	0.13	1	0.26	1	0.16	1
0.17	1	0.19	1	0.48	1	0.26	1	0.23	1	0.10	1
0.34	1	0.20	1	0.24	1	0.25	1	0.30	1	0.17	1
2.79	1	2.20	1	0.22	1	4.03	1	0.32	1	0.26	1
0.19	1	0.25	1	0.23	1	0.09	1	0.27	1	0.24	1
0.20	1	0.22	1	0.21	1	0.11	1	0.24	1	0.09	1
0.14	1	0.20	1	0.27	1	0.06	1	0.30	1	2.11	1
0.16	1	0.21	1	0.21	1	0.14	1	0.23	1	0.25	1
0.19	1	0.20	1	0.91	1	0.09	1	0.23	1	0.29	1
0.18	1	0.21	1	0.26	1	0.12	1	0.23	1	0.25	1
0.18	1	0.21	1	0.24	1	0.09	1	0.23	1	0.25	1
0.18	1	0.21	1	0.23	1	0.12	1	0.25	1	0.27	1
0.19	1	0.17	1	0.22	1	0.21	1	0.23	1	0.25	1
0.15	1	0.21	1	0.26	1	0.52	1	0.26	1	0.26	1
0.21	1	0.21	1	0.20	1	2.57	1	0.56	1	0.26	1
0.16	1	0.33	1	2.86	1	0.20	1	9.96	1	0.28	1
0.16	1	0.23	1	0.27	1	0.22	1	0.17	1	0.29	1
0.35	1	3.18	1	0.25	1	0.08	1	0.19	1	0.26	1
0.17	1	0.23	1	0.24	1	0.11	1	0.25	1	0.24	1
0.19	1	0.20	1	0.34	1	0.07	1	0.18	1	2.55	1

time A	leuko A	time A	leuko A	time B	leuko B	time C	leuko C	time D	leuko D	time E	leuko E
0.19	1	0.18	1	0.24	1	0.95	1	0.25	1	0.25	1
4.08	1	0.20	1	0.25	1	0.08	1	0.19	1	0.29	1
0.19	1	0.18	1	0.21	1	0.50	1	0.20	1	0.28	1
0.19	1	0.21	1	0.31	1	0.08	1	0.19	1	0.30	1
0.19	1	0.21	1	0.27	1	0.14	1	0.17	1	0.29	1
0.17	1	0.21	1	0.20	1	0.08	1	0.19	1	0.27	1
0.18	1	0.23	1	0.27	1	0.13	1	0.18	1	0.28	1
0.18	1	0.20	1	0.24	1	0.23	1	0.20	1	0.26	1
0.18	1	0.21	1	0.20	1	0.23	1	0.18	1	0.27	1
0.19	1	0.19	1	7.43	1	0.07	1	0.16	1	0.01	1
0.18	1	0.22	1	0.30	1	0.13	1	0.19	1	0.25	1
3.83	1	0.19	1	1.00	1	1.72	1	0.17	1	0.34	1
0.22	1	0.19	1	0.31	1	0.23	1	0.20	1	0.25	1
0.22	1	0.21	1	0.26	1	0.22	1	0.20	1	3.00	1
0.19	1	6.65	1	0.22	1	0.19	1	0.19	1	0.24	1
0.21	1	0.24	1	5.49	1	0.23	1	0.20	1	0.28	1
0.18	1	0.19	1	0.26	1	0.06	1	0.18	1	0.29	1
0.17	1	0.20	1	0.20	1	0.12	1	0.18	1	0.29	1
0.18	1	0.17	1	0.80	1	0.20	1	0.34	1	0.27	1
0.19	1	0.21	1	0.26	1	0.45	1	0.21	1	0.27	1
0.20	1	0.19	1	0.22	1	0.21	1	0.18	1	0.11	1
0.18	1	0.20	1	0.21	1	0.19	1	0.20	1	0.17	1
0.17	1	0.19	1	0.23	1	0.07	1	0.19	1	0.29	1
0.19	1	0.22	1	0.28	1	0.12	1	0.22	1	0.26	1
0.19	1	0.20	1	0.20	1	0.50	1	0.19	1	0.10	1
0.18	1	0.20	1	0.25	1	0.22	1	0.18	1	0.13	1
0.20	1	3.22	1	0.20	1	0.23	1	3.92	1	3.37	1
0.19	1	0.23	1	7.88	1	3.40	1	0.22	1	0.29	1
0.20	1	0.18	1	0.26	1	0.05	1	0.22	1	0.28	1
0.20	1	0.20	1	0.24	1	0.12	1	0.19	1	0.27	1
0.19	1	0.17	1	0.22	1	0.26	1	0.20	1	2.48	1
4.17	1	0.23	1	0.20	1	0.21	1	0.19	1	0.25	1
0.20	1	0.31	1	0.31	1	0.06	1	0.21	1	0.28	1
0.17	1	0.19	1	0.23	1	0.14	1	0.21	1	0.25	1
0.18	1	0.23	1	0.20	1	0.06	1	0.20	1	0.28	1
0.16	1	0.19	1	0.19	1	0.51	1	0.19	1	0.26	1
0.18	1	0.19	1	0.33	1	0.25	1	0.20	1	0.25	1
0.16	1	0.20	1	0.23	1	0.09	1	0.21	1	0.26	1
0.18	1	0.19	1	0.19	1	0.15	1	0.21	1	1.84	1
0.17	1	0.18	1	0.21	1	0.06	1	0.20	1	0.25	1
0.19	1	0.19	1	0.19	1	0.14	1	0.21	1	0.29	1
0.18	1	0.21	1	4.22	1	0.50	1	0.20	1	0.25	1
0.18	1	0.18	1	0.25	1	0.36	1	0.21	1	0.26	1
0.18	1	0.21	1	0.23	1	0.08	1	0.04	1	0.30	1
0.18	1	0.21	1	0.43	1	3.36	1	0.13	1	0.27	1
3.53	1	3.86	1	0.24	1	0.21	1	0.23	1	0.28	1
0.17	1	0.20	1	0.21	1	0.08	1	0.21	1	0.27	1
0.27	1	0.18	1	0.41	1	0.11	1	0.24	1	0.24	1
0.20	1	0.19	1	0.25	1	0.06	1	0.22	1	0.22	1
0.19	1	0.14	1	0.23	1	0.22	1	0.21	1	7.19	1
0.20	1	0.25	1	0.19	1	0.25	1	0.17	1	0.22	1

time A	leuko A	time A	leuko A	time B	leuko B	time C	leuko C	time D	leuko D	time E	leuko E
0.17	1	0.19	1	0.26	1	0.30	1	0.21	1	0.26	1
0.21	1	0.21	1	0.21	1	0.94	1	0.21	1	0.23	1
0.17	1	0.19	1	0.19	1	0.29	1	0.24	1	0.25	1
0.19	1	0.20	1	0.22	1	0.11	1	0.24	1	0.27	1
0.20	1	0.19	1	4.72	1	0.15	1	0.27	1	0.25	1
0.20	1	0.19	1	0.28	1	0.36	1	0.28	1	0.25	1
0.19	1	0.20	1	0.23	1	0.11	1	2.80	1	0.26	1
0.21	1	0.19	1	0.29	1	0.01	1	0.43	1	0.25	1
0.20	1	0.20	1	0.23	1	8.13	1	0.36	1	4.37	1
0.18	1	0.19	1	0.21	1	0.22	1	0.43	1	0.44	1
0.20	1	0.21	1	0.28	1	0.08	1	0.45	1	0.26	1
0.21	1	0.19	1	0.23	1	0.13	1	8.85	1	0.28	1
0.21	1	0.20	1	0.22	1	0.09	1	5.83	1	0.24	1
5.17	1	0.16	1	0.21	1	0.13	1	0.22	1	0.27	1
0.22	1	3.13	1	0.26	1	0.11	1	0.20	1	0.31	1
0.18	1	0.26	1	0.22	1	0.12	1	0.20	1	0.25	1
0.19	1	0.20	1	0.19	1	0.08	1	0.18	1	0.28	1
0.20	1	0.21	1	0.22	1	0.10	1	0.18	1	0.28	1
0.20	1	0.19	1	0.18	1	0.09	1	0.26	1	0.24	1
0.18	1	0.21	1	5.43	1	0.11	1	0.19	1	2.99	1
0.36	1	0.19	1	0.25	1	0.21	1	0.20	1	0.28	1
0.20	1	0.23	1	0.29	1	0.07	1	0.18	1	0.28	1
0.18	1	0.19	1	0.23	1	0.11	1	0.19	1	0.30	1
0.18	1	0.20	1	0.20	1	0.08	1	0.18	1	0.26	1
2.63	1	0.20	1	0.21	1	0.69	1	0.18	1	0.28	1
0.21	1	0.22	1	0.29	1	4.81	1	0.19	1	0.27	1
0.19	1	2.49	1	0.24	1	0.22	1	0.19	1	2.57	1
0.19	1	0.25	1	0.33	1	0.25	1	0.16	1	0.23	1
0.19	1	0.19	1	0.23	1	0.20	1	0.16	1	0.28	1
0.17	1	0.20	1	0.20	1	0.20	1	0.17	1	0.45	1
0.19	1	0.20	1	0.19	1	0.20	1	0.21	1	0.34	1
0.18	1	0.19	1	0.27	1	0.22	1	0.19	1	0.26	1
0.18	1	0.19	1	0.22	1	0.21	1	0.20	1	0.25	1
0.20	1	0.19	1	0.22	1	0.09	1	0.18	1	0.27	1
0.19	1	0.19	1	0.22	1	0.11	1	0.20	1	0.27	1
0.19	1	0.21	1	0.22	1	0.07	1	0.21	1	3.18	1
0.19	1	0.38	1	0.25	1	0.14	1	0.20	1	0.24	1
0.21	1	0.22	1	0.22	1	0.24	1	0.26	1	0.44	1
0.19	1	0.18	1	0.21	1	0.07	1	0.20	1	0.27	1
0.17	1	0.19	1	0.23	1	0.10	1	0.25	1	0.26	1
1.94	1	0.20	1	0.20	1	0.24	1	0.21	1	0.27	1
0.23	1	0.18	1	0.23	1	0.22	1	0.34	1	0.27	1
0.19	1	1.96	1	0.21	1	3.77	1	0.20	1	0.26	1
0.18	1	0.31	1	10.74	1	3.95	1	0.33	1	0.25	1
0.20	1	0.22	1	0.26	1	0.22	1	5.17	1	0.25	1
0.19	1	0.19	1	0.21	1	0.18	1	0.25	1	3.07	1
0.18	1	0.22	1	0.82	1	0.39	1	0.19	1	0.25	1
0.20	1	0.20	1	0.25	1	0.09	1	0.61	1	0.26	1
0.19	1	0.26	1	0.21	1	0.11	1	0.22	1	0.39	1
0.20	1	0.20	1	0.20	1	0.07	1	0.52	1	0.24	1
0.22	1	0.20	1	0.42	1	0.14	1	0.36	1	0.26	1

time A	leuko A	time A	leuko A	time B	leuko B	time C	leuko C	time D	leuko D	time E	leuko E
0.35	1	0.18	1	1.22	1	0.20	1	0.32	1	0.31	1
0.30	1	0.50	1	0.27	1	0.09	1	0.75	1	0.65	1
2.19	1	5.33	1	0.25	1	0.12	1	0.25	1	0.30	1
0.22	1	0.21	1	0.20	1	0.25	1	0.58	1	0.26	1
0.19	1	0.17	1	0.28	1	0.21	1	0.09	1	0.49	1
0.19	1	0.18	1	0.21	1	0.23	1	7.96	1	0.15	1
0.19	1	0.19	1	0.27	1	0.36	1	0.21	1	0.13	1
0.22	1	0.18	1	0.23	1	3.42	1	0.22	1	2.68	1
0.22	1	0.18	1	0.22	1	0.20	1	0.19	1	0.23	1
0.17	1	0.21	1	0.23	1	0.21	1	0.19	1	0.32	1
0.19	1	4.88	1	7.06	1	0.31	1	0.20	1	0.34	1
0.20	1	0.24	1	0.31	1	1.74	1	0.19	1	0.31	1
0.18	1	0.20	1	0.44	1	0.08	1	0.17	1	0.27	1
0.20	1	0.19	1	0.25	1	0.12	1	0.16	1	0.30	1
0.20	1	0.21	1	0.25	1	0.08	1	0.18	1	0.29	1
0.19	1	0.20	1	0.22	1	0.10	1	0.18	1	0.26	1
0.21	1	0.19	1	0.99	1	0.20	1	0.19	1	0.26	1
0.21	1	0.17	1	0.27	1	0.21	1	0.21	1	4.67	1
0.20	1	2.25	1	0.34	1	0.21	1	0.22	1	0.25	1
0.15	1	0.23	1	0.24	1	0.07	1	0.23	1	0.28	1
0.19	1	0.11	1	0.21	1	0.07	1	0.22	1	0.27	1
0.19	1	0.28	1	0.29	1	0.18	1	0.23	1	0.25	1
0.19	1	0.18	1	0.21	1	0.20	1	0.21	1	0.25	1
0.19	1	0.18	1	0.18	1	0.20	1	0.25	1	8.91	1
0.21	1	0.20	1	0.26	1	0.39	1	0.44	1	0.25	1
0.20	1	0.20	1	0.22	1	0.21	1	0.31	1	0.35	1
0.49	1	0.24	1	5.31	1	0.07	1	0.27	1	0.27	1
4.41	1	0.22	1	0.24	1	0.23	1	0.20	1	0.23	1
0.24	1	0.40	1	0.21	1	3.53	1	0.27	1	0.28	1
0.14	1	2.80	1	0.22	1	0.22	1	5.45	1	2.43	1
0.26	1	0.45	1	0.37	1	0.24	1	0.21	1	0.76	1
0.20	1	0.20	1	0.22	1	0.33	1	0.47	1	0.27	1
0.20	1	0.20	1	0.22	1	0.26	1	0.51	1	0.26	1
0.17	1	0.19	1	0.31	1	0.23	1	0.23	1	2.79	1
0.21	1	0.19	1	0.23	1	0.25	1	0.19	1	0.21	1
0.34	1	0.20	1	0.22	1	0.09	1	0.17	1	0.38	1
0.22	1	0.18	1	0.22	1	0.10	1	0.18	1	0.25	1
0.20	1	0.17	1	0.29	1	0.21	1	0.18	1	0.25	1
0.20	1	8.42	1	0.22	1	0.21	1	0.18	1	0.28	1
0.43	1	0.24	1	0.24	1	0.36	1	0.19	1	0.39	1
0.59	1	0.33	1	0.18	1	0.63	1	0.19	1	2.67	1
0.24	1	0.19	1	0.42	1	20.34	1	2.88	1	0.27	1
0.18	1	0.18	1	0.24	1	0.78	1	0.22	1	0.28	1
0.19	1	0.19	1	0.22	1	5.28	1	0.17	1	0.25	1
0.20	1	0.14	1	2.30	1	0.20	1	0.19	1	0.26	1
0.20	1	0.18	1	NA	NA	0.26	1	0.21	1	0.26	1
0.20	1	0.17	1	NA	NA	0.33	1	8.85	1	0.25	1
0.18	1	0.18	1	NA	NA	0.18	1	0.20	1	0.26	1
0.20	1	0.19	1	NA	NA	0.19	1	0.18	1	0.28	1
0.19	1	0.16	1	NA	NA	0.27	1	0.27	1	1.80	1
0.21	1	0.19	1	NA	NA	0.31	1	0.29	1	0.25	1

time A	leuko A	time A	leuko A	time B	leuko B	time C	leuko C	time D	leuko D	time E	leuko E
0.19	1	NA	NA	NA	NA	0.17	1	0.37	1	0.27	1
0.22	1	NA	NA	NA	NA	0.27	1	0.21	1	0.27	1
2.76	1	NA	NA	NA	NA	0.19	1	0.24	1	5.86	1
0.24	1	NA	NA	NA	NA	0.21	1	0.33	1	0.41	1
0.19	1	NA	NA	NA	NA	0.17	1	0.20	1	0.27	1
0.22	1	NA	NA	NA	NA	0.22	1	0.29	1	0.22	1
0.20	1	NA	NA	NA	NA	0.06	1	0.29	1	NA	NA
0.19	1	NA	NA	NA	NA	8.33	1	0.18	1	NA	NA
0.18	1	NA	NA	NA	NA	0.26	1	0.39	1	NA	NA
0.20	1	NA	NA	NA	NA	NA	NA	0.18	1	NA	NA
0.20	1	NA	NA	NA	NA	NA	NA	0.29	1	NA	NA
0.20	1	NA	NA	NA	NA	NA	NA	0.54	1	NA	NA
0.18	1	NA	NA	NA	NA	NA	NA	0.20	1	NA	NA
NA	NA	NA	NA	NA	NA	NA	NA	0.23	1	NA	NA
NA	NA	NA	NA	NA	NA	NA	NA	1.46	1	NA	NA
NA	NA	NA	NA	NA	NA	NA	NA	0.22	1	NA	NA
NA	NA	NA	NA	NA	NA	NA	NA	0.22	1	NA	NA

1.6 OPT271

Table 6: Raw data of TBS OPT271.

time A	leuko A	time A	leuko A	time B	leuko B	time C	leuko C	time D	leuko D	time E	leuko E
0.00	1	0.00	1	0.00	0	0.00	1	0.00	1	0.00	1
0.26	1	0.25	1	0.34	0	0.20	1	0.24	1	0.28	1
0.32	1	5.69	1	3.97	0	0.42	1	0.23	1	0.25	1
0.20	1	0.27	1	1.84	1	0.92	1	2.58	1	0.90	1
0.17	1	0.24	1	0.28	1	7.02	1	0.18	1	0.28	1
0.19	1	0.21	1	1.24	1	0.09	1	0.40	1	0.26	1
0.19	1	8.53	1	2.12	1	4.88	1	0.19	1	0.91	0
0.18	1	2.32	1	0.28	1	0.21	1	0.47	1	0.31	0
0.17	1	3.30	1	0.26	1	2.37	1	0.35	1	0.74	0
0.20	1	2.57	1	15.04	1	0.21	1	0.28	1	2.44	0
0.15	1	0.24	1	0.30	1	2.61	1	0.41	1	9.97	1
0.23	1	0.22	1	0.97	1	1.38	1	0.21	1	0.25	1
0.22	1	0.19	1	0.25	1	0.03	1	0.14	1	5.02	0
6.52	1	0.18	1	0.61	1	0.13	1	0.14	1	7.39	1
9.01	1	1.50	1	6.98	1	0.20	1	6.65	0	0.26	1
6.18	1	2.58	1	0.27	1	0.53	1	0.57	0	0.23	1
0.23	1	0.25	1	0.46	1	0.09	1	1.30	0	0.25	1
3.36	1	0.21	1	0.26	1	2.28	1	4.56	0	0.27	1
0.87	1	0.20	1	0.53	1	0.06	1	0.39	0	0.27	1
4.88	1	0.19	1	0.25	1	0.13	1	0.69	0	0.76	1
0.25	1	0.18	1	0.25	1	0.22	1	0.76	0	0.75	0
0.28	1	0.19	1	0.24	1	0.19	1	0.63	0	3.77	0
0.20	1	0.23	1	0.24	1	0.08	1	2.09	0	4.80	0
4.35	1	1.53	1	0.73	0	0.11	1	0.95	0	2.45	0
0.25	1	0.22	1	7.65	1	0.18	1	0.62	0	5.19	1
3.73	1	0.18	1	0.35	1	0.22	1	25.39	1	0.23	1
0.23	1	0.17	1	0.27	1	0.17	1	0.21	1	6.10	0
0.38	1	0.18	1	0.67	1	0.19	1	0.33	1	7.37	1
0.40	1	2.55	1	71.36	0	0.20	1	0.19	1	0.26	1
6.01	1	0.21	1	0.53	0	0.20	1	0.28	1	0.25	1
0.27	1	0.19	1	1.04	1	0.36	1	0.19	1	0.28	1
0.17	1	0.17	1	0.33	1	0.60	1	0.15	1	0.58	1
3.58	1	0.18	1	1.50	1	6.22	1	0.16	1	0.29	1
0.20	1	1.88	1	0.29	1	0.22	1	0.24	1	0.26	1
0.20	1	4.11	1	0.23	1	0.21	1	0.17	1	0.32	1
0.20	1	0.24	1	0.19	1	0.32	1	0.17	1	0.24	1
0.20	1	0.27	1	0.54	1	0.26	1	0.16	1	3.31	0
0.21	1	0.78	1	0.30	1	0.22	1	0.22	1	10.44	1
0.21	1	1.73	0	0.50	1	0.21	1	0.28	1	0.26	1
0.17	1	4.30	1	17.19	0	0.20	1	0.22	1	0.23	1
0.20	1	0.25	1	0.45	0	2.30	1	0.35	1	0.25	1
0.21	1	0.20	1	0.37	0	0.21	1	0.23	1	0.26	1
0.21	1	0.19	1	1.03	0	0.20	1	0.19	1	0.23	1
0.21	1	0.19	1	2.10	1	0.20	1	0.47	1	0.24	1
0.30	1	0.19	1	0.29	1	0.08	1	2.86	0	0.23	1
4.15	1	0.20	1	0.41	1	0.31	1	0.86	0	8.50	1
0.23	1	7.54	1	0.55	1	0.08	1	1.11	0	0.29	1
0.21	1	0.24	1	0.30	1	0.11	1	2.68	0	0.26	1

time A	leuko A	time A	leuko A	time B	leuko B	time C	leuko C	time D	leuko D	time E	leuko E
0.20	1	0.22	1	0.26	1	0.10	1	1.89	0	0.27	1
0.19	1	2.15	1	9.17	1	1.66	1	1.40	0	2.40	0
0.18	1	0.23	1	0.28	1	0.21	1	3.24	0	6.37	0
0.46	0	0.21	1	0.25	1	0.19	1	4.50	1	8.15	1
4.50	0	0.23	1	1.27	1	0.23	1	0.33	1	0.22	1
0.36	0	0.20	1	0.34	1	0.24	1	0.35	1	0.33	1
1.58	1	0.17	1	0.28	1	2.01	1	0.25	1	0.25	1
0.21	1	7.41	1	0.25	1	0.19	1	0.27	1	3.92	0
0.19	1	0.25	1	0.29	1	0.22	1	0.25	1	0.52	0
0.20	1	0.22	1	6.24	0	0.19	1	0.26	1	0.71	0
0.19	1	0.21	1	1.08	0	0.22	1	0.45	1	1.66	0
0.22	1	0.19	1	1.18	0	0.05	1	0.23	1	1.86	0
0.18	1	7.89	1	1.35	0	0.11	1	0.25	1	5.99	1
0.15	1	0.22	1	17.90	0	3.05	1	0.48	1	0.34	1
0.18	1	0.21	1	0.51	0	0.08	1	0.25	1	0.32	1
0.29	0	0.21	1	0.57	0	0.12	1	0.29	1	0.37	1
0.70	0	0.21	1	0.52	0	0.07	1	1.19	0	0.74	1
0.70	0	0.20	1	2.22	0	0.16	1	0.30	0	0.26	1
5.26	1	0.19	1	0.51	0	0.22	1	0.34	0	0.23	1
0.24	1	8.73	1	2.15	0	0.22	1	0.61	0	0.25	1
1.22	0	0.22	1	1.24	1	0.21	1	1.01	0	0.27	1
4.26	1	0.20	1	0.30	1	0.04	1	0.99	0	6.78	0
0.22	1	0.18	1	0.22	1	0.12	1	2.01	0	2.40	0
5.85	1	4.84	1	0.40	1	0.19	1	1.53	0	3.07	0
0.22	1	0.21	1	0.43	1	0.20	1	0.40	0	6.16	1
0.21	1	0.21	1	0.67	1	0.19	1	1.08	0	0.31	1
0.26	1	0.20	1	14.74	0	0.06	1	1.65	0	0.28	1
0.20	1	0.19	1	0.46	0	0.12	1	1.95	0	6.65	0
0.19	1	0.21	1	1.09	0	0.07	1	3.20	1	1.44	0
0.52	0	0.21	1	0.87	0	0.14	1	0.28	1	5.61	1
0.24	0	3.28	1	2.76	1	0.21	1	0.71	1	0.27	1
6.47	1	0.22	1	0.36	1	0.21	1	0.23	1	0.25	1
0.28	1	0.21	1	0.30	1	0.23	1	0.22	1	0.95	1
0.25	1	0.22	1	0.35	1	0.18	1	0.37	1	0.26	1
0.25	1	0.26	1	0.29	1	1.45	1	0.21	1	9.06	0
0.36	0	0.22	1	0.64	1	0.21	1	0.30	1	1.71	0
0.31	0	0.19	1	0.31	1	0.22	1	0.64	1	3.68	0
2.09	1	0.31	1	0.23	1	0.19	1	0.24	1	4.29	1
0.24	1	7.55	1	25.79	0	0.22	1	0.24	1	0.28	1
0.19	1	0.24	1	0.41	0	0.19	1	4.22	0	6.56	0
0.19	1	0.21	1	0.28	0	0.22	1	0.32	0	3.09	0
0.19	1	0.19	1	5.78	0	0.34	1	0.33	0	8.06	1
0.18	1	0.20	1	0.34	0	0.24	1	0.60	0	0.30	1
0.18	1	0.19	1	0.27	0	0.35	1	3.47	0	0.28	1
1.85	0	0.20	1	7.94	1	0.18	1	0.60	0	0.27	1
2.91	1	0.20	1	0.36	1	0.20	1	0.65	0	0.27	1
0.22	1	0.20	1	0.29	1	2.19	1	0.94	0	0.30	1
0.22	1	0.23	1	0.25	1	0.18	1	1.63	0	0.27	1
0.19	1	0.22	1	4.93	1	0.25	1	1.46	0	0.24	1
0.18	1	6.26	1	0.33	1	2.86	1	1.05	0	1.85	0
0.18	1	0.24	1	0.25	1	0.20	1	0.98	0	7.75	0

time A	leuko A	time A	leuko A	time B	leuko B	time C	leuko C	time D	leuko D	time E	leuko E
0.17	1	0.24	1	0.25	1	0.22	1	2.47	0	76.23	1
0.18	1	0.24	1	0.58	1	0.23	1	7.27	1	0.29	1
0.30	1	0.24	1	0.24	1	0.19	1	0.23	1	0.26	1
0.19	1	5.33	1	1.25	1	0.26	1	0.98	1	3.91	0
4.37	1	0.28	1	0.30	1	1.56	1	0.23	1	10.71	1
0.25	1	0.20	1	6.75	1	0.36	1	0.29	1	0.25	1
0.20	1	0.22	1	0.35	1	2.51	1	0.52	1	0.37	1
3.20	1	0.18	1	0.29	1	4.38	1	0.22	1	0.26	1
0.22	1	0.18	1	0.81	1	0.32	1	0.28	1	0.26	1
0.19	1	5.55	1	0.40	1	0.19	1	0.20	1	0.27	1
0.20	1	1.10	1	0.36	1	0.21	1	0.29	1	0.25	1
0.21	1	1.83	1	0.32	1	0.07	1	0.23	1	0.24	1
0.16	1	0.22	1	0.41	1	0.10	1	0.70	1	44.41	1
0.18	1	0.21	1	4.95	1	0.22	1	0.23	1	0.34	1
3.92	0	1.81	1	0.39	1	0.21	1	10.70	0	0.22	1
2.19	1	36.86	1	0.29	1	0.18	1	1.03	0	0.29	1
0.18	1	1.13	0	0.29	1	0.19	1	2.12	0	0.23	1
0.19	1	3.77	0	0.60	1	0.07	1	0.82	0	0.25	1
0.17	1	22.73	1	0.50	1	0.16	1	5.27	0	0.22	1
0.15	1	0.20	1	0.43	1	1.74	1	1.61	0	3.25	0
0.19	1	0.43	1	0.34	1	2.45	1	6.92	1	1.84	0
0.34	1	1.33	0	3.73	0	0.22	1	0.48	1	15.19	1
0.22	1	2.36	1	0.42	0	0.08	1	0.19	1	0.22	1
0.21	1	0.27	1	5.38	1	0.11	1	0.18	1	0.28	1
0.15	1	0.17	1	0.35	1	0.23	1	0.40	1	0.24	1
6.26	1	0.20	1	0.30	1	0.20	1	0.17	1	13.43	1
0.24	1	0.20	1	1.41	1	0.20	1	0.20	1	0.21	1
0.19	1	0.41	1	0.35	1	0.20	1	0.35	1	0.29	1
0.17	1	1.19	0	0.96	0	0.19	1	0.43	1	2.82	0
4.27	1	5.87	1	0.45	0	0.20	1	0.19	1	2.05	0
0.21	1	0.24	1	11.41	0	0.20	1	0.29	1	8.99	1
0.19	1	0.21	1	3.61	1	1.81	1	0.36	1	0.30	1
0.17	1	0.18	1	0.39	1	0.22	1	0.18	1	15.97	1
0.17	1	0.15	1	0.28	1	0.20	1	0.23	1	8.43	1
0.15	1	0.17	1	0.42	1	0.40	1	0.37	1	0.25	1
0.15	1	0.19	1	0.30	1	0.23	1	2.12	0	2.27	0
0.18	1	0.20	1	0.26	1	1.40	1	0.61	0	12.60	1
0.18	1	6.97	1	3.97	0	1.99	1	0.27	0	0.24	1
2.44	1	0.27	1	10.66	1	0.18	1	0.62	0	0.80	1
0.24	1	0.19	1	0.33	1	0.21	1	0.53	0	7.41	1
0.20	1	0.16	1	3.40	1	0.08	1	1.72	0	0.27	1
0.18	1	0.27	1	0.29	1	0.10	1	1.23	0	8.17	1
0.19	1	3.66	1	0.23	1	0.08	1	0.61	0	0.28	1
0.26	1	0.21	1	0.69	1	0.13	1	0.89	0	0.24	1
0.17	1	0.19	1	0.24	1	1.57	1	3.89	0	4.80	0
0.19	1	0.22	1	0.23	1	0.20	1	0.53	0	12.74	1
0.19	1	0.36	1	0.35	1	0.21	1	0.34	0	0.23	1
0.17	1	0.17	1	4.23	0	0.20	1	0.76	0	0.27	1
0.17	1	0.22	1	0.38	0	0.26	1	0.41	0	0.11	1
6.52	1	6.93	1	5.59	1	0.32	1	0.25	0	0.23	1
0.32	1	0.17	1	0.31	1	0.21	1	5.52	1	25.63	1

time A	leuko A	time A	leuko A	time B	leuko B	time C	leuko C	time D	leuko D	time E	leuko E
0.26	1	0.24	1	0.43	1	0.20	1	0.24	1	0.33	1
0.21	1	0.20	1	0.25	1	0.20	1	0.22	1	0.32	1
0.17	1	1.76	0	0.32	1	0.20	1	0.26	1	6.17	1
0.29	0	0.22	0	22.23	1	0.18	1	0.22	1	0.26	1
0.61	0	10.77	1	0.35	1	0.08	1	0.37	1	NA	NA
2.54	1	0.01	1	0.61	1	0.09	1	0.20	1	NA	NA
1.62	1	0.20	1	0.27	1	0.36	1	0.21	1	NA	NA
2.89	1	0.21	1	0.24	1	2.54	1	0.43	1	NA	NA
0.21	1	8.67	1	0.23	1	0.21	1	0.44	1	NA	NA
0.18	1	10.34	1	3.76	0	0.22	1	0.22	1	NA	NA
0.20	1	0.18	1	0.60	0	0.08	1	0.23	1	NA	NA
0.20	1	0.25	1	0.88	1	0.12	1	0.42	1	NA	NA
1.75	1	NA	NA	0.37	1	0.21	1	0.39	1	NA	NA
0.21	1	NA	NA	0.38	1	0.42	1	0.34	1	NA	NA
0.19	1	NA	NA	4.90	0	0.20	1	0.24	1	NA	NA
3.29	1	NA	NA	1.25	1	0.19	1	0.17	1	NA	NA
0.21	1	NA	NA	0.30	1	0.21	1	0.17	1	NA	NA
0.20	1	NA	NA	1.18	0	1.13	1	0.38	0	NA	NA
NA	NA	NA	NA	3.50	1	0.10	1	5.87	0	NA	NA
NA	NA	NA	NA	0.34	1	0.10	1	1.43	0	NA	NA
NA	NA	NA	NA	0.28	1	0.20	1	1.02	0	NA	NA
NA	NA	NA	NA	0.25	1	0.08	1	0.78	0	NA	NA
NA	NA	NA	NA	0.64	1	0.14	1	4.70	0	NA	NA
NA	NA	NA	NA	0.34	1	0.90	1	0.67	0	NA	NA
NA	NA	NA	NA	9.71	1	0.24	1	1.74	0	NA	NA
NA	NA	NA	NA	0.32	1	0.20	1	0.37	0	NA	NA
NA	NA	NA	NA	0.56	1	0.67	1	0.50	0	NA	NA
NA	NA	NA	NA	0.30	1	0.22	1	5.44	1	NA	NA
NA	NA	NA	NA	4.59	0	0.21	1	0.22	1	NA	NA
NA	NA	NA	NA	0.35	0	0.23	1	0.32	1	NA	NA
NA	NA	NA	NA	0.64	0	0.70	1	0.20	1	NA	NA
NA	NA	NA	NA	0.79	1	0.76	1	0.26	1	NA	NA
NA	NA	NA	NA	0.29	1	0.23	1	0.22	1	NA	NA
NA	NA	NA	NA	0.70	1	0.22	1	0.22	1	NA	NA
NA	NA	NA	NA	0.37	1	0.21	1	0.23	1	NA	NA
NA	NA	NA	NA	0.27	1	0.21	1	0.21	1	NA	NA
NA	NA	NA	NA	0.24	1	0.21	1	0.23	1	NA	NA
NA	NA	NA	NA	0.25	1	0.88	1	0.29	1	NA	NA
NA	NA	NA	NA	0.26	1	0.23	1	0.22	1	NA	NA
NA	NA	NA	NA	5.23	1	3.48	1	0.39	1	NA	NA
NA	NA	NA	NA	0.32	1	0.25	1	0.22	1	NA	NA
NA	NA	NA	NA	0.27	1	1.55	1	0.30	1	NA	NA
NA	NA	NA	NA	0.44	1	0.23	1	0.40	1	NA	NA
NA	NA	NA	NA	0.29	1	0.97	1	0.22	1	NA	NA
NA	NA	NA	NA	3.24	0	0.24	1	0.21	1	NA	NA
NA	NA	NA	NA	10.99	0	0.22	1	0.20	1	NA	NA
NA	NA	NA	NA	2.70	1	3.36	1	0.17	1	NA	NA
NA	NA	NA	NA	0.26	1	1.51	1	0.21	1	NA	NA
NA	NA	NA	NA	0.22	1	1.44	1	0.54	1	NA	NA
NA	NA	NA	NA	0.22	1	0.21	1	0.30	0	NA	NA
NA	NA	NA	NA	0.24	1	0.07	1	0.25	0	NA	NA

time A	leuko A	time A	leuko A	time B	leuko B	time C	leuko C	time D	leuko D	time E	leuko E
NA	NA	NA	NA	0.67	1	0.26	1	0.21	0	NA	NA
NA	NA	NA	NA	0.27	1	1.38	1	0.22	0	NA	NA
NA	NA	NA	NA	0.24	1	2.01	1	2.02	0	NA	NA
NA	NA	NA	NA	13.25	1	0.22	1	0.55	0	NA	NA
NA	NA	NA	NA	0.33	1	2.59	1	0.29	0	NA	NA
NA	NA	NA	NA	0.31	1	0.06	1	0.66	0	NA	NA
NA	NA	NA	NA	0.30	1	5.30	1	2.01	0	NA	NA
NA	NA	NA	NA	6.94	0	5.19	1	0.61	0	NA	NA
NA	NA	NA	NA	1.70	0	11.78	1	0.92	0	NA	NA
NA	NA	NA	NA	11.03	1	0.22	1	1.14	0	NA	NA
NA	NA	NA	NA	0.32	1	0.08	1	1.06	0	NA	NA
NA	NA	NA	NA	0.25	1	0.14	1	1.01	0	NA	NA
NA	NA	NA	NA	0.22	1	0.07	1	1.56	0	NA	NA
NA	NA	NA	NA	4.09	1	0.23	1	0.45	0	NA	NA
NA	NA	NA	NA	0.28	1	0.22	1	0.83	0	NA	NA
NA	NA	NA	NA	5.88	1	1.31	1	0.42	0	NA	NA
NA	NA	NA	NA	0.38	1	0.21	1	15.93	1	NA	NA
NA	NA	NA	NA	0.28	1	0.21	1	0.24	1	NA	NA
NA	NA	NA	NA	3.85	1	0.24	1	0.31	1	NA	NA
NA	NA	NA	NA	0.36	1	0.22	1	0.49	1	NA	NA
NA	NA	NA	NA	0.30	1	0.19	1	0.22	1	NA	NA
NA	NA	NA	NA	0.27	1	0.42	1	0.20	1	NA	NA
NA	NA	NA	NA	0.91	0	1.38	1	0.33	1	NA	NA
NA	NA	NA	NA	3.52	1	0.21	1	0.22	1	NA	NA
NA	NA	NA	NA	0.32	1	0.21	1	0.21	1	NA	NA
NA	NA	NA	NA	2.15	1	0.21	1	0.27	1	NA	NA
NA	NA	NA	NA	0.78	1	0.07	1	0.22	1	NA	NA
NA	NA	NA	NA	4.80	0	2.05	1	0.30	1	NA	NA
NA	NA	NA	NA	1.17	1	0.21	1	0.93	0	NA	NA
NA	NA	NA	NA	0.32	1	0.20	1	1.04	0	NA	NA
NA	NA	NA	NA	0.26	1	0.18	1	1.67	0	NA	NA
NA	NA	NA	NA	0.27	1	0.23	1	1.05	0	NA	NA
NA	NA	NA	NA	0.25	1	0.26	1	0.43	0	NA	NA
NA	NA	NA	NA	0.27	1	0.19	1	1.81	0	NA	NA
NA	NA	NA	NA	0.20	1	0.20	1	1.18	0	NA	NA
NA	NA	NA	NA	9.57	1	0.21	1	0.49	0	NA	NA
NA	NA	NA	NA	0.41	1	4.25	0	0.92	0	NA	NA
NA	NA	NA	NA	3.23	0	3.40	0	5.16	1	NA	NA
NA	NA	NA	NA	0.67	0	2.14	1	0.24	1	NA	NA
NA	NA	NA	NA	0.73	0	0.21	1	0.18	1	NA	NA
NA	NA	NA	NA	0.28	0	0.20	1	0.24	1	NA	NA
NA	NA	NA	NA	5.38	0	0.19	1	0.30	1	NA	NA
NA	NA	NA	NA	3.38	1	0.21	1	0.88	0	NA	NA
NA	NA	NA	NA	0.40	1	0.29	1	0.74	0	NA	NA
NA	NA	NA	NA	0.31	1	0.27	1	0.65	0	NA	NA
NA	NA	NA	NA	0.27	1	0.31	1	1.36	0	NA	NA
NA	NA	NA	NA	6.43	1	0.08	1	1.23	0	NA	NA
NA	NA	NA	NA	0.39	1	0.10	1	0.34	0	NA	NA
NA	NA	NA	NA	0.29	1	0.21	1	1.61	0	NA	NA
NA	NA	NA	NA	0.27	1	0.20	1	1.68	0	NA	NA
NA	NA	NA	NA	3.13	1	0.22	1	2.48	0	NA	NA

time A	leuko A	time A	leuko A	time B	leuko B	time C	leuko C	time D	leuko D	time E	leuko E
NA	NA	NA	NA	0.36	1	0.19	1	9.08	1	NA	NA
NA	NA	NA	NA	1.86	0	0.20	1	0.23	1	NA	NA
NA	NA	NA	NA	0.38	0	0.21	1	0.23	1	NA	NA
NA	NA	NA	NA	0.32	0	0.22	1	0.79	1	NA	NA
NA	NA	NA	NA	4.41	1	0.08	1	0.34	1	NA	NA
NA	NA	NA	NA	0.34	1	0.13	1	0.27	1	NA	NA
NA	NA	NA	NA	0.28	1	0.61	1	0.23	1	NA	NA
NA	NA	NA	NA	1.39	0	0.21	1	0.26	1	NA	NA
NA	NA	NA	NA	0.51	0	0.21	1	0.31	1	NA	NA
NA	NA	NA	NA	1.80	0	0.54	1	1.17	0	NA	NA
NA	NA	NA	NA	0.45	0	1.07	0	0.42	0	NA	NA
NA	NA	NA	NA	7.18	1	2.97	1	0.67	0	NA	NA
NA	NA	NA	NA	0.59	1	0.27	1	2.15	0	NA	NA
NA	NA	NA	NA	0.82	1	0.21	1	0.38	0	NA	NA
NA	NA	NA	NA	NA	NA	0.47	1	0.47	0	NA	NA
NA	NA	NA	NA	NA	NA	0.18	1	0.84	0	NA	NA
NA	NA	NA	NA	NA	NA	2.93	0	1.31	0	NA	NA
NA	NA	NA	NA	NA	NA	3.06	1	0.68	0	NA	NA
NA	NA	NA	NA	NA	NA	0.21	1	0.55	0	NA	NA
NA	NA	NA	NA	NA	NA	0.27	1	0.95	0	NA	NA
NA	NA	NA	NA	NA	NA	0.20	1	0.32	0	NA	NA
NA	NA	NA	NA	NA	NA	0.20	1	0.77	0	NA	NA
NA	NA	NA	NA	NA	NA	0.39	1	0.29	0	NA	NA
NA	NA	NA	NA	NA	NA	0.19	1	3.60	1	NA	NA
NA	NA	NA	NA	NA	NA	3.61	1	0.47	1	NA	NA
NA	NA	NA	NA	NA	NA	0.01	0	0.22	1	NA	NA
NA	NA	NA	NA	NA	NA	0.32	1	0.34	1	NA	NA
NA	NA	NA	NA	NA	NA	0.85	1	0.23	1	NA	NA
NA	NA	NA	NA	NA	NA	0.21	1	0.37	1	NA	NA
NA	NA	NA	NA	NA	NA	0.27	1	0.22	1	NA	NA
NA	NA	NA	NA	NA	NA	2.10	1	0.26	1	NA	NA
NA	NA	NA	NA	NA	NA	0.23	1	0.35	1	NA	NA
NA	NA	NA	NA	NA	NA	0.45	1	0.23	1	NA	NA
NA	NA	NA	NA	NA	NA	8.29	1	0.16	1	NA	NA
NA	NA	NA	NA	NA	NA	2.74	1	0.33	1	NA	NA
NA	NA	NA	NA	NA	NA	0.23	1	0.21	1	NA	NA
NA	NA	NA	NA	NA	NA	15.59	1	0.25	1	NA	NA
NA	NA	NA	NA	NA	NA	0.10	1	0.18	1	NA	NA
NA	NA	NA	NA	NA	NA	1.55	1	1.82	0	NA	NA
NA	NA	NA	NA	NA	NA	6.43	1	0.26	0	NA	NA
NA	NA	NA	NA	NA	NA	2.14	1	0.51	0	NA	NA
NA	NA	NA	NA	NA	NA	0.27	1	1.26	0	NA	NA
NA	NA	NA	NA	NA	NA	0.27	1	0.49	0	NA	NA
NA	NA	NA	NA	NA	NA	0.10	1	0.53	0	NA	NA
NA	NA	NA	NA	NA	NA	2.37	1	0.29	0	NA	NA
NA	NA	NA	NA	NA	NA	0.22	1	0.24	0	NA	NA
NA	NA	NA	NA	NA	NA	0.28	1	0.50	0	NA	NA
NA	NA	NA	NA	NA	NA	0.45	1	0.32	0	NA	NA
NA	NA	NA	NA	NA	NA	0.01	1	1.03	0	NA	NA
NA	NA	NA	NA	NA	NA	0.05	1	0.40	0	NA	NA
NA	NA	NA	NA	NA	NA	0.16	1	0.67	0	NA	NA

time A	leuko A	time A	leuko A	time B	leuko B	time C	leuko C	time D	leuko D	time E	leuko E
NA	NA	NA	NA	NA	NA	0.45	1	0.37	0	NA	NA
NA	NA	NA	NA	NA	NA	2.54	1	0.33	0	NA	NA
NA	NA	NA	NA	NA	NA	0.24	1	0.32	0	NA	NA
NA	NA	NA	NA	NA	NA	0.41	1	0.46	0	NA	NA
NA	NA	NA	NA	NA	NA	1.74	1	0.33	0	NA	NA
NA	NA	NA	NA	NA	NA	0.24	1	4.59	1	NA	NA
NA	NA	NA	NA	NA	NA	0.29	1	0.21	1	NA	NA
NA	NA	NA	NA	NA	NA	0.28	1	0.20	1	NA	NA
NA	NA	NA	NA	NA	NA	42.80	1	0.31	1	NA	NA
NA	NA	NA	NA	NA	NA	0.27	1	0.26	1	NA	NA
NA	NA	NA	NA	NA	NA	0.48	1	0.23	1	NA	NA
NA	NA	NA	NA	NA	NA	0.09	1	0.18	1	NA	NA
NA	NA	NA	NA	NA	NA	0.10	1	0.22	1	NA	NA
NA	NA	NA	NA	NA	NA	0.76	1	0.51	1	NA	NA
NA	NA	NA	NA	NA	NA	0.11	1	0.22	1	NA	NA
NA	NA	NA	NA	NA	NA	0.11	1	0.20	1	NA	NA
NA	NA	NA	NA	NA	NA	2.83	1	0.16	1	NA	NA
NA	NA	NA	NA	NA	NA	28.98	1	0.38	0	NA	NA
NA	NA	NA	NA	NA	NA	0.30	1	0.23	0	NA	NA
NA	NA	NA	NA	NA	NA	0.76	1	0.52	0	NA	NA
NA	NA	NA	NA	NA	NA	0.08	1	1.86	0	NA	NA
NA	NA	NA	NA	NA	NA	0.11	1	1.60	0	NA	NA
NA	NA	NA	NA	NA	NA	0.21	1	0.75	0	NA	NA
NA	NA	NA	NA	NA	NA	0.09	1	1.78	0	NA	NA
NA	NA	NA	NA	NA	NA	0.11	1	0.63	0	NA	NA
NA	NA	NA	NA	NA	NA	0.21	1	14.34	1	NA	NA
NA	NA	NA	NA	NA	NA	0.20	1	0.21	1	NA	NA
NA	NA	NA	NA	NA	NA	0.22	1	0.18	1	NA	NA
NA	NA	NA	NA	NA	NA	0.20	1	0.24	1	NA	NA
NA	NA	NA	NA	NA	NA	0.35	1	0.20	1	NA	NA
NA	NA	NA	NA	NA	NA	0.28	1	0.19	1	NA	NA
NA	NA	NA	NA	NA	NA	0.24	1	0.23	1	NA	NA
NA	NA	NA	NA	NA	NA	0.25	1	0.20	1	NA	NA
NA	NA	NA	NA	NA	NA	0.10	1	0.18	1	NA	NA
NA	NA	NA	NA	NA	NA	0.11	1	0.31	1	NA	NA
NA	NA	NA	NA	NA	NA	0.08	1	0.20	1	NA	NA
NA	NA	NA	NA	NA	NA	0.11	1	0.18	1	NA	NA
NA	NA	NA	NA	NA	NA	0.22	1	0.26	1	NA	NA
NA	NA	NA	NA	NA	NA	0.29	1	4.48	0	NA	NA
NA	NA	NA	NA	NA	NA	0.29	1	0.97	0	NA	NA
NA	NA	NA	NA	NA	NA	0.38	1	0.52	0	NA	NA
NA	NA	NA	NA	NA	NA	0.29	1	2.54	0	NA	NA
NA	NA	NA	NA	NA	NA	0.26	1	0.85	0	NA	NA
NA	NA	NA	NA	NA	NA	0.24	1	1.43	0	NA	NA
NA	NA	NA	NA	NA	NA	0.29	1	0.51	0	NA	NA
NA	NA	NA	NA	NA	NA	0.25	1	0.50	0	NA	NA
NA	NA	NA	NA	NA	NA	0.23	1	0.55	0	NA	NA
NA	NA	NA	NA	NA	NA	0.20	1	0.32	0	NA	NA
NA	NA	NA	NA	NA	NA	0.88	1	3.67	1	NA	NA
NA	NA	NA	NA	NA	NA	0.45	1	0.36	1	NA	NA
NA	NA	NA	NA	NA	NA	0.24	1	2.69	1	NA	NA

time A	leuko A	time A	leuko A	time B	leuko B	time C	leuko C	time D	leuko D	time E	leuko E
NA	NA	NA	NA	NA	NA	0.32	1	0.37	1	NA	NA
NA	NA	NA	NA	NA	NA	0.27	1	0.30	1	NA	NA
NA	NA	NA	NA	NA	NA	0.37	1	0.41	1	NA	NA
NA	NA	NA	NA	NA	NA	1.49	1	0.36	1	NA	NA
NA	NA	NA	NA	NA	NA	0.23	1	0.40	1	NA	NA
NA	NA	NA	NA	NA	NA	0.26	1	0.24	1	NA	NA
NA	NA	NA	NA	NA	NA	0.19	1	0.21	1	NA	NA
NA	NA	NA	NA	NA	NA	0.22	1	0.76	0	NA	NA
NA	NA	NA	NA	NA	NA	0.21	1	0.82	0	NA	NA
NA	NA	NA	NA	NA	NA	0.36	1	0.43	0	NA	NA
NA	NA	NA	NA	NA	NA	0.19	1	1.15	0	NA	NA
NA	NA	NA	NA	NA	NA	0.23	1	0.36	0	NA	NA
NA	NA	NA	NA	NA	NA	0.50	1	0.80	0	NA	NA
NA	NA	NA	NA	NA	NA	0.20	1	0.31	0	NA	NA
NA	NA	NA	NA	NA	NA	0.20	1	0.33	0	NA	NA
NA	NA	NA	NA	NA	NA	0.21	1	0.38	0	NA	NA
NA	NA	NA	NA	NA	NA	0.23	1	0.30	0	NA	NA
NA	NA	NA	NA	NA	NA	0.20	1	1.30	0	NA	NA
NA	NA	NA	NA	NA	NA	0.27	1	NA	NA	NA	NA
NA	NA	NA	NA	NA	NA	0.25	1	NA	NA	NA	NA
NA	NA	NA	NA	NA	NA	0.22	1	NA	NA	NA	NA
NA	NA	NA	NA	NA	NA	0.25	1	NA	NA	NA	NA
NA	NA	NA	NA	NA	NA	0.26	1	NA	NA	NA	NA
NA	NA	NA	NA	NA	NA	0.25	1	NA	NA	NA	NA
NA	NA	NA	NA	NA	NA	0.27	1	NA	NA	NA	NA
NA	NA	NA	NA	NA	NA	0.37	1	NA	NA	NA	NA
NA	NA	NA	NA	NA	NA	0.26	1	NA	NA	NA	NA
NA	NA	NA	NA	NA	NA	0.27	1	NA	NA	NA	NA
NA	NA	NA	NA	NA	NA	0.08	1	NA	NA	NA	NA
NA	NA	NA	NA	NA	NA	0.15	1	NA	NA	NA	NA
NA	NA	NA	NA	NA	NA	0.27	1	NA	NA	NA	NA
NA	NA	NA	NA	NA	NA	0.08	1	NA	NA	NA	NA
NA	NA	NA	NA	NA	NA	0.20	1	NA	NA	NA	NA
NA	NA	NA	NA	NA	NA	0.27	1	NA	NA	NA	NA
NA	NA	NA	NA	NA	NA	0.08	1	NA	NA	NA	NA
NA	NA	NA	NA	NA	NA	0.17	1	NA	NA	NA	NA
NA	NA	NA	NA	NA	NA	0.26	1	NA	NA	NA	NA
NA	NA	NA	NA	NA	NA	0.26	1	NA	NA	NA	NA
NA	NA	NA	NA	NA	NA	0.25	1	NA	NA	NA	NA
NA	NA	NA	NA	NA	NA	0.25	1	NA	NA	NA	NA
NA	NA	NA	NA	NA	NA	0.25	1	NA	NA	NA	NA
NA	NA	NA	NA	NA	NA	0.89	1	NA	NA	NA	NA
NA	NA	NA	NA	NA	NA	0.12	1	NA	NA	NA	NA
NA	NA	NA	NA	NA	NA	0.16	1	NA	NA	NA	NA
NA	NA	NA	NA	NA	NA	0.24	1	NA	NA	NA	NA
NA	NA	NA	NA	NA	NA	0.23	1	NA	NA	NA	NA
NA	NA	NA	NA	NA	NA	0.23	1	NA	NA	NA	NA
NA	NA	NA	NA	NA	NA	0.47	1	NA	NA	NA	NA
NA	NA	NA	NA	NA	NA	0.21	1	NA	NA	NA	NA
NA	NA	NA	NA	NA	NA	0.22	1	NA	NA	NA	NA
NA	NA	NA	NA	NA	NA	0.25	1	NA	NA	NA	NA

time A	leuko A	time A	leuko A	time B	leuko B	time C	leuko C	time D	leuko D	time E	leuko E
NA	NA	NA	NA	NA	NA	0.24	1	NA	NA	NA	NA
NA	NA	NA	NA	NA	NA	0.31	1	NA	NA	NA	NA
NA	NA	NA	NA	NA	NA	0.26	1	NA	NA	NA	NA
NA	NA	NA	NA	NA	NA	0.22	1	NA	NA	NA	NA
NA	NA	NA	NA	NA	NA	0.20	1	NA	NA	NA	NA
NA	NA	NA	NA	NA	NA	0.23	1	NA	NA	NA	NA
NA	NA	NA	NA	NA	NA	0.25	1	NA	NA	NA	NA
NA	NA	NA	NA	NA	NA	0.27	1	NA	NA	NA	NA
NA	NA	NA	NA	NA	NA	0.24	1	NA	NA	NA	NA
NA	NA	NA	NA	NA	NA	0.33	1	NA	NA	NA	NA
NA	NA	NA	NA	NA	NA	0.39	1	NA	NA	NA	NA
NA	NA	NA	NA	NA	NA	0.79	1	NA	NA	NA	NA
NA	NA	NA	NA	NA	NA	0.22	1	NA	NA	NA	NA
NA	NA	NA	NA	NA	NA	0.24	1	NA	NA	NA	NA
NA	NA	NA	NA	NA	NA	0.25	1	NA	NA	NA	NA
NA	NA	NA	NA	NA	NA	0.62	1	NA	NA	NA	NA
NA	NA	NA	NA	NA	NA	0.65	1	NA	NA	NA	NA
NA	NA	NA	NA	NA	NA	0.50	1	NA	NA	NA	NA
NA	NA	NA	NA	NA	NA	0.32	1	NA	NA	NA	NA
NA	NA	NA	NA	NA	NA	0.24	1	NA	NA	NA	NA
NA	NA	NA	NA	NA	NA	1.85	1	NA	NA	NA	NA
NA	NA	NA	NA	NA	NA	1.00	1	NA	NA	NA	NA
NA	NA	NA	NA	NA	NA	0.16	1	NA	NA	NA	NA
NA	NA	NA	NA	NA	NA	0.21	1	NA	NA	NA	NA
NA	NA	NA	NA	NA	NA	1.67	1	NA	NA	NA	NA
NA	NA	NA	NA	NA	NA	0.23	1	NA	NA	NA	NA
NA	NA	NA	NA	NA	NA	0.22	1	NA	NA	NA	NA
NA	NA	NA	NA	NA	NA	0.20	1	NA	NA	NA	NA
NA	NA	NA	NA	NA	NA	0.19	1	NA	NA	NA	NA
NA	NA	NA	NA	NA	NA	0.20	1	NA	NA	NA	NA
NA	NA	NA	NA	NA	NA	0.21	1	NA	NA	NA	NA
NA	NA	NA	NA	NA	NA	0.18	1	NA	NA	NA	NA
NA	NA	NA	NA	NA	NA	0.20	1	NA	NA	NA	NA
NA	NA	NA	NA	NA	NA	0.20	1	NA	NA	NA	NA
NA	NA	NA	NA	NA	NA	0.24	1	NA	NA	NA	NA
NA	NA	NA	NA	NA	NA	0.25	1	NA	NA	NA	NA
NA	NA	NA	NA	NA	NA	0.22	1	NA	NA	NA	NA
NA	NA	NA	NA	NA	NA	0.23	1	NA	NA	NA	NA
NA	NA	NA	NA	NA	NA	0.48	1	NA	NA	NA	NA
NA	NA	NA	NA	NA	NA	0.23	1	NA	NA	NA	NA
NA	NA	NA	NA	NA	NA	0.26	1	NA	NA	NA	NA
NA	NA	NA	NA	NA	NA	0.33	1	NA	NA	NA	NA
NA	NA	NA	NA	NA	NA	0.46	1	NA	NA	NA	NA
NA	NA	NA	NA	NA	NA	0.26	1	NA	NA	NA	NA
NA	NA	NA	NA	NA	NA	0.22	1	NA	NA	NA	NA
NA	NA	NA	NA	NA	NA	0.24	1	NA	NA	NA	NA
NA	NA	NA	NA	NA	NA	0.22	1	NA	NA	NA	NA
NA	NA	NA	NA	NA	NA	0.23	1	NA	NA	NA	NA
NA	NA	NA	NA	NA	NA	0.21	1	NA	NA	NA	NA
NA	NA	NA	NA	NA	NA	0.21	1	NA	NA	NA	NA
NA	NA	NA	NA	NA	NA	0.84	1	NA	NA	NA	NA

time A	leuko A	time A	leuko A	time B	leuko B	time C	leuko C	time D	leuko D	time E	leuko E
NA	NA	NA	NA	NA	NA	0.28	1	NA	NA	NA	NA
NA	NA	NA	NA	NA	NA	0.11	1	NA	NA	NA	NA
NA	NA	NA	NA	NA	NA	0.10	1	NA	NA	NA	NA
NA	NA	NA	NA	NA	NA	0.22	1	NA	NA	NA	NA
NA	NA	NA	NA	NA	NA	0.09	1	NA	NA	NA	NA
NA	NA	NA	NA	NA	NA	0.12	1	NA	NA	NA	NA
NA	NA	NA	NA	NA	NA	0.20	1	NA	NA	NA	NA
NA	NA	NA	NA	NA	NA	0.07	1	NA	NA	NA	NA
NA	NA	NA	NA	NA	NA	0.23	1	NA	NA	NA	NA
NA	NA	NA	NA	NA	NA	0.22	1	NA	NA	NA	NA
NA	NA	NA	NA	NA	NA	0.27	1	NA	NA	NA	NA
NA	NA	NA	NA	NA	NA	0.21	1	NA	NA	NA	NA
NA	NA	NA	NA	NA	NA	0.20	1	NA	NA	NA	NA
NA	NA	NA	NA	NA	NA	0.21	1	NA	NA	NA	NA
NA	NA	NA	NA	NA	NA	0.22	1	NA	NA	NA	NA
NA	NA	NA	NA	NA	NA	0.22	1	NA	NA	NA	NA
NA	NA	NA	NA	NA	NA	0.18	1	NA	NA	NA	NA
NA	NA	NA	NA	NA	NA	0.22	1	NA	NA	NA	NA
NA	NA	NA	NA	NA	NA	0.22	1	NA	NA	NA	NA
NA	NA	NA	NA	NA	NA	0.21	1	NA	NA	NA	NA
NA	NA	NA	NA	NA	NA	0.22	1	NA	NA	NA	NA
NA	NA	NA	NA	NA	NA	0.21	1	NA	NA	NA	NA
NA	NA	NA	NA	NA	NA	0.23	1	NA	NA	NA	NA
NA	NA	NA	NA	NA	NA	0.23	1	NA	NA	NA	NA
NA	NA	NA	NA	NA	NA	0.22	1	NA	NA	NA	NA
NA	NA	NA	NA	NA	NA	0.21	1	NA	NA	NA	NA
NA	NA	NA	NA	NA	NA	0.23	1	NA	NA	NA	NA
NA	NA	NA	NA	NA	NA	0.23	1	NA	NA	NA	NA
NA	NA	NA	NA	NA	NA	0.23	1	NA	NA	NA	NA
NA	NA	NA	NA	NA	NA	0.23	1	NA	NA	NA	NA
NA	NA	NA	NA	NA	NA	0.25	1	NA	NA	NA	NA
NA	NA	NA	NA	NA	NA	0.27	1	NA	NA	NA	NA
NA	NA	NA	NA	NA	NA	0.26	1	NA	NA	NA	NA
NA	NA	NA	NA	NA	NA	0.25	1	NA	NA	NA	NA
NA	NA	NA	NA	NA	NA	0.61	1	NA	NA	NA	NA
NA	NA	NA	NA	NA	NA	0.18	1	NA	NA	NA	NA
NA	NA	NA	NA	NA	NA	0.23	1	NA	NA	NA	NA
NA	NA	NA	NA	NA	NA	0.21	1	NA	NA	NA	NA
NA	NA	NA	NA	NA	NA	0.23	1	NA	NA	NA	NA
NA	NA	NA	NA	NA	NA	0.21	1	NA	NA	NA	NA
NA	NA	NA	NA	NA	NA	0.23	1	NA	NA	NA	NA
NA	NA	NA	NA	NA	NA	0.21	1	NA	NA	NA	NA
NA	NA	NA	NA	NA	NA	0.24	1	NA	NA	NA	NA
NA	NA	NA	NA	NA	NA	0.20	1	NA	NA	NA	NA
NA	NA	NA	NA	NA	NA	0.22	1	NA	NA	NA	NA
NA	NA	NA	NA	NA	NA	0.22	1	NA	NA	NA	NA
NA	NA	NA	NA	NA	NA	0.10	1	NA	NA	NA	NA
NA	NA	NA	NA	NA	NA	0.12	0	NA	NA	NA	NA
NA	NA	NA	NA	NA	NA	0.24	0	NA	NA	NA	NA
NA	NA	NA	NA	NA	NA	0.17	1	NA	NA	NA	NA
NA	NA	NA	NA	NA	NA	0.25	1	NA	NA	NA	NA

time A	leuko A	time A	leuko A	time B	leuko B	time C	leuko C	time D	leuko D	time E	leuko E
NA	NA	NA	NA	NA	NA	0.19	1	NA	NA	NA	NA

1.7 SL:13

Table 7: Raw data of TBS SL:13.

time A	leuko A	time A	leuko A	time B	leuko B	time C	leuko C	time D	leuko D	time E	leuko E
0.00	1	0.00	1	0.00	1	0.00	1	0.00	1	0.00	1
0.69	1	0.25	1	0.35	1	0.22	1	0.22	1	0.30	1
0.22	1	0.22	1	0.27	1	0.22	1	0.22	1	2.44	0
0.08	1	0.22	1	0.60	1	0.23	1	0.41	1	0.65	0
0.09	1	0.21	1	0.28	1	0.21	1	0.24	1	0.31	0
0.19	1	0.23	1	0.24	1	0.22	1	0.31	1	5.13	0
0.19	1	0.19	1	0.36	1	0.20	1	1.44	1	1.19	0
0.18	1	1.29	0	0.23	1	0.44	1	0.39	1	1.18	0
0.17	1	0.27	0	6.09	0	0.01	1	0.19	1	0.31	0
1.04	0	0.26	0	1.76	0	0.18	1	0.30	1	0.84	0
0.45	0	0.59	0	0.33	0	0.19	1	0.23	1	0.31	0
1.77	0	0.25	0	0.43	0	0.07	1	0.23	1	0.21	0
1.37	0	0.30	0	0.75	0	2.70	0	0.28	1	0.48	0
1.99	0	3.78	1	9.33	0	0.26	0	0.17	1	1.66	0
1.16	0	0.24	1	0.38	0	1.62	0	2.04	0	0.35	0
2.04	1	0.16	1	0.29	0	0.96	0	0.35	0	0.38	0
0.15	1	0.16	1	1.74	1	1.72	0	3.31	0	0.87	0
0.12	1	0.19	1	0.27	1	0.26	0	0.59	0	11.42	1
0.23	1	0.32	1	0.38	1	0.58	0	6.59	0	0.28	1
0.21	1	0.18	1	0.22	1	0.36	0	0.89	0	0.08	1
0.16	1	0.21	1	9.54	0	0.71	0	7.43	1	0.14	1
0.16	1	0.35	1	0.34	0	0.41	0	0.21	1	0.26	1
0.08	1	0.70	0	0.41	0	0.48	0	0.20	1	0.46	1
0.10	1	0.25	0	0.61	0	0.37	0	0.21	1	4.49	0
0.17	1	0.25	0	1.02	1	0.53	0	0.18	1	0.25	0
1.00	0	0.27	0	0.23	1	0.53	0	0.22	1	0.58	0
0.24	0	0.21	0	0.24	1	1.04	0	0.17	1	0.24	0
0.20	0	3.14	1	0.69	1	0.84	0	0.20	1	0.53	0
1.41	0	0.23	1	0.26	1	0.37	0	0.19	1	0.23	0
0.29	0	0.20	1	0.22	1	1.68	0	0.18	1	0.25	0
0.26	0	0.18	1	0.21	1	0.27	0	0.41	1	1.96	0
0.56	0	0.29	1	0.57	0	0.59	0	0.58	1	0.61	0
0.24	0	0.15	1	0.98	0	0.25	0	0.21	1	0.74	0
0.20	0	0.16	1	0.52	0	0.27	0	0.22	1	0.28	0
0.24	0	0.19	1	0.62	0	0.32	0	0.18	1	0.26	0
3.49	1	0.37	0	0.27	0	0.47	0	4.55	0	0.28	0
0.24	1	0.25	0	7.57	1	4.58	1	5.10	0	1.50	0
0.37	1	0.20	0	0.29	1	0.23	1	1.91	0	0.30	0
0.23	1	0.22	0	0.24	1	0.22	1	0.78	0	0.77	0
0.20	1	0.25	0	0.21	1	0.02	1	1.77	0	1.42	0
0.21	1	0.21	0	2.17	0	0.19	1	4.11	0	1.56	0
9.37	1	0.40	0	0.40	0	0.35	1	3.72	0	1.02	0
0.25	1	0.24	0	1.34	0	0.28	1	3.37	0	0.30	0
0.17	1	0.18	0	0.67	0	2.05	0	9.79	1	0.86	0
0.17	1	1.81	1	1.28	0	0.29	0	0.24	1	11.41	1
0.21	1	2.60	0	1.33	0	1.65	0	0.22	1	0.25	1
0.17	1	0.20	0	0.33	0	0.61	0	0.17	1	0.31	1
0.21	1	0.20	0	10.82	0	0.25	0	0.20	1	8.29	0

time A	leuko A	time A	leuko A	time B	leuko B	time C	leuko C	time D	leuko D	time E	leuko E
2.03	0	0.21	0	7.41	1	1.52	0	0.20	1	2.86	0
0.27	0	0.19	0	0.29	1	0.29	0	0.24	1	2.20	0
0.22	0	0.19	0	0.31	1	1.98	0	0.17	1	0.29	0
0.20	0	0.30	0	0.45	1	0.34	0	0.27	1	0.53	0
0.29	0	1.54	1	0.25	1	0.83	0	0.23	1	0.69	0
0.30	0	0.23	1	0.30	1	1.14	0	0.94	1	1.37	0
0.21	0	0.20	1	8.90	0	0.46	0	0.23	1	0.48	0
0.19	0	0.16	1	0.47	0	0.39	0	0.20	1	0.29	0
0.17	0	0.18	1	0.92	0	0.44	0	3.07	0	0.25	0
0.34	0	0.18	1	0.93	0	3.63	1	0.75	0	6.15	1
0.95	0	0.18	1	0.42	0	0.22	1	2.38	0	0.24	1
0.90	0	0.40	0	1.15	0	0.23	1	1.49	0	0.30	1
2.05	0	0.19	0	0.85	0	0.26	1	2.04	0	3.15	0
0.24	0	0.19	0	0.56	0	0.19	1	5.09	0	0.79	0
0.21	0	0.22	0	0.84	0	0.23	1	2.15	0	0.28	0
7.23	1	0.21	0	1.96	1	0.07	1	3.73	0	2.59	0
0.31	1	0.18	0	0.30	1	0.12	1	14.71	1	0.30	0
0.20	1	0.23	0	0.26	1	0.07	1	0.45	1	0.23	0
0.21	1	0.18	0	0.23	1	7.65	0	0.64	1	0.60	0
0.20	1	0.14	0	9.10	0	1.24	0	0.67	1	0.26	0
0.24	1	0.26	0	0.44	0	1.19	0	0.44	1	1.40	0
0.21	1	0.28	0	0.96	0	4.51	1	0.26	1	0.29	0
0.28	1	0.24	0	0.36	0	0.23	1	0.21	1	0.45	0
2.83	0	0.22	0	0.49	0	0.24	1	0.19	1	0.88	0
0.24	0	0.32	0	0.69	0	0.44	1	0.23	1	0.30	0
0.19	0	0.19	0	0.37	0	0.18	1	0.21	1	0.75	0
0.25	0	0.20	0	0.51	0	0.21	1	0.70	1	0.93	0
1.33	0	11.19	1	0.65	0	0.01	1	0.22	1	1.72	0
0.85	0	0.30	1	0.36	0	0.18	1	0.20	1	1.25	0
4.20	1	0.27	1	4.41	1	2.83	0	4.13	0	0.60	0
0.26	1	2.91	0	0.27	1	0.69	0	0.22	0	0.91	0
0.10	1	0.24	0	0.23	1	0.91	0	1.14	0	0.30	0
0.10	1	0.21	0	0.44	1	0.42	0	6.33	0	6.99	1
0.20	1	0.25	0	0.27	1	0.42	0	0.44	0	0.26	1
0.18	1	0.47	0	0.21	1	1.14	0	2.43	0	0.25	1
0.17	1	0.26	0	7.40	0	0.71	0	6.68	0	3.91	0
0.18	1	0.20	0	0.43	0	3.77	0	1.48	0	3.32	0
0.66	0	0.20	0	0.42	0	0.51	0	1.20	0	1.61	0
0.22	0	0.22	0	0.66	0	7.57	1	21.86	1	1.49	0
0.21	0	0.37	0	0.43	0	0.24	1	0.19	1	0.26	0
0.19	0	0.23	0	0.37	0	0.01	1	0.17	1	0.78	0
0.44	0	0.19	0	0.37	0	0.27	1	0.19	1	0.45	0
1.86	0	0.26	0	1.84	0	0.23	1	0.15	1	0.53	0
5.86	1	0.31	0	0.50	0	0.27	1	0.36	1	0.26	0
0.23	1	0.22	0	0.35	0	0.68	1	0.19	1	0.25	0
0.23	1	5.78	1	0.32	0	2.53	0	0.18	1	1.97	0
0.19	1	0.26	1	0.98	0	1.15	0	0.19	1	0.58	0
0.22	1	0.19	1	0.72	0	0.63	0	0.18	1	0.76	0
2.93	0	0.18	1	0.47	0	1.79	0	0.24	1	0.72	0
0.28	0	0.77	0	1.27	1	2.78	0	0.21	1	1.89	0
0.21	0	0.28	0	0.25	1	1.58	0	0.31	1	0.33	0

time A	leuko A	time A	leuko A	time B	leuko B	time C	leuko C	time D	leuko D	time E	leuko E
0.22	0	0.28	0	0.51	1	5.30	1	0.27	1	0.22	0
0.21	0	0.23	0	0.24	1	0.23	1	0.31	1	0.23	0
0.20	0	0.22	0	0.46	1	0.23	1	0.48	1	0.98	0
0.20	0	0.31	0	0.40	1	0.61	1	0.32	1	0.31	0
0.19	0	4.51	1	0.29	1	1.60	0	0.22	1	0.30	0
0.20	0	0.23	1	0.26	1	0.30	0	1.20	0	0.77	0
0.18	0	0.21	1	14.17	0	0.26	0	0.26	0	0.28	0
0.22	0	0.26	1	0.48	0	3.13	0	0.45	0	0.45	0
3.93	1	0.22	1	0.59	0	0.54	0	1.07	0	1.62	0
0.13	1	1.65	0	0.41	0	0.87	0	0.24	0	8.96	1
0.11	1	0.26	0	0.55	0	7.04	1	1.42	0	0.24	1
0.21	1	0.23	0	0.57	0	0.22	1	0.96	0	0.31	1
0.21	1	0.29	0	0.41	0	0.34	1	0.26	0	0.27	1
0.20	1	0.19	0	0.86	0	0.31	1	0.97	0	0.25	1
0.19	1	0.21	0	0.37	0	0.24	1	2.78	0	0.25	1
2.64	0	0.21	0	0.29	0	0.22	1	0.49	0	0.24	1
0.25	0	0.26	0	0.54	0	0.06	1	0.68	0	0.24	1
0.45	0	0.23	0	0.59	0	0.24	1	1.74	0	4.59	0
0.50	0	0.41	0	5.28	0	0.06	1	0.63	0	0.27	0
0.68	0	1.89	1	0.43	0	0.13	1	0.29	0	0.23	0
0.28	0	0.26	1	0.29	0	0.07	1	0.37	0	2.92	0
0.23	0	0.22	1	0.23	0	0.17	1	0.33	0	0.94	0
0.21	0	0.42	0	0.93	1	0.21	1	1.54	0	2.72	0
0.18	0	0.24	0	0.29	1	0.21	1	0.46	0	0.35	0
0.21	0	0.29	0	0.24	1	0.24	1	0.58	0	0.71	0
0.82	0	0.20	0	0.35	1	0.32	1	9.31	1	0.70	0
7.29	1	0.20	0	0.25	1	1.86	0	0.21	1	0.78	0
0.38	1	0.20	0	0.31	1	1.14	0	0.23	1	1.36	0
0.24	1	3.60	1	0.23	1	0.56	0	0.24	1	0.57	0
0.23	1	0.22	1	0.23	1	1.95	0	0.31	1	0.25	0
0.21	1	0.16	1	0.22	1	0.29	0	0.19	1	1.05	0
0.22	1	0.17	1	7.39	1	0.62	0	0.21	1	0.28	0
0.18	1	0.18	1	0.28	1	0.40	0	0.19	1	0.23	0
0.20	1	0.18	1	0.47	1	0.31	0	0.18	1	7.12	1
0.18	1	0.17	1	0.24	1	0.84	0	0.16	1	0.27	1
0.20	1	0.15	1	0.21	1	0.29	0	0.18	1	0.28	1
0.20	1	0.17	1	0.21	1	1.75	0	0.20	1	0.28	1
6.90	0	0.14	1	0.28	1	1.18	0	0.17	1	3.87	0
0.26	0	0.42	0	0.22	1	1.83	0	0.18	1	0.26	0
0.17	0	0.27	0	0.28	1	0.34	0	3.64	0	0.22	0
0.15	0	0.26	0	0.22	1	1.07	0	3.24	0	0.42	0
0.21	0	0.23	0	0.21	1	1.03	0	14.87	0	0.23	0
0.20	0	0.20	0	0.18	1	3.96	1	5.88	0	0.54	0
0.19	0	0.19	0	0.26	1	0.23	1	7.69	1	2.51	0
0.17	0	0.19	0	0.21	1	0.07	1	0.18	1	0.26	0
0.20	0	0.25	0	0.21	1	0.13	1	0.18	1	0.46	0
0.19	0	0.22	0	6.96	0	0.23	1	0.24	1	0.93	0
0.21	0	0.22	0	0.34	0	0.26	1	0.17	1	0.88	0
0.21	0	1.81	1	0.35	0	0.23	1	0.18	1	0.56	0
0.21	0	0.22	1	3.27	0	0.29	1	0.18	1	0.82	0
0.26	0	0.16	1	0.35	0	0.34	1	0.17	1	6.22	0

time A	leuko A	time A	leuko A	time B	leuko B	time C	leuko C	time D	leuko D	time E	leuko E
0.22	0	0.17	1	0.28	0	1.44	1	0.18	1	0.27	0
0.20	0	0.18	1	0.24	0	3.94	0	1.10	1	1.57	0
0.27	0	0.17	1	0.23	0	0.30	0	0.20	1	0.28	0
0.43	0	0.18	1	5.31	1	1.19	0	0.50	1	0.65	0
0.20	0	0.16	1	1.07	1	0.33	0	0.43	1	4.99	1
4.95	1	0.15	1	0.31	1	0.86	0	0.75	1	0.33	1
0.23	1	0.16	1	6.70	0	2.15	0	0.23	1	0.28	1
0.22	1	0.16	1	0.37	0	2.41	0	0.22	1	3.82	0
12.31	0	0.15	1	0.45	0	0.99	0	0.28	1	0.26	0
0.24	0	1.86	0	1.32	0	0.87	0	0.28	1	0.76	0
0.21	0	0.20	0	0.35	0	3.25	1	8.19	0	0.81	0
0.21	0	0.17	0	0.36	0	0.24	1	1.18	0	4.30	0
0.16	0	0.18	0	1.28	0	0.23	1	0.29	0	0.32	0
0.23	0	0.19	0	1.18	0	0.22	1	4.24	0	3.38	0
0.33	0	0.20	0	0.61	0	0.26	1	1.80	0	0.99	0
7.77	1	0.16	0	0.93	0	0.45	1	0.31	0	1.06	0
0.31	1	0.35	0	0.30	0	0.22	1	0.92	0	0.25	0
0.23	1	0.19	0	0.26	0	0.21	1	4.13	0	0.28	0
0.21	1	0.26	0	0.51	1	4.28	0	7.17	1	0.55	0
0.18	1	0.66	0	0.28	1	0.29	0	0.19	1	0.26	0
0.20	1	0.25	0	0.24	1	0.26	0	0.20	1	1.02	0
0.18	1	0.25	0	0.22	1	1.84	0	0.19	1	0.26	0
0.18	1	0.26	0	6.35	1	0.59	0	0.16	1	0.34	0
0.19	1	0.37	0	0.29	1	0.70	0	0.17	1	0.46	0
0.19	1	2.72	1	0.93	1	0.40	0	0.28	1	6.94	1
0.19	1	0.21	1	0.28	1	0.60	0	0.32	1	0.32	1
1.68	0	0.18	1	0.22	1	0.90	0	0.96	1	0.33	1
0.31	0	0.17	1	0.34	1	1.03	0	0.37	1	1.94	0
2.76	0	0.18	1	0.24	1	0.84	0	0.56	1	0.32	0
0.85	0	0.19	1	0.22	1	0.28	0	0.21	1	0.66	0
0.24	0	0.19	1	5.05	0	0.78	0	3.59	0	0.43	0
1.34	0	0.84	0	0.38	0	1.15	0	0.63	0	1.44	0
0.24	0	0.24	0	0.47	0	3.80	1	1.37	0	1.56	0
3.22	1	0.20	0	0.74	0	0.26	1	1.05	0	1.62	0
0.24	1	0.21	0	0.42	0	0.35	1	0.97	0	0.64	0
0.20	1	0.20	0	0.33	0	0.20	1	0.54	0	0.57	0
0.19	1	0.21	0	0.29	0	0.24	1	1.39	0	0.25	0
0.18	1	0.20	0	0.30	0	0.19	1	1.14	0	0.43	0
0.18	1	0.20	0	0.63	0	0.07	1	0.91	0	0.27	0
0.16	1	0.21	0	0.98	0	0.30	1	0.64	0	0.31	0
0.18	1	0.51	0	0.44	0	1.98	1	0.44	0	1.87	0
0.17	1	0.26	0	0.29	0	0.24	1	0.99	0	0.47	0
0.18	1	0.21	0	6.09	0	0.09	1	1.12	0	0.25	0
0.15	1	1.92	1	0.34	0	0.19	1	3.04	0	0.88	0
0.09	1	0.22	1	0.30	0	3.03	0	1.59	0	0.27	0
30.53	1	0.17	1	0.24	0	0.29	0	0.39	0	1.16	0
0.24	1	0.16	1	0.85	0	0.94	0	0.36	0	0.74	0
0.24	1	0.18	1	0.35	0	0.25	0	1.16	0	0.30	0
0.22	1	1.13	0	0.33	0	0.31	0	1.03	0	0.42	0
0.23	1	0.23	0	0.29	0	1.60	0	7.83	1	6.20	1
0.20	1	0.20	0	0.41	0	0.99	0	0.22	1	0.30	1

time A	leuko A	time A	leuko A	time B	leuko B	time C	leuko C	time D	leuko D	time E	leuko E
0.18	1	0.21	0	0.31	0	0.26	0	0.21	1	0.32	1
0.18	1	0.20	0	3.05	0	0.24	0	0.25	1	0.28	1
0.29	0	0.17	0	0.29	0	0.41	0	0.20	1	0.35	1
0.26	0	0.17	0	0.26	0	0.48	0	1.16	1	0.28	1
0.18	0	2.11	1	0.58	1	0.32	0	1.92	0	1.83	0
0.20	0	0.22	1	0.28	1	1.08	0	0.38	0	0.79	0
0.21	0	2.86	1	3.85	0	0.31	0	0.29	0	1.49	0
0.10	0	0.22	1	0.26	0	0.46	0	4.63	0	0.27	0
0.24	0	0.28	1	0.23	0	0.41	0	0.30	0	1.15	0
0.19	0	0.19	1	2.26	1	0.90	0	0.32	0	0.32	0
0.18	0	0.16	1	0.31	1	1.17	0	3.86	0	1.03	0
0.18	0	0.18	1	0.36	1	0.31	0	0.72	0	0.29	0
0.19	0	0.17	1	0.21	1	3.98	1	0.31	0	3.72	0
0.18	0	0.17	1	3.44	0	0.21	1	0.44	0	0.29	0
0.22	0	1.16	0	0.34	0	0.20	1	0.31	0	0.88	0
0.20	0	0.24	0	0.73	0	0.19	1	1.76	0	0.28	0
0.19	0	0.17	0	1.09	0	0.22	1	8.02	1	0.25	0
0.18	0	0.19	0	0.42	0	0.19	1	0.19	1	0.37	0
2.90	1	0.22	0	0.27	0	0.09	1	0.19	1	0.23	0
0.24	1	0.31	0	0.24	0	0.13	1	0.19	1	6.97	1
0.19	1	0.22	0	0.24	0	1.70	0	0.23	1	0.26	1
0.18	1	0.17	0	0.36	0	0.56	0	0.20	1	0.35	1
0.20	1	0.18	0	0.26	0	0.24	0	0.18	1	0.52	1
0.17	1	0.14	0	2.14	0	0.40	0	0.17	1	0.32	1
2.44	0	0.16	0	0.73	0	0.28	0	0.19	1	0.36	1
0.26	0	0.19	0	1.57	1	0.40	0	0.21	1	0.30	1
0.26	0	0.16	0	0.27	1	0.26	0	0.16	1	0.59	1
0.22	0	0.18	0	0.23	1	0.62	0	0.24	1	3.76	0
0.23	0	3.02	1	0.21	1	0.69	0	0.23	1	0.33	0
3.70	1	0.23	1	7.31	1	0.80	0	0.25	0	1.44	0
0.25	1	0.18	1	0.25	1	0.84	0	0.24	0	3.00	0
0.21	1	0.16	1	0.81	1	1.73	0	0.62	0	0.35	0
0.17	1	0.18	1	0.24	1	0.62	0	0.30	0	0.37	0
0.22	1	0.18	1	0.21	1	0.35	0	1.18	0	2.58	0
0.19	1	3.06	0	3.31	0	0.67	0	0.32	0	0.31	0
0.85	0	0.21	0	0.42	0	0.39	0	1.51	0	0.35	0
0.20	0	0.20	0	0.33	0	0.69	0	1.25	0	0.83	0
0.21	0	3.94	1	0.31	0	0.37	0	5.36	0	0.33	0
0.18	0	0.23	1	2.70	0	0.36	0	0.70	0	0.32	0
0.22	0	0.18	1	0.33	0	3.62	1	0.83	0	1.04	0
0.25	0	0.20	1	1.56	0	0.23	1	3.04	1	0.69	0
0.17	0	0.29	1	11.78	1	0.23	1	0.18	1	1.93	0
0.22	0	0.20	1	0.25	1	0.21	1	0.23	1	0.30	0
0.24	0	0.17	1	0.22	1	0.22	1	0.21	1	0.82	0
0.23	0	0.19	1	0.36	1	0.47	1	0.21	1	7.52	1
0.21	0	0.19	1	0.19	1	0.22	1	0.22	1	0.28	1
0.23	0	0.19	1	0.38	1	13.50	0	0.21	1	0.27	1
0.18	0	0.20	1	0.21	1	0.89	0	0.20	1	0.41	1
0.20	0	0.60	0	0.21	1	1.04	0	0.21	1	0.38	1
0.18	0	0.20	0	0.20	1	0.95	0	0.18	1	0.32	1
0.20	0	0.18	0	0.33	1	1.00	0	0.17	1	0.42	1

time A	leuko A	time A	leuko A	time B	leuko B	time C	leuko C	time D	leuko D	time E	leuko E
0.19	0	0.20	0	0.27	1	1.00	0	0.71	0	0.27	1
0.18	0	0.24	0	0.24	1	0.53	0	0.20	0	3.36	0
3.64	1	0.27	0	4.34	0	0.54	0	1.46	0	0.28	0
0.25	1	0.31	0	0.80	0	1.15	0	1.01	0	1.57	0
0.19	1	3.36	1	0.34	0	0.39	0	0.23	0	0.26	0
0.19	1	0.19	1	0.29	0	0.74	0	0.26	0	0.66	0
0.07	1	0.20	1	0.25	0	1.76	0	1.03	0	0.26	0
0.14	1	0.22	1	0.27	0	0.86	0	0.94	0	0.50	0
0.23	1	0.22	1	0.29	0	0.89	0	3.30	0	0.84	0
0.13	1	0.19	1	0.75	0	0.29	0	2.58	0	0.92	0
0.16	1	1.46	0	0.31	0	2.97	1	31.20	0	1.30	0
0.75	0	0.22	0	0.26	0	0.22	1	0.50	0	2.21	0
0.24	0	0.21	0	0.26	0	0.21	1	0.38	0	0.24	0
0.14	0	0.18	0	0.25	0	0.25	1	5.22	1	0.53	0
0.37	0	0.21	0	0.24	0	0.46	1	0.21	1	0.93	0
0.22	0	0.20	0	5.42	1	1.62	0	0.21	1	2.35	0
0.21	0	0.22	0	0.31	1	0.25	0	0.17	1	2.12	0
0.23	0	0.26	0	0.07	1	0.29	0	0.18	1	1.55	0
0.17	0	2.23	1	0.11	1	0.22	0	0.17	1	0.24	0
0.21	0	0.20	1	0.28	1	0.42	0	0.18	1	NA	NA
0.21	0	0.19	1	0.22	1	0.56	0	0.19	1	NA	NA
0.21	0	0.23	1	0.65	1	0.42	0	0.19	1	NA	NA
1.99	1	0.36	1	0.22	1	0.95	0	0.19	1	NA	NA
0.24	1	0.25	1	2.78	0	1.67	0	0.19	1	NA	NA
0.08	1	0.19	1	0.39	0	1.04	0	0.17	1	NA	NA
0.11	1	0.15	1	0.31	0	1.56	0	0.19	1	NA	NA
0.18	1	0.19	1	0.62	0	0.86	0	0.19	1	NA	NA
0.18	1	0.16	1	0.31	0	4.31	1	0.22	1	NA	NA
0.17	1	0.16	1	0.64	0	0.21	1	0.01	0	NA	NA
2.17	0	0.59	0	0.29	0	0.22	1	0.19	0	NA	NA
2.48	0	0.21	0	8.52	1	0.20	1	1.11	0	NA	NA
0.21	0	0.20	0	0.24	1	0.20	1	0.49	0	NA	NA
2.83	1	0.18	0	0.63	1	1.08	1	0.65	0	NA	NA
0.25	1	0.20	0	0.22	1	0.10	1	0.79	0	NA	NA
0.18	1	0.31	0	0.34	1	1.17	0	0.94	0	NA	NA
0.19	1	0.23	0	0.24	1	3.20	0	0.84	0	NA	NA
0.17	1	0.18	0	0.42	1	0.72	0	0.93	0	NA	NA
6.87	1	0.18	0	0.24	1	1.77	0	0.48	0	NA	NA
0.26	1	2.55	1	0.21	1	1.06	0	0.63	0	NA	NA
0.19	1	0.20	1	0.20	1	2.53	0	0.71	0	NA	NA
0.19	1	0.21	1	0.20	1	3.55	1	0.35	0	NA	NA
0.18	1	0.20	1	6.11	0	0.24	1	0.26	0	NA	NA
0.19	1	0.16	1	0.37	0	0.09	1	0.33	0	NA	NA
0.17	1	0.19	1	0.31	0	0.13	1	9.51	1	NA	NA
4.40	0	0.19	1	0.28	0	0.22	1	0.21	1	NA	NA
0.24	0	0.20	1	0.34	0	0.10	1	0.19	1	NA	NA
0.20	0	0.21	1	2.53	0	0.11	1	0.43	1	NA	NA
0.20	0	0.20	1	0.60	0	0.51	1	0.19	1	NA	NA
0.18	0	0.63	0	1.51	1	0.22	1	0.19	1	NA	NA
0.18	0	0.22	0	0.29	1	0.07	1	0.18	1	NA	NA
6.70	1	0.18	0	0.22	1	0.12	1	0.34	1	NA	NA

time A	leuko A	time A	leuko A	time B	leuko B	time C	leuko C	time D	leuko D	time E	leuko E
0.23	1	0.21	0	0.20	1	0.08	1	0.19	1	NA	NA
0.22	1	0.19	0	0.32	1	6.06	0	0.18	1	NA	NA
0.18	1	0.17	0	0.23	1	5.53	0	0.19	1	NA	NA
0.18	1	0.20	0	0.21	1	2.23	0	3.39	0	NA	NA
0.19	1	0.22	0	0.28	1	8.50	1	0.29	0	NA	NA
0.16	1	0.46	0	0.22	1	0.23	1	1.18	0	NA	NA
6.77	1	0.22	0	0.22	1	4.90	1	0.41	0	NA	NA
0.25	1	0.16	0	0.20	1	0.10	1	1.09	0	NA	NA
0.19	1	0.18	0	6.58	1	4.25	1	0.48	0	NA	NA
0.19	1	0.19	0	0.31	1	0.04	1	0.24	0	NA	NA
0.27	1	0.22	0	3.37	0	0.12	1	0.30	0	NA	NA
0.21	1	0.18	0	0.36	0	0.07	1	1.40	0	NA	NA
0.19	1	3.41	1	0.66	0	0.15	1	0.96	0	NA	NA
3.90	0	0.23	1	0.32	0	0.21	1	0.94	0	NA	NA
7.92	1	0.15	1	0.64	0	0.24	1	2.66	0	NA	NA
0.24	1	0.21	1	0.31	0	4.35	0	2.93	0	NA	NA
0.19	1	0.21	1	0.91	0	1.87	0	0.61	0	NA	NA
0.19	1	0.18	1	0.43	0	3.74	1	7.02	1	NA	NA
0.18	1	0.90	1	0.39	0	0.01	1	0.21	1	NA	NA
4.90	1	0.21	1	0.50	0	0.18	1	0.17	1	NA	NA
1.72	0	0.38	0	1.24	0	0.07	1	0.21	1	NA	NA
0.25	0	0.82	0	4.50	1	0.13	1	0.19	1	NA	NA
0.36	0	0.21	0	0.24	1	0.21	1	0.22	1	NA	NA
1.16	0	0.15	0	0.39	1	0.19	1	0.21	1	NA	NA
4.49	1	0.22	0	0.23	1	0.08	1	0.20	1	NA	NA
1.05	0	0.22	0	0.47	1	0.11	1	0.22	1	NA	NA
0.23	0	0.20	0	0.23	1	0.21	1	0.20	1	NA	NA
0.23	0	0.25	0	0.21	1	1.36	0	0.16	1	NA	NA
0.24	0	0.31	0	3.92	0	3.83	0	0.28	0	NA	NA
0.23	0	0.22	0	0.45	0	0.28	0	0.21	0	NA	NA
0.23	0	0.20	0	0.38	0	3.18	0	0.19	0	NA	NA
0.18	0	0.18	0	0.46	0	4.24	1	1.39	0	NA	NA
0.17	0	0.34	0	0.27	0	0.24	1	0.36	0	NA	NA
1.76	1	0.23	0	0.37	0	0.09	1	0.48	0	NA	NA
0.26	1	0.23	0	0.28	0	4.00	0	0.43	0	NA	NA
0.21	1	2.73	1	1.07	0	0.31	0	0.63	0	NA	NA
0.20	1	0.29	1	0.36	0	1.20	0	1.11	0	NA	NA
0.19	1	0.17	1	0.42	0	0.85	0	0.56	0	NA	NA
0.16	1	0.17	1	0.33	0	1.30	0	0.55	0	NA	NA
0.19	1	0.20	1	2.22	1	1.71	0	0.36	0	NA	NA
0.19	1	0.37	1	0.24	1	0.87	0	1.65	0	NA	NA
0.19	1	0.41	1	0.34	1	4.41	1	1.89	0	NA	NA
0.18	1	0.65	0	0.22	1	0.10	1	0.97	0	NA	NA
0.19	1	0.22	0	0.22	1	0.11	1	0.68	0	NA	NA
0.17	1	0.39	0	0.21	1	2.54	0	0.59	0	NA	NA
1.62	0	0.22	0	4.94	1	1.08	0	0.48	0	NA	NA
0.64	0	0.24	0	0.27	1	0.27	0	0.25	0	NA	NA
0.99	0	0.22	0	0.23	1	0.86	0	6.72	1	NA	NA
1.54	0	0.21	0	0.22	1	0.24	0	0.20	1	NA	NA
0.52	0	0.39	0	4.72	0	1.05	0	0.26	1	NA	NA
4.85	1	NA	NA	4.34	0	0.23	0	0.17	1	NA	NA

time A	leuko A	time A	leuko A	time B	leuko B	time C	leuko C	time D	leuko D	time E	leuko E
0.27	1	NA	NA	0.27	0	0.41	0	0.37	1	NA	NA
0.20	1	NA	NA	4.27	1	3.18	0	0.17	1	NA	NA
0.19	1	NA	NA	0.25	1	3.08	1	0.48	1	NA	NA
0.19	1	NA	NA	0.22	1	0.31	1	0.19	1	NA	NA
0.18	1	NA	NA	0.55	1	0.28	1	0.28	1	NA	NA
0.19	1	NA	NA	0.20	1	0.27	1	0.89	0	NA	NA
6.31	0	NA	NA	0.41	1	0.26	1	0.36	0	NA	NA
3.01	1	NA	NA	0.22	1	0.26	1	0.57	0	NA	NA
0.27	1	NA	NA	0.21	1	0.23	1	0.96	0	NA	NA
0.20	1	NA	NA	6.02	0	0.31	1	0.26	0	NA	NA
0.58	1	NA	NA	0.36	0	0.25	1	1.32	0	NA	NA
4.28	0	NA	NA	0.29	0	0.22	1	0.32	0	NA	NA
3.24	1	NA	NA	1.12	0	1.70	0	1.59	0	NA	NA
0.35	1	NA	NA	0.27	0	2.44	0	0.79	0	NA	NA
2.40	0	NA	NA	6.59	0	0.81	0	0.74	0	NA	NA
1.01	0	NA	NA	0.28	0	0.91	0	0.97	0	NA	NA
0.31	0	NA	NA	0.38	0	4.92	0	5.19	0	NA	NA
0.26	0	NA	NA	0.24	0	0.54	0	4.76	1	NA	NA
0.85	0	NA	NA	0.33	0	2.42	1	0.20	1	NA	NA
0.37	0	NA	NA	0.21	0	0.22	1	0.18	1	NA	NA
0.29	0	NA	NA	0.80	0	0.21	1	0.32	1	NA	NA
2.06	1	NA	NA	0.24	0	0.08	1	0.19	1	NA	NA
0.24	1	NA	NA	1.99	0	0.19	1	0.45	1	NA	NA
0.21	1	NA	NA	0.20	0	0.21	1	0.20	1	NA	NA
0.55	1	NA	NA	0.48	0	0.07	1	0.19	1	NA	NA
0.53	0	NA	NA	0.24	0	0.37	1	0.35	1	NA	NA
0.42	0	NA	NA	2.85	1	0.32	1	1.48	0	NA	NA
0.30	0	NA	NA	0.27	1	0.20	1	0.27	0	NA	NA
0.27	0	NA	NA	0.23	1	0.25	1	1.30	0	NA	NA
0.55	0	NA	NA	0.22	1	0.07	1	0.18	0	NA	NA
3.54	1	NA	NA	0.29	1	6.89	0	0.30	0	NA	NA
0.24	1	NA	NA	0.18	1	9.02	0	0.30	0	NA	NA
0.25	1	NA	NA	0.26	1	NA	NA	0.83	0	NA	NA
1.11	1	NA	NA	0.20	1	NA	NA	0.25	0	NA	NA
1.22	0	NA	NA	0.21	1	NA	NA	0.44	0	NA	NA
0.42	0	NA	NA	0.31	1	NA	NA	0.43	0	NA	NA
0.40	0	NA	NA	0.24	1	NA	NA	1.00	0	NA	NA
1.19	0	NA	NA	0.23	1	NA	NA	0.26	0	NA	NA
3.43	1	NA	NA	NA	NA	NA	NA	1.02	0	NA	NA
0.27	1	NA	NA	NA	NA	NA	NA	1.31	0	NA	NA
0.26	1	NA	NA	NA	NA	NA	NA	0.30	0	NA	NA
0.20	1	NA	NA	NA	NA	NA	NA	0.39	0	NA	NA
0.07	1	NA	NA	NA	NA	NA	NA	0.83	0	NA	NA
0.10	1	NA	NA	NA	NA	NA	NA	0.38	0	NA	NA
1.42	0	NA	NA	NA	NA	NA	NA	0.31	0	NA	NA
0.37	0	NA	NA	NA	NA	NA	NA	0.50	0	NA	NA
0.96	0	NA	NA	NA	NA	NA	NA	NA	NA	NA	NA
0.29	0	NA	NA	NA	NA	NA	NA	NA	NA	NA	NA
0.25	0	NA	NA	NA	NA	NA	NA	NA	NA	NA	NA
1.49	0	NA	NA	NA	NA	NA	NA	NA	NA	NA	NA

1.8 SL007

Table 8: Raw data of TBS SL007.

time A	leuko A	time A	leuko A	time B	leuko B	time D	leuko D	time E	leuko E
0.00	1	0.00	1	0.00	0	0.00	1	0.00	1
0.27	1	0.29	1	1.39	0	0.23	1	0.24	1
0.21	1	0.61	1	1.73	0	0.19	1	0.26	1
0.24	1	0.25	1	2.28	0	0.33	1	0.27	1
1.14	1	0.21	1	2.88	1	0.20	1	0.26	1
0.22	1	0.21	1	0.36	1	0.28	1	0.27	1
0.20	1	0.19	1	6.43	1	0.21	1	0.29	1
0.20	1	0.19	1	0.29	1	0.18	1	0.67	1
0.19	1	0.18	1	1.73	1	0.25	1	0.28	1
0.22	1	0.19	1	0.30	1	0.21	1	0.36	1
0.19	1	0.23	1	0.53	1	0.28	1	6.01	1
0.20	1	2.95	0	0.25	1	0.20	1	0.33	1
0.20	1	10.47	1	0.25	1	0.27	1	0.24	1
0.21	1	0.24	1	0.20	1	0.21	1	0.28	1
0.19	1	0.36	1	6.25	0	0.21	1	0.26	1
0.17	1	0.19	1	1.12	0	0.37	1	0.28	1
4.03	0	0.18	1	5.05	0	0.17	1	0.24	1
1.99	1	0.18	1	1.17	1	0.34	1	0.30	1
0.27	1	0.17	1	0.28	1	0.20	1	0.29	1
0.20	1	0.19	1	0.25	1	0.23	1	0.27	1
0.20	1	0.17	1	0.66	1	0.43	1	0.27	1
0.21	1	0.17	1	0.26	1	0.35	1	63.90	0
0.18	1	0.18	1	0.22	1	0.41	1	4.15	1
0.18	1	0.18	1	0.23	1	0.21	1	0.36	1
0.22	1	0.20	1	0.55	1	0.39	1	0.26	1
0.19	1	0.16	1	0.26	1	0.26	1	0.24	1
0.30	1	0.16	1	0.24	1	0.63	1	9.03	1
0.18	1	0.61	0	0.88	1	0.34	1	0.28	1
0.19	1	5.18	1	0.25	1	0.78	1	0.27	1
5.30	1	0.30	1	0.36	1	6.54	0	0.25	1
0.23	1	0.20	1	0.22	1	6.53	0	0.93	1
0.20	1	0.17	1	0.22	1	20.14	0	0.29	1
0.19	1	0.18	1	7.74	1	1.66	0	0.04	1
0.19	1	0.18	1	0.27	1	18.13	1	0.01	1
0.16	1	0.13	1	0.26	1	0.73	1	0.25	1
0.22	1	0.24	1	0.22	1	0.42	1	0.49	1
0.26	1	0.20	1	2.15	0	0.23	1	0.39	1
0.17	1	0.21	1	6.76	1	0.20	1	0.62	1
0.19	1	0.19	1	0.30	1	0.21	1	0.27	1
0.10	1	0.19	1	0.23	1	0.17	1	0.30	1
0.18	1	0.19	1	0.24	1	0.44	1	0.29	1
0.42	1	0.17	1	0.23	1	0.21	1	0.05	1
2.13	1	0.18	1	0.38	1	0.20	1	0.23	1
0.25	1	4.33	0	0.23	1	0.18	1	0.29	1
0.19	1	3.46	1	0.26	1	0.38	1	0.29	1
0.19	1	0.28	1	0.45	1	0.28	1	0.30	1
0.21	1	0.18	1	0.27	1	0.20	1	0.03	1
0.18	1	0.18	1	0.20	1	0.22	1	2.04	0
0.21	1	0.18	1	5.43	0	0.20	1	7.61	0

time A	leuko A	time A	leuko A	time B	leuko B	time D	leuko D	time E	leuko E
0.17	1	0.09	1	1.59	0	0.31	1	10.07	1
0.07	1	0.25	1	7.65	1	0.19	1	0.33	1
0.11	1	0.33	1	0.27	1	0.23	1	0.42	1
0.26	1	0.32	1	0.23	1	0.19	1	0.27	1
0.11	1	0.17	1	0.23	1	0.19	1	0.28	1
0.18	1	0.20	1	1.84	1	10.02	0	0.28	1
0.21	1	0.20	1	0.23	1	3.99	0	0.33	1
3.19	1	0.17	1	0.23	1	1.97	0	0.60	1
0.40	1	2.58	1	3.93	0	15.88	1	0.62	1
0.27	1	0.24	1	5.86	0	0.21	1	0.33	1
0.18	1	0.18	1	5.53	1	0.44	1	6.29	0
0.19	1	0.19	1	0.29	1	0.20	1	14.78	1
0.20	1	0.18	1	0.26	1	0.53	1	0.27	1
0.18	1	0.19	1	1.27	1	0.19	1	0.28	1
0.18	1	0.19	1	0.25	1	0.67	1	0.30	1
0.20	1	0.18	1	0.24	1	0.22	1	0.31	1
0.18	1	0.18	1	0.20	1	0.42	1	0.34	1
0.22	1	0.17	1	0.28	1	0.22	1	0.48	1
0.20	1	0.19	1	0.95	1	0.22	1	0.07	1
0.19	1	0.19	1	9.19	1	0.23	1	0.28	1
0.23	1	0.20	1	0.27	1	0.19	1	10.05	0
2.77	1	3.26	0	0.22	1	0.24	1	14.47	1
0.26	1	2.90	1	0.68	1	0.22	1	0.29	1
0.20	1	0.26	1	0.20	1	0.25	1	0.29	1
0.21	1	0.19	1	0.38	1	0.23	1	0.25	1
0.19	1	0.19	1	0.83	1	4.27	0	0.04	1
0.20	1	0.19	1	4.50	0	1.61	0	0.24	1
0.19	1	0.18	1	2.73	0	1.23	0	0.05	1
0.19	1	0.19	1	0.33	0	9.28	0	0.01	1
0.09	1	0.18	1	5.86	1	1.24	0	0.02	1
0.10	1	0.19	1	0.26	1	3.52	0	0.19	1
4.62	0	0.19	1	0.22	1	1.75	0	0.26	1
7.52	0	0.20	1	0.18	1	0.29	0	0.26	1
8.37	0	0.39	1	1.62	1	5.05	0	10.21	0
3.60	1	6.26	1	0.23	1	4.12	1	5.88	0
0.40	1	0.27	1	0.18	1	0.19	1	7.24	1
0.29	1	0.19	1	0.19	1	0.14	1	0.27	1
0.23	1	0.19	1	17.49	1	0.17	1	0.25	1
0.22	1	0.20	1	0.27	1	0.69	1	0.29	1
0.44	1	0.20	1	1.11	1	0.23	1	0.27	1
0.22	1	0.13	1	0.22	1	0.22	1	0.30	1
3.30	0	0.27	1	0.57	1	0.24	1	0.30	1
5.13	1	0.21	1	0.25	1	0.38	1	0.30	1
0.29	1	0.16	1	0.22	1	0.26	1	0.28	1
0.23	1	0.17	1	0.20	1	0.22	1	0.26	1
0.20	1	0.10	1	14.93	1	0.21	1	0.37	1
0.21	1	0.24	1	0.22	1	0.20	1	19.52	1
0.22	1	0.22	1	0.22	1	0.17	1	0.33	1
0.21	1	0.20	1	0.19	1	0.23	1	0.29	1
0.21	1	0.18	1	1.08	1	0.20	1	0.26	1
0.19	1	0.18	1	0.22	1	0.23	1	0.25	1
0.20	1	0.22	1	0.20	1	0.31	1	0.27	1

time A	leuko A	time A	leuko A	time B	leuko B	time D	leuko D	time E	leuko E
0.19	1	0.18	1	0.20	1	3.93	0	0.29	1
0.20	1	4.48	1	0.42	1	2.16	0	0.24	1
0.21	1	0.24	1	0.22	1	1.32	0	0.31	1
0.22	1	0.19	1	0.21	1	7.20	0	0.26	1
0.19	1	0.22	1	0.19	1	0.96	0	0.26	1
0.22	1	0.20	1	16.40	0	1.02	0	8.44	0
11.23	1	0.24	1	0.72	0	2.18	0	10.51	1
0.24	1	0.21	1	3.38	1	14.53	1	0.55	1
0.21	1	0.21	1	0.28	1	0.22	1	0.33	1
0.21	1	0.20	1	1.01	1	0.22	1	0.27	1
0.20	1	0.17	1	0.24	1	0.23	1	0.28	1
0.20	1	0.18	1	6.54	1	0.19	1	0.27	1
0.20	1	0.17	1	0.24	1	0.32	1	0.09	1
0.13	1	0.19	1	0.21	1	0.20	1	24.14	1
0.26	1	0.21	1	0.22	1	0.20	1	0.27	1
0.26	1	0.17	1	0.19	1	0.28	1	0.27	1
0.20	1	0.21	1	3.29	1	0.22	1	0.26	1
0.20	1	0.20	1	0.24	1	0.22	1	0.01	1
0.19	1	0.17	1	0.21	1	0.21	1	0.28	1
0.22	1	0.17	1	0.20	1	0.20	1	0.07	1
0.18	1	0.59	0	0.20	1	0.19	1	0.26	1
0.16	1	0.23	0	1.35	0	0.21	1	0.01	1
13.57	1	0.28	0	18.46	1	0.20	1	0.18	1
0.35	1	4.54	1	0.29	1	0.19	1	0.01	1
0.28	1	0.26	1	0.71	1	0.20	1	0.29	1
0.41	1	0.21	1	0.60	1	0.19	1	0.08	1
0.25	1	0.19	1	0.24	1	0.20	1	0.18	1
0.20	1	0.20	1	0.23	1	0.21	1	4.68	0
0.20	1	0.18	1	0.20	1	0.20	1	11.21	1
0.18	1	0.17	1	0.20	1	0.29	1	0.29	1
0.20	1	0.19	1	14.29	1	0.22	1	0.26	1
0.21	1	0.20	1	0.22	1	0.19	1	0.27	1
14.55	1	0.17	1	0.50	1	0.20	1	0.26	1
0.28	1	0.18	1	0.26	1	0.27	1	0.25	1
0.20	1	0.19	1	0.23	1	0.29	1	0.27	1
0.21	1	0.19	1	0.22	1	0.17	1	0.29	1
0.20	1	0.19	1	0.19	1	0.17	1	0.29	1
0.19	1	0.19	1	0.25	1	1.58	0	0.45	1
0.21	1	2.77	0	7.75	1	12.82	0	5.97	0
0.20	1	3.41	0	0.24	1	6.79	1	9.47	1
0.19	1	3.77	1	0.83	1	0.19	1	0.31	1
0.21	1	0.26	1	0.22	1	0.29	1	0.25	1
0.20	1	0.21	1	8.16	1	0.28	1	0.29	1
0.22	1	0.21	1	0.27	1	0.28	1	0.27	1
0.21	1	0.09	1	0.22	1	0.41	1	0.24	1
0.21	1	0.28	1	0.22	1	0.33	1	0.27	1
0.22	1	0.18	1	0.20	1	0.20	1	0.26	1
0.20	1	0.19	1	0.33	1	0.20	1	0.27	1
0.23	1	0.19	1	0.21	1	0.18	1	0.27	1
0.20	1	0.18	1	3.82	0	0.17	1	0.25	1
0.18	1	0.19	1	13.60	1	0.21	1	0.06	1
0.17	1	0.22	1	0.24	1	0.18	1	0.19	1

time A	leuko A	time A	leuko A	time B	leuko B	time D	leuko D	time E	leuko E
7.65	1	3.75	1	4.06	0	0.18	1	0.08	1
0.37	1	0.24	1	0.70	1	0.15	1	0.18	1
0.25	1	0.31	1	0.31	1	0.18	1	0.01	1
0.22	1	0.21	1	0.23	1	0.18	1	0.23	1
0.23	1	0.17	1	0.21	1	0.17	1	0.05	1
0.21	1	0.18	1	0.40	1	0.18	1	24.25	1
0.21	1	0.16	1	4.93	1	0.17	1	0.28	1
0.20	1	0.16	1	0.26	1	0.18	1	0.28	1
0.22	1	0.19	1	0.59	1	0.20	1	0.26	1
0.20	1	0.19	1	7.16	1	0.19	1	0.06	1
0.19	1	0.20	1	0.27	1	0.16	1	0.20	1
0.19	1	0.19	1	9.78	1	0.18	1	0.29	1
8.27	1	0.19	1	0.27	1	0.16	1	0.32	1
0.30	1	0.21	1	0.23	1	0.16	1	0.57	1
0.22	1	0.20	1	0.73	1	0.51	1	0.31	1
0.20	1	0.40	1	5.80	0	15.72	0	0.29	1
0.22	1	8.99	1	2.25	0	14.77	1	0.02	1
0.20	1	0.25	1	1.97	1	0.22	1	1.07	0
0.20	1	0.20	1	0.29	1	0.19	1	15.38	1
0.23	1	0.20	1	0.22	1	0.37	1	0.28	1
0.21	1	0.21	1	0.21	1	0.21	1	0.30	1
0.24	1	0.36	1	0.27	1	0.20	1	0.27	1
0.20	1	0.22	1	4.12	1	0.18	1	0.26	1
0.27	1	0.21	1	1.05	1	0.31	1	0.56	1
0.29	1	0.19	1	0.64	1	0.23	1	0.29	1
0.21	1	0.20	1	15.53	1	0.20	1	0.31	1
0.18	1	0.19	1	0.80	0	0.20	1	0.40	1
11.18	1	0.19	1	8.48	1	0.23	1	25.88	1
0.30	1	0.19	1	0.33	1	0.18	1	0.30	1
0.26	1	0.19	1	8.11	1	0.27	1	0.34	1
0.23	1	1.94	0	0.34	1	0.21	1	0.31	1
0.23	1	3.74	0	0.43	1	0.21	1	0.31	1
0.23	1	3.06	1	0.27	1	0.21	1	0.30	1
0.20	1	0.24	1	6.79	0	0.18	1	0.30	1
0.20	1	0.18	1	3.90	1	0.20	1	0.26	1
0.23	1	0.19	1	0.38	1	0.21	1	0.31	1
0.17	1	0.19	1	0.49	1	0.20	1	0.33	1
0.20	1	0.18	1	0.24	1	0.23	1	0.26	1
0.21	1	0.17	1	0.24	1	0.21	1	0.01	1
6.67	1	0.18	1	0.37	1	0.22	1	2.10	0
0.27	1	0.20	1	0.27	1	0.20	1	7.27	0
0.21	1	0.22	1	0.22	1	0.19	1	0.36	0
0.19	1	0.23	1	0.23	1	0.18	1	11.90	1
0.19	1	0.50	0	0.32	1	0.18	1	0.26	1
0.18	1	3.49	1	0.20	1	0.15	1	0.11	1
0.13	1	0.22	1	0.22	1	1.07	0	0.15	1
0.27	1	0.20	1	4.69	0	6.25	0	0.45	1
0.21	1	0.18	1	13.49	0	13.20	1	0.28	1
0.17	1	0.17	1	1.01	1	0.20	1	0.40	1
11.36	1	0.21	1	0.27	1	0.19	1	0.31	1
0.25	1	0.17	1	0.24	1	0.25	1	0.33	1
0.18	1	0.23	1	1.89	1	0.18	1	0.27	1

time A	leuko A	time A	leuko A	time B	leuko B	time D	leuko D	time E	leuko E
0.17	1	0.24	1	0.26	1	0.21	1	0.24	1
0.18	1	0.19	1	0.23	1	0.17	1	24.25	1
0.18	1	0.16	1	9.70	1	0.20	1	0.34	1
0.17	1	8.87	0	0.22	1	0.20	1	0.77	1
0.17	1	2.54	1	0.20	1	0.25	1	0.31	1
0.19	1	0.25	1	0.23	1	0.18	1	0.29	1
0.17	1	0.20	1	2.03	1	0.19	1	0.29	1
0.18	1	0.17	1	0.21	1	0.54	1	0.31	1
0.20	1	0.17	1	0.20	1	0.20	1	16.65	0
0.21	1	0.18	1	0.19	1	0.21	1	13.19	1
0.21	1	0.18	1	6.24	0	0.18	1	0.24	1
0.20	1	0.18	1	2.99	0	0.23	1	0.32	1
0.36	1	0.18	1	8.93	0	0.19	1	0.30	1
0.17	1	0.19	1	0.86	0	0.20	1	0.29	1
0.21	1	0.17	1	2.57	1	0.17	1	0.27	1
0.20	1	0.18	1	0.27	1	0.18	1	0.27	1
0.17	1	0.18	1	0.21	1	0.25	1	0.09	1
35.48	1	0.20	1	0.19	1	0.22	1	0.18	1
0.26	1	0.25	1	0.48	1	0.22	1	0.01	1
0.21	1	0.20	1	0.26	1	0.20	1	0.01	1
0.20	1	NA	NA	0.41	1	0.20	1	0.28	1
0.21	1	NA	NA	0.22	1	0.18	1	0.03	1
0.22	1	NA	NA	0.20	1	0.16	1	0.01	1
0.20	1	NA	NA	0.21	1	10.83	0	0.18	1
0.22	1	NA	NA	5.48	0	2.30	0	0.06	1
0.19	1	NA	NA	2.28	1	17.81	1	0.01	1
0.18	1	NA	NA	0.21	1	0.22	1	0.16	1
0.19	1	NA	NA	0.22	1	0.42	1	0.44	1
0.15	1	NA	NA	0.21	1	0.21	1	0.30	1
18.18	1	NA	NA	0.19	1	0.30	1	0.30	1
0.35	1	NA	NA	0.65	1	0.18	1	0.39	1
0.25	1	NA	NA	0.19	1	0.19	1	0.05	1
0.19	1	NA	NA	0.50	1	0.32	1	2.02	0
0.22	1	NA	NA	0.25	1	0.25	1	7.55	0
0.21	1	NA	NA	0.24	1	0.27	1	2.19	0
0.08	1	NA	NA	0.21	1	0.18	1	5.01	0
0.10	1	NA	NA	0.64	1	0.21	1	NA	NA
0.18	1	NA	NA	0.28	1	0.22	1	NA	NA
0.18	1	NA	NA	0.22	1	0.19	1	NA	NA
0.20	1	NA	NA	0.96	0	0.22	1	NA	NA
0.18	1	NA	NA	NA	NA	0.19	1	NA	NA
0.21	1	NA	NA	NA	NA	0.91	1	NA	NA
0.21	1	NA	NA	NA	NA	0.24	1	NA	NA
0.18	1	NA	NA	NA	NA	0.21	1	NA	NA
0.20	1	NA	NA	NA	NA	0.20	1	NA	NA
0.19	1	NA	NA	NA	NA	0.25	1	NA	NA
0.20	1	NA	NA	NA	NA	0.23	1	NA	NA
0.14	1	NA	NA	NA	NA	2.13	0	NA	NA
9.31	0	NA	NA	NA	NA	22.67	1	NA	NA
5.09	1	NA	NA	NA	NA	0.20	1	NA	NA
0.26	1	NA	NA	NA	NA	0.35	1	NA	NA
0.19	1	NA	NA	NA	NA	0.21	1	NA	NA

time A	leuko A	time A	leuko A	time B	leuko B	time D	leuko D	time E	leuko E
0.21	1	NA	NA	NA	NA	0.22	1	NA	NA
0.17	1	NA	NA	NA	NA	0.22	1	NA	NA
0.36	1	NA	NA	NA	NA	0.19	1	NA	NA
0.22	1	NA	NA	NA	NA	0.18	1	NA	NA
0.18	1	NA	NA	NA	NA	0.20	1	NA	NA
0.18	1	NA	NA	NA	NA	0.21	1	NA	NA
0.21	1	NA	NA	NA	NA	0.19	1	NA	NA
0.18	1	NA	NA	NA	NA	0.21	1	NA	NA
0.18	1	NA	NA	NA	NA	0.51	1	NA	NA
0.21	1	NA	NA	NA	NA	0.22	1	NA	NA
6.95	1	NA	NA	NA	NA	0.19	1	NA	NA
0.29	1	NA	NA	NA	NA	0.21	1	NA	NA
0.21	1	NA	NA	NA	NA	0.22	1	NA	NA
0.22	1	NA	NA	NA	NA	0.20	1	NA	NA
0.16	1	NA	NA	NA	NA	0.21	1	NA	NA
0.32	1	NA	NA	NA	NA	0.23	1	NA	NA
0.12	1	NA	NA	NA	NA	0.21	1	NA	NA
0.21	1	NA	NA	NA	NA	3.41	0	NA	NA
0.24	1	NA	NA	NA	NA	0.26	0	NA	NA
0.19	1	NA	NA	NA	NA	0.77	0	NA	NA
0.21	1	NA	NA	NA	NA	2.62	0	NA	NA
0.22	1	NA	NA	NA	NA	6.57	0	NA	NA
19.81	1	NA	NA	NA	NA	3.96	0	NA	NA
0.28	1	NA	NA	NA	NA	NA	NA	NA	NA
0.19	1	NA	NA	NA	NA	NA	NA	NA	NA
0.20	1	NA	NA	NA	NA	NA	NA	NA	NA
0.21	1	NA	NA	NA	NA	NA	NA	NA	NA
0.21	1	NA	NA	NA	NA	NA	NA	NA	NA
0.21	1	NA	NA	NA	NA	NA	NA	NA	NA
0.20	1	NA	NA	NA	NA	NA	NA	NA	NA
0.03	1	NA	NA	NA	NA	NA	NA	NA	NA
0.03	1	NA	NA	NA	NA	NA	NA	NA	NA
0.11	1	NA	NA	NA	NA	NA	NA	NA	NA
0.07	1	NA	NA	NA	NA	NA	NA	NA	NA
0.09	1	NA	NA	NA	NA	NA	NA	NA	NA
0.19	1	NA	NA	NA	NA	NA	NA	NA	NA
0.22	1	NA	NA	NA	NA	NA	NA	NA	NA
0.41	1	NA	NA	NA	NA	NA	NA	NA	NA
1.28	0	NA	NA	NA	NA	NA	NA	NA	NA
2.75	0	NA	NA	NA	NA	NA	NA	NA	NA
6.27	1	NA	NA	NA	NA	NA	NA	NA	NA
0.29	1	NA	NA	NA	NA	NA	NA	NA	NA
0.17	1	NA	NA	NA	NA	NA	NA	NA	NA
0.17	1	NA	NA	NA	NA	NA	NA	NA	NA
0.18	1	NA	NA	NA	NA	NA	NA	NA	NA
0.17	1	NA	NA	NA	NA	NA	NA	NA	NA
0.19	1	NA	NA	NA	NA	NA	NA	NA	NA
0.27	1	NA	NA	NA	NA	NA	NA	NA	NA
0.20	1	NA	NA	NA	NA	NA	NA	NA	NA
0.17	1	NA	NA	NA	NA	NA	NA	NA	NA
0.16	1	NA	NA	NA	NA	NA	NA	NA	NA
0.19	1	NA	NA	NA	NA	NA	NA	NA	NA

time A	leuko A	time A	leuko A	time B	leuko B	time D	leuko D	time E	leuko E
0.18	1	NA	NA	NA	NA	NA	NA	NA	NA
0.15	1	NA	NA	NA	NA	NA	NA	NA	NA
0.17	1	NA	NA	NA	NA	NA	NA	NA	NA
0.15	1	NA	NA	NA	NA	NA	NA	NA	NA
2.79	0	NA	NA	NA	NA	NA	NA	NA	NA
6.26	1	NA	NA	NA	NA	NA	NA	NA	NA
0.26	1	NA	NA	NA	NA	NA	NA	NA	NA
0.17	1	NA	NA	NA	NA	NA	NA	NA	NA
0.18	1	NA	NA	NA	NA	NA	NA	NA	NA
0.17	1	NA	NA	NA	NA	NA	NA	NA	NA
0.19	1	NA	NA	NA	NA	NA	NA	NA	NA
0.18	1	NA	NA	NA	NA	NA	NA	NA	NA
0.17	1	NA	NA	NA	NA	NA	NA	NA	NA
0.19	1	NA	NA	NA	NA	NA	NA	NA	NA
0.22	1	NA	NA	NA	NA	NA	NA	NA	NA
0.21	1	NA	NA	NA	NA	NA	NA	NA	NA
0.20	1	NA	NA	NA	NA	NA	NA	NA	NA
0.31	1	NA	NA	NA	NA	NA	NA	NA	NA
0.20	1	NA	NA	NA	NA	NA	NA	NA	NA
0.18	1	NA	NA	NA	NA	NA	NA	NA	NA
0.17	1	NA	NA	NA	NA	NA	NA	NA	NA
0.19	1	NA	NA	NA	NA	NA	NA	NA	NA
0.21	1	NA	NA	NA	NA	NA	NA	NA	NA
4.81	1	NA	NA	NA	NA	NA	NA	NA	NA
0.24	1	NA	NA	NA	NA	NA	NA	NA	NA
0.19	1	NA	NA	NA	NA	NA	NA	NA	NA
0.19	1	NA	NA	NA	NA	NA	NA	NA	NA
0.18	1	NA	NA	NA	NA	NA	NA	NA	NA
0.19	1	NA	NA	NA	NA	NA	NA	NA	NA
0.18	1	NA	NA	NA	NA	NA	NA	NA	NA
0.17	1	NA	NA	NA	NA	NA	NA	NA	NA
0.20	1	NA	NA	NA	NA	NA	NA	NA	NA
0.16	1	NA	NA	NA	NA	NA	NA	NA	NA
0.18	1	NA	NA	NA	NA	NA	NA	NA	NA
0.16	1	NA	NA	NA	NA	NA	NA	NA	NA
0.14	1	NA	NA	NA	NA	NA	NA	NA	NA
0.15	1	NA	NA	NA	NA	NA	NA	NA	NA
0.36	1	NA	NA	NA	NA	NA	NA	NA	NA
0.20	1	NA	NA	NA	NA	NA	NA	NA	NA
0.17	1	NA	NA	NA	NA	NA	NA	NA	NA
0.18	1	NA	NA	NA	NA	NA	NA	NA	NA
0.20	1	NA	NA	NA	NA	NA	NA	NA	NA
0.18	1	NA	NA	NA	NA	NA	NA	NA	NA
6.98	1	NA	NA	NA	NA	NA	NA	NA	NA
0.31	1	NA	NA	NA	NA	NA	NA	NA	NA
0.22	1	NA	NA	NA	NA	NA	NA	NA	NA
0.17	1	NA	NA	NA	NA	NA	NA	NA	NA
0.19	1	NA	NA	NA	NA	NA	NA	NA	NA
0.36	1	NA	NA	NA	NA	NA	NA	NA	NA
0.21	1	NA	NA	NA	NA	NA	NA	NA	NA
0.17	1	NA	NA	NA	NA	NA	NA	NA	NA
0.18	1	NA	NA	NA	NA	NA	NA	NA	NA

time A	leuko A	time A	leuko A	time B	leuko B	time D	leuko D	time E	leuko E
0.20	1	NA	NA	NA	NA	NA	NA	NA	NA
0.18	1	NA	NA	NA	NA	NA	NA	NA	NA
0.19	1	NA	NA	NA	NA	NA	NA	NA	NA
0.19	1	NA	NA	NA	NA	NA	NA	NA	NA
0.16	1	NA	NA	NA	NA	NA	NA	NA	NA
6.04	1	NA	NA	NA	NA	NA	NA	NA	NA
0.25	1	NA	NA	NA	NA	NA	NA	NA	NA
0.19	1	NA	NA	NA	NA	NA	NA	NA	NA
0.18	1	NA	NA	NA	NA	NA	NA	NA	NA
0.19	1	NA	NA	NA	NA	NA	NA	NA	NA
0.18	1	NA	NA	NA	NA	NA	NA	NA	NA
0.20	1	NA	NA	NA	NA	NA	NA	NA	NA
0.19	1	NA	NA	NA	NA	NA	NA	NA	NA
0.22	1	NA	NA	NA	NA	NA	NA	NA	NA
0.28	1	NA	NA	NA	NA	NA	NA	NA	NA
0.19	1	NA	NA	NA	NA	NA	NA	NA	NA
0.19	1	NA	NA	NA	NA	NA	NA	NA	NA
0.21	1	NA	NA	NA	NA	NA	NA	NA	NA
0.16	1	NA	NA	NA	NA	NA	NA	NA	NA
0.18	1	NA	NA	NA	NA	NA	NA	NA	NA
0.14	1	NA	NA	NA	NA	NA	NA	NA	NA
0.20	1	NA	NA	NA	NA	NA	NA	NA	NA
0.71	1	NA	NA	NA	NA	NA	NA	NA	NA
0.30	1	NA	NA	NA	NA	NA	NA	NA	NA
0.22	1	NA	NA	NA	NA	NA	NA	NA	NA
0.68	0	NA	NA	NA	NA	NA	NA	NA	NA
1.04	0	NA	NA	NA	NA	NA	NA	NA	NA
5.91	0	NA	NA	NA	NA	NA	NA	NA	NA
8.21	1	NA	NA	NA	NA	NA	NA	NA	NA
0.30	1	NA	NA	NA	NA	NA	NA	NA	NA
0.21	1	NA	NA	NA	NA	NA	NA	NA	NA
0.19	1	NA	NA	NA	NA	NA	NA	NA	NA
0.21	1	NA	NA	NA	NA	NA	NA	NA	NA
0.27	1	NA	NA	NA	NA	NA	NA	NA	NA
0.27	1	NA	NA	NA	NA	NA	NA	NA	NA
0.28	1	NA	NA	NA	NA	NA	NA	NA	NA
0.24	1	NA	NA	NA	NA	NA	NA	NA	NA
0.20	1	NA	NA	NA	NA	NA	NA	NA	NA
0.18	1	NA	NA	NA	NA	NA	NA	NA	NA
0.22	1	NA	NA	NA	NA	NA	NA	NA	NA
0.18	1	NA	NA	NA	NA	NA	NA	NA	NA
0.19	1	NA	NA	NA	NA	NA	NA	NA	NA
0.21	1	NA	NA	NA	NA	NA	NA	NA	NA
0.20	1	NA	NA	NA	NA	NA	NA	NA	NA
0.25	1	NA	NA	NA	NA	NA	NA	NA	NA
0.24	1	NA	NA	NA	NA	NA	NA	NA	NA
0.22	1	NA	NA	NA	NA	NA	NA	NA	NA
0.32	1	NA	NA	NA	NA	NA	NA	NA	NA
25.73	1	NA	NA	NA	NA	NA	NA	NA	NA
0.30	1	NA	NA	NA	NA	NA	NA	NA	NA
0.21	1	NA	NA	NA	NA	NA	NA	NA	NA
0.10	1	NA	NA	NA	NA	NA	NA	NA	NA

time A	leuko A	time A	leuko A	time B	leuko B	time D	leuko D	time E	leuko E
0.08	1	NA	NA	NA	NA	NA	NA	NA	NA
0.10	1	NA	NA	NA	NA	NA	NA	NA	NA
0.09	1	NA	NA	NA	NA	NA	NA	NA	NA
0.09	1	NA	NA	NA	NA	NA	NA	NA	NA
0.12	1	NA	NA	NA	NA	NA	NA	NA	NA
0.06	1	NA	NA	NA	NA	NA	NA	NA	NA
0.09	1	NA	NA	NA	NA	NA	NA	NA	NA
0.64	0	NA	NA	NA	NA	NA	NA	NA	NA
5.70	0	NA	NA	NA	NA	NA	NA	NA	NA
4.74	1	NA	NA	NA	NA	NA	NA	NA	NA
0.24	1	NA	NA	NA	NA	NA	NA	NA	NA
0.19	1	NA	NA	NA	NA	NA	NA	NA	NA
0.17	1	NA	NA	NA	NA	NA	NA	NA	NA
0.18	1	NA	NA	NA	NA	NA	NA	NA	NA
0.19	1	NA	NA	NA	NA	NA	NA	NA	NA
0.17	1	NA	NA	NA	NA	NA	NA	NA	NA
0.17	1	NA	NA	NA	NA	NA	NA	NA	NA
0.01	1	NA	NA	NA	NA	NA	NA	NA	NA
0.17	1	NA	NA	NA	NA	NA	NA	NA	NA
0.20	1	NA	NA	NA	NA	NA	NA	NA	NA
0.19	1	NA	NA	NA	NA	NA	NA	NA	NA
0.19	1	NA	NA	NA	NA	NA	NA	NA	NA
0.16	1	NA	NA	NA	NA	NA	NA	NA	NA
0.18	1	NA	NA	NA	NA	NA	NA	NA	NA
0.19	1	NA	NA	NA	NA	NA	NA	NA	NA
2.37	1	NA	NA	NA	NA	NA	NA	NA	NA
0.27	1	NA	NA	NA	NA	NA	NA	NA	NA
0.51	1	NA	NA	NA	NA	NA	NA	NA	NA
0.21	1	NA	NA	NA	NA	NA	NA	NA	NA
0.17	1	NA	NA	NA	NA	NA	NA	NA	NA
0.18	1	NA	NA	NA	NA	NA	NA	NA	NA
0.16	1	NA	NA	NA	NA	NA	NA	NA	NA
0.16	1	NA	NA	NA	NA	NA	NA	NA	NA
0.16	1	NA	NA	NA	NA	NA	NA	NA	NA
0.40	1	NA	NA	NA	NA	NA	NA	NA	NA
0.28	1	NA	NA	NA	NA	NA	NA	NA	NA
0.18	1	NA	NA	NA	NA	NA	NA	NA	NA
0.18	1	NA	NA	NA	NA	NA	NA	NA	NA
0.18	1	NA	NA	NA	NA	NA	NA	NA	NA
0.19	1	NA	NA	NA	NA	NA	NA	NA	NA
0.18	1	NA	NA	NA	NA	NA	NA	NA	NA
0.17	1	NA	NA	NA	NA	NA	NA	NA	NA
0.19	1	NA	NA	NA	NA	NA	NA	NA	NA
0.15	1	NA	NA	NA	NA	NA	NA	NA	NA
0.16	1	NA	NA	NA	NA	NA	NA	NA	NA
0.18	1	NA	NA	NA	NA	NA	NA	NA	NA
20.87	1	NA	NA	NA	NA	NA	NA	NA	NA
0.25	1	NA	NA	NA	NA	NA	NA	NA	NA
0.20	1	NA	NA	NA	NA	NA	NA	NA	NA
0.20	1	NA	NA	NA	NA	NA	NA	NA	NA
0.21	1	NA	NA	NA	NA	NA	NA	NA	NA
0.19	1	NA	NA	NA	NA	NA	NA	NA	NA

time A	leuko A	time A	leuko A	time B	leuko B	time D	leuko D	time E	leuko E
0.19	1	NA	NA	NA	NA	NA	NA	NA	NA
0.16	1	NA	NA	NA	NA	NA	NA	NA	NA
0.18	1	NA	NA	NA	NA	NA	NA	NA	NA
0.18	1	NA	NA	NA	NA	NA	NA	NA	NA
0.15	1	NA	NA	NA	NA	NA	NA	NA	NA
0.15	1	NA	NA	NA	NA	NA	NA	NA	NA
0.16	1	NA	NA	NA	NA	NA	NA	NA	NA
0.17	1	NA	NA	NA	NA	NA	NA	NA	NA
0.17	1	NA	NA	NA	NA	NA	NA	NA	NA
0.18	1	NA	NA	NA	NA	NA	NA	NA	NA
0.12	1	NA	NA	NA	NA	NA	NA	NA	NA
0.32	1	NA	NA	NA	NA	NA	NA	NA	NA
18.96	1	NA	NA	NA	NA	NA	NA	NA	NA
0.27	1	NA	NA	NA	NA	NA	NA	NA	NA
0.21	1	NA	NA	NA	NA	NA	NA	NA	NA
0.20	1	NA	NA	NA	NA	NA	NA	NA	NA
0.08	1	NA	NA	NA	NA	NA	NA	NA	NA
0.10	1	NA	NA	NA	NA	NA	NA	NA	NA
0.16	1	NA	NA	NA	NA	NA	NA	NA	NA
0.18	1	NA	NA	NA	NA	NA	NA	NA	NA
0.17	1	NA	NA	NA	NA	NA	NA	NA	NA
0.18	1	NA	NA	NA	NA	NA	NA	NA	NA
0.20	1	NA	NA	NA	NA	NA	NA	NA	NA
0.16	1	NA	NA	NA	NA	NA	NA	NA	NA
0.18	1	NA	NA	NA	NA	NA	NA	NA	NA
0.17	1	NA	NA	NA	NA	NA	NA	NA	NA
0.15	1	NA	NA	NA	NA	NA	NA	NA	NA
0.17	1	NA	NA	NA	NA	NA	NA	NA	NA
4.16	1	NA	NA	NA	NA	NA	NA	NA	NA
0.24	1	NA	NA	NA	NA	NA	NA	NA	NA
0.15	1	NA	NA	NA	NA	NA	NA	NA	NA
0.26	1	NA	NA	NA	NA	NA	NA	NA	NA
0.23	1	NA	NA	NA	NA	NA	NA	NA	NA
0.21	1	NA	NA	NA	NA	NA	NA	NA	NA
0.21	1	NA	NA	NA	NA	NA	NA	NA	NA
0.18	1	NA	NA	NA	NA	NA	NA	NA	NA
0.18	1	NA	NA	NA	NA	NA	NA	NA	NA
0.19	1	NA	NA	NA	NA	NA	NA	NA	NA
0.33	1	NA	NA	NA	NA	NA	NA	NA	NA
0.20	1	NA	NA	NA	NA	NA	NA	NA	NA
11.09	1	NA	NA	NA	NA	NA	NA	NA	NA
0.24	1	NA	NA	NA	NA	NA	NA	NA	NA
0.19	1	NA	NA	NA	NA	NA	NA	NA	NA
0.17	1	NA	NA	NA	NA	NA	NA	NA	NA
0.13	1	NA	NA	NA	NA	NA	NA	NA	NA
0.22	1	NA	NA	NA	NA	NA	NA	NA	NA
0.18	1	NA	NA	NA	NA	NA	NA	NA	NA
0.33	1	NA	NA	NA	NA	NA	NA	NA	NA
0.17	1	NA	NA	NA	NA	NA	NA	NA	NA
0.20	1	NA	NA	NA	NA	NA	NA	NA	NA
0.38	1	NA	NA	NA	NA	NA	NA	NA	NA
0.26	1	NA	NA	NA	NA	NA	NA	NA	NA

time A	leuko A	time A	leuko A	time B	leuko B	time D	leuko D	time E	leuko E
0.91	1	NA	NA	NA	NA	NA	NA	NA	NA
0.24	1	NA	NA	NA	NA	NA	NA	NA	NA
0.29	1	NA	NA	NA	NA	NA	NA	NA	NA
0.22	1	NA	NA	NA	NA	NA	NA	NA	NA
0.17	1	NA	NA	NA	NA	NA	NA	NA	NA
0.24	1	NA	NA	NA	NA	NA	NA	NA	NA
0.28	1	NA	NA	NA	NA	NA	NA	NA	NA
0.25	1	NA	NA	NA	NA	NA	NA	NA	NA
0.23	1	NA	NA	NA	NA	NA	NA	NA	NA
0.38	1	NA	NA	NA	NA	NA	NA	NA	NA
0.22	1	NA	NA	NA	NA	NA	NA	NA	NA
0.18	1	NA	NA	NA	NA	NA	NA	NA	NA

1.9 SL057

Table 9: Raw data of TBS SL057.

time A	leuko A	time A	leuko A	time B	leuko B	time C	leuko C	time D	leuko D	time E	leuko E
0.00	1	0.00	1	0.00	1	0.00	1	0.00	1	0.00	1
0.30	1	0.26	1	0.38	1	0.25	1	0.20	1	0.24	1
0.27	1	0.23	1	0.28	1	0.23	1	0.24	1	0.31	1
0.25	1	0.21	1	0.27	1	1.21	1	0.19	1	0.28	1
0.23	1	0.24	1	0.56	1	0.21	1	0.22	1	0.32	1
0.22	1	0.21	1	0.30	1	0.39	1	0.18	1	0.28	1
0.20	1	0.21	1	0.28	1	0.22	1	0.25	1	0.27	1
0.21	1	3.96	0	0.28	1	0.09	1	0.21	1	0.29	1
0.22	1	4.43	1	0.79	1	0.13	1	0.19	1	0.29	1
2.50	0	0.25	1	0.29	1	0.21	1	0.21	1	0.28	1
0.28	0	0.24	1	0.26	1	0.10	1	0.21	1	0.27	1
0.21	0	0.73	0	0.24	1	0.12	1	0.23	1	41.33	0
0.20	0	0.22	0	0.36	1	0.20	1	0.21	1	0.24	0
0.21	0	0.14	0	0.27	1	0.22	1	0.24	1	0.27	0
0.21	0	0.28	0	4.93	1	0.35	1	0.41	1	0.50	0
0.19	0	0.24	0	0.28	1	0.31	1	0.21	1	0.67	0
0.21	0	0.20	0	0.23	1	0.20	1	0.26	1	0.25	0
0.20	0	0.22	0	0.25	1	0.22	1	0.45	1	0.21	0
0.16	0	0.18	0	0.33	1	0.08	1	0.20	1	0.67	0
0.22	0	0.54	0	0.26	1	0.12	1	0.19	1	0.26	0
0.23	0	0.28	0	0.21	1	0.31	1	0.26	1	0.25	0
0.22	0	0.23	0	0.24	1	1.11	0	0.23	1	0.24	0
0.20	0	3.04	1	0.31	1	0.31	0	0.26	1	0.21	0
0.19	0	0.22	1	0.25	1	0.29	0	0.22	1	0.19	0
0.19	0	0.21	1	0.25	1	0.34	0	1.77	0	1.55	0
0.17	0	0.19	1	0.24	1	0.22	0	0.24	0	0.28	0
0.20	0	0.20	1	0.26	1	0.26	0	0.82	0	0.37	0
0.18	0	0.20	1	0.21	1	0.34	0	0.21	0	0.27	0
0.18	0	0.17	1	0.26	1	0.27	0	0.28	0	0.60	0
0.18	0	0.54	0	0.23	1	0.29	0	0.73	0	0.23	0
0.20	0	0.24	0	0.21	1	0.20	0	0.77	0	0.60	0
0.21	0	0.17	0	8.13	0	0.22	0	0.46	0	0.26	0
0.20	0	0.20	0	0.35	0	0.37	0	0.29	0	0.27	0
0.20	0	0.16	0	0.28	0	0.24	0	0.32	0	0.30	0
0.19	0	0.20	0	0.65	0	1.56	0	0.94	0	0.25	0
0.18	0	0.18	0	0.31	0	0.24	0	0.24	0	0.21	0
0.18	0	0.18	0	0.37	0	0.28	0	0.26	0	0.24	0
0.19	0	0.17	0	0.66	0	0.21	0	0.27	0	0.23	0
0.19	0	0.19	0	0.33	0	0.25	0	0.44	0	0.23	0
0.20	0	0.19	0	0.28	0	0.24	0	0.19	0	0.39	0
0.19	0	0.18	0	0.26	0	0.27	0	0.23	0	0.24	0
0.16	0	0.40	0	0.75	0	0.30	0	0.30	0	0.71	0
0.19	0	0.25	0	0.35	0	0.34	0	0.24	0	0.27	0
0.19	0	2.02	1	0.29	0	0.23	0	0.48	0	0.24	0
0.17	0	0.23	1	0.26	0	0.23	0	0.22	0	0.67	0
0.20	0	0.21	1	0.27	0	0.24	0	0.30	0	0.24	0
0.17	0	0.19	1	0.33	0	0.23	0	0.22	0	0.21	0
0.19	0	0.20	1	0.27	0	0.22	0	0.23	0	0.22	0

time A	leuko A	time A	leuko A	time B	leuko B	time C	leuko C	time D	leuko D	time E	leuko E
0.17	0	0.37	1	0.27	0	0.24	0	0.35	0	0.20	0
0.19	0	0.19	1	0.48	0	0.25	0	0.29	0	0.41	0
0.20	0	0.20	1	0.33	0	0.27	0	0.66	0	0.21	0
0.18	0	0.17	1	0.32	0	0.35	0	0.39	0	0.46	0
0.20	0	0.18	1	1.39	0	0.34	0	0.18	0	0.90	0
0.18	0	0.17	1	0.33	0	0.23	0	0.26	0	0.71	0
0.19	0	0.17	1	0.29	0	0.33	0	0.21	0	0.23	0
0.22	0	0.19	1	0.29	0	0.21	0	0.29	0	2.31	0
0.21	0	0.19	1	0.29	0	0.23	0	0.22	0	0.26	0
0.20	0	0.16	1	0.28	0	0.32	0	0.31	0	0.20	0
0.22	0	0.37	0	0.26	0	0.25	0	0.27	0	0.61	0
0.19	0	0.25	0	0.27	0	0.23	0	0.21	0	0.23	0
0.21	0	0.28	0	0.32	0	0.21	0	0.22	0	0.24	0
0.18	0	0.21	0	0.27	0	0.23	0	0.44	0	0.25	0
0.19	0	0.16	0	0.28	0	0.33	0	0.44	0	0.23	0
0.17	0	0.19	0	0.26	0	0.26	0	0.44	0	0.23	0
0.19	0	0.17	0	0.25	0	0.24	0	0.22	0	0.22	0
0.16	0	0.22	0	0.26	0	0.46	0	0.18	0	0.21	0
0.18	0	0.19	0	0.25	0	0.56	0	0.27	0	1.87	0
0.22	0	0.17	0	0.28	0	0.22	0	0.21	0	0.24	0
0.18	0	0.17	0	0.26	0	0.23	0	0.23	0	0.26	0
0.20	0	0.19	0	0.22	0	0.25	0	0.20	0	0.23	0
0.22	0	0.17	0	0.35	0	0.23	0	0.20	0	0.25	0
0.21	0	0.20	0	0.28	0	0.25	0	0.21	0	0.23	0
0.20	0	0.17	0	0.26	0	0.23	0	0.19	0	0.21	0
0.21	0	0.21	0	0.26	0	0.22	0	0.19	0	0.47	0
0.19	0	0.18	0	0.60	0	0.39	0	0.21	0	0.79	0
0.20	0	0.14	0	0.28	0	1.05	0	0.49	0	0.26	0
0.21	0	0.26	0	0.28	0	0.20	0	0.20	0	1.16	0
0.18	0	0.19	0	0.26	0	0.61	0	0.22	0	0.25	0
0.17	0	0.21	0	0.27	0	0.27	0	0.22	0	0.21	0
0.19	0	0.18	0	0.58	0	0.26	0	0.22	0	0.24	0
0.20	0	0.19	0	0.28	0	0.26	0	0.20	0	0.59	0
0.16	0	0.19	0	0.27	0	0.37	0	0.21	0	0.26	0
0.18	0	0.19	0	0.25	0	0.25	0	0.25	0	0.50	0
0.19	0	0.19	0	0.32	0	0.28	0	0.23	0	0.48	0
0.19	0	0.18	0	0.25	0	0.29	0	0.19	0	0.27	0
0.20	0	0.20	0	0.26	0	0.39	0	0.21	0	0.20	0
0.19	0	0.19	0	0.25	0	0.23	0	0.23	0	2.56	0
0.22	0	0.18	0	0.31	0	0.26	0	0.19	0	0.25	0
0.21	0	0.15	0	0.27	0	0.22	0	0.22	0	0.20	0
0.20	0	0.16	0	0.28	0	0.28	0	0.19	0	0.24	0
0.19	0	0.17	0	0.26	0	0.25	0	0.21	0	0.18	0
5.75	1	0.20	0	0.25	0	0.35	0	0.20	0	0.21	0
0.31	1	0.20	0	0.31	0	0.36	0	0.22	0	0.22	0
0.23	1	0.16	0	0.24	0	0.39	0	0.15	0	0.33	0
0.20	1	0.17	0	0.30	0	0.20	0	0.23	0	0.24	0
0.20	1	0.20	0	0.25	0	0.22	0	0.20	0	0.24	0
0.21	1	0.18	0	0.27	0	0.26	0	0.21	0	0.44	0
0.21	1	0.15	0	0.24	0	0.24	0	0.34	0	1.03	0
0.21	1	0.19	0	0.31	0	0.43	0	0.22	0	0.26	0

time A	leuko A	time A	leuko A	time B	leuko B	time C	leuko C	time D	leuko D	time E	leuko E
0.19	1	0.16	0	0.29	0	0.32	0	0.20	0	0.58	0
0.19	1	0.18	0	0.31	0	0.23	0	0.22	0	0.26	0
0.19	1	0.18	0	0.28	0	0.22	0	0.21	0	0.23	0
0.18	1	0.17	0	0.28	0	0.35	0	0.24	0	0.29	0
0.19	1	0.19	0	0.26	0	0.68	0	0.21	0	0.26	0
0.21	1	0.15	0	0.26	0	1.11	0	0.20	0	0.61	0
0.69	0	0.20	0	0.31	0	0.28	0	0.20	0	0.20	0
0.24	0	0.18	0	0.26	0	0.29	0	0.19	0	0.23	0
0.22	0	0.18	0	0.25	0	0.28	0	0.21	0	0.22	0
0.20	0	0.19	0	0.25	0	0.26	0	0.35	0	0.26	0
0.28	0	0.18	0	0.26	0	0.24	0	0.23	0	2.07	0
0.28	0	0.20	0	0.34	0	0.27	0	0.19	0	0.22	0
0.24	0	0.18	0	0.24	0	0.27	0	0.21	0	2.60	0
0.18	0	0.19	0	0.25	0	0.41	0	0.23	0	0.29	0
0.23	0	0.18	0	0.25	0	0.29	0	0.21	0	0.22	0
0.18	0	0.18	0	0.28	0	0.51	0	0.19	0	0.21	0
0.21	0	0.20	0	0.28	0	0.33	0	0.20	0	0.21	0
0.16	0	0.18	0	0.27	0	0.27	0	0.23	0	0.50	0
0.21	0	0.14	0	0.26	0	0.29	0	0.21	0	0.26	0
0.20	0	0.26	0	0.25	0	0.28	0	0.22	0	0.83	0
0.19	0	0.24	0	0.27	0	0.41	0	0.21	0	0.28	0
0.20	0	0.18	0	0.27	0	0.31	0	0.19	0	0.16	0
0.20	0	0.19	0	0.26	0	0.27	0	0.21	0	0.26	0
0.19	0	0.18	0	0.27	0	0.25	0	0.18	0	0.33	0
0.18	0	0.19	0	0.24	0	0.25	0	0.21	0	0.22	0
0.19	0	0.18	0	0.31	0	0.26	0	0.23	0	0.34	0
0.19	0	0.16	0	0.26	0	0.27	0	0.22	0	2.68	0
0.20	0	0.20	0	0.25	0	0.22	0	0.19	0	0.29	0
0.20	0	0.18	0	0.25	0	0.36	0	0.20	0	0.73	0
0.17	0	0.20	0	0.29	0	0.39	0	0.17	0	0.23	0
0.21	0	0.18	0	0.25	0	0.27	0	0.22	0	0.21	0
0.19	0	0.19	0	0.25	0	0.31	0	0.19	0	0.87	0
0.18	0	0.21	0	0.26	0	0.28	0	0.21	0	0.23	0
0.19	0	0.19	0	2.27	0	0.30	0	0.19	0	0.20	0
0.20	0	0.22	0	0.28	0	0.31	0	0.21	0	0.39	0
0.22	0	0.20	0	0.27	0	0.48	0	0.20	0	0.24	0
0.22	0	0.20	0	0.93	0	0.24	0	0.20	0	0.19	0
0.20	0	0.20	0	0.28	0	0.23	0	0.19	0	0.21	0
0.19	0	0.17	0	0.28	0	0.30	0	0.21	0	0.26	0
0.21	0	0.20	0	0.26	0	0.29	0	0.21	0	107.22	1
0.19	0	0.20	0	0.32	0	0.27	0	0.22	0	0.01	1
11.83	1	0.23	0	0.26	0	0.25	0	0.18	0	0.26	1
0.27	1	0.18	0	0.28	0	0.24	0	0.20	0	0.27	1
0.18	1	0.24	0	0.26	0	0.25	0	0.21	0	0.27	1
0.15	1	0.31	0	0.35	0	0.27	0	0.19	0	0.26	1
0.17	1	0.24	0	0.24	0	0.23	0	0.20	0	0.28	1
0.16	1	0.22	0	0.28	0	0.26	0	0.19	0	0.27	1
0.18	1	0.25	0	0.26	0	0.27	0	0.21	0	4.32	0
0.17	1	5.18	1	0.31	0	0.23	0	0.19	0	0.52	0
0.17	1	0.25	1	0.28	0	0.27	0	0.22	0	0.52	0
0.17	1	0.19	1	0.25	0	0.27	0	0.21	0	0.22	0

time A	leuko A	time A	leuko A	time B	leuko B	time C	leuko C	time D	leuko D	time E	leuko E
0.19	1	0.18	1	0.24	0	0.21	0	0.20	0	0.60	0
0.16	1	0.20	1	0.29	0	0.22	0	0.20	0	0.26	0
0.19	1	0.19	1	0.26	0	0.23	0	0.20	0	0.22	0
0.17	1	0.19	1	0.27	0	0.46	0	0.16	0	0.24	0
0.51	0	0.20	1	0.26	0	0.46	0	0.20	0	0.22	0
0.43	0	0.18	1	0.30	0	0.51	0	0.20	0	0.20	0
0.52	0	0.20	1	0.26	0	0.37	0	0.20	0	0.22	0
0.22	0	0.20	1	0.27	0	0.25	0	0.19	0	0.20	0
0.52	0	0.18	1	0.26	0	0.97	0	0.19	0	0.22	0
0.23	0	0.17	1	0.29	0	0.28	0	0.21	0	0.22	0
0.40	0	0.16	1	0.28	0	0.29	0	0.16	0	0.20	0
0.21	0	0.21	1	0.23	0	1.21	0	0.20	0	0.20	0
0.22	0	0.20	1	0.28	0	6.12	1	0.18	0	0.66	0
0.19	0	2.43	0	0.25	0	0.09	1	0.18	0	0.23	0
0.22	0	0.27	0	0.26	0	0.13	1	0.20	0	0.23	0
0.22	0	0.20	0	0.28	0	0.22	1	0.21	0	1.34	0
0.23	0	0.22	0	0.28	0	0.31	1	0.19	0	0.25	0
6.47	1	0.24	0	0.27	0	0.23	1	0.19	0	0.19	0
0.31	1	0.22	0	0.25	0	0.43	1	0.51	0	0.23	0
0.23	1	0.18	0	0.27	0	0.32	1	0.20	0	0.24	0
0.22	1	0.20	0	0.26	0	0.45	1	0.23	0	0.27	0
0.20	1	0.23	0	0.27	0	0.24	1	0.49	0	0.20	0
0.20	1	0.23	0	0.28	0	0.21	1	0.21	0	0.20	0
0.21	1	0.23	0	0.27	0	0.22	1	0.32	0	0.58	0
0.20	1	0.25	0	0.26	0	0.23	1	0.54	0	0.24	0
0.20	1	0.19	0	0.26	0	0.24	1	0.27	0	1.13	0
0.21	1	0.21	0	0.26	0	0.27	1	0.34	0	0.27	0
0.72	0	0.30	0	0.29	0	0.19	1	0.25	0	0.23	0
0.21	0	0.24	0	0.26	0	0.22	1	0.27	0	0.20	0
0.19	0	0.20	0	0.27	0	0.21	1	0.24	0	1.08	0
0.22	0	0.20	0	0.25	0	0.23	1	0.25	0	0.25	0
0.22	0	0.19	0	0.30	0	0.20	1	0.24	0	0.21	0
0.18	0	0.21	0	0.27	0	0.22	1	0.23	0	0.22	0
0.18	0	0.18	0	0.26	0	0.21	1	0.24	0	0.94	0
0.18	0	0.20	0	0.25	0	0.22	1	0.22	0	0.25	0
0.18	0	0.21	0	0.29	0	0.40	1	0.21	0	0.38	0
0.21	0	0.17	0	0.27	0	0.22	1	0.20	0	0.24	0
0.23	0	0.21	0	0.26	0	0.22	1	0.22	0	0.20	0
0.22	0	0.20	0	0.30	0	0.24	1	0.22	0	0.19	0
0.19	0	0.21	0	0.25	0	0.23	1	0.21	0	0.58	0
0.20	0	0.21	0	0.25	0	3.34	0	0.22	0	0.29	0
0.21	0	0.20	0	0.33	0	0.31	0	0.22	0	0.46	0
0.20	0	0.19	0	0.26	0	0.29	0	0.20	0	0.24	0
0.20	0	0.20	0	0.27	0	0.57	0	0.19	0	0.26	0
0.22	0	0.32	0	0.25	0	0.30	0	0.23	0	0.81	0
0.20	0	0.52	0	0.28	0	0.36	0	0.22	0	0.23	0
0.20	0	0.29	0	0.27	0	0.26	0	0.20	0	0.31	0
0.31	0	0.29	0	0.26	0	0.25	0	0.19	0	0.23	0
0.20	0	0.20	0	0.24	0	0.23	0	0.22	0	0.29	0
0.21	0	0.19	0	0.30	0	0.43	0	0.20	0	0.43	0
0.20	0	0.20	0	0.23	0	0.24	0	0.20	0	0.26	0

time A	leuko A	time A	leuko A	time B	leuko B	time C	leuko C	time D	leuko D	time E	leuko E
0.21	0	0.19	0	0.25	0	0.24	0	0.19	0	0.32	0
0.19	0	0.19	0	0.32	0	0.21	0	0.21	0	0.32	0
0.20	0	0.20	0	0.26	0	0.28	0	0.21	0	0.24	0
0.21	0	0.20	0	0.24	0	0.25	0	0.18	0	0.48	0
0.20	0	0.20	0	0.32	0	0.24	0	0.21	0	0.65	0
0.19	0	0.23	0	0.22	0	0.27	0	0.19	0	0.25	0
0.20	0	0.23	0	0.27	0	0.22	0	0.22	0	0.25	0
0.20	0	0.19	0	0.42	0	0.27	0	0.17	0	0.31	0
0.20	0	0.23	0	0.31	0	0.59	0	0.20	0	0.22	0
0.22	0	0.20	0	0.25	0	0.26	0	0.21	0	0.20	0
0.22	0	0.21	0	2.22	0	0.23	0	0.23	0	0.22	0
0.19	0	0.17	0	0.26	0	0.24	0	0.19	0	0.37	0
0.31	0	0.23	0	0.22	0	0.28	0	0.20	0	0.22	0
0.22	0	0.21	0	1.18	0	0.22	0	0.18	0	0.24	0
0.23	0	0.17	0	0.21	0	0.25	0	0.20	0	0.26	0
0.20	0	0.10	0	0.27	0	0.22	0	0.21	0	1.01	0
0.19	0	0.22	0	0.36	0	0.50	0	0.20	0	0.30	0
4.58	1	0.21	0	0.28	0	0.27	0	0.20	0	0.45	0
0.26	1	0.20	0	0.25	0	0.23	0	0.18	0	0.29	0
0.21	1	0.22	0	0.48	0	0.32	0	0.18	0	0.35	0
0.21	1	0.21	0	0.27	0	0.31	0	0.20	0	0.37	0
0.20	1	0.23	0	0.24	0	0.24	0	0.20	0	0.35	0
0.19	1	0.20	0	0.39	0	0.26	0	0.16	0	1.33	0
0.20	1	0.21	0	0.25	0	0.23	0	0.17	0	0.22	0
0.18	1	0.18	0	0.26	0	0.25	0	0.25	0	0.88	0
0.19	1	0.23	0	0.23	0	0.33	0	0.24	0	0.21	0
0.18	1	0.25	0	7.07	0	0.62	0	0.21	0	0.58	0
0.16	1	0.22	0	0.25	0	0.23	0	0.20	0	0.25	0
0.18	1	0.20	0	0.25	0	0.23	0	0.19	0	0.20	0
0.19	1	0.20	0	3.19	0	0.38	0	0.17	0	0.24	0
0.18	1	0.22	0	0.26	0	0.24	0	0.19	0	0.39	0
0.21	1	0.19	0	0.25	0	0.23	0	0.21	0	0.64	0
0.18	1	0.20	0	0.32	0	0.41	0	0.18	0	0.23	0
0.18	1	0.20	0	0.24	0	0.27	0	0.15	0	0.25	0
0.20	1	0.20	0	0.26	0	0.28	0	0.18	0	0.21	0
3.44	0	0.19	0	0.23	0	0.32	0	0.27	0	0.38	0
0.98	0	0.19	0	0.31	0	0.25	0	0.21	0	0.21	0
0.25	0	0.21	0	0.22	0	0.23	0	0.22	0	0.20	0
0.22	0	0.21	0	0.24	0	0.25	0	0.20	0	0.20	0
0.20	0	0.20	0	0.24	0	0.23	0	0.22	0	0.24	0
0.19	0	0.18	0	0.23	0	0.22	0	0.20	0	0.32	0
0.20	0	0.19	0	NA	NA	0.22	0	0.21	0	0.37	0
0.18	0	0.22	0	NA	NA	0.24	0	0.18	0	1.06	0
0.18	0	0.16	0	NA	NA	0.28	0	0.20	0	0.21	0
0.22	0	10.33	0	NA	NA	0.28	0	0.20	0	0.19	0
0.16	0	7.17	1	NA	NA	0.35	0	0.19	0	0.41	0
0.21	0	0.26	1	NA	NA	0.30	0	0.20	0	0.22	0
0.19	0	0.18	1	NA	NA	0.27	0	0.21	0	0.22	0
0.19	0	0.21	1	NA	NA	0.31	0	0.19	0	0.21	0
0.19	0	0.27	1	NA	NA	0.24	0	0.22	0	0.22	0
0.21	0	0.20	1	NA	NA	0.22	0	0.20	0	0.25	0

time A	leuko A	time A	leuko A	time B	leuko B	time C	leuko C	time D	leuko D	time E	leuko E
0.18	0	0.15	1	NA	NA	0.21	0	0.21	0	0.35	0
0.19	0	0.17	1	NA	NA	0.22	0	0.20	0	0.24	0
0.22	0	0.15	1	NA	NA	0.22	0	0.21	0	0.28	0
0.20	0	0.10	1	NA	NA	0.42	0	0.21	0	0.24	0
0.21	0	0.21	1	NA	NA	0.29	0	0.21	0	0.22	0
0.20	0	0.11	1	NA	NA	0.38	0	0.23	0	0.22	0
0.20	0	0.17	1	NA	NA	0.30	0	0.23	0	1.30	0
0.20	0	0.13	1	NA	NA	0.32	0	0.20	0	0.30	0
0.21	0	0.16	1	NA	NA	0.26	0	0.22	0	0.21	0
0.18	0	0.12	1	NA	NA	0.63	0	0.19	0	0.24	0
0.19	0	0.17	1	NA	NA	0.22	0	0.21	0	NA	NA
0.19	0	0.19	1	NA	NA	0.29	0	0.22	0	NA	NA
0.21	0	0.88	0	NA	NA	0.26	0	0.22	0	NA	NA
0.20	0	0.24	0	NA	NA	0.24	0	0.19	0	NA	NA
0.20	0	0.18	0	NA	NA	0.31	0	0.72	0	NA	NA
0.21	0	1.53	0	NA	NA	0.28	0	0.22	0	NA	NA
0.21	0	1.14	0	NA	NA	0.23	0	0.24	0	NA	NA
0.19	0	0.23	0	NA	NA	0.25	0	0.23	0	NA	NA
0.21	0	0.21	0	NA	NA	0.24	0	0.23	0	NA	NA
0.18	0	0.16	0	NA	NA	0.30	0	0.20	0	NA	NA
0.20	0	0.19	0	NA	NA	0.25	0	0.22	0	NA	NA
0.18	0	0.17	0	NA	NA	0.58	0	0.20	0	NA	NA
0.20	0	0.30	0	NA	NA	0.30	0	0.23	0	NA	NA
0.21	0	0.17	0	NA	NA	0.27	0	0.22	0	NA	NA
0.20	0	0.17	0	NA	NA	0.61	0	0.22	0	NA	NA
0.23	0	0.19	0	NA	NA	0.24	0	0.22	0	NA	NA
0.22	0	0.21	0	NA	NA	0.28	0	0.22	0	NA	NA
0.21	0	0.18	0	NA	NA	0.23	0	0.22	0	NA	NA
0.23	0	0.19	0	NA	NA	0.26	0	0.21	0	NA	NA
0.19	0	0.19	0	NA	NA	0.21	0	0.22	0	NA	NA
0.22	0	0.16	0	NA	NA	0.19	0	0.22	0	NA	NA
0.20	0	0.18	0	NA	NA	0.29	0	0.24	0	NA	NA
0.19	0	0.26	0	NA	NA	0.29	0	0.22	0	NA	NA
0.19	0	0.23	0	NA	NA	0.23	0	0.19	0	NA	NA
0.21	0	0.18	0	NA	NA	0.23	0	0.20	0	NA	NA
0.21	0	0.19	0	NA	NA	0.24	0	0.21	0	NA	NA
0.21	0	0.19	0	NA	NA	0.25	0	0.22	0	NA	NA
0.23	0	0.19	0	NA	NA	0.27	0	0.20	0	NA	NA
0.21	0	0.21	0	NA	NA	0.29	0	0.19	0	NA	NA
0.21	0	0.18	0	NA	NA	0.65	0	0.20	0	NA	NA
0.22	0	0.19	0	NA	NA	0.24	0	0.20	0	NA	NA
0.23	0	0.19	0	NA	NA	0.24	0	0.21	0	NA	NA
NA	NA	0.20	0	NA	NA	0.23	0	0.21	0	NA	NA
NA	NA	0.18	0	NA	NA	0.26	0	0.21	0	NA	NA
NA	NA	0.20	0	NA	NA	0.26	0	0.35	0	NA	NA
NA	NA	0.21	0	NA	NA	0.28	0	0.20	0	NA	NA
NA	NA	0.22	0	NA	NA	0.26	0	0.20	0	NA	NA
NA	NA	0.18	0	NA	NA	0.23	0	0.19	0	NA	NA
NA	NA	0.19	0	NA	NA	0.26	0	0.20	0	NA	NA
NA	NA	0.19	0	NA	NA	0.23	0	0.20	0	NA	NA
NA	NA	0.20	0	NA	NA	0.22	0	0.20	0	NA	NA

time A	leuko A	time A	leuko A	time B	leuko B	time C	leuko C	time D	leuko D	time E	leuko E
NA	NA	0.19	0	NA	NA	0.26	0	0.21	0	NA	NA
NA	NA	NA	NA	NA	NA	0.40	0	0.18	0	NA	NA
NA	NA	NA	NA	NA	NA	0.21	0	0.16	0	NA	NA
NA	NA	NA	NA	NA	NA	0.22	0	0.19	0	NA	NA
NA	NA	NA	NA	NA	NA	0.21	0	0.21	0	NA	NA
NA	NA	NA	NA	NA	NA	0.23	0	0.20	0	NA	NA
NA	NA	NA	NA	NA	NA	0.35	0	0.18	0	NA	NA
NA	NA	NA	NA	NA	NA	0.26	0	0.18	0	NA	NA
NA	NA	NA	NA	NA	NA	0.33	0	0.20	0	NA	NA
NA	NA	NA	NA	NA	NA	0.22	0	0.18	0	NA	NA
NA	NA	NA	NA	NA	NA	0.23	0	0.21	0	NA	NA
NA	NA	NA	NA	NA	NA	0.25	0	0.20	0	NA	NA
NA	NA	NA	NA	NA	NA	0.39	0	0.20	0	NA	NA
NA	NA	NA	NA	NA	NA	0.19	0	0.20	0	NA	NA
NA	NA	NA	NA	NA	NA	0.21	0	0.21	0	NA	NA
NA	NA	NA	NA	NA	NA	0.24	0	0.18	0	NA	NA
NA	NA	NA	NA	NA	NA	0.24	0	0.20	0	NA	NA
NA	NA	NA	NA	NA	NA	0.21	0	0.20	0	NA	NA
NA	NA	NA	NA	NA	NA	0.24	0	0.21	0	NA	NA
NA	NA	NA	NA	NA	NA	0.21	0	0.22	0	NA	NA
NA	NA	NA	NA	NA	NA	0.22	0	0.20	0	NA	NA
NA	NA	NA	NA	NA	NA	0.30	0	0.20	0	NA	NA
NA	NA	NA	NA	NA	NA	0.21	0	0.20	0	NA	NA
NA	NA	NA	NA	NA	NA	0.22	0	0.21	0	NA	NA
NA	NA	NA	NA	NA	NA	0.27	0	0.21	0	NA	NA
NA	NA	NA	NA	NA	NA	0.29	0	0.18	0	NA	NA
NA	NA	NA	NA	NA	NA	0.31	0	0.22	0	NA	NA
NA	NA	NA	NA	NA	NA	0.23	0	0.19	0	NA	NA
NA	NA	NA	NA	NA	NA	0.28	0	0.19	0	NA	NA
NA	NA	NA	NA	NA	NA	0.26	0	0.20	0	NA	NA
NA	NA	NA	NA	NA	NA	0.42	0	0.21	0	NA	NA
NA	NA	NA	NA	NA	NA	0.25	0	0.21	0	NA	NA
NA	NA	NA	NA	NA	NA	0.21	0	0.20	0	NA	NA
NA	NA	NA	NA	NA	NA	0.23	0	0.20	0	NA	NA
NA	NA	NA	NA	NA	NA	0.23	0	0.20	0	NA	NA
NA	NA	NA	NA	NA	NA	0.32	0	0.18	0	NA	NA
NA	NA	NA	NA	NA	NA	0.21	0	0.20	0	NA	NA
NA	NA	NA	NA	NA	NA	0.21	0	0.19	0	NA	NA
NA	NA	NA	NA	NA	NA	0.33	0	0.20	0	NA	NA
NA	NA	NA	NA	NA	NA	0.44	0	0.19	0	NA	NA
NA	NA	NA	NA	NA	NA	0.21	0	0.20	0	NA	NA
NA	NA	NA	NA	NA	NA	0.25	0	0.17	0	NA	NA
NA	NA	NA	NA	NA	NA	0.22	0	0.19	0	NA	NA
NA	NA	NA	NA	NA	NA	0.24	0	0.20	0	NA	NA
NA	NA	NA	NA	NA	NA	0.24	0	0.21	0	NA	NA
NA	NA	NA	NA	NA	NA	0.27	0	0.20	0	NA	NA
NA	NA	NA	NA	NA	NA	0.24	0	0.20	0	NA	NA
NA	NA	NA	NA	NA	NA	0.24	0	0.22	0	NA	NA
NA	NA	NA	NA	NA	NA	0.43	0	0.16	0	NA	NA
NA	NA	NA	NA	NA	NA	0.34	0	0.20	0	NA	NA
NA	NA	NA	NA	NA	NA	0.21	0	0.20	0	NA	NA

time A	leuko A	time A	leuko A	time B	leuko B	time C	leuko C	time D	leuko D	time E	leuko E
NA	NA	NA	NA	NA	NA	0.23	0	0.19	0	NA	NA
NA	NA	NA	NA	NA	NA	0.42	0	0.21	0	NA	NA
NA	NA	NA	NA	NA	NA	0.22	0	0.18	0	NA	NA
NA	NA	NA	NA	NA	NA	0.28	0	0.17	0	NA	NA
NA	NA	NA	NA	NA	NA	0.29	0	0.19	0	NA	NA
NA	NA	NA	NA	NA	NA	0.23	0	0.18	0	NA	NA
NA	NA	NA	NA	NA	NA	0.28	0	0.17	0	NA	NA
NA	NA	NA	NA	NA	NA	0.21	0	0.18	0	NA	NA
NA	NA	NA	NA	NA	NA	0.24	0	0.18	0	NA	NA
NA	NA	NA	NA	NA	NA	0.23	0	0.20	0	NA	NA
NA	NA	NA	NA	NA	NA	0.36	0	0.15	0	NA	NA
NA	NA	NA	NA	NA	NA	0.21	0	0.21	0	NA	NA
NA	NA	NA	NA	NA	NA	0.30	0	0.19	0	NA	NA
NA	NA	NA	NA	NA	NA	0.25	0	0.21	0	NA	NA
NA	NA	NA	NA	NA	NA	0.39	0	0.20	0	NA	NA
NA	NA	NA	NA	NA	NA	0.20	0	0.19	0	NA	NA
NA	NA	NA	NA	NA	NA	0.35	0	0.19	0	NA	NA
NA	NA	NA	NA	NA	NA	0.35	0	0.20	0	NA	NA
NA	NA	NA	NA	NA	NA	0.48	0	0.19	0	NA	NA
NA	NA	NA	NA	NA	NA	0.26	0	0.20	0	NA	NA
NA	NA	NA	NA	NA	NA	0.28	0	0.20	0	NA	NA
NA	NA	NA	NA	NA	NA	0.26	0	0.21	0	NA	NA
NA	NA	NA	NA	NA	NA	0.28	0	0.21	0	NA	NA
NA	NA	NA	NA	NA	NA	0.25	0	0.20	0	NA	NA
NA	NA	NA	NA	NA	NA	0.24	0	0.21	0	NA	NA
NA	NA	NA	NA	NA	NA	0.34	0	0.16	0	NA	NA
NA	NA	NA	NA	NA	NA	0.23	0	0.26	0	NA	NA
NA	NA	NA	NA	NA	NA	0.30	0	0.22	0	NA	NA
NA	NA	NA	NA	NA	NA	0.28	0	0.22	0	NA	NA
NA	NA	NA	NA	NA	NA	0.22	0	0.20	0	NA	NA
NA	NA	NA	NA	NA	NA	0.30	0	0.23	0	NA	NA
NA	NA	NA	NA	NA	NA	0.24	0	0.22	0	NA	NA
NA	NA	NA	NA	NA	NA	0.24	0	0.19	0	NA	NA
NA	NA	NA	NA	NA	NA	0.26	0	0.22	0	NA	NA
NA	NA	NA	NA	NA	NA	0.28	0	0.22	0	NA	NA
NA	NA	NA	NA	NA	NA	0.27	0	0.23	0	NA	NA
NA	NA	NA	NA	NA	NA	0.39	0	0.22	0	NA	NA
NA	NA	NA	NA	NA	NA	0.26	0	0.21	0	NA	NA
NA	NA	NA	NA	NA	NA	0.23	0	0.20	0	NA	NA
NA	NA	NA	NA	NA	NA	0.32	0	0.19	0	NA	NA
NA	NA	NA	NA	NA	NA	0.22	0	0.21	0	NA	NA
NA	NA	NA	NA	NA	NA	0.27	0	0.21	0	NA	NA
NA	NA	NA	NA	NA	NA	0.31	0	0.21	0	NA	NA
NA	NA	NA	NA	NA	NA	0.19	0	0.19	0	NA	NA
NA	NA	NA	NA	NA	NA	0.30	0	0.21	0	NA	NA
NA	NA	NA	NA	NA	NA	0.33	0	0.20	0	NA	NA
NA	NA	NA	NA	NA	NA	0.22	0	0.16	0	NA	NA
NA	NA	NA	NA	NA	NA	0.23	0	0.20	0	NA	NA
NA	NA	NA	NA	NA	NA	0.37	0	0.20	0	NA	NA
NA	NA	NA	NA	NA	NA	0.29	0	0.20	0	NA	NA
NA	NA	NA	NA	NA	NA	0.24	0	NA	NA	NA	NA

time A	leuko A	time A	leuko A	time B	leuko B	time C	leuko C	time D	leuko D	time E	leuko E
NA	NA	NA	NA	NA	NA	0.25	0	NA	NA	NA	NA
NA	NA	NA	NA	NA	NA	0.27	0	NA	NA	NA	NA
NA	NA	NA	NA	NA	NA	0.24	0	NA	NA	NA	NA
NA	NA	NA	NA	NA	NA	0.37	0	NA	NA	NA	NA
NA	NA	NA	NA	NA	NA	1.05	0	NA	NA	NA	NA
NA	NA	NA	NA	NA	NA	0.24	0	NA	NA	NA	NA

2 Leukocyte and parasite counts per HPFs

We present here the result of the (calibrated) HPFs calling algorithm. For each HPF, the leukocyte and parasite counts are given.

2.1 A094

Table 10: leuko/parasite data per HPF for the six readings of TBS A094.

	leuko B	para B	leuko D	para D
HPF1	0	0	0	0
HPF2	10	0	9	0
HPF3	8	0	6	0
HPF4	7	0	8	0
HPF5	7	0	10	0
HPF6	5	0	9	0
HPF7	7	0	8	0
HPF8	8	0	9	0
HPF9	7	0	1	0
HPF10	10	0	9	0
HPF11	7	0	8	0
HPF12	7	0	20	0
HPF13	7	0	8	0
HPF14	7	0	7	0
HPF15	7	0	8	0
HPF16	6	0	9	0
HPF17	6	0	10	0
HPF18	8	0	11	0
HPF19	6	0	13	0
HPF20	6	0	10	0
HPF21	6	0	8	0
HPF22	8	0	16	0
HPF23	8	0	9	0
HPF24	6	0	8	0
HPF25	6	0	5	0
HPF26	7	0	8	0
HPF27	8	0	5	0
HPF28	8	0	10	0
HPF29	9	0	9	0
HPF30	6	0	9	0
HPF31	9	0	10	0
HPF32	8	0	10	0
HPF33	7	0	10	0
HPF34	2	0	10	0
HPF35	7	0	12	0
HPF36	9	0	12	0
HPF37	15	0	7	0
HPF38	11	0	7	0
HPF39	6	0	6	0
HPF40	15	0	9	0
HPF41	12	0	13	0
HPF42	10	0	16	0
HPF43	16	0	7	0
HPF44	17	0	9	0
HPF45	11	0	6	0
HPF46	9	0	7	0
HPF47	6	0	15	0
HPF48	7	0	21	0

	leuko B	para B	leuko D	para D
HPF49	7	0	4	0
HPF50	5	0	8	0
HPF51	7	0	4	0
HPF52	6	0	9	0
HPF53	7	0	13	0
HPF54	7	0	12	0
HPF55	7	0	5	0
HPF56	7	0	NA	NA
HPF57	7	0	NA	NA
HPF58	7	0	NA	NA
HPF59	8	0	NA	NA
HPF60	8	0	NA	NA
HPF61	8	0	NA	NA
HPF62	7	0	NA	NA
HPF63	1	0	NA	NA
HPF64	7	0	NA	NA
HPF65	7	0	NA	NA
HPF66	5	0	NA	NA
HPF67	3	0	NA	NA
	506	0	502	0

2.2 A098

Table 11: leuko/parasite data per HPF for the six readings of TBS A098.

	leuko A	para A	leuko A	para A	leuko B	para B	leuko C	para C	leuko D	para D	leuko E	para E
HPF1	0	0	0	2	0	1	0	0	0	0	0	0
HPF2	7	0	6	0	6	0	8	0	5	0	6	0
HPF3	7	0	9	0	8	0	9	0	8	0	6	0
HPF4	6	0	10	0	10	0	8	0	6	4	2	0
HPF5	6	0	11	0	7	0	10	0	7	0	6	0
HPF6	7	0	9	0	6	1	8	0	9	0	6	1
HPF7	6	0	8	0	9	1	7	0	13	1	2	1
HPF8	6	0	6	0	9	0	6	0	6	0	10	0
HPF9	6	0	5	0	8	1	9	0	12	0	7	0
HPF10	6	0	7	0	8	0	7	0	7	0	7	2
HPF11	9	0	8	0	8	0	10	0	8	0	9	0
HPF12	7	0	9	0	5	0	12	0	11	2	8	1
HPF13	7	0	5	0	10	0	10	0	7	0	4	1
HPF14	7	0	9	0	8	0	5	0	6	1	2	1
HPF15	8	0	8	0	6	0	9	0	2	1	11	0
HPF16	8	0	13	0	7	0	9	0	8	0	7	1
HPF17	1	0	7	1	6	0	5	0	7	0	8	0
HPF18	8	1	7	0	18	1	6	0	8	1	5	1
HPF19	7	0	8	0	7	0	7	0	8	0	11	1
HPF20	5	3	8	1	6	2	7	0	4	0	3	1
HPF21	8	0	5	1	10	0	9	0	10	0	7	0
HPF22	8	0	10	0	6	0	4	0	16	2	9	0
HPF23	8	1	8	0	7	1	9	0	8	0	9	0
HPF24	7	0	10	0	9	0	8	0	11	1	7	0
HPF25	6	0	5	1	13	0	8	0	9	0	5	1
HPF26	10	3	3	1	8	0	8	0	7	1	4	1
HPF27	10	1	2	2	6	0	8	0	4	1	3	1
HPF28	8	0	5	2	6	1	7	0	10	0	7	0
HPF29	5	1	6	0	8	0	6	0	NA	NA	5	0
HPF30	8	1	NA	NA	6	0	8	0	NA	NA	7	1
HPF31	NA	NA	NA	NA	8	0	8	0	NA	NA	7	0
HPF32	NA	NA	NA	NA	8	0	9	0	NA	NA	6	0
HPF33	NA	NA	NA	NA	6	0	8	0	NA	NA	7	1
HPF34	NA	NA	NA	NA	6	1	7	0	NA	NA	NA	NA
HPF35	NA	NA	NA	NA	1	1	9	0	NA	NA	NA	NA
HPF36	NA	NA	NA	NA	8	0	16	0	NA	NA	NA	NA
HPF37	NA	NA	NA	NA	4	0	10	0	NA	NA	NA	NA
HPF38	NA	NA	NA	NA	9	0	10	0	NA	NA	NA	NA
HPF39	NA	NA	NA	NA	9	0	17	0	NA	NA	NA	NA
HPF40	NA	NA	NA	NA	7	0	10	0	NA	NA	NA	NA
HPF41	NA	NA	NA	NA	9	0	9	0	NA	NA	NA	NA
HPF42	NA	NA	NA	NA	6	1	10	0	NA	NA	NA	NA
HPF43	NA	NA	NA	NA	8	0	7	0	NA	NA	NA	NA
HPF44	NA	NA	NA	NA	8	0	10	0	NA	NA	NA	NA
HPF45	NA	NA	NA	NA	6	0	17	0	NA	NA	NA	NA
HPF46	NA	NA	NA	NA	4	0	14	0	NA	NA	NA	NA
HPF47	NA	NA	NA	NA	6	0	13	0	NA	NA	NA	NA

	leuko A	para A	leuko A	para A	leuko B	para B	leuko C	para C	leuko D	para D	leuko E	para E
HPF48	NA	NA	NA	NA	9	0	12	0	NA	NA	NA	NA
HPF49	NA	NA	NA	NA	6	0	6	0	NA	NA	NA	NA
HPF50	NA	NA	NA	NA	9	0	6	0	NA	NA	NA	NA
HPF51	NA	NA	NA	NA	5	1	9	0	NA	NA	NA	NA
HPF52	NA	NA	NA	NA	3	1	22	0	NA	NA	NA	NA
HPF53	NA	NA	NA	NA	7	0	17	0	NA	NA	NA	NA
HPF54	NA	NA	NA	NA	8	0	14	0	NA	NA	NA	NA
HPF55	NA	NA	NA	NA	9	0	9	0	NA	NA	NA	NA
HPF56	NA	NA	NA	NA	5	3	5	0	NA	NA	NA	NA
HPF57	NA	NA	NA	NA	9	0	7	0	NA	NA	NA	NA
HPF58	NA	NA	NA	NA	5	0	7	0	NA	NA	NA	NA
HPF59	NA	NA	NA	NA	11	0	10	0	NA	NA	NA	NA
HPF60	NA	NA	NA	NA	5	0	6	0	NA	NA	NA	NA
HPF61	NA	NA	NA	NA	11	2	NA	NA	NA	NA	NA	NA
HPF62	NA	NA	NA	NA	11	0	NA	NA	NA	NA	NA	NA
HPF63	NA	NA	NA	NA	9	0	NA	NA	NA	NA	NA	NA
HPF64	NA	NA	NA	NA	15	0	NA	NA	NA	NA	NA	NA
HPF65	NA	NA	NA	NA	9	0	NA	NA	NA	NA	NA	NA
HPF66	NA	NA	NA	NA	6	1	NA	NA	NA	NA	NA	NA
HPF67	NA	NA	NA	NA	9	0	NA	NA	NA	NA	NA	NA
HPF68	NA	NA	NA	NA	8	1	NA	NA	NA	NA	NA	NA
	202	11	207	11	513	21	541	0	217	15	203	16

2.3 A100

Table 12: leuko/parasite data per HPF for the six readings of TBS A100.

	leuko A	para A	leuko A	para A	leuko B	para B	leuko C	para C	leuko D	para D	leuko E	para E
HPF1	0	0	0	0	0	0	0	0	0	0	0	0
HPF2	10	0	11	0	7	0	5	0	14	0	5	0
HPF3	6	0	9	0	7	0	9	0	8	0	6	0
HPF4	11	0	9	0	16	0	13	0	9	0	10	0
HPF5	17	0	11	0	8	0	20	0	13	0	7	0
HPF6	12	0	14	0	7	0	20	0	8	0	14	0
HPF7	17	0	17	0	11	0	19	0	19	0	5	0
HPF8	14	0	16	0	17	0	16	0	6	0	12	0
HPF9	27	0	13	0	18	0	17	0	6	0	9	0
HPF10	22	0	19	0	8	0	18	0	6	0	11	0
HPF11	19	0	21	0	12	0	22	0	6	0	11	0
HPF12	19	0	14	0	16	0	17	0	26	0	11	0
HPF13	18	0	14	0	7	0	13	0	10	0	8	0
HPF14	7	0	14	0	6	0	10	0	10	0	10	0
HPF15	17	0	16	0	10	0	6	0	8	0	11	0
HPF16	12	0	14	0	11	0	7	0	17	0	5	0
HPF17	16	0	14	0	14	0	19	0	24	0	10	0
HPF18	8	0	18	0	9	0	18	0	17	0	9	0
HPF19	15	0	12	0	9	0	18	0	42	0	7	0
HPF20	15	0	7	0	16	0	17	0	13	0	11	0
HPF21	13	0	13	0	15	0	7	0	4	0	12	0
HPF22	9	0	14	0	14	0	6	0	8	0	11	0
HPF23	13	0	17	0	14	0	6	0	28	0	16	0
HPF24	15	0	15	0	5	0	6	0	19	0	11	0
HPF25	13	0	10	0	14	0	10	0	19	0	11	0
HPF26	11	0	8	0	9	0	10	0	16	0	10	0
HPF27	16	0	10	0	7	0	16	0	33	0	9	0
HPF28	20	0	5	0	7	0	13	0	18	0	16	0
HPF29	19	0	7	0	14	0	18	0	31	0	12	0
HPF30	13	0	14	0	7	0	9	0	24	0	10	0
HPF31	12	0	11	0	13	0	16	0	24	0	19	0
HPF32	20	0	11	0	4	0	20	0	13	0	15	0
HPF33	13	0	10	0	21	0	9	0	28	0	17	0
HPF34	15	0	11	0	14	0	14	0	NA	NA	13	0
HPF35	17	0	14	0	11	0	19	0	NA	NA	21	0
HPF36	13	0	12	0	9	0	7	0	NA	NA	13	0
HPF37	NA	NA	10	0	6	0	17	0	NA	NA	16	0
HPF38	NA	NA	16	0	10	0	8	0	NA	NA	8	0
HPF39	NA	NA	14	0	15	0	15	0	NA	NA	8	0
HPF40	NA	NA	11	0	7	0	15	0	NA	NA	13	0
HPF41	NA	NA	19	0	10	0	7	0	NA	NA	9	0
HPF42	NA	NA	NA	NA	12	0	10	0	NA	NA	10	0
HPF43	NA	NA	NA	NA	19	0	17	0	NA	NA	7	0
HPF44	NA	NA	NA	NA	10	0	10	0	NA	NA	12	0
HPF45	NA	NA	NA	NA	19	0	23	0	NA	NA	7	0
HPF46	NA	NA	NA	NA	16	0	25	0	NA	NA	9	0
HPF47	NA	NA	NA	NA	NA	NA	NA	NA	NA	NA	11	0

	leuko A	para A	leuko A	para A	leuko B	para B	leuko C	para C	leuko D	para D	leuko E	para E
HPF48	NA	NA	NA	NA	NA	NA	NA	NA	NA	NA	9	0
	514	0	515	0	511	0	617	0	527	0	507	0

2.4 OPT211

Table 13: leuko/parasite data per HPF for the six readings of TBS OPT211.

	leuko A	para A	leuko A	para A	leuko B	para B	leuko C	para C	leuko D	para D	leuko E	para E
HPF1	0	292	0	0	0	0	0	210	0	386	0	0
HPF2	5	0	3	180	12	324	10	398	7	0	4	318
HPF3	NA	NA	5	230	NA	NA	NA	NA	5	51	NA	NA
HPF4	NA	NA	NA	NA	NA	NA	NA	NA	2	25	NA	NA
HPF5	NA	NA	NA	NA	NA	NA	NA	NA	1	15	NA	NA
HPF6	NA	NA	NA	NA	NA	NA	NA	NA	1	21	NA	NA
HPF7	NA	NA	NA	NA	NA	NA	NA	NA	1	13	NA	NA
HPF8	NA	NA	NA	NA	NA	NA	NA	NA	2	5	NA	NA
HPF9	NA	NA	NA	NA	NA	NA	NA	NA	1	19	NA	NA
HPF10	NA	NA	NA	NA	NA	NA	NA	NA	1	6	NA	NA
HPF11	NA	NA	NA	NA	NA	NA	NA	NA	1	20	NA	NA
HPF12	NA	NA	NA	NA	NA	NA	NA	NA	1	15	NA	NA
HPF13	NA	NA	NA	NA	NA	NA	NA	NA	1	4	NA	NA
	5	292	8	410	12	324	10	608	24	580	4	318

2.5 OPT257

Table 14: leuko/parasite data per HPF for the six readings of TBS OPT257.

	leuko A	para A	leuko A	para A	leuko B	para B	leuko C	para C	leuko D	para D	leuko E	para E
HPF1	0	0	0	0	0	0	0	0	0	0	0	0
HPF2	8	0	12	0	10	0	19	1	10	0	6	0
HPF3	8	0	15	0	5	0	12	0	10	0	6	0
HPF4	9	0	11	0	13	0	24	0	10	0	10	0
HPF5	14	0	7	0	7	0	22	0	8	0	10	0
HPF6	17	0	14	0	7	0	15	1	8	0	8	0
HPF7	9	0	11	0	10	0	10	0	9	0	11	0
HPF8	11	0	14	0	14	0	8	0	15	0	14	0
HPF9	16	0	19	0	7	0	32	0	9	0	12	0
HPF10	17	0	15	0	7	0	11	0	10	0	7	0
HPF11	16	0	20	0	17	0	8	0	8	0	10	0
HPF12	19	0	16	0	12	0	12	0	4	0	8	0
HPF13	9	0	11	0	13	0	13	0	12	0	7	0
HPF14	10	0	18	0	13	0	10	0	6	0	12	0
HPF15	10	1	14	0	9	0	10	0	10	0	13	0
HPF16	3	0	16	0	13	0	23	0	10	0	15	0
HPF17	15	0	18	0	10	0	24	0	11	0	10	0
HPF18	19	0	20	0	8	0	32	0	10	0	7	0
HPF19	11	0	13	0	11	0	10	0	7	0	10	0
HPF20	15	0	18	0	4	0	9	0	19	0	10	0
HPF21	13	0	8	0	9	0	9	0	22	0	10	0
HPF22	17	0	15	0	8	0	4	0	15	0	7	0
HPF23	22	0	16	0	5	0	18	0	17	0	19	0
HPF24	15	0	16	0	17	0	11	0	11	0	10	0
HPF25	19	0	14	0	18	0	6	0	11	0	11	0
HPF26	14	0	17	0	9	0	11	0	8	0	9	0
HPF27	18	0	12	0	12	0	16	0	7	0	10	0
HPF28	10	0	19	0	12	0	17	0	9	0	14	0
HPF29	20	0	20	0	9	0	8	0	29	0	5	0
HPF30	14	0	12	0	22	0	7	0	7	0	8	0
HPF31	19	0	16	0	8	0	17	0	5	0	14	0
HPF32	11	0	11	0	13	0	17	0	22	0	10	0
HPF33	16	0	8	0	10	0	16	0	31	0	4	0
HPF34	13	0	8	0	7	0	4	0	31	0	8	0
HPF35	25	0	11	0	14	0	17	0	6	0	15	0
HPF36	13	0	9	0	9	0	15	0	32	0	13	0
HPF37	13	0	13	0	9	0	15	0	5	0	14	0
HPF38	11	0	NA	NA	14	0	2	0	7	0	13	0
HPF39	NA	NA	NA	NA	14	0	NA	NA	24	0	4	0
HPF40	NA	NA	NA	NA	15	0	NA	NA	12	0	8	0
HPF41	NA	NA	NA	NA	24	0	NA	NA	5	0	11	0
HPF42	NA	NA	NA	NA	8	0	NA	NA	16	0	10	0
HPF43	NA	NA	NA	NA	10	0	NA	NA	6	0	11	0
HPF44	NA	NA	NA	NA	6	0	NA	NA	NA	NA	7	0
HPF45	NA	NA	NA	NA	10	0	NA	NA	NA	NA	9	0
HPF46	NA	NA	NA	NA	19	0	NA	NA	NA	NA	10	0
HPF47	NA	NA	NA	NA	NA	NA	NA	NA	NA	NA	7	0

	leuko A	para A	leuko A	para A	leuko B	para B	leuko C	para C	leuko D	para D	leuko E	para E
HPF48	NA	NA	NA	NA	NA	NA	NA	NA	NA	NA	6	0
HPF49	NA	NA	NA	NA	NA	NA	NA	NA	NA	NA	10	0
HPF50	NA	NA	NA	NA	NA	NA	NA	NA	NA	NA	6	0
HPF51	NA	NA	NA	NA	NA	NA	NA	NA	NA	NA	10	0
HPF52	NA	NA	NA	NA	NA	NA	NA	NA	NA	NA	7	0
HPF53	NA	NA	NA	NA	NA	NA	NA	NA	NA	NA	9	0
HPF54	NA	NA	NA	NA	NA	NA	NA	NA	NA	NA	8	0
	519	1	507	0	501	0	514	2	524	0	513	0

2.6 OPT271

Table 15: leuko/parasite data per HPF for the six readings of TBS OPT271.

	leuko A	para A	leuko A	para A	leuko B	para B	leuko C	para C	leuko D	para D	leuko E	para E
HPF1	0	0	0	0	0	3	0	0	0	0	0	0
HPF2	13	0	7	0	6	0	8	0	14	11	6	4
HPF3	6	0	1	0	5	0	9	0	19	7	2	1
HPF4	6	0	7	0	9	1	14	0	13	12	7	4
HPF5	7	0	8	0	4	2	9	0	11	13	2	1
HPF6	13	0	10	0	9	4	9	0	13	6	9	1
HPF7	6	3	5	1	6	0	5	0	15	15	8	0
HPF8	9	3	7	0	8	11	7	0	18	10	4	2
HPF9	2	1	9	0	6	4	21	0	22	18	4	5
HPF10	8	2	5	0	8	6	12	0	12	9	9	3
HPF11	4	2	7	0	10	0	12	0	5	9	3	2
HPF12	7	1	11	0	10	0	12	0	9	14	5	3
HPF13	10	0	8	0	8	2	12	0	15	18	2	2
HPF14	10	1	11	0	5	3	6	0	12	8	8	2
HPF15	10	0	5	0	6	1	7	0	13	10	3	1
HPF16	4	0	6	0	9	2	14	0	10	11	8	0
HPF17	9	0	7	2	5	0	10	0	NA	NA	7	2
HPF18	11	0	3	1	6	2	6	0	NA	NA	7	2
HPF19	5	2	6	1	3	1	8	0	NA	NA	5	1
HPF20	10	0	8	0	2	1	8	0	NA	NA	8	1
HPF21	3	0	5	0	10	3	7	0	NA	NA	8	0
HPF22	NA	NA	11	2	8	0	6	0	NA	NA	2	0
HPF23	NA	NA	5	0	5	2	1	0	NA	NA	NA	NA
HPF24	NA	NA	3	0	8	0	6	0	NA	NA	NA	NA
HPF25	NA	NA	NA	NA	4	2	7	0	NA	NA	NA	NA
HPF26	NA	NA	NA	NA	6	0	7	0	NA	NA	NA	NA
HPF27	NA	NA	NA	NA	7	1	5	0	NA	NA	NA	NA
HPF28	NA	NA	NA	NA	4	1	9	2	NA	NA	NA	NA
HPF29	NA	NA	NA	NA	9	5	19	0	NA	NA	NA	NA
HPF30	NA	NA	NA	NA	10	3	4	1	NA	NA	NA	NA
HPF31	NA	NA	NA	NA	3	4	5	1	NA	NA	NA	NA
HPF32	NA	NA	NA	NA	3	0	8	1	NA	NA	NA	NA
HPF33	NA	NA	NA	NA	NA	NA	8	0	NA	NA	NA	NA
HPF34	NA	NA	NA	NA	NA	NA	2	0	NA	NA	NA	NA
HPF35	NA	NA	NA	NA	NA	NA	8	0	NA	NA	NA	NA
HPF36	NA	NA	NA	NA	NA	NA	8	0	NA	NA	NA	NA
HPF37	NA	NA	NA	NA	NA	NA	7	0	NA	NA	NA	NA
HPF38	NA	NA	NA	NA	NA	NA	5	0	NA	NA	NA	NA
HPF39	NA	NA	NA	NA	NA	NA	6	0	NA	NA	NA	NA
HPF40	NA	NA	NA	NA	NA	NA	29	0	NA	NA	NA	NA
HPF41	NA	NA	NA	NA	NA	NA	6	0	NA	NA	NA	NA
HPF42	NA	NA	NA	NA	NA	NA	9	0	NA	NA	NA	NA
HPF43	NA	NA	NA	NA	NA	NA	29	0	NA	NA	NA	NA
HPF44	NA	NA	NA	NA	NA	NA	21	0	NA	NA	NA	NA
HPF45	NA	NA	NA	NA	NA	NA	5	0	NA	NA	NA	NA
HPF46	NA	NA	NA	NA	NA	NA	8	0	NA	NA	NA	NA
HPF47	NA	NA	NA	NA	NA	NA	26	0	NA	NA	NA	NA

	leuko A	para A	leuko A	para A	leuko B	para B	leuko C	para C	leuko D	para D	leuko E	para E
HPF48	NA	NA	NA	NA	NA	NA	35	0	NA	NA	NA	NA
HPF49	NA	NA	NA	NA	NA	NA	13	2	NA	NA	NA	NA
HPF50	NA	NA	NA	NA	NA	NA	3	0	NA	NA	NA	NA
	153	15	155	7	202	64	501	7	201	171	117	37

2.7 SL:13

Table 16: leuko/parasite data per HPF for the six readings of TBS SL:13.

	leuko A	para A	leuko A	para A	leuko B	para B	leuko C	para C	leuko D	para D	leuko E	para E
HPF1	0	0	0	0	0	0	0	0	0	0	0	0
HPF2	9	6	7	6	8	8	12	24	6	0	2	15
HPF3	10	10	9	5	4	4	7	14	8	6	6	21
HPF4	6	0	8	9	7	5	9	3	11	0	3	10
HPF5	7	15	1	7	4	8	8	9	4	8	3	20
HPF6	8	6	7	16	6	9	7	6	13	8	3	25
HPF7	8	6	3	15	4	10	4	6	13	9	8	16
HPF8	5	11	4	6	6	14	16	16	18	20	4	18
HPF9	7	11	5	10	8	16	10	9	14	4	3	17
HPF10	11	19	3	6	9	0	8	14	9	0	3	22
HPF11	3	7	10	10	15	8	8	0	9	8	6	15
HPF12	11	7	12	15	3	12	4	19	8	0	8	17
HPF13	12	0	7	12	6	0	8	19	4	19	8	18
HPF14	8	16	5	7	6	25	7	15	6	12	NA	NA
HPF15	6	5	10	14	2	3	5	12	13	11	NA	NA
HPF16	6	18	6	3	4	12	7	6	11	13	NA	NA
HPF17	9	11	11	7	9	7	12	3	15	15	NA	NA
HPF18	7	3	6	8	12	13	11	2	11	14	NA	NA
HPF19	5	0	11	9	8	7	10	4	11	19	NA	NA
HPF20	7	6	10	15	11	7	3	7	9	12	NA	NA
HPF21	7	0	8	15	13	11	3	9	9	20	NA	NA
HPF22	7	1	7	8	7	11	10	6	NA	NA	NA	NA
HPF23	6	4	NA	NA	10	3	12	2	NA	NA	NA	NA
HPF24	1	8	NA	NA	8	17	NA	NA	NA	NA	NA	NA
HPF25	12	5	NA	NA	12	0	NA	NA	NA	NA	NA	NA
HPF26	7	1	NA	NA	NA	NA	NA	NA	NA	NA	NA	NA
HPF27	4	1	NA	NA	NA	NA	NA	NA	NA	NA	NA	NA
HPF28	2	7	NA	NA	NA	NA	NA	NA	NA	NA	NA	NA
HPF29	4	5	NA	NA	NA	NA	NA	NA	NA	NA	NA	NA
HPF30	4	4	NA	NA	NA	NA	NA	NA	NA	NA	NA	NA
HPF31	6	6	NA	NA	NA	NA	NA	NA	NA	NA	NA	NA
	205	199	150	203	182	210	181	205	202	198	57	214

2.8 SL007

Table 17: leuko/parasite data per HPF for the five readings of TBS SL007.

	leuko A	para A	leuko A	para A	leuko B	para B	leuko D	para D	leuko E	para E
HPF1	0	0	0	0	0	4	0	0	0	0
HPF2	4	0	11	1	10	3	26	0	10	0
HPF3	12	1	15	1	10	0	3	4	11	1
HPF4	12	0	15	1	9	1	21	3	8	0
HPF5	13	0	13	0	11	2	6	0	17	2
HPF6	14	0	13	1	7	2	11	9	10	1
HPF7	14	0	12	0	8	0	4	0	9	1
HPF8	10	3	19	0	8	3	14	7	12	2
HPF9	7	1	19	3	10	0	30	2	11	0
HPF10	16	0	15	2	10	0	28	1	11	1
HPF11	16	0	12	0	8	2	29	2	7	0
HPF12	10	0	16	0	9	0	12	0	14	1
HPF13	20	0	14	2	5	1	16	2	10	1
HPF14	12	0	11	1	8	0	16	0	18	0
HPF15	15	0	11	1	10	0	6	1	12	1
HPF16	12	0	16	0	9	1	12	0	9	0
HPF17	10	0	NA	NA	2	1	9	6	12	3
HPF18	20	0	NA	NA	7	0	NA	NA	11	0
HPF19	12	0	NA	NA	7	2	NA	NA	7	1
HPF20	19	1	NA	NA	9	1	NA	NA	23	4
HPF21	13	0	NA	NA	6	1	NA	NA	NA	NA
HPF22	12	0	NA	NA	12	2	NA	NA	NA	NA
HPF23	16	2	NA	NA	6	0	NA	NA	NA	NA
HPF24	16	1	NA	NA	8	4	NA	NA	NA	NA
HPF25	18	0	NA	NA	10	1	NA	NA	NA	NA
HPF26	20	0	NA	NA	5	0	NA	NA	NA	NA
HPF27	14	0	NA	NA	9	1	NA	NA	NA	NA
HPF28	17	0	NA	NA	NA	NA	NA	NA	NA	NA
HPF29	3	3	NA	NA	NA	NA	NA	NA	NA	NA
HPF30	20	0	NA	NA	NA	NA	NA	NA	NA	NA
HPF31	11	2	NA	NA	NA	NA	NA	NA	NA	NA
HPF32	18	0	NA	NA	NA	NA	NA	NA	NA	NA
HPF33	19	0	NA	NA	NA	NA	NA	NA	NA	NA
HPF34	18	0	NA	NA	NA	NA	NA	NA	NA	NA
HPF35	16	0	NA	NA	NA	NA	NA	NA	NA	NA
HPF36	12	0	NA	NA	NA	NA	NA	NA	NA	NA
HPF37	12	0	NA	NA	NA	NA	NA	NA	NA	NA
HPF38	12	0	NA	NA	NA	NA	NA	NA	NA	NA
	515	14	212	13	213	32	243	37	222	19

2.9 SL057

Table 18: leuko/parasite data per HPF for the six readings of TBS SL057.

	leuko A	para A	leuko A	para A	leuko B	para B	leuko C	para C	leuko D	para D	leuko E	para E
HPF1	0	0	0	0	0	0	0	0	0	0	0	0
HPF2	9	82	7	1	8	0	3	0	24	380	11	127
HPF3	14	35	3	11	6	0	18	141	NA	NA	8	116
HPF4	14	13	7	14	17	211	28	221	NA	NA	NA	NA
HPF5	10	41	15	89	NA	NA	NA	NA	NA	NA	NA	NA
HPF6	18	58	16	83	NA	NA	NA	NA	NA	NA	NA	NA
HPF7	NA	NA	18	40	NA	NA	NA	NA	NA	NA	NA	NA
	65	229	66	238	31	211	49	362	24	380	19	243
